# Point Mutations at a Key Site Alter the Cytochrome P450 OleP Structural Dynamics

**DOI:** 10.3390/biom12010055

**Published:** 2021-12-31

**Authors:** Linda Celeste Montemiglio, Elena Gugole, Ida Freda, Cécile Exertier, Lucia D’Auria, Cheng Giuseppe Chen, Alessandro Nicola Nardi, Gabriele Cerutti, Giacomo Parisi, Marco D’Abramo, Carmelinda Savino, Beatrice Vallone

**Affiliations:** 1 Institute of Molecular Biology and Pathology, CNR c/o Department of Biochemical Sciences “A. Rossi Fanelli”, University of Rome, Sapienza, P.le A. Moro 5, 00185 Rome, Italy; lindaceleste.montemiglio@cnr.it (L.C.M.); cecile.exertier@uniroma1.it (C.E.); 2 Department of Biochemical Sciences “A. Rossi Fanelli”, University of Rome, Sapienza, P.le A. Moro 5, 00185 Rome, Italy; elena.gugole@uniroma1.it (E.G.); ida.freda@uniroma1.it (I.F.); lucia.dauria@ibs.fr (L.D.); gc2695@columbia.edu (G.C.); 3 Department of Chemistry, University of Rome, Sapienza, P.le A. Moro 5, 00185 Rome, Italy; giuseppe.chen@uniroma1.it (C.G.C.); alessandronicola.nardi@uniroma1.it (A.N.N.); marco.dabramo@uniroma1.it (M.D.); 4 Center for Life Nano & Neuro-Science, Fondazione Istituto Italiano di Tecnologia, IIT, 00185 Rome, Italy; giacomo.parisi@iit.it

**Keywords:** cytochrome P450, OleP, site directed mutagenesis, X-ray crystallography, P450 structural dynamics, MD simulation, conformational space

## Abstract

Substrate binding to the cytochrome P450 OleP is coupled to a large open-to-closed transition that remodels the active site, minimizing its exposure to the external solvent. When the aglycone substrate binds, a small empty cavity is formed between the I and G helices, the BC loop, and the substrate itself, where solvent molecules accumulate mediating substrate-enzyme interactions. Herein, we analyzed the role of this cavity in substrate binding to OleP by producing three mutants (E89Y, G92W, and S240Y) to decrease its volume. The crystal structures of the OleP mutants in the closed state bound to the aglycone 6DEB showed that G92W and S240Y occupied the cavity, providing additional contact points with the substrate. Conversely, mutation E89Y induces a flipped-out conformation of this amino acid side chain, that points towards the bulk, increasing the empty volume. Equilibrium titrations and molecular dynamic simulations indicate that the presence of a bulky residue within the cavity impacts the binding properties of the enzyme, perturbing the conformational space explored by the complexes. Our data highlight the relevance of this region in OleP substrate binding and suggest that it represents a key substrate-protein contact site to consider in the perspective of redirecting its activity towards alternative compounds.

## 1. Introduction

Cytochrome P450s (P450s) comprise a huge superfamily of ubiquitous heme-thiolate enzymes. Despite sharing a common fold, P450s catalyze different types of regio-, chemo-, and stereospecific reactions on a vast variety of structurally unrelated compounds [1,2].

Notwithstanding the wide substrate versatility, substrate binding to P450s is a finely regulated event. Regulation is achieved through enzyme-substrate interactions established within the active site that often activate conformational transitions. The structural dynamics of P450s mainly consist of the rearrangements of specific secondary structural elements that close the active site around the substrate, promoting the displacement of the axial water ligand from the heme iron that switches to the high spin-state, and minimizing the access to the external environment [3]. Productive binding occurs when the architecture and the chemistry of the catalytic chamber fit the chemistry and the geometry of the substrate. The overall catalytic efficiency of the enzyme depends on the frequency of the achievement of this state.

The 8.8a-oleandolide epoxygenase OleP, from *Streptomyces antibioticus*, is the P450 involved in the polyketide biosynthesis of the antibiotic oleandomycin [4,5]. It introduces an epoxide function to the C8-C8a of the macrolactone ring of two structurally different intermediates, namely the aglycone 8.8a-oleandolide (DEO) and the C3-mono-glycosylated L-olivosyl-8.8a-deoxyoleandolide (L-O-DEO) [6].

Substrate binding to OleP is coupled to a large structural transition, which originates from a small ensemble of short-range contacts established in the active site of the enzyme. This activates an intramolecular cascade of interactions that affect the overall protein structure [7,8]. The subsequent open-to-closed structural rearrangement allows the enzyme to achieve the catalytically competent conformation. Indeed, the crystal structures of the substrate-bound OleP in the open and the closed conformation revealed that the typical signatures of a P450 productive substrate binding are observed only in the closed state: (i) The C8-C8a bond of the substrate is oriented towards and at about 4 Å from the heme iron; (ii) the heme is pentacoordinated, due to the displacement of the sixth heme-iron coordinating water molecule; (iii) the I helix bends and breaks the helical geometry at its center forming a cleft crucial for the proton shuttle delivery process [9]; (iv) the active site is closed, thus limiting the possibility for water to enter from the bulk during the catalytic reaction [10].

**Figure 1:**
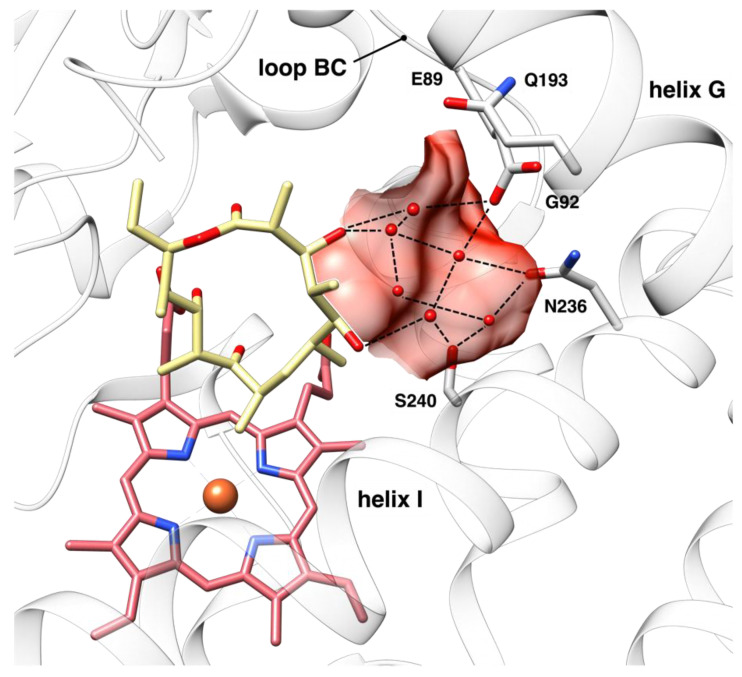
The solvent cavity. Close up view of the active site of OleP bound to 6DEB in the closed state (light grey, ribbon representation, pdb code 5MNS) [8]. The solvent cavity is highlighted and shown as a red surface. Secondary structural elements and the main residues (shown as sticks) that line the cavity are labelled. Water molecules are shown as red spheres. Hydrogen bonds are represented as dashed lines. Heme and 6DEB are shown in red and khaki sticks, respectively.

Similarly to other bacterial P450s, the central portion of OleP I helix plays a crucial role in substrate sensing and in the correlated structural changes. More precisely, the substrate interaction with residues at the conserved A/G_n−1_-G_n_-XX-T_n+3_ motif at the center of the helix, induces its bending [11]. This, in OleP, allows the formation of direct or indirect contacts between the substrate and residues at the N-terminus of the helix, namely N236 and S240, that might trigger the movement of the F/G unit, thus contributing to the overall closure efficiency [8,10]. In the crystal structure of OleP bound to the aglycone intermediate, these interactions are mediated by solvent molecules that accumulate in a small polar pocket, the so-called “solvent cavity”, that forms upon enzyme closure [8,10]. The solvent cavity is delimited by the C-terminus of the BC loop (residues 85–93), the N-terminus segments of the helices G (residues 185–197) and I (residues 232–243), including N236 and S240, and the substrate itself (Figure 1). This cavity is distant from the iron and is located in the vicinity of the site that hosts substrate aglycone moiety. Given the intrinsic flexibility of a linkage that is mediated by dynamic solvent molecules, the structural transition upon aglycone binding to the closed and catalytically competent conformation occurs only for a minor population of OleP [8,10]. On the other hand, in silico and crystallographic analyses suggested that when L-O-DEO is bound, the solvent cavity hosts its glycosyl unit, that directly interacts with N236 and S240 on the N-terminus of the I helix. The direct contact with these two residues would more efficiently trigger the active site closure, inducing the displacement of solvent molecules from the active site, thus avoiding or limiting spurious reactions [10].

Therefore, enzyme-substrate contacts that are established within the solvent cavity may constitute the efficient trigger required for the conformational change, stabilizing the closure, and locking OleP in the high-spin state conformation.

In this work, we investigated the role of the solvent cavity in substrate binding to OleP by introducing mutations at positions lining this region and placed on the BC loop and on the I helix, namely E89 and G92 (BC loop) and S240 (I helix). Mutants have been designed based on the previously reported structure of the closed aglycone-bound OleP, with the aim to decrease the volume of the cavity where the mutated residue could still accommodate their hydrophobic side chain, without hampering aglycone substrate binding. This was achieved by substitutions of E89 by tyrosine, G92 by tryptophan, and S240 by tyrosine. We performed equilibrium binding experiments and a structural analysis based on X-ray crystallography and MD simulations, using the substrate analog 6-deoxyerythronolide B (6DEB). The effects of the introduced mutations on the aglycone binding properties, on the structural organization of the active site, and on the conformational plasticity of OleP-6DEB complex are examined and discussed.

## 2. Materials and Methods

### 2.1. Site-Directed Mutagenesis, Expression, and Purification

Recombinant His-tagged OleP mutants E89Y, G92W, and S240Y were produced using the Quick Change Lightning Kit (Agilent Technologies, Santa Clara, CA, USA) and Q5 Site-Directed Mutagenesis Kit (NEB, Ipswich, MA, USA), following the manufacturer’s instructions and using the pET28b(+)-OleP wild type construct as a template.

**Figure 2:**
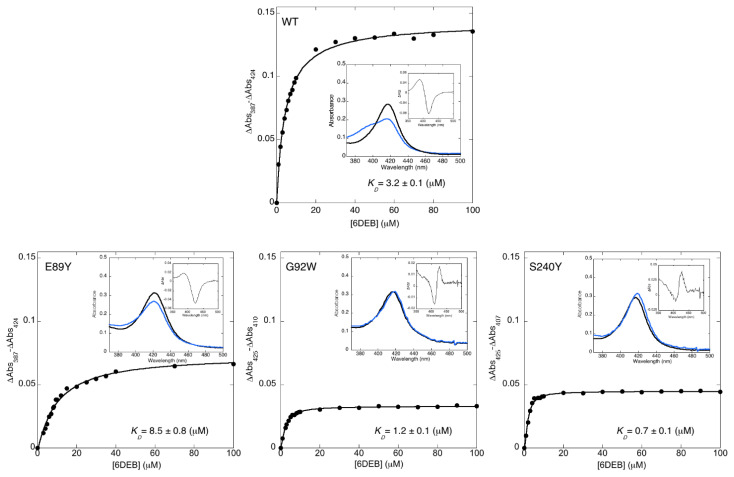
Binding of 6DEB to OleP mutants. Titration plots with hyperbolic fits, from which the dissociation constants (*K_D_*) for 6DEB were derived. Data refer to the absorbance monitored at 298 K using a constant concentration of OleP in 50 mM Hepes and 200 mM NaCl, pH 7.5. Insets: Spectral changes induced by 6DEB in the WT and mutants OleP, respectively. Black and light blue spectra were recorded before and after equilibrium titrations, with the final 6DEB concentration of 100 µM. Right small insets are difference spectra recorded after the addition of 6DEB (final concentration 100 µM). OleP wild type-6DEB binding data are taken from [10].

All of the mutants were expressed in *E. coli* BL21 (DE3) and purified as previously described [7]. Homogeneity and monodispersion were assessed by SDS-PAGE and gel filtration chromatography using a 10/400 column packed with Tosoh HW-55S (Tosoh Bioscience) resin in 20 mM Tris·HCl, 200 mM NaCl, pH 8.0 at 293 K. All of the three mutants were eluted as monomers. Preliminary optical characterization revealed that G92W and S240Y mutants show a Soret band maximum at 417 nm, as reported for the OleP wild type, whereas the E89Y mutant displays a slight red shift of the maximum absorbance peak at 421 nm (Figure 2). All of the spectral determinations were made using a Jasco V-650 spectrophotometer.

### 2.2. Equilibrium Binding Analyses

All of the OleP mutants were tested for binding to the substrate analogue 6-deoxyerythronolide B (6DEB), kindly supplied by Biotica Technology Ltd., Cambridge, UK. The 6DEB stock solution (26 mM) was prepared in dimethyl sulfoxide (DMSO, St. Louis, Sigma Aldrich, MO, USA). OleP mutants were titrated at 298 K at a concentration of 2.5 μM with 6DEB ranging from 0 to 100 μM in a buffer containing 50 mM Hepes and 0.2 M NaCl, pH 7.5, thus keeping DMSO below 1%. UV visible spectra (200–820 nm) were recorded after each substrate addition and the appropriate blank was subtracted. The change in absorbance difference between the peaks and troughs ((ΔA_387_ − ΔA_424_) for the OleP wild type and E89Y; (ΔA_425_ − ΔA_410_) for G92W and S240Y), were plotted against the substrate concentration (Figure 2).

The dissociation constant (*K_D_*) was estimated using the Kaleidagraph software package. A nonlinear regression analysis was applied using the quadratic equation:

$${\Delta \mathrm{AU}}_{obs}=\frac{{\Delta \mathrm{AU}}_{max}}{2}\left(\left[E_{0}\right]+\left[S_{0}\right]+K_{D}-{\left({\left(\left[E_{0}\right]+\left[S_{0}\right]+K_{D}\right)}^{2}-4\left(\left[E_{0}\right]\left[S_{0}\right]\right)\right)}^{\frac{1}{2}}\right)$$

where ΔAU*_obs_* is the absorbance difference, ΔAU*_max_* is the maximum absorbance difference extrapolated to the infinite ligand concentration, and [E_0_] and [S_0_] are the enzyme and the substrate analytical concentrations, respectively. All of the binding experiments were performed in duplicate.

To monitor the effect of the ionic strength on the spin-state equilibrium in OleP mutants bound to 6DEB, 1.5 μM protein in the presence of 150 μM of 6DEB was titrated at 298 K with an increasing concentration of sodium formate, ranging from 0 to 3.5 M, in a final volume of 750 μL of 50 mM Hepes and 200 mM NaCl, pH 7.5. The absorption shift of the Soret peak was followed by collecting UV-visible spectra (200–800 nm) after each substrate addition, and the appropriate blank was subtracted.

All of the spectral determinations were made using a Jasco V-650 spectrophotometer controlled by a Jasco programmable Peltier element (EHC-716) and a HP-8453 spectrophotometer equipped with a Peltier temperature control accessory (HP-S9090A).

### 2.3. Protein Crystallization, Data Collection, and Data Reduction

Purified E89Y, G92W, and S240Y OleP mutants (in 20 mM Tris·HCl and 200 mM NaCl, pH 8.0) were co-crystallized at a concentration of 19 mg/mL (0.45 mM) with 6DEB at saturating concentration (1 mM), using the hanging-drop vapor-diffusion method at 293 K. Crystals of the three mutants were obtained in sodium formate, as detailed in Table 1, in a drop containing protein-ligand complex and precipitant solution in 1:1 ratio. The streak seeding technique was applied for G92W-6DEB and E89Y-6DEB to improve the crystal diffraction quality.

Crystals of E89Y-6DEB and G92W-6DEB were mounted on nylon loops and cryoprotected by immersion in a solution containing the crystallization buffer supplemented with 20% *v*/*v* glycerol prior to flash-freezing in liquid nitrogen, while S240Y-6DEB crystals were frozen without the cryo-protectant.

X-ray diffraction data were collected at 100 K on a PILATUS detector (Dectris, Baden-Dättwil, Switzerland) at ELETTRA synchrotron (Trieste, Italy). Data were indexed, scaled, and integrated using XDS package [12] and Aimless [13]. Crystal parameters and collection statistics are reported in Table 1.

### 2.4. Structure Determination and Model Refinement

Regarding all of the OleP mutants bound to 6DEB, initial phases were calculated by molecular replacement using MOLREP [14] in the CCP4 package (version 7.0.071). The coordinates of a single closed monomer of OleP-6DEB (pdb code: 5MNS [8]) deprived of ligands, waters, and ions were used as a search model. Six monomers (A–F) were found in the asymmetric unit of the three structures. Cycles of refinement and model building were performed with Refmac5 in the CCP4 suite [15] and Coot 0.8.9.1 [16,17], using the |F_o_|–|F_c_| map contoured at ±3 σ and the 2|F_o_|–|F_c_| at ±1 σ. Five percent of the reflections were excluded from refinement and used for the R_free_ calculation [18]. The geometric quality of the final models was assessed with MolProbity [19].

Depending on the monomer, the first 6–14 N-terminal residues are missing in the models due to the insufficient electron density given the intrinsic flexibility of the tract, except for monomer C of G92W-6DEB and monomer B of S240Y-6DEB structures, whose electron density allowed for the first time the reconstruction of the full N-terminal tail (Figure S1). Indeed, in G92W-6DEB, the tail of monomer C fortuitously enters the solvent channel between the symmetric monomers A and D, establishing lattice contacts with residues at the BC loop and G helix of the former and B and L helices and the β-harpin β3 of the latter chain. Those interactions stabilize its position. In S240Y-6DEB, the N-tail of monomer B is stabilized by crystal contacts established with residues at the BC loop, helices C and D, and the β-harpin β4 of monomer C of the asymmetric unit and with residues at the BC loop and at the N-terminus of G helix of the symmetric monomer E.

The similarity among copies of the same mutant is very high, the overall root-mean-square deviation (rmsd) on alpha carbons (Cα) ranging from 0.6 to 0.8–0.9 Å using monomer C as a reference. For this reason, for the structural analysis herein discussed, we chose monomer C in all of the reported structures as a representative for each OleP variant, which is defined by the best electron density and lower average B factor. If not otherwise stated, all of the structural features described in this work are found in all of the monomers, when allowed by the quality of the corresponding electron density map. All of the final statistics of the data collection and model refinement are reported in Table 1. All of the figures were produced with Chimera [20]. The atomic coordinates and structure factors of E89Y-, G92W-, and S240Y-6DEB complexes have been deposited in the Protein Data Bank (Accession numbers: 7Q6R, 7Q89, and 7Q6X, respectively).

### 2.5. Molecular Dynamic (MD) Simulations

All of the atoms molecular dynamic simulations of OleP WT, G92W, and E89Y mutants in water solution have been performed by means of the Gromacs software package [21]. The starting structure of the wild type OleP-6DEB complex in the closed state has been taken from the X-ray deposited data (pdb code: 5MNS) [8], while the G92W-6DEB and E89Y-6DEB structures were taken from our crystallographic data, using monomer C in all of the cases. The systems have been solvated using the SPC water model in a box of 8.707 nm and then minimized with the steepest descent algorithm. After short equilibration phases of ~20 ns, MD simulations have been performed for 400 ns using a time step of 2 fs. The temperature has been fixed at 300 K using the v-rescale algorithm.

The electrostatic interactions have been evaluated via the PME method. The cutoff radius for both electrostatic and van der Waals interactions was set to 1.1 nm. The analysis of the structural-dynamical fluctuations has been performed by the calculation of root mean square fluctuations (RMSFs).

The essential dynamics analysis [22,23], a technique able to extract the principal molecular motions, has been performed on the trajectory formed by concatenating the trajectories of the three mutants to obtain a description of the conformations sampled by the three proteins on a common conformational space. This procedure has been extensively applied in previous works dealing with the comparison of MD trajectories of different proteins [24,25,26].

The evaluation of the solvent cavity of the WT and the two mutants was performed by means of MDpocket software [27].

## 3. Results

### 3.1. Impact of Bulkier Substitutions at Residues E89, G92, and S240 to the Binding Properties of OleP

To assess how mutations at the solvent cavity affect the functional properties of OleP, we performed equilibrium binding experiments using 6DEB, monitoring the spin state shift of the heme iron that accompanies ligand binding to the P450 distal pocket. Binding of 6DEB to wild type OleP produces a low- to high-spin shift, which is responsible for the typical type I change of the low Soret absorption maximum from ~424 to ~387 nm in the UV-visible spectrum (Figure 2) [20].

Therefore, the change in heme absorption was measured at 298 K as a function of substrate concentration for all of the mutants, while the enzyme concentration remained fixed. The observed binding curves were satisfactorily fitted with a hyperbolic equation, the estimated *K_D_* are reported in Figure 2.

Similarly to the wild type, binding to E89Y causes a type I spectral change. This mutation marginally alters the affinity for the aglycone, but it halves the amplitude of the spectral transition, thus indicating the presence at the equilibrium of a minor population of enzyme, which is converted to the high spin state by 6DEB binding with respect to OleP wild type [28]. Binding of 6DEB to G92W and S240Y produced a spectroscopic shift different to what was expected, with a peak at 425 nm and a trough at about 410 nm, which is typical of Type II ligands, i.e., binders with inhibitory behavior (Figure 2) [7,29]. In these cases, the estimated *K_D_* values revealed some increase in 6DEB affinity with respect to the wild type.

### 3.2. Mutations at E89, G92, and S240 with Bulky Aromatic Sidechain Do Not Alter the Global Structure of OleP

To analyze how mutations are accommodated in the OleP active site, we determined the crystallographic structures of E89Y, G92W, and S240Y in complex with 6DEB.

Crystals were obtained in the C2 space group, diffracted at resolutions between 2.1 and 2.7 Å, and contained six monomers in the asymmetric unit.

The overall structure of the complex of 6DEB with mutants resembles the wild type complex obtained under similar crystallization conditions, characterized by high ionic strength [8]. In all of the cases, the six chains identified in the asymmetric unit show the enzyme bound to the substrate, adopting a closed conformation (Figure S2). As a quantitative measure of the similarity, the rmsd of the Cα with respect to the wild type was determined, showing differences below the intrinsic error of structural determination. The major ones (rmsd ≥ 0.8 Å) are confined to the HI loop (residues 220–230) and to the regions around mutations in the case of G92W and S240Y (Figure S2).

All of the structural features typical of the wild type OleP bound to 6DEB in the closed state are maintained in the mutants. More in details, (i) the closure of the active site involves a pronounced structural reorganization of the F, G, and I helices, of the HI loop over the heme pocket and less pronounced of the BC loop if compared to OleP-6DEB in the open state [8]; (ii) the active site is occupied by one molecule of 6DEB that binds almost perpendicularly to the tetraporphirinic ring, placing the C8-C8a bond, target of OleP, parallel to the heme iron and at a distance of about 4 Å; (iii) the position adopted by the aglycone substrate allows the displacement of the sixth coordinating water molecule, which is in the penta-coordination state; (iv) the geometry of I helix is altered at the level of the 244–248 turn in the middle of the helix, which causes the formation of the small catalytic cleft that is considered crucial for the enzymatic reaction (Figure S3).

Therefore, in the presence of E89Y, G92W, and S240Y mutations, OleP still binds 6DEB and, in the crystallization conditions explored, it adopts a closed and catalytically competent conformation.

### 3.3. Structural Reorganization of the Active Site in E89Y, G92W, and S240Y Bound to 6DEB

The presence of the E89Y, G92W, and S240Y mutations in the OleP active site has been initially assessed by calculating the omit map for the relative residues. A clear bulkier electron density, in correspondence of the mutated sites, was observed.

**Figure 3:**
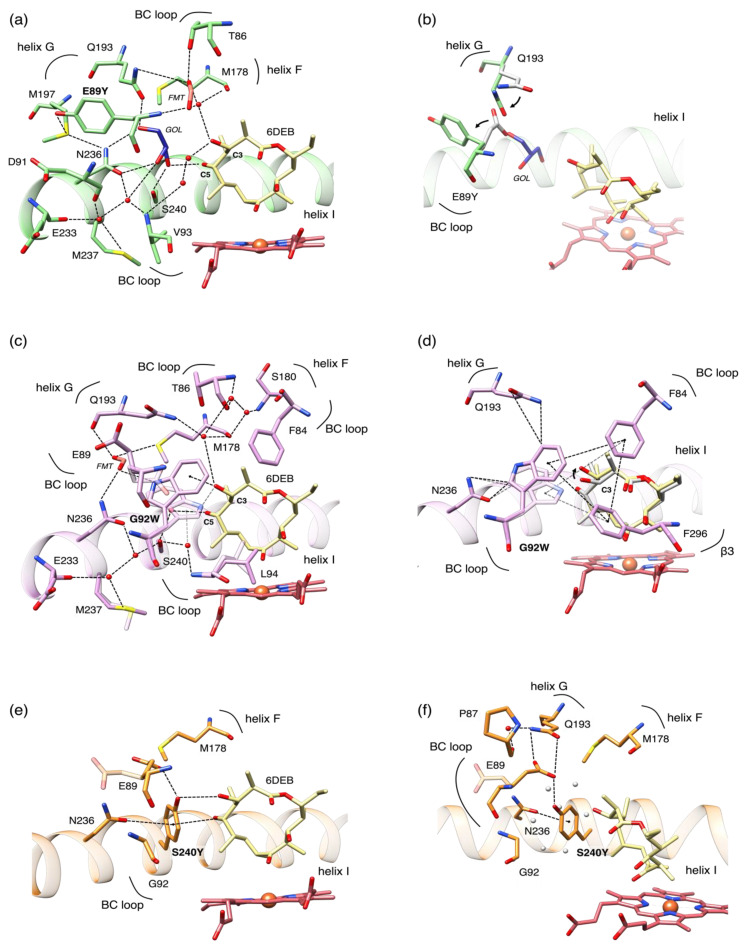
Structural changes in the solvent cavity of OleP mutants. Close up view of the changes in the solvent cavity induced by mutations at residues E89Y (panels (**a**,**b**)), G92W (panels (**c**,**d**)), S240Y (panels (**e**,**f**)). Right panels show the cavity in the orientation that better renders the differences between the mutant and wild type. Residues involved in contacts within 4 Å of distance are shown as sticks and labelled. Dashed lines indicate hydrogen bonds or π-interactions. Double conformations are reported, showing the less occupied conformer in transparency. Solvent molecules of glycerol (GOL) and formate ion (FMT) are labelled in italic. Heme and 6DEB are represented as red and khaki sticks, respectively; waters found in the structure of mutants are in red spheres, while those found in the wild type structure are in white spheres.

The analysis of the crystal structures revealed that all of the mutations are accommodated with minimal changes of the OleP active site. As predicted, the substitution of G92 with a tryptophan and S240 with a tyrosine resulted in filling the solvent cavity, which is occupied by the bulky aromatic sidechains. This reduces to some extent the volume of the active site in both mutants, which is ~606.7 Å^3^ in the wild type and ~582.5 and ~566.8 Å^3^ in G92W and S240Y, respectively (Figure 3 and Figure S4) [30]. A different scenario was found in the E89Y structure. The tyrosine side chain at position 89 is not located inside the cavity, but it rotates towards the bulk. In the wild type OleP, the glutamate 89 was found in a double conformation, one that exposed the residue towards the solvent (flipped-out), and one towards the polar cavity (flipped-in) [8]. Therefore, the substitution with a tyrosine stabilizes the flipped-out conformation, since we found the amino acid exposed to the solvent in all of the chains identified in the asymmetric unit. The effect of this structural change is a slight increase of the volume of the catalytic pocket with respect to the wild type, which has been measured to be 671.2 Å^3^ (Figure 3 and Figure S4).

The changes in the substrate-binding pocket induced by mutations do not alter the position and the conformation of 6DEB within the OleP active site, which is the same in all of the structures, as observed by the structural superposition (Figure S3b). All of the van der Waals interactions established with OleP residues present in the main cavity of the active site are preserved by mutations introduced at the solvent cavity. Those contacts involve residues located on the BC loop (M83, F84, L94), the F helix (M178, L179), the central portion of I helix (I243, A244, T248), the β-hairpin β3 (V291, A293, G294, S295, F296), and the β-hairpin β4 (L396, I397). The carbonyl-aromatic residue interaction established between the C1-carbonyl of 6DEB, F84 (BC loop), and F296 (β-hairpin β3) was also found (Figure S5) [8,10]. Conversely, mutations affect the structural organization of protein residues and solvent molecules within the solvent cavity in the active site together with the substrate-protein interactions established at that site (Figure S6). 

In E89Y, the rotation that orients the side chain of the residue on the opposite side to the heme binding pocket eliminates the water mediated contacts established by the E89 side chain. Similarly to the wild type structure, solvent molecules, including a formate ion, enter the cavity and mediate protein-substrate interactions (Figure 3a). Moreover, in E89Y, we identified one molecule of glycerol (GOL), used to cryoprotect the formed crystal, absent in the wild type, that occupies the position of the E89 sidechain and of two water molecules (Figure S6). In most cases, the positions adopted by solvent molecules in E89Y follow the pattern of waters found in the wild type complex (Figure S6), and favor the formation of indirect contacts between the C3 and the C5 hydroxyl groups of 6DEB with protein residues located on the BC loop (T86, E89Y, D91, V93), on the N-terminus of I helix (E233, N236, M237, S240), on F helix (M178) and G helix (Q193, M197) (Figure 3a). Therefore, in the presence of E89Y mutation, the effect of the stabilization of the flipped-out conformation of the residue is limited to minor structural changes within the solvent cavity: (i) A slight enlargement of the cavity; (ii) the rearrangement of Q193 on G helix that rotates about 45° in the direction of the place left by the carboxyl group at the Cδ of the glutamate side chain to compensate for its absence (Figure 3b). In this position, Q193 indirectly contacts N236 on the I helix through GOL.

In G92W and S240Y mutants, the bulkier and more hydrophobic groups are located in the cavity, causing, respectively, the partial and the total displacement of the solvent molecules and resulting in the formation of direct interactions between the enzyme and the substrate (Figure 3c–f).

In G92W, the tryptophan adopts a double conformation in conjunction with E89 (Figure 3c). In both conformations, W92 forms a direct π-interaction with the OH at the C3 of 6DEB. As a consequence, the C3-OH bond of the substrate rotates about 70° with respect to the wild type OleP-6DEB complex. In addition, one of the solvent molecules still found in the cavity bridges the contact between the hydroxyl moiety at C5 of 6DEB and S240 in the I helix. Of note, in one of the conformers adopted by W92, the methyl at the C2 of 6DEB is sandwiched between the aromatic side chains of W92 and F84, establishing an additional direct contact (Figure 3d).

In S240Y mutant, new direct interactions are formed between OleP and the substrate, namely a hydrogen bond between the hydroxyl group of Y240 and the OH at C3 of 6DEB and a long π-interaction (~4 Å) between the C5-OH of 6DEB and the aromatic ring of Y240 (Figure 3e). In this mutant, no solvent molecules were identified in the cavity as they were fully displaced by the side chain of the mutated residue (Figure 3f).

The insertion of a tryptophan in position 92 and of a tyrosine in position 240 also generate additional contacts within the protein structure, involving residues located in the active site of the enzyme. In G92W, we identified: (i) A T-stacking interaction between W92 and F84 (BC loop) and F296 (β-hairpin β3); (ii) an induced dipole-dipole type interaction between the aromatic ring of W92 and the δ-carbonyl group of N236 and the ε-amino group of Q193 (Figure 3d). In S240Y, we observed (i) a hydrogen bond formed between the γ-carboxyl group of E89 and the hydroxyl group of Y240; (ii) an induced dipole-dipole type interaction between the aromatic ring of W92 and the δ-carbonyl group of N236; iii) hydrophobic contacts between the Y240, M178 (F helix), and G92 (BC loop) (Figure 3f).

### 3.4. Mutations at the Solvent Cavity Alter the Conformational Dynamics of the Substrate-Bound OleP

In order to analyze the effect of mutations on the conformational dynamics of the closed-bound OleP, MD simulations were performed on the wild type OleP (pdb code: 5MNS) [8], E89Y, and G92W mutant closed structures. The RMSFs analysis shows similar patterns between the three systems. As expected, the residues that exhibit the larger fluctuations are located in the regions connecting the different protein elements with well-defined secondary structures.

**Figure 4:**
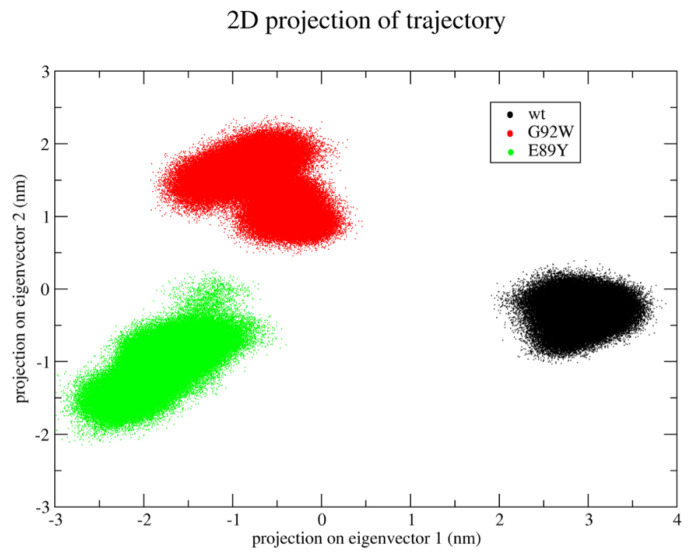
Differences in the accessible conformational space between the OleP E89Y and G92W mutants and the wild type. Trajectory projections on the essential subspace as defined by the first two eigenvectors obtained by means of essential dynamics analysis.

Differences in the accessible conformational space between the two OleP mutants and the wild type emerge from the essential dynamics analysis of the concatenated trajectory. In fact, the projections of the three trajectories on the essential subspace—as defined by the first two eigenvectors—clearly show that WT, G92W, and E89Y explore different regions of this subspace (Figure 4). In particular, the first eigenvector discriminates between the WT and the two mutants, whereas the second eigenvector is able to discriminate also between the two mutants.

Interestingly, the analysis of the eigenvalues associated to the calculated eigenvectors shows that the first two eigenvectors describe ~64% of the total variance observed along the MD trajectories, suggesting that they are able to properly describe the main protein motions (see Figure S7 for the eigenvalue spectrum).

The analysis of the components of these two eigenvectors shows that the essential protein motions are mainly due to specific regions of the proteins. In particular, the first eigenvector describes the coupled motions occurring in residues 78–94 (which include part of the BC loop), 131–158 (helices D and E), and 209–242 (which include helix H, the HI loop, and the N-terminus of the helix I).

The local analysis of the protein cavities as obtained by MD simulations show that in the G92W mutant the distance distribution maximum between the heme-iron and substrate is slightly higher than the WT and E89Y mutant (Figure S8). This might justify the uncomplete shift towards the high-spin state of the G92W mutant observed by means of UV-visible spectroscopy. Furthermore, the calculation of the solvent cavity volumes as provided by MD trajectories confirms the analysis of the crystal structure, where an increase of the E89Y cavity volume with respect to the WT and G92W has been observed (Figure S9).

These results highlight the relevant effects on the structural-dynamical behavior due to single mutations, which are able to shift the accessible conformational space, at both local and global level, within the ns time-scale.

## 4. Discussion

The mechanism that elicits the productive structural response of a P450 upon substrate binding is characteristic of each specific member of the family and it depends on each enzyme’s intrinsic structural plasticity and on the nature of the physiological substrate. Its full understanding is very relevant in the perspective of rationally exploiting the unique and versatile catalytic properties of each P450 on alternative compounds for diverse biotechnological applications [31,32,33].

In this work, we analyzed the case of the bacterial cytochrome P450 OleP. The substrate versatility of this enzyme, that naturally catalyzes the epoxidation of aliphatic carbons of macrolidic substrates, has lately attracted the pharmaceutical research attention given its capability to hydroxylate steroid compounds [34,35,36]. More recently, Grobe et al. have successfully produced an OleP variant which is able to regioselectively convert lithocolic acid to ursodexosycholic acid (UDCA), an expensive drug used in the therapy of cystic fibrosis and of primary biliary cholangitis [37].

Given the emerging interest towards the OleP enzymatic activity, in this study we have extended the characterization of the substrate binding properties of this P450, analyzing the role of a small polar cavity of the active site of the protein (the solvent cavity), that forms upon OleP closure, in the substrate binding process and in the subsequent structural rearrangements that lead to the productive substrate-bound state. We produced three OleP mutants to introduce a steric perturbation to the cavity. We substituted residues lining the cavity, namely E89, G92 (BC loop), and S240 (I helix), with bulky amino acids to fill its volume.

The crystal structure of the mutants, obtained in conditions that are known to stabilize the closed conformation of OleP, i.e., high ionic strength, confirmed that substitutions at G92 and S240 with a tryptophan and a tyrosine, respectively, occupied the cavity. Within the solvent cavity, the aromatic side chain of S240Y and G92W form additional direct and directional contacts with the substrate and with other residues within the distal pocket, inducing the partial or the total removal of solvent molecules from that region. Conversely, E89Y mutation directs the amino acid side chain towards the outer environment, increasing the empty volume, as shown by both crystal structure and MD simulation analyses. Equilibrium binding experiments with 6DEB performed on the latter mutant showed only a slight decrease in the binding capacity with respect to the wild type enzyme. On the contrary, the presence of G92W and S240Y mutations alters the binding properties of OleP to 6DEB. Indeed, the type I spectral change observed upon 6DEB binding, is converted to a type II shift of the UV-visible heme Soret peak, with some increase in affinity. This spectroscopic behavior is typical of a ligand that upon binding induces the stabilization of the hexacoordinate low spin state of the P450 heme iron. This, in our system, might correspond to the stabilization of a complex in which 6DEB is too distant from the heme iron to induce the displacement of the sixth coordinating water molecule, whose strong ligand field appears further enhanced by substrate binding. This state in OleP has been previously described as the open-bound form [8,10]. On the basis of these observations, we hypothesize that in those mutants the substrate binding event fails triggering protein closure. Therefore, in G92W and S240Y, the presence of a bulky and hydrophobic residue in the place of an empty and polar cavity reduces the structural dynamics of the OleP-6DEB complex. These mutations seem to alter the equilibrium between open and closed forms, further stabilizing the open conformation, at low ionic strength experimental conditions (close to the physiological one, I ~0.2 M). On the other hand, in crystals obtained at higher ionic strength (I ~4.4 M), we observed only closed monomers of the complex for all mutants.

**Figure 5:**
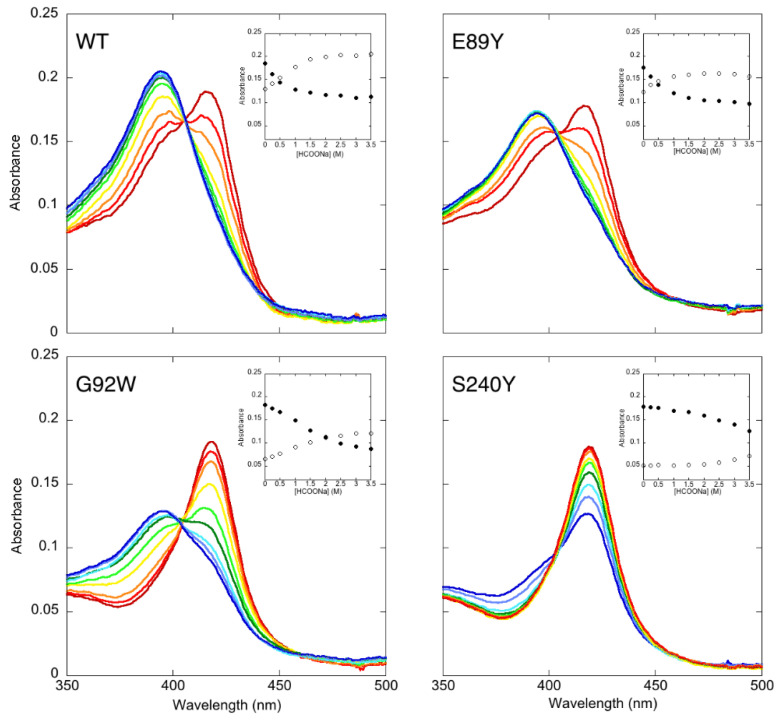
Effect of the ionic strength on the spin-state equilibrium in OleP mutants bound to 6DEB. UV-visible absorption spectra of OleP WT (2 µM) and mutants (1.5 µM) in the presence of 150 µM 6DEB collected upon addition of increasing concentration of sodium formate at 298 K, in 50 mM Hepes, and 200 mM NaCl, pH 7.5. Rainbow colors indicate the direction of the absorption changes observed changing from 0 (dark red) to 3.5 M (blue) sodium formate (light red = 0.25 M, orange = 0.5 M, yellow = 1 M, light green = 1.5 M, dark green = 2 M, cyan = 2.5 M, purple = 3 M). Relative insets: Absorbance intensities of OleP WT and mutants bound to 6DEB as a function of total sodium formate concentration are reported. Data refer to the absorbance monitored at 419 nm (full dots) and at 387 nm (empty dots). OleP wild type binding data are taken from [10].

In light of this evidence and with the aim to demonstrate an effect of G92W and S240Y mutant on the structural dynamics of OleP, we examined the change of the UV-visible spectra of all OleP mutants bound to 6DEB as a function of sodium formate concentration (Figure 5). Increasing the ionic strength induces an increase in the absorbance at 387 nm and a decrease at 419 nm in all mutants, which is indicative of the conversion to the high-spin state as a consequence of the displacement of the axial water ligand from the heme iron (Figure 5).

Notably, the heme reactivity response to the change of ionic strength conditions depends on the mutation. Indeed, while the effect of the ionic strength on the spin state equilibrium of the wild type and E89Y is comparable, showing the total low-to-high spin shift at I = 2.5–3 M, in G92W and S240Y mutants the shift is not complete even at the highest ionic strength conditions tested (I = 3.5 M). In particular, the S240Y mutant bound to 6DEB only starts shifting the equilibrium towards the high spin at I ≥ 3 M. The different degree of response to ionic strength conditions may arise from a different effect of the mutations at position 92 and 240 on the structural dynamics of the protein. In agreement with this observation, the MD simulations performed on the WT, E89Y, and G92W closed complex clearly show that the effect of the mutations is to alter their accessible conformational space. Indeed, the principal components analysis on the MD trajectories revealed that the regions sampled in the common essential subspace of the three OleP forms are different. In particular, the first eigenvector, i.e., the principal motion direction, can discriminate between WT and the two mutants, whereas the second eigenvector is able to further discriminate between the G92W and the wild type and E89Y complexes (the eigenvector components as well as their associated eigenvalues are reported in Supplemental Material). The G92W mutation is also able to alter the arrangement of the substrate with respect to the heme iron. In addition to these results, the ionic strength dependence of the spin-state equilibrium data suggests a further effect of G92W and S240Y mutations on the open-bound state that alters the overall OleP-6DEB dynamics. We inspected the open OleP-6DEB wild type structure (PDB code 5MNV, [8]) in which G92W and S240Y mutations were modeled. Since the magnitude of the motions experienced by the BC loop (aa 83–100) in the open-to-closed transition is small (~3 Å), it is plausible that direct contacts of W92 with the substrate and the protein may be already formed in the open state. Indeed, we found that W92 may simultaneously contact I243 on the I helix and 6DEB, forming a triangle of van der Waals interactions that compacts and reduces the open structure flexibility and that provides some stabilization of the open complex with respect to the unbound form (Figure S10a). In the open structure of S240Y-6DEB, the tyrosine would be far from the distal pocket and the substrate, covering the N-terminus of I helix. The superposition of the modeled open structure onto the crystallographic closed complex suggests that the presence of a tyrosine at position 240 would jeopardize the enzyme closure since its repositioning could lead to a collision with the BC loop at residues 91–93 (Figure S10b).

This qualitative structural analysis suggests that G92W and S240Y mutations may constitute a wedge that alters the intrinsic flexibility of OleP, increasing the free energy barrier for the open-to-closed transition. Therefore, at physiological ionic strength conditions, substrate binding to G92W and S240Y mutants cannot trigger the structural transition that induces the displacement of the water molecule from the sixth coordination position of the iron and the spin-state shift, not even on a partial population of the enzyme, as it occurs in the case of wild type and E89Y variants (Figure 5). We cannot exclude that S240Y and G92W mutations could also alter the conformational space of the unbound protein.

All considered, our data suggest that filling the solvent cavity with bulky residues is not a viable strategy in future engineering work on the P450 OleP. The consequent alteration of the conformational dynamics, that emerges from the data herein discussed, seems to induce the accumulation at equilibrium of the non-productive form of the enzyme-substrate complex. This is in contrast to other P450 enzymes, such as the CYP102A1 from *Bacillus megaterium*, where bulkier mutations at a site topologically homologous to the solvent cavity of OleP enhanced substrate binding affinity and the catalytic efficiency of the enzyme towards fatty acid substrates [38]. As a further observation, since the solvent cavity represents a region of the active site which is crucial for establishing productive substrate-OleP interactions that trigger the closure, it is important that its volume remains available to allow for the substrate to contact residues at the N-terminus of the I helix. Therefore, the choice of alternative substrates should fall on those whose chemical structure is potentially able to enter the cavity and interact with the residues at that site.

In conclusion, our data point to a role for the solvent cavity as the pivot of the OleP dynamics. In the perspective of further engineering the P450 OleP for future sustainable enzymatic production processes, it must be considered that the intrinsic flexibility of this site must be preserved to allow for the proper protein movements, which are productively triggered by substrate binding.

## Figures and Tables

**Table 1:** Data collection, refinement, statistics, and validation. The highest-resolution shell is shown in parentheses.

Data Collection	E89Y-6DEB	G92W-6DEB	S240Y-6DEB
PDB ID	7Q6R	7Q89	7Q6X
Crystallization conditions	4.4 M HCOONa (20% glycerol)	4.4 M HCOONa (20% glycerol)	4.4 M HCOONa
Space group	C2	C2	C2
Unit cell (Å, °)	a = 248.13,	a = 247.96,	a = 248.62,
	b = 111.12,	b = 110.23,	b = 110.36,
	c = 160.54,	c = 159.55,	c = 160.05,
	β = 129.7	β = 129.7	β = 129.6
Resolution (Å)	50.0–2.44 (2.59–2.44)	50.0–1.96 (2.08–1.96)	55.0–2.50 (2.65–2.50)
Total measurements	837,853	1,528,540	781,652
Unique reflections	241,832	462,752	225,386
Completeness (%)	98.4 (97.2)	98.8 (97.8)	99.2 (99.1)
Multiplicity	3.5 (3.2)	3.30 (3.1)	3.5 (3.4)
R_merge_^a^ (%)	5.0 (62.0)	8.0 (180.7)	20.4 (376.0)
CC1/2 (%)	99.9 (75.7)	99.7 (32.3)	99.2 (10.1)
<I/σ (I)>	14.34 (1.9)	7.34 (0.47)	5.59 (0.33)
Wilson B-value (Å^2^)	66.4	53.3	66.4
**Refinement**			
Molecules per asymmetric unit	6	6	6
Resolution range (Å)	48.03–2.44	48.03–2.08	51.3–2.70
R_work_/R_free_^b^ (%)	20.3/25.0	18.6/23.2	20.4/26.8
**Deviations from ideal geometry**			
Bond (Å)	0.02	0.02	0.007
Angles (°)	2.17	2.09	1.52
Ramachandran (%) Favoured/allowed/outliers *	96.3/3.7/0.0	95.7/4.2/0.1	94.2/5.5/0.3
**Validation**			
MolProbity score	1.83	2.05	2.47
Clash score	5.76	12.1	8.54
**Mean B-factors (Å^2^)**			
Protein	64.4	59.3	70.2
HEM/6DEB	47.4/52.6	41.8/46.0	48.1/59.5
H_2_O/Na^+^/FMT/GOL	58.8/-/81.1/75.1	64.2/54.6/84.5/67.4	53.7/-/86.3/-
**No. of Atoms**			
Protein	19439	19442	20068
HEM/6DEB	258/162/-/-	258/162	258/162
H_2_O/Na^+^/FMT/GOL	375/-/99/84	1339/8/417/78	224/-/18/-

^a^ R_merge_ is defined as ∑_h,k,l_∑_i_ |I_i_(h,k,l) − <I_i_(h,k,l)>|/∑_h,k,l_∑_i_I_i_(h,k,l), where I_i_(h,k,l) is the ith observation of reflection h,k,l and <I_i_ (h,k,l)> is the weighted mean of all observations (after rejection of outliers). ^b^ R_work_ is defined as ∑|*F_o_*|−|*F_c_*|/∑|*F_o_*| and indicates the accuracy of the model. R_free_ is based on 5% of the data randomly selected and is not used in the refinement. * Excluding prolines and glycines.

## Data Availability

Data presented in this study are openly available in the Protein Data Bank with deposition codes 7Q6R (E89Y-6DEB), 7Q89 (G92W-6DEB), 7Q6X (S240Y-6DEB).

## Supplementary Materials

The following are available online at https://www.mdpi.com/article/10.3390/biom12010055/s1. Figure S1: Reconstruction of the N-terminus of OleP in G92W-6DEB and S240Y-6DEB; Figure S2: Overall structure of OleP mutants bound to 6DEB; Figure S3: The typical structural features of the closed OleP-6DEB complex are preserved in mutants; Figure S4: Binding site of OleP wt (pdb 5MNS) and mutants bound to 6DEB; Figure S5: Active site organization of OleP mutants bound to 6DEB; Figure S6: The solvent cavity in OleP wild type and mutants; Figure S7: Essential dynamics of concatenated trajectory: Inspection of eigenvalues; Figure S8: The distributions of the minimum pair distance between the heme iron and substrate heavy atoms for OleP WT, E89Y, and G92W bound to 6DEB, as obtained by MD simulations; Figure S9: The distributions of the volume of the OleP active site for the WT, E89Y, and G92W, as obtained by MD simulations; Figure S10: Modeling G92W and S240Y in the open-6DEB bound OleP wild type structure.
