# Supplementary material for: Point Mutations at a Key Site Alter the Cytochrome P450 OleP Structural Dynamics
Source: Biomolecules. 2021 Dec 31;12(1):55. doi: 10.3390/biom12010055 (PMC8774231; doi:10.3390/biom12010055)
Supplement: Supplementary file 1 [file biomolecules-12-00055-s001.zip › Supplementary_Files_Revised/s240y-6deb_D_1292119137_val-report-full_P1.pdf]

Preliminary Full wwPDB X-ray Structure Validation Report (i)

Dec 17, 2021 – 11:28 am GMT

Deposition ID : D_1292119137

This is a Preliminary Full wwPDB X-ray Structure Validation Report.

This report is produced by the wwPDB Deposition System during initial deposition but before annotation of the structure.

We welcome your comments at *validation@mail.wwpdb.org*
A user guide is available at
https://www.wwpdb.org/validation/2017/XrayValidationReportHelp
with specific help available everywhere you see the (i) symbol.

PRELIMINARY VALIDATION REPORT

The following versions of software and data (see references (i)) were used in the production of this report:

MolProbity	:	4.02b-467
Mogul	:	1.8.4, CSD as541be (2020)
Xtriage (Phenix)	:	1.13
EDS	:	2.24
buster-report	:	1.1.7 (2018)
Percentile statistics	:	20191225.v01 (using entries in the PDB archive December 25th 2019)
Refmac	:	5.8.0267
CCP4	:	7.1.010 (Gargrove)
Ideal geometry (proteins)	:	Engh & Huber (2001)
Ideal geometry (DNA, RNA)	:	Parkinson et al. (1996)
Validation Pipeline (wwPDB-VP)	:	2.24

1 Overall quality at a glance (i)

The following experimental techniques were used to determine the structure:
X-RAY DIFFRACTION

The reported resolution of this entry is 2.70 Å.

Percentile scores (ranging between 0-100) for global validation metrics of the entry are shown in the following graphic. The table shows the number of entries on which the scores are based.

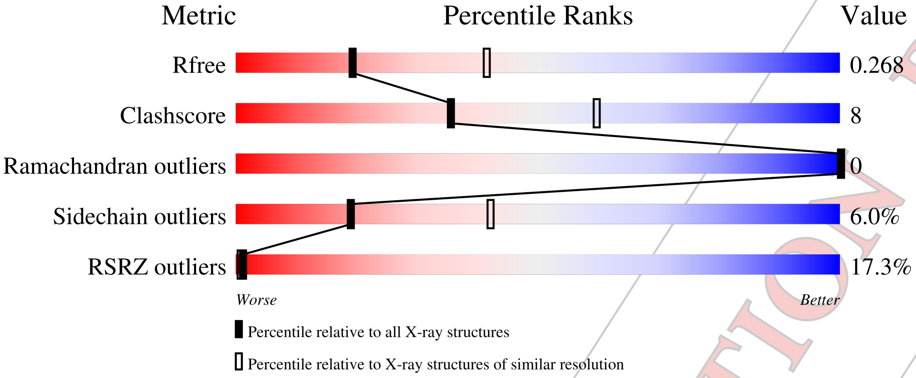

Metric	Whole archive (#Entries)	Similar resolution (#Entries, resolution range(Å))
R_{free}	130704	2808 (2.70-2.70)
Clashscore	141614	3122 (2.70-2.70)
Ramachandran outliers	138981	3069 (2.70-2.70)
Sidechain outliers	138945	3069 (2.70-2.70)
RSRZ outliers	127900	2737 (2.70-2.70)

The table below summarises the geometric issues observed across the polymeric chains and their fit to the electron density. The red, orange, yellow and green segments of the lower bar indicate the fraction of residues that contain outliers for >=3, 2, 1 and 0 types of geometric quality criteria respectively. A grey segment represents the fraction of residues that are not modelled. The numeric value for each fraction is indicated below the corresponding segment, with a dot representing fractions <=5% The upper red bar (where present) indicates the fraction of residues that have poor fit to the electron density. The numeric value is given above the bar.

Mol	Chain	Length	Quality of chain
1	A	397	10% / 78% 20% •
2	B	406	9% / 81% 18% •
3	C	399	5% / 84% 14% •
4	D	396	16% / 75% 24% •
5	E	395	31% / 78% 21% •

Continued on next page...

PRELIMINARY VALIDATION REPORT

Continued from previous page...

Mol	Chain	Length	Quality of chain
6	F	397	34% 80% 18% •

The following table lists non-polymeric compounds, carbohydrate monomers and non-standard residues in protein, DNA, RNA chains that are outliers for geometric or electron-density-fit criteria:

Mol	Type	Chain	Res	Chirality	Geometry	Clashes	Electron density
9	FMT	A	504	-	-	-	X
9	FMT	H	3	-	-	-	X

PRELIMINARY VALIDATION REPORT

2 Entry composition (i)

There are 10 unique types of molecules in this entry. The entry contains 20720 atoms, of which 0 are hydrogens and 0 are deuteriums.

In the tables below, the ZeroOcc column contains the number of atoms modelled with zero occupancy, the AltConf column contains the number of residues with at least one atom in alternate conformation and the Trace column contains the number of residues modelled with at most 2 atoms.

- Molecule 1 is a protein called Cytochrome P-450.

Mol	Chain	Residues	Atoms					ZeroOcc	AltConf	Trace
1	A	397	Total<br>3355	C<br>2126	N<br>598	O<br>616	S<br>15	0	41	0

There is a discrepancy between the modelled and reference sequences:

Chain	Residue	Modelled	Actual	Comment	Reference
A	240	TYR	SER	engineered mutation	UNP Q59819

- Molecule 2 is a protein called Cytochrome P-450.

Mol	Chain	Residues	Atoms					ZeroOcc	AltConf	Trace
2	B	406	Total<br>3361	C<br>2128	N<br>603	O<br>617	S<br>13	0	32	0

There is a discrepancy between the modelled and reference sequences:

Chain	Residue	Modelled	Actual	Comment	Reference
B	240	TYR	SER	engineered mutation	UNP Q59819

- Molecule 3 is a protein called Cytochrome P-450.

Mol	Chain	Residues	Atoms					ZeroOcc	AltConf	Trace
3	C	399	Total<br>3262	C<br>2060	N<br>585	O<br>604	S<br>13	0	24	0

There is a discrepancy between the modelled and reference sequences:

Chain	Residue	Modelled	Actual	Comment	Reference
C	240	TYR	SER	engineered mutation	UNP Q59819

- Molecule 4 is a protein called Cytochrome P-450.

PRELIMINARY VALIDATION REPORT

Mol	Chain	Residues	Atoms					ZeroOcc	AltConf	Trace
			Total	C	N	O	S			
4	D	396	3396	2161	606	616	13	0	48	0

There is a discrepancy between the modelled and reference sequences:

Chain	Residue	Modelled	Actual	Comment	Reference
D	240	TYR	SER	engineered mutation	UNP Q59819

- Molecule 5 is a protein called Cytochrome P-450.

Mol	Chain	Residues	Atoms					ZeroOcc	AltConf	Trace
			Total	C	N	O	S			
5	E	395	3282	2082	587	599	14	0	31	0

There is a discrepancy between the modelled and reference sequences:

Chain	Residue	Modelled	Actual	Comment	Reference
E	240	TYR	SER	engineered mutation	UNP Q59819

- Molecule 6 is a protein called Cytochrome P-450.

Mol	Chain	Residues	Atoms					ZeroOcc	AltConf	Trace
			Total	C	N	O	S			
6	F	397	3402	2161	601	626	14	0	50	0

There is a discrepancy between the modelled and reference sequences:

Chain	Residue	Modelled	Actual	Comment	Reference
F	240	TYR	SER	engineered mutation	UNP Q59819

- Molecule 7 is PROTOPORPHYRIN IX CONTAINING FE (three-letter code: HEM) (formula: $C_{34}H_{32}FeN_4O_4$).

PRELIMINARY VALIDATION REPORT

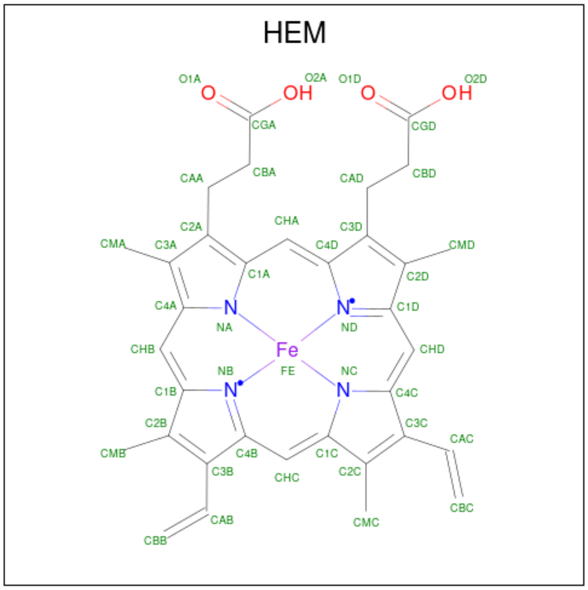

Mol	Chain	Residues	Atoms	ZeroOcc	AltConf
7	A	1	Total C Fe N O<br>43 34 1 4 4	0	0
7	B	1	Total C Fe N O<br>43 34 1 4 4	0	0
7	C	1	Total C Fe N O<br>43 34 1 4 4	0	0
7	D	1	Total C Fe N O<br>43 34 1 4 4	0	0
7	E	1	Total C Fe N O<br>43 34 1 4 4	0	0
7	F	1	Total C Fe N O<br>43 34 1 4 4	0	0

- Molecule 8 is 6-DEOXYERYTHRONOLIDE B (three-letter code: DEB) (formula: $C_{21}H_{38}O_6$).

PRELIMINARY VALIDATION REPORT

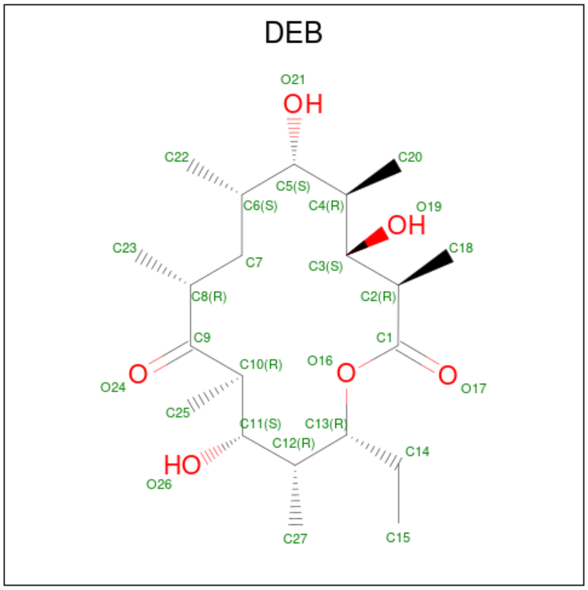

Mol	Chain	Residues	Atoms			ZeroOcc	AltConf
			Total	C	O		
8	A	1	27	21	6	0	0
8	B	1	27	21	6	0	0
8	C	1	27	21	6	0	0
8	D	1	27	21	6	0	0
8	E	1	27	21	6	0	0
8	F	1	27	21	6	0	0

- Molecule 9 is FORMIC ACID (three-letter code: FMT) (formula: CH_2O_2).

PRELIMINARY VALIDATION REPORT

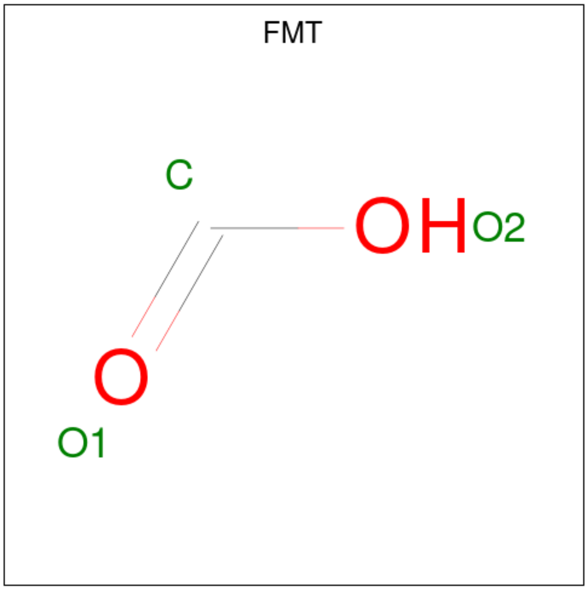

Mol	Chain	Residues	Atoms	ZeroOcc	AltConf
9	A	1	Total C O<br>3 1 2	0	0
9	A	1	Total C O<br>3 1 2	0	0
9	A	1	Total C O<br>3 1 2	0	0
9	H	1	Total C O<br>3 1 2	0	0
9	H	1	Total C O<br>3 1 2	0	0
9	H	1	Total C O<br>3 1 2	0	0

- Molecule 10 is water.

Mol	Chain	Residues	Atoms	ZeroOcc	AltConf
10	G	224	Total O<br>224 224	0	7

PRELIMINARY VALIDATION REPORT

3 Residue-property plots (i)

These plots are drawn for all protein, RNA, DNA and oligosaccharide chains in the entry. The first graphic for a chain summarises the proportions of the various outlier classes displayed in the second graphic. The second graphic shows the sequence view annotated by issues in geometry and electron density. Residues are color-coded according to the number of geometric quality criteria for which they contain at least one outlier: green = 0, yellow = 1, orange = 2 and red = 3 or more. A red dot above a residue indicates a poor fit to the electron density (RSRZ > 2). Stretches of 2 or more consecutive residues without any outlier are shown as a green connector. Residues present in the sample, but not in the model, are shown in grey.

• Molecule 1: Cytochrome P-450

Chain A:

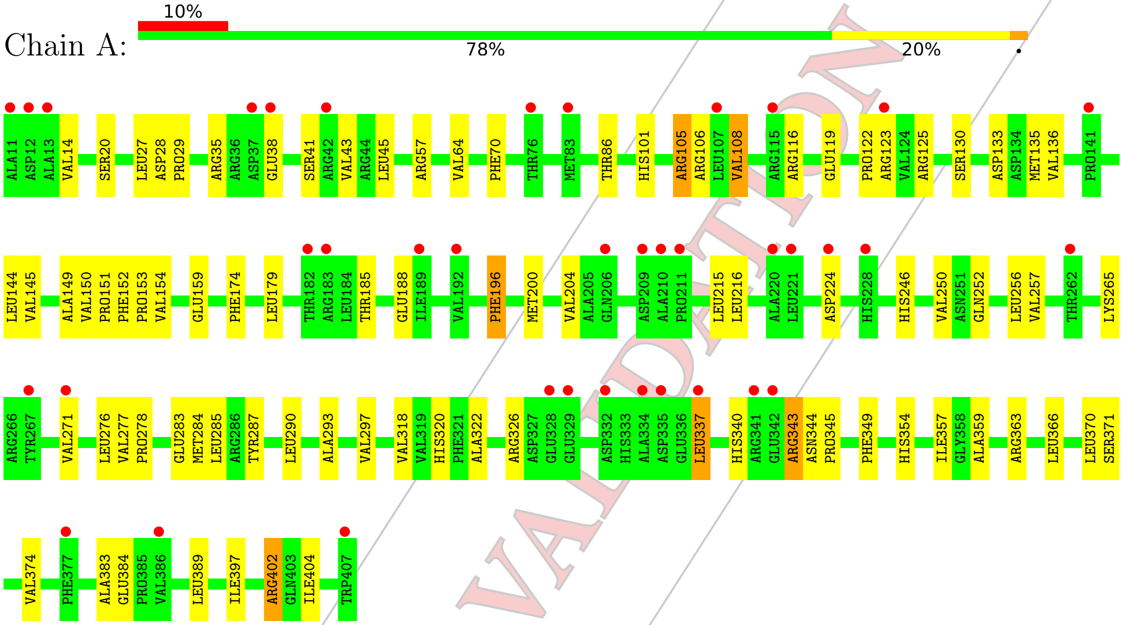

• Molecule 2: Cytochrome P-450

Chain B:

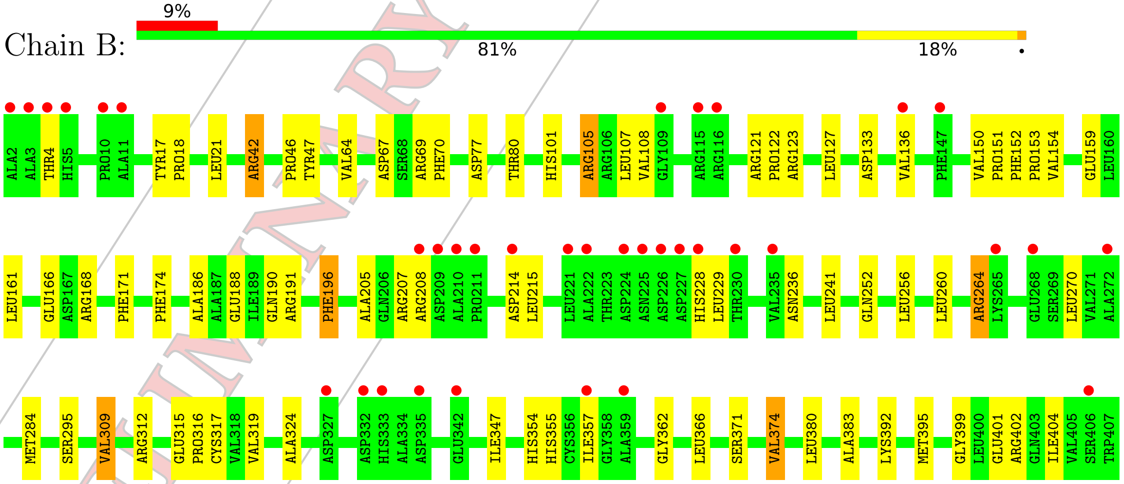

• Molecule 3: Cytochrome P-450

Chain C:

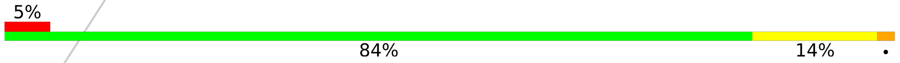

PRELIMINARY VALIDATION REPORT

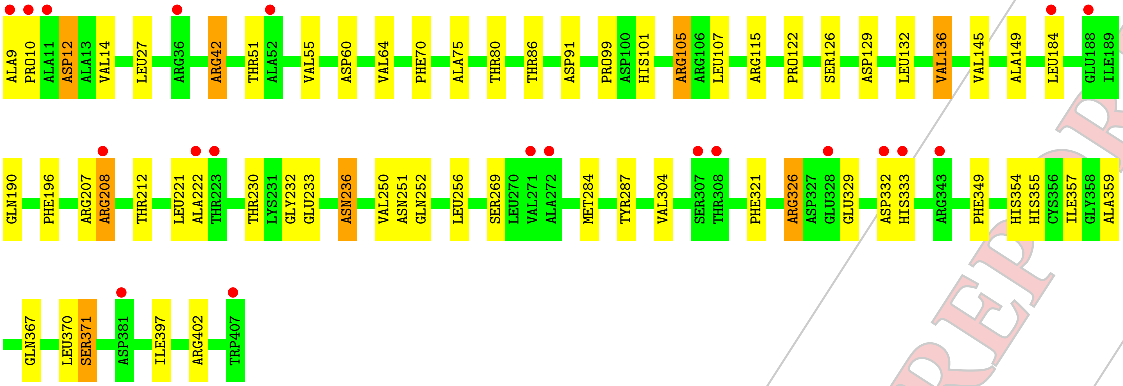

- Molecule 4: Cytochrome P-450

Chain D:

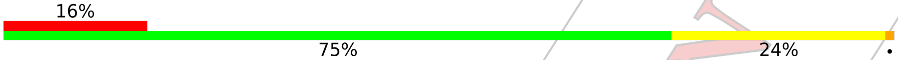

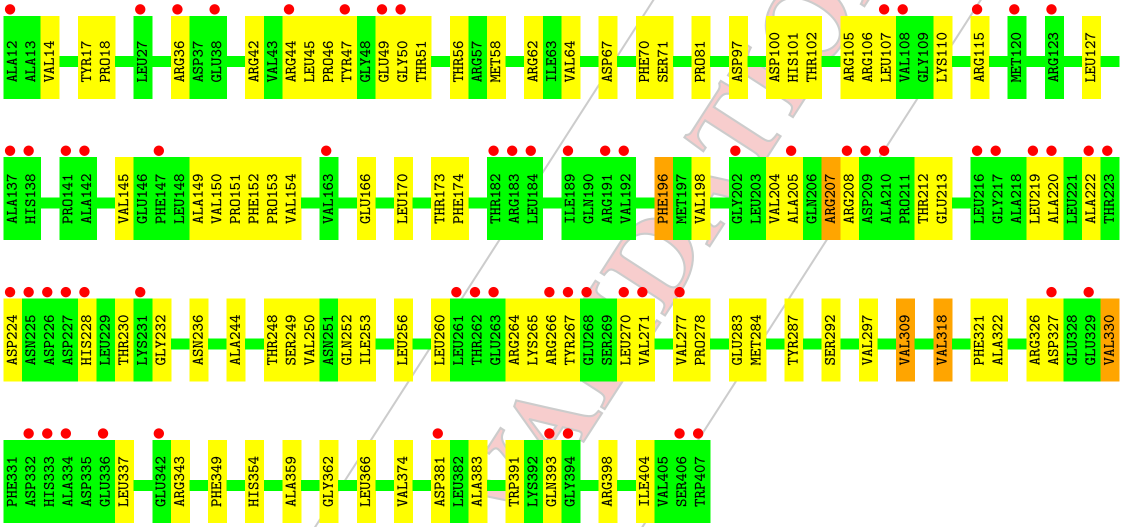

- Molecule 5: Cytochrome P-450

Chain E:

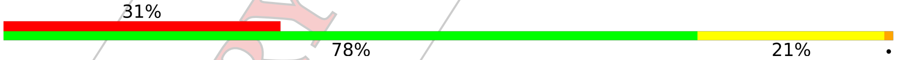

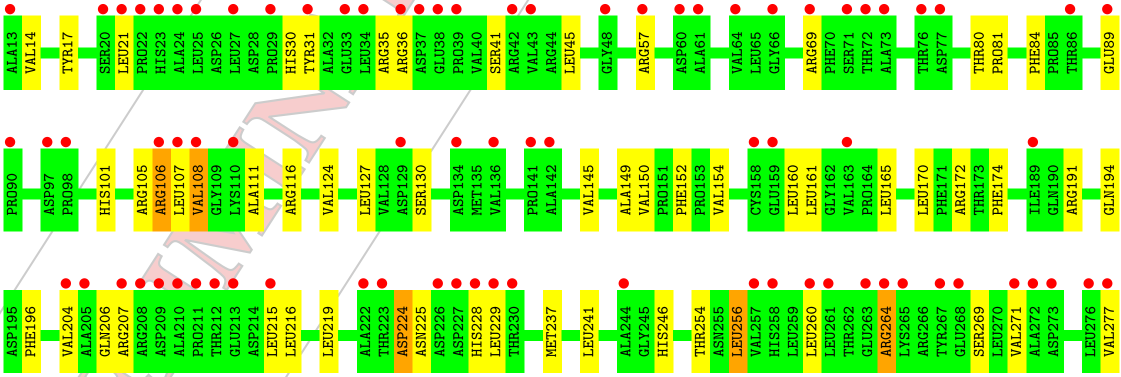

PRELIMINARY VALIDATION REPORT

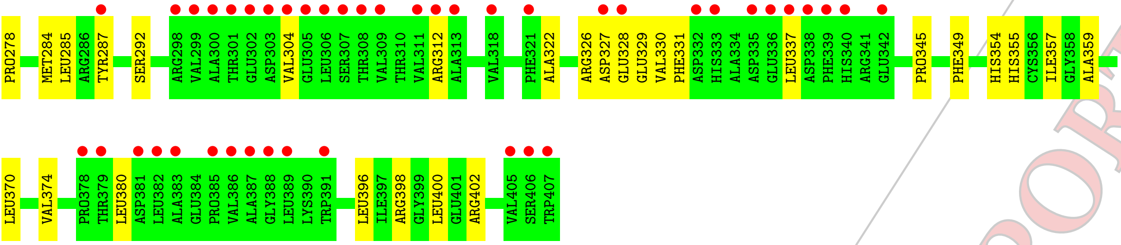

• Molecule 6: Cytochrome P-450

Chain F:

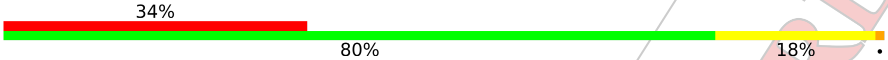

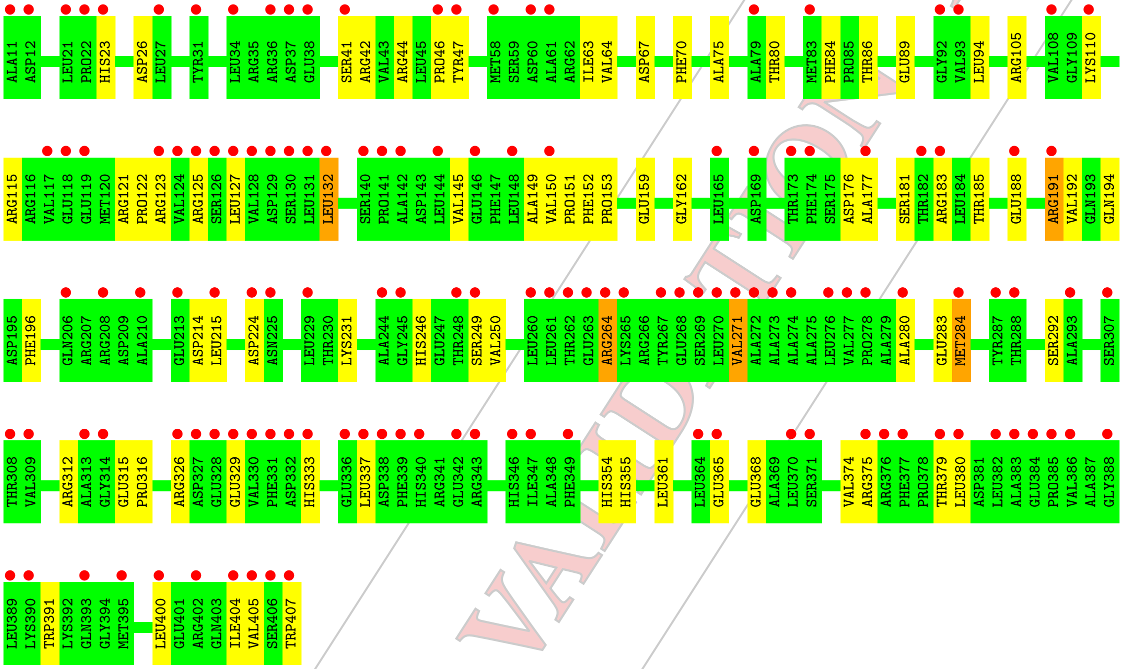

PRELIMINARY VALIDATION REPORT

4 Data and refinement statistics (i)

Property	Value	Source
Space group	C 1 2 1	Depositor
Cell constants<br>a, b, c, α, β, γ	248.62Å 110.36Å 160.05Å<br>90.00° 129.63° 90.00°	Depositor
Resolution (Å)	51.34 – 2.70<br>51.29 – 2.50	Depositor<br>EDS
% Data completeness<br>(in resolution range)	99.9 (51.34-2.70)<br>99.8 (51.29-2.50)	Depositor<br>EDS
R_{merge}	(Not available)	Depositor
R_{sym}	(Not available)	Depositor
$< I/\sigma(I) >$ [1]	1.34 (at 2.51Å)	Xtriage
Refinement program	REFMAC 5.8.0267	Depositor
R, R_{free}	0.204 , 0.268<br>0.206 , 0.268	Depositor<br>DCC
R_{free} test set	5559 reflections (4.84%)	wwPDB-VP
Wilson B-factor (Å^2)	75.4	Xtriage
Anisotropy	0.139	Xtriage
Bulk solvent k_{sol}(e/Å^3), B_{sol}(Å^2)	(Not available) , (Not available)	EDS
L-test for twinning[2]	$< \|L\| > = 0.49$, $< L^2 > = 0.32$	Xtriage
Estimated twinning fraction	0.008 for -h-2*l,-k,l	Xtriage
F_o,F_c correlation	0.96	EDS
Total number of atoms	20720	wwPDB-VP
Average B, all atoms (Å^2)	70.0	wwPDB-VP

Xtriage's analysis on translational NCS is as follows: *The analyses of the Patterson function reveals a significant off-origin peak that is 40.21 % of the origin peak, indicating pseudo-translational symmetry. The chance of finding a peak of this or larger height randomly in a structure without pseudo-translational symmetry is equal to 2.8308e-04. The detected translational NCS is most likely also responsible for the elevated intensity ratio.*

[1]Intensities estimated from amplitudes.

[2]Theoretical values of $< |L| >$, $< L^2 >$ for acentric reflections are 0.5, 0.333 respectively for untwinned datasets, and 0.375, 0.2 for perfectly twinned datasets.

PRELIMINARY VALIDATION REPORT

5 Model quality (i)

5.1 Standard geometry (i)

Bond lengths and bond angles in the following residue types are not validated in this section: FMT, DEB, HEM

The Z score for a bond length (or angle) is the number of standard deviations the observed value is removed from the expected value. A bond length (or angle) with $|Z| > 5$ is considered an outlier worth inspection. RMSZ is the root-mean-square of all Z scores of the bond lengths (or angles).

Mol	Chain	Bond lengths		Bond angles	
		RMSZ	#|Z| >5	RMSZ	#|Z| >5
1	A	0.68	0/3543	0.84	0/4812
2	B	0.68	0/3531	0.85	0/4801
3	C	0.66	0/3395	0.82	0/4620
4	D	0.70	0/3617	0.83	0/4912
5	E	0.69	0/3446	0.84	0/4679
6	F	0.73	0/3622	0.84	0/4917
All	All	0.69	0/21154	0.84	0/28741

There are no bond length outliers.

There are no bond angle outliers.

There are no chirality outliers.

There are no planarity outliers.

5.2 Too-close contacts (i)

In the following table, the Non-H and H(model) columns list the number of non-hydrogen atoms and hydrogen atoms in the chain respectively. The H(added) column lists the number of hydrogen atoms added and optimized by MolProbity. The Clashes column lists the number of clashes within the asymmetric unit, whereas Symm-Clashes lists symmetry-related clashes.

Mol	Chain	Non-H	H(model)	H(added)	Clashes	Symm-Clashes
1	A	3355	0	3408	57	0
2	B	3361	0	3417	49	0
3	C	3262	0	3276	47	0
4	D	3396	0	3495	74	0
5	E	3282	0	3347	58	0
6	F	3402	0	3494	37	0
7	A	43	0	30	4	0

Continued on next page...

PRELIMINARY VALIDATION REPORT

Continued from previous page...

Mol	Chain	Non-H	H(model)	H(added)	Clashes	Symm-Clashes
7	B	43	0	30	5	0
7	C	43	0	30	7	0
7	D	43	0	30	7	0
7	E	43	0	30	2	0
7	F	43	0	30	4	0
8	A	27	0	38	0	0
8	B	27	0	38	0	0
8	C	27	0	38	0	0
8	D	27	0	38	1	0
8	E	27	0	38	0	0
8	F	27	0	38	1	0
9	A	9	0	3	1	0
9	H	9	0	3	0	0
10	G	224	0	0	10	0
All	All	20720	0	20851	335	0

The all-atom clashscore is defined as the number of clashes found per 1000 atoms (including hydrogen atoms). The all-atom clashscore for this structure is 8.

All (335) close contacts within the same asymmetric unit are listed below, sorted by their clash magnitude.

Atom-1	Atom-2	Interatomic distance (Å)	Clash overlap (Å)
4:D:42[A]:ARG:NE	10:G:131[A]:HOH:O	1.80	1.15
5:E:106[A]:ARG:CG	5:E:106[A]:ARG:HH21	1.61	1.14
5:E:106[B]:ARG:HH11	5:E:106[B]:ARG:HG2	0.95	1.09
5:E:106[A]:ARG:HH21	5:E:106[A]:ARG:HG2	1.13	1.08
1:A:106[B]:ARG:HG2	1:A:106[B]:ARG:HH11	1.14	1.06
2:B:154[B]:VAL:HG11	2:B:168[B]:ARG:HD2	1.43	1.00
5:E:106[B]:ARG:HG2	5:E:106[B]:ARG:NH1	1.71	0.97
5:E:36[B]:ARG:NH1	5:E:328[B]:GLU:OE1	1.99	0.96
5:E:101[B]:HIS:CE1	5:E:354[B]:HIS:CD2	2.58	0.91
3:C:42[A]:ARG:HG2	10:G:120[A]:HOH:O	1.71	0.91
1:A:106[B]:ARG:HH11	1:A:106[B]:ARG:CG	1.86	0.87
2:B:264:ARG:NH1	2:B:380:LEU:O	2.09	0.85
5:E:106[B]:ARG:HH11	5:E:106[B]:ARG:CG	1.85	0.83
7:B:501:HEM:HMC1	7:B:501:HEM:HBC2	1.58	0.82
4:D:100[B]:ASP:OD1	4:D:228[B]:HIS:HE1	1.63	0.81
1:A:150:VAL:O	1:A:154:VAL:HG23	1.79	0.81
3:C:105[A]:ARG:HG3	3:C:105[A]:ARG:HH11	1.47	0.80
4:D:297:VAL:HG22	4:D:318:VAL:CG2	2.12	0.79
5:E:224:ASP:OD2	10:G:143:HOH:O	2.03	0.77

Continued on next page...

PRELIMINARY VALIDATION REPORT

Continued from previous page...

Atom-1	Atom-2	Interatomic distance (Å)	Clash overlap (Å)
5:E:107[A]:LEU:HD11	5:E:229:LEU:CD1	2.16	0.76
5:E:106[A]:ARG:HG2	5:E:106[A]:ARG:NH2	1.94	0.76
2:B:256:LEU:HD22	2:B:284:MET:HB3	1.67	0.75
1:A:106[B]:ARG:HG2	1:A:106[B]:ARG:NH1	1.95	0.74
5:E:106[A]:ARG:CG	5:E:106[A]:ARG:NH2	2.32	0.74
4:D:287:TYR:O	4:D:287:TYR:HD1	1.71	0.73
5:E:105:ARG:NH2	5:E:355:HIS:O	2.19	0.73
1:A:343[A]:ARG:NH2	10:G:187:HOH:O	2.21	0.72
4:D:42[A]:ARG:CD	10:G:131[A]:HOH:O	2.32	0.71
5:E:101[B]:HIS:HE1	5:E:354[B]:HIS:CD2	2.07	0.71
2:B:270:LEU:HB2	2:B:374[A]:VAL:HG21	1.72	0.71
5:E:216:LEU:HD23	5:E:219:LEU:HD12	1.73	0.71
6:F:145:VAL:HA	6:F:149:ALA:HB3	1.73	0.71
4:D:297:VAL:HG22	4:D:318:VAL:HG23	1.71	0.70
4:D:102:THR:OG1	10:G:52:HOH:O	2.08	0.70
5:E:107[A]:LEU:HD21	5:E:229:LEU:HD12	1.73	0.70
5:E:106[A]:ARG:HH21	5:E:106[A]:ARG:HG3	1.54	0.70
1:A:101[B]:HIS:CE1	1:A:354[B]:HIS:CD2	2.80	0.70
2:B:123[B]:ARG:HH22	2:B:159[B]:GLU:HG2	1.55	0.69
6:F:105:ARG:NH2	6:F:355:HIS:O	2.25	0.69
4:D:287:TYR:O	4:D:287:TYR:CD1	2.45	0.69
2:B:270:LEU:CB	2:B:374[A]:VAL:HG21	2.24	0.67
3:C:101[B]:HIS:CE1	3:C:354[B]:HIS:CD2	2.82	0.67
3:C:91:ASP:OD1	10:G:125:HOH:O	2.15	0.65
4:D:152:PHE:HB3	4:D:153:PRO:HD3	1.78	0.65
5:E:101[B]:HIS:HE1	5:E:354[B]:HIS:HD2	1.43	0.65
2:B:392:LYS:HG3	2:B:399:GLY:O	1.96	0.65
3:C:145:VAL:HA	3:C:149:ALA:HB3	1.78	0.65
4:D:383:ALA:HB3	4:D:404:ILE:HG22	1.79	0.64
7:F:501:HEM:HMB2	7:F:501:HEM:HBB2	1.79	0.64
5:E:106[A]:ARG:NH2	5:E:106[A]:ARG:HG3	2.09	0.64
2:B:188:GLU:O	2:B:191[B]:ARG:HG2	1.99	0.63
1:A:145:VAL:HA	1:A:149:ALA:HB3	1.80	0.63
6:F:42:ARG:NH2	10:G:148:HOH:O	2.31	0.63
3:C:9[B]:ALA:HA	3:C:12[B]:ASP:OD1	1.99	0.63
5:E:17:TYR:HE1	5:E:31:TYR:HH	1.45	0.63
5:E:35:ARG:HG2	5:E:57[A]:ARG:HG2	1.81	0.62
2:B:123[B]:ARG:NH2	2:B:159[B]:GLU:HG2	2.15	0.62
4:D:232:GLY:O	4:D:236:ASN:HB2	2.00	0.62
4:D:101[B]:HIS:CE1	4:D:354[B]:HIS:CD2	2.89	0.61
5:E:107[A]:LEU:HD11	5:E:229:LEU:HD13	1.83	0.61

Continued on next page...

PRELIMINARY VALIDATION REPORT

Continued from previous page...

Atom-1	Atom-2	Interatomic distance (Å)	Clash overlap (Å)
1:A:106[B]:ARG:CG	1:A:106[B]:ARG:NH1	2.51	0.61
4:D:49[C]:GLU:OE1	4:D:49[C]:GLU:HA	2.01	0.61
1:A:283:GLU:HG3	1:A:337:LEU:HD22	1.83	0.60
4:D:106[A]:ARG:NH2	4:D:110[A]:LYS:CE	2.65	0.60
7:D:501:HEM:HBB2	7:D:501:HEM:HMB2	1.83	0.60
6:F:177:ALA:HB3	6:F:192:VAL:HG11	1.84	0.60
3:C:105[A]:ARG:HH11	3:C:105[A]:ARG:CG	2.13	0.59
4:D:45:LEU:HB2	4:D:81:PRO:HB3	1.84	0.59
4:D:270:LEU:HB3	4:D:374:VAL:HG21	1.82	0.59
2:B:123[B]:ARG:HH12	2:B:159[B]:GLU:HG2	1.68	0.59
1:A:204:VAL:HG12	1:A:216:LEU:HD22	1.84	0.58
2:B:207:ARG:NH2	2:B:214:ASP:OD2	2.36	0.58
2:B:309:VAL:HG13	3:C:122:PRO:HA	1.84	0.58
4:D:100[B]:ASP:OD1	4:D:228[B]:HIS:CE1	2.52	0.58
4:D:106[A]:ARG:HH22	4:D:110[A]:LYS:HE2	1.69	0.58
5:E:69[A]:ARG:HE	5:E:304:VAL:HG22	1.69	0.58
3:C:105[A]:ARG:NH2	3:C:355:HIS:O	2.37	0.58
3:C:251:ASN:HD22	3:C:397:ILE:HD12	1.68	0.58
5:E:108:VAL:HG22	5:E:215[B]:LEU:HD11	1.85	0.58
3:C:9[B]:ALA:N	3:C:10[B]:PRO:CD	2.67	0.57
5:E:292:SER:HA	5:E:398:ARG:HE	1.70	0.56
6:F:280:ALA:O	6:F:284[B]:MET:HG3	2.06	0.56
2:B:152:PHE:HB3	2:B:153:PRO:HD3	1.87	0.56
2:B:161:LEU:O	2:B:214:ASP:HB3	2.06	0.56
3:C:14:VAL:HG21	3:C:51[B]:THR:HG23	1.86	0.56
6:F:361:LEU:O	6:F:365[B]:GLU:HG2	2.05	0.56
1:A:256:LEU:HD22	1:A:284[B]:MET:HB3	1.88	0.56
4:D:64:VAL:HA	4:D:70:PHE:CD2	2.40	0.56
4:D:271:VAL:HA	4:D:374:VAL:HG13	1.88	0.56
6:F:121:ARG:N	6:F:122:PRO:HD2	2.21	0.55
5:E:107[A]:LEU:HD21	5:E:229:LEU:CD1	2.35	0.55
1:A:185:THR:OG1	1:A:188:GLU:HG3	2.07	0.55
3:C:208[B]:ARG:HH21	3:C:208[B]:ARG:HB2	1.72	0.55
2:B:256:LEU:HD22	2:B:284:MET:CB	2.37	0.54
1:A:290:LEU:HD22	7:A:501:HEM:HMB3	1.89	0.54
3:C:256:LEU:HD22	3:C:284:MET:HB3	1.90	0.54
3:C:230:THR:OG1	3:C:233:GLU:HG3	2.08	0.54
7:B:501:HEM:HBC2	7:B:501:HEM:CMC	2.31	0.54
6:F:46:PRO:HB2	6:F:47:TYR:CD1	2.43	0.54
4:D:205:ALA:HA	4:D:208[B]:ARG:HG3	1.89	0.54
7:E:501:HEM:HBC2	7:E:501:HEM:HMC2	1.90	0.54

Continued on next page...

PRELIMINARY VALIDATION REPORT

Continued from previous page...

Atom-1	Atom-2	Interatomic distance (Å)	Clash overlap (Å)
5:E:256:LEU:HD22	5:E:284:MET:HB3	1.89	0.53
4:D:391:TRP:O	4:D:393[A]:GLN:HG3	2.09	0.53
3:C:232:GLY:O	3:C:236:ASN:HB2	2.09	0.53
7:E:501:HEM:HHC	7:E:501:HEM:HBB2	1.90	0.53
2:B:64:VAL:HA	2:B:70:PHE:CD2	2.43	0.53
2:B:123[B]:ARG:NH1	2:B:159[B]:GLU:HG2	2.24	0.53
1:A:179:LEU:HD13	1:A:397[A]:ILE:CD1	2.39	0.52
3:C:252:GLN:O	3:C:256:LEU:HG	2.09	0.52
6:F:64:VAL:HA	6:F:70:PHE:CD2	2.43	0.52
2:B:133:ASP:O	2:B:136[A]:VAL:HG22	2.09	0.52
4:D:71:SER:OG	4:D:97:ASP:OD2	2.17	0.52
7:D:501:HEM:HBB2	7:D:501:HEM:CMB	2.39	0.52
5:E:191[A]:ARG:NH2	5:E:194[A]:GLN:OE1	2.42	0.52
6:F:125[B]:ARG:HE	6:F:125[B]:ARG:HA	1.74	0.52
7:A:501:HEM:HBB2	7:A:501:HEM:HMB1	1.91	0.52
1:A:271:VAL:HA	1:A:374:VAL:HG13	1.92	0.52
4:D:322:ALA:O	4:D:326:ARG:HG2	2.10	0.52
3:C:132:LEU:O	3:C:136:VAL:HG13	2.10	0.51
4:D:106[A]:ARG:NH2	4:D:110[A]:LYS:NZ	2.58	0.51
1:A:174:PHE:HB3	1:A:196:PHE:CD2	2.45	0.51
6:F:23[A]:HIS:HD2	6:F:26[A]:ASP:OD2	1.93	0.51
2:B:252:GLN:OE1	2:B:252:GLN:HA	2.11	0.51
3:C:12[B]:ASP:OD1	3:C:12[B]:ASP:N	2.43	0.51
4:D:100[B]:ASP:HB2	10:G:205[B]:HOH:O	2.09	0.51
4:D:106[A]:ARG:HH22	4:D:110[A]:LYS:CE	2.23	0.51
2:B:105:ARG:NH2	2:B:355:HIS:O	2.44	0.51
2:B:256:LEU:CD2	2:B:284:MET:HB3	2.39	0.51
5:E:170:LEU:HD22	5:E:174:PHE:CZ	2.45	0.51
3:C:14:VAL:HG11	3:C:42[B]:ARG:HB3	1.92	0.51
1:A:105:ARG:HD2	1:A:357:ILE:HD12	1.93	0.51
1:A:343[A]:ARG:HD3	1:A:345:PRO:HD3	1.92	0.51
1:A:366:LEU:HD11	7:A:501:HEM:HBB1	1.93	0.51
5:E:264:ARG:NH2	5:E:380:LEU:O	2.44	0.51
1:A:277:VAL:HB	1:A:278:PRO:HD3	1.92	0.51
5:E:349:PHE:CE1	5:E:359:ALA:HA	2.46	0.51
1:A:122:PRO:HB3	4:D:309:VAL:HG12	1.92	0.50
4:D:150:VAL:O	4:D:154:VAL:HG23	2.12	0.50
2:B:108:VAL:HG11	2:B:241:LEU:HD11	1.93	0.50
1:A:252:GLN:O	1:A:256:LEU:HG	2.12	0.50
2:B:101[B]:HIS:HE1	7:B:501:HEM:O2D	1.95	0.50
4:D:170:LEU:C	4:D:170:LEU:HD23	2.32	0.50

Continued on next page...

PRELIMINARY VALIDATION REPORT

Continued from previous page...

Atom-1	Atom-2	Interatomic distance (Å)	Clash overlap (Å)
6:F:150:VAL:HB	6:F:151:PRO:HD3	1.94	0.50
6:F:379:THR:O	6:F:407:TRP:HA	2.11	0.50
4:D:45:LEU:HD12	4:D:81:PRO:HB2	1.95	0.49
2:B:105:ARG:HD2	2:B:357:ILE:HD12	1.94	0.49
7:D:501:HEM:HBA2	8:D:502:DEB:H253	1.94	0.49
3:C:99:PRO:HD2	10:G:123:HOH:O	2.12	0.49
5:E:105:ARG:HD3	5:E:357:ILE:HD12	1.93	0.49
1:A:384:GLU:OE1	1:A:389:LEU:HD23	2.12	0.49
2:B:383:ALA:HB3	2:B:404:ILE:HG22	1.93	0.49
6:F:94:LEU:HA	6:F:354[B]:HIS:HD2	1.77	0.49
1:A:144:LEU:HD21	1:A:257:VAL:HG21	1.95	0.49
3:C:55:VAL:HG13	3:C:60:ASP:HB2	1.95	0.49
3:C:9[B]:ALA:CA	3:C:12[B]:ASP:OD1	2.60	0.49
4:D:256:LEU:CD2	4:D:284:MET:HB3	2.43	0.49
4:D:256:LEU:HD22	4:D:284:MET:HB3	1.95	0.49
4:D:266:ARG:CZ	4:D:337:LEU:HD12	2.42	0.49
1:A:108:VAL:HG22	1:A:215[A]:LEU:HD22	1.94	0.48
1:A:246:HIS:O	1:A:250:VAL:HG23	2.13	0.48
3:C:287:TYR:O	3:C:326:ARG:NH1	2.45	0.48
5:E:45:LEU:HD22	5:E:81:PRO:HB2	1.95	0.48
1:A:133:ASP:O	1:A:136:VAL:HG22	2.13	0.48
1:A:150:VAL:O	1:A:154:VAL:CG2	2.58	0.48
6:F:75:ALA:HA	6:F:80:THR:HG21	1.95	0.48
1:A:285:LEU:HD13	1:A:349:PHE:CE2	2.48	0.48
1:A:322:ALA:O	1:A:326:ARG:HG2	2.14	0.48
5:E:145:VAL:HA	5:E:149:ALA:HB3	1.95	0.48
5:E:322:ALA:O	5:E:326:ARG:HG2	2.14	0.48
6:F:283:GLU:HG3	6:F:337[B]:LEU:HD23	1.95	0.48
6:F:185:THR:HG23	6:F:188[A]:GLU:OE1	2.14	0.48
2:B:205:ALA:HA	2:B:208[B]:ARG:HE	1.79	0.48
2:B:260:LEU:HG	2:B:284:MET:HE1	1.95	0.47
4:D:166[A]:GLU:HB2	5:E:228[A]:HIS:CE1	2.49	0.47
6:F:94:LEU:HD12	6:F:354[B]:HIS:HD2	1.80	0.47
2:B:67:ASP:CG	2:B:69:ARG:HE	2.17	0.47
4:D:149:ALA:O	4:D:250:VAL:HG22	2.14	0.47
4:D:260:LEU:HD22	4:D:267:TYR:HA	1.95	0.47
6:F:162:GLY:HA3	6:F:214:ASP:CG	2.35	0.47
1:A:64:VAL:HA	1:A:70:PHE:CD2	2.50	0.47
5:E:165:LEU:HD12	5:E:165:LEU:HA	1.80	0.47
4:D:101[A]:HIS:CD2	4:D:354[A]:HIS:CE1	3.02	0.47
3:C:105[A]:ARG:CG	3:C:105[A]:ARG:NH1	2.71	0.47

Continued on next page...

PRELIMINARY VALIDATION REPORT

Continued from previous page...

Atom-1	Atom-2	Interatomic distance (Å)	Clash overlap (Å)
7:B:501:HEM:HBB2	7:B:501:HEM:HMB2	1.96	0.47
6:F:123[B]:ARG:NH2	6:F:127[B]:LEU:HD11	2.30	0.47
2:B:174:PHE:HB3	2:B:196:PHE:CD2	2.49	0.47
5:E:106[B]:ARG:NH1	5:E:106[B]:ARG:CG	2.54	0.47
4:D:42[B]:ARG:NH1	4:D:51[B]:THR:OG1	2.48	0.46
4:D:327:ASP:HB3	4:D:330:VAL:CG1	2.45	0.46
7:D:501:HEM:HBC2	7:D:501:HEM:CMC	2.45	0.46
4:D:270:LEU:CB	4:D:374:VAL:HG21	2.46	0.46
4:D:283:GLU:HG3	4:D:337:LEU:CD2	2.45	0.46
7:F:501:HEM:HMC2	7:F:501:HEM:HBC2	1.97	0.46
3:C:349:PHE:CE1	3:C:359:ALA:HA	2.50	0.46
4:D:14:VAL:HG12	4:D:42[B]:ARG:HB3	1.98	0.46
1:A:130:SER:O	1:A:133:ASP:HB2	2.15	0.46
3:C:107:LEU:HD11	3:C:222:ALA:HB2	1.97	0.46
4:D:244:ALA:O	4:D:248:THR:OG1	2.25	0.46
4:D:277:VAL:HB	4:D:278:PRO:HD3	1.96	0.46
1:A:38[A]:GLU:CD	1:A:41:SER:HB3	2.36	0.46
7:C:501:HEM:HBC2	7:C:501:HEM:HMC2	1.98	0.46
7:D:501:HEM:HBC2	7:D:501:HEM:HMC2	1.98	0.46
2:B:186:ALA:O	2:B:190:GLN:HB2	2.17	0.45
1:A:116:ARG:O	1:A:119:GLU:HB2	2.17	0.45
1:A:152:PHE:HB3	1:A:153:PRO:HD3	1.97	0.45
4:D:150:VAL:N	4:D:151:PRO:HD2	2.32	0.45
1:A:276:LEU:HD11	1:A:340:HIS:NE2	2.31	0.45
1:A:297:VAL:HG22	1:A:318:VAL:HB	1.98	0.45
4:D:349:PHE:CE1	4:D:359:ALA:HA	2.52	0.45
6:F:271:VAL:HA	6:F:374:VAL:HG13	1.99	0.45
6:F:23[A]:HIS:CD2	6:F:26[A]:ASP:OD2	2.70	0.45
1:A:14:VAL:HG12	1:A:43:VAL:HA	1.99	0.44
1:A:256:LEU:HD22	1:A:284[A]:MET:HB3	1.99	0.44
3:C:367:GLN:O	3:C:371:SER:HB2	2.17	0.44
5:E:150:VAL:O	5:E:154:VAL:HG13	2.17	0.44
3:C:321:PHE:CZ	7:C:501:HEM:HBA2	2.53	0.44
6:F:152:PHE:HB3	6:F:153:PRO:HD3	1.98	0.44
1:A:179:LEU:HD13	1:A:397[A]:ILE:HD11	2.00	0.44
2:B:150:VAL:O	2:B:154[A]:VAL:HG22	2.17	0.44
2:B:309:VAL:HG13	3:C:122:PRO:CA	2.48	0.44
5:E:154:VAL:HG12	5:E:246:HIS:HB2	1.98	0.44
1:A:343[A]:ARG:NH2	9:A:504:FMT:O2	2.51	0.44
5:E:69[B]:ARG:HD2	5:E:304:VAL:HG22	1.99	0.44
6:F:264:ARG:NH2	6:F:380:LEU:O	2.51	0.44

Continued on next page...

PRELIMINARY VALIDATION REPORT

Continued from previous page...

Atom-1	Atom-2	Interatomic distance (Å)	Clash overlap (Å)
1:A:265[B]:LYS:HA	1:A:265[B]:LYS:HD3	1.85	0.44
3:C:101[B]:HIS:NE2	3:C:105[B]:ARG:HD3	2.33	0.44
1:A:359:ALA:O	1:A:363:ARG:HG3	2.18	0.44
2:B:150:VAL:N	2:B:151:PRO:HD2	2.33	0.44
3:C:75:ALA:HA	3:C:80:THR:HG21	1.99	0.44
4:D:106[A]:ARG:NH2	4:D:110[A]:LYS:HE2	2.29	0.44
1:A:20:SER:OG	1:A:28:ASP:OD2	2.21	0.44
4:D:174:PHE:HB3	4:D:196:PHE:CD2	2.53	0.44
6:F:191[B]:ARG:NH2	6:F:194[B]:GLN:OE1	2.51	0.44
2:B:315:GLU:HA	2:B:316:PRO:HD3	1.86	0.44
5:E:215[A]:LEU:HD23	5:E:215[A]:LEU:HA	1.90	0.44
1:A:293:ALA:HA	1:A:320:HIS:CE1	2.53	0.43
2:B:77:ASP:HB3	2:B:80:THR:OG1	2.18	0.43
1:A:35:ARG:HG2	1:A:57[B]:ARG:HG3	1.99	0.43
4:D:46:PRO:HB2	4:D:47:TYR:CD1	2.53	0.43
4:D:101[A]:HIS:CE1	4:D:105:ARG:HD3	2.53	0.43
4:D:256:LEU:HD22	4:D:284:MET:CB	2.48	0.43
5:E:111:ALA:CB	5:E:215[A]:LEU:HD21	2.48	0.43
6:F:63:ILE:O	6:F:67:ASP:HB2	2.17	0.43
3:C:101[B]:HIS:HE1	7:C:501:HEM:O2D	2.00	0.43
4:D:58:MET:HE2	4:D:62:ARG:HG3	2.00	0.43
5:E:260:LEU:HD11	5:E:370[B]:LEU:HD11	2.00	0.43
2:B:270:LEU:HB3	2:B:374[A]:VAL:HG21	1.99	0.43
1:A:179:LEU:HD13	1:A:397[A]:ILE:HD13	1.99	0.43
4:D:252:GLN:OE1	4:D:252:GLN:HA	2.19	0.43
4:D:166[A]:GLU:HB2	5:E:228[A]:HIS:HE1	1.84	0.43
5:E:57[B]:ARG:HG3	5:E:327:ASP:OD2	2.18	0.43
5:E:111:ALA:HB2	5:E:215[A]:LEU:HD21	2.01	0.43
2:B:46:PRO:HB2	2:B:47:TYR:CD1	2.53	0.43
1:A:343[A]:ARG:HH11	1:A:344:ASN:H	1.67	0.43
2:B:362:GLY:O	2:B:366:LEU:HG	2.18	0.43
3:C:55:VAL:HG21	3:C:64:VAL:HG21	2.01	0.43
3:C:332:ASP:C	3:C:333:HIS:ND1	2.72	0.43
3:C:357:ILE:HG22	7:C:501:HEM:C2D	2.54	0.43
7:C:501:HEM:HBC2	7:C:501:HEM:CMC	2.49	0.43
4:D:219:LEU:O	4:D:222:ALA:HB3	2.19	0.43
5:E:124:VAL:HG13	5:E:152:PHE:HE1	1.83	0.43
1:A:200:MET:O	1:A:204:VAL:HG13	2.19	0.42
2:B:324:ALA:CB	2:B:347:ILE:HD11	2.49	0.42
4:D:287:TYR:CD2	4:D:337:LEU:HG	2.54	0.42
5:E:287:TYR:CG	5:E:337:LEU:HD13	2.54	0.42

Continued on next page...

PRELIMINARY VALIDATION REPORT

Continued from previous page...

Atom-1	Atom-2	Interatomic distance (Å)	Clash overlap (Å)
4:D:207:ARG:HB2	4:D:220:ALA:CB	2.49	0.42
2:B:123[B]:ARG:HH12	2:B:159[B]:GLU:CG	2.32	0.42
3:C:14:VAL:HG11	3:C:42[A]:ARG:HB3	2.02	0.42
3:C:208[B]:ARG:CB	3:C:208[B]:ARG:NH2	2.82	0.42
4:D:152:PHE:CB	4:D:153:PRO:HD3	2.45	0.42
6:F:176[B]:ASP:OD1	6:F:183[B]:ARG:CZ	2.67	0.42
6:F:125[B]:ARG:NH1	6:F:368:GLU:O	2.52	0.42
6:F:391:TRP:CE2	6:F:400:LEU:HD21	2.55	0.42
4:D:166[B]:GLU:OE1	4:D:166[B]:GLU:HA	2.19	0.42
5:E:69[B]:ARG:CD	5:E:304:VAL:HG22	2.49	0.42
2:B:123[B]:ARG:CZ	2:B:159[B]:GLU:HG2	2.50	0.42
2:B:152:PHE:CB	2:B:153:PRO:HD3	2.50	0.42
2:B:354[B]:HIS:HD2	7:B:501:HEM:O1D	2.02	0.42
2:B:295:SER:HB3	2:B:319:VAL:O	2.20	0.42
3:C:70:PHE:CE2	3:C:304:VAL:HG11	2.55	0.42
3:C:256:LEU:HD22	3:C:284:MET:CB	2.49	0.42
4:D:292:SER:CB	4:D:398:ARG:HE	2.32	0.42
4:D:249:SER:O	4:D:253:ILE:HG13	2.20	0.41
5:E:35:ARG:O	5:E:57[A]:ARG:HD2	2.19	0.41
3:C:64:VAL:HA	3:C:70:PHE:CD2	2.55	0.41
3:C:207:ARG:HD3	3:C:212:THR:OG1	2.20	0.41
7:C:501:HEM:HBB2	7:C:501:HEM:HMB2	2.03	0.41
4:D:107[A]:LEU:HD23	4:D:219:LEU:CD2	2.50	0.41
5:E:285:LEU:HD13	5:E:349:PHE:CE2	2.55	0.41
6:F:132:LEU:HD12	6:F:132:LEU:HA	1.89	0.41
4:D:17:TYR:CD1	4:D:18:PRO:HA	2.56	0.41
4:D:44[A]:ARG:HG3	4:D:50:GLY:HA2	2.03	0.41
3:C:221:LEU:HD23	3:C:221:LEU:HA	1.93	0.41
3:C:357:ILE:CG2	7:C:501:HEM:HMD2	2.51	0.41
4:D:283:GLU:HG3	4:D:337:LEU:HD22	2.03	0.41
6:F:315:GLU:HA	6:F:316:PRO:HD3	1.84	0.41
3:C:64:VAL:HA	3:C:70:PHE:CE2	2.55	0.41
4:D:36[A]:ARG:HE	4:D:36[A]:ARG:HB2	1.70	0.41
5:E:237:MET:O	5:E:241:LEU:HG	2.21	0.41
6:F:84:PHE:CD2	8:F:502:DEB:H182	2.56	0.41
7:F:501:HEM:HBB2	7:F:501:HEM:CMB	2.49	0.41
7:F:501:HEM:HBC2	7:F:501:HEM:CMC	2.51	0.41
4:D:321:PHE:HZ	7:D:501:HEM:O2A	2.04	0.41
1:A:123[B]:ARG:NH1	1:A:159[B]:GLU:OE2	2.46	0.41
1:A:150:VAL:N	1:A:151:PRO:HD2	2.36	0.41
1:A:287:TYR:O	1:A:326:ARG:NH1	2.54	0.41

Continued on next page...

PRELIMINARY VALIDATION REPORT

Continued from previous page...

Atom-1	Atom-2	Interatomic distance (Å)	Clash overlap (Å)
2:B:121:ARG:HB3	2:B:122:PRO:HD3	2.03	0.41
2:B:312:ARG:NH2	3:C:129:ASP:OD2	2.46	0.41
4:D:101[B]:HIS:HE1	7:D:501:HEM:O2D	2.04	0.41
4:D:207:ARG:HD2	4:D:212:THR:OG1	2.21	0.41
5:E:84:PHE:HB3	5:E:396:LEU:HD11	2.02	0.41
2:B:17:TYR:HA	2:B:18:PRO:C	2.41	0.41
5:E:254:THR:HB	5:E:400:LEU:HB2	2.02	0.40
5:E:277:VAL:HB	5:E:278:PRO:HD3	2.03	0.40
6:F:284[B]:MET:HG2	6:F:337[B]:LEU:HD21	2.02	0.40
1:A:150:VAL:N	1:A:151:PRO:CD	2.85	0.40
2:B:107:LEU:HD11	2:B:229:LEU:HD13	2.03	0.40
5:E:160:LEU:HG	5:E:215[A]:LEU:HD12	2.03	0.40
6:F:123[B]:ARG:CZ	6:F:127[B]:LEU:HD11	2.51	0.40
1:A:28:ASP:HA	1:A:29:PRO:HD3	1.96	0.40
4:D:362:GLY:O	4:D:366:LEU:HG	2.21	0.40
1:A:383:ALA:HB3	1:A:404:ILE:HG22	2.04	0.40
7:A:501:HEM:HMD1	7:A:501:HEM:HBD2	2.03	0.40
4:D:145:VAL:HA	4:D:149:ALA:HB3	2.02	0.40
5:E:271:VAL:HA	5:E:374:VAL:HG13	2.03	0.40
5:E:331:PHE:CE1	5:E:345:PRO:HD2	2.56	0.40

There are no symmetry-related clashes.

5.3 Torsion angles (i)

5.3.1 Protein backbone (i)

In the following table, the Percentiles column shows the percent Ramachandran outliers of the chain as a percentile score with respect to all X-ray entries followed by that with respect to entries of similar resolution.

The Analysed column shows the number of residues for which the backbone conformation was analysed, and the total number of residues.

Mol	Chain	Analysed	Favoured	Allowed	Outliers	Percentiles	
1	A	436/397 (110%)	410 (94%)	26 (6%)	0	100	100
2	B	437/406 (108%)	417 (95%)	20 (5%)	0	100	100
3	C	420/399 (105%)	403 (96%)	17 (4%)	0	100	100
4	D	443/396 (112%)	410 (93%)	33 (7%)	0	100	100

Continued on next page...

PRELIMINARY VALIDATION REPORT

Continued from previous page...

Mol	Chain	Analysed	Favoured	Allowed	Outliers	Percentiles	
5	E	424/395 (107%)	398 (94%)	26 (6%)	0	100	100
6	F	445/397 (112%)	423 (95%)	22 (5%)	0	100	100
All	All	2605/2390 (109%)	2461 (94%)	144 (6%)	0	100	100

There are no Ramachandran outliers to report.

5.3.2 Protein sidechains ⓘ

In the following table, the Percentiles column shows the percent sidechain outliers of the chain as a percentile score with respect to all X-ray entries followed by that with respect to entries of similar resolution.

The Analysed column shows the number of residues for which the sidechain conformation was analysed, and the total number of residues.

Mol	Chain	Analysed	Rotameric	Outliers	Percentiles	
1	A	373/332 (112%)	356 (95%)	17 (5%)	27	54
2	B	371/338 (110%)	349 (94%)	22 (6%)	19	43
3	C	355/333 (107%)	332 (94%)	23 (6%)	17	38
4	D	380/331 (115%)	358 (94%)	22 (6%)	20	43
5	E	362/331 (109%)	334 (92%)	28 (8%)	13	30
6	F	380/330 (115%)	348 (92%)	32 (8%)	11	25
All	All	2221/1995 (111%)	2077 (94%)	144 (6%)	19	38

All (144) residues with a non-rotameric sidechain are listed below:

Mol	Chain	Res	Type
1	A	27	LEU
1	A	45	LEU
1	A	86	THR
1	A	105	ARG
1	A	108	VAL
1	A	125	ARG
1	A	135	MET
1	A	196	PHE
1	A	224[A]	ASP
1	A	224[B]	ASP
1	A	337	LEU
1	A	343[A]	ARG

Continued on next page...

PRELIMINARY VALIDATION REPORT

Continued from previous page...

Mol	Chain	Res	Type
1	A	343[B]	ARG
1	A	370	LEU
1	A	371	SER
1	A	402[A]	ARG
1	A	402[B]	ARG
2	B	4	THR
2	B	21	LEU
2	B	42[A]	ARG
2	B	42[B]	ARG
2	B	105	ARG
2	B	127	LEU
2	B	166	GLU
2	B	171	PHE
2	B	196	PHE
2	B	215	LEU
2	B	228[A]	HIS
2	B	228[B]	HIS
2	B	236	ASN
2	B	264	ARG
2	B	309	VAL
2	B	317	CYS
2	B	371	SER
2	B	374[A]	VAL
2	B	374[B]	VAL
2	B	395	MET
2	B	401	GLU
2	B	402	ARG
3	C	12[A]	ASP
3	C	12[B]	ASP
3	C	27	LEU
3	C	42[A]	ARG
3	C	42[B]	ARG
3	C	86	THR
3	C	105[A]	ARG
3	C	105[B]	ARG
3	C	115	ARG
3	C	126	SER
3	C	136	VAL
3	C	184	LEU
3	C	190	GLN
3	C	196	PHE
3	C	208[A]	ARG

Continued on next page...

PRELIMINARY VALIDATION REPORT

Continued from previous page...

Mol	Chain	Res	Type
3	C	208[B]	ARG
3	C	236	ASN
3	C	269	SER
3	C	326	ARG
3	C	329	GLU
3	C	370	LEU
3	C	371	SER
3	C	402	ARG
4	D	56	THR
4	D	67	ASP
4	D	115[A]	ARG
4	D	115[B]	ARG
4	D	127	LEU
4	D	173	THR
4	D	196	PHE
4	D	204	VAL
4	D	207	ARG
4	D	213[A]	GLU
4	D	213[B]	GLU
4	D	224[A]	ASP
4	D	224[B]	ASP
4	D	230	THR
4	D	264	ARG
4	D	265[A]	LYS
4	D	265[B]	LYS
4	D	309	VAL
4	D	318	VAL
4	D	330	VAL
4	D	343[A]	ARG
4	D	343[B]	ARG
5	E	14	VAL
5	E	21	LEU
5	E	30	HIS
5	E	41	SER
5	E	80	THR
5	E	89	GLU
5	E	106[A]	ARG
5	E	106[B]	ARG
5	E	108	VAL
5	E	116	ARG
5	E	127	LEU
5	E	130	SER

Continued on next page...

PRELIMINARY VALIDATION REPORT

Continued from previous page...

Mol	Chain	Res	Type
5	E	161	LEU
5	E	172	ARG
5	E	196	PHE
5	E	204	VAL
5	E	206	GLN
5	E	207	ARG
5	E	224	ASP
5	E	225	ASN
5	E	256	LEU
5	E	264	ARG
5	E	269	SER
5	E	312[A]	ARG
5	E	312[B]	ARG
5	E	329	GLU
5	E	330	VAL
5	E	402	ARG
6	F	41	SER
6	F	86	THR
6	F	89[A]	GLU
6	F	89[B]	GLU
6	F	110[A]	LYS
6	F	110[B]	LYS
6	F	115	ARG
6	F	132	LEU
6	F	159	GLU
6	F	181	SER
6	F	191[A]	ARG
6	F	191[B]	ARG
6	F	196	PHE
6	F	215	LEU
6	F	224[A]	ASP
6	F	224[B]	ASP
6	F	231	LYS
6	F	246[A]	HIS
6	F	246[B]	HIS
6	F	249	SER
6	F	264	ARG
6	F	271	VAL
6	F	284[A]	MET
6	F	284[B]	MET
6	F	292	SER
6	F	312	ARG

Continued on next page...

PRELIMINARY VALIDATION REPORT

Continued from previous page...

Mol	Chain	Res	Type
6	F	326	ARG
6	F	333	HIS
6	F	375[A]	ARG
6	F	375[B]	ARG
6	F	404	ILE
6	F	405	VAL

Sometimes sidechains can be flipped to improve hydrogen bonding and reduce clashes. All (5) such sidechains are listed below:

Mol	Chain	Res	Type
3	C	193	GLN
5	E	96	GLN
5	E	206	GLN
5	E	320	HIS
6	F	193	GLN

5.3.3 RNA (i)

There are no RNA molecules in this entry.

5.4 Non-standard residues in protein, DNA, RNA chains (i)

There are no non-standard protein/DNA/RNA residues in this entry.

5.5 Carbohydrates (i)

There are no monosaccharides in this entry.

5.6 Ligand geometry (i)

18 ligands are modelled in this entry.

In the following table, the Counts columns list the number of bonds (or angles) for which Mogul statistics could be retrieved, the number of bonds (or angles) that are observed in the model and the number of bonds (or angles) that are defined in the Chemical Component Dictionary. The Link column lists molecule types, if any, to which the group is linked. The Z score for a bond length (or angle) is the number of standard deviations the observed value is removed from the expected value. A bond length (or angle) with $|Z| > 2$ is considered an outlier worth inspection. RMSZ is the root-mean-square of all Z scores of the bond lengths (or angles).

PRELIMINARY VALIDATION REPORT

Mol	Type	Chain	Res	Link	Bond lengths			Bond angles		
					Counts	RMSZ	#|Z| > 2	Counts	RMSZ	#|Z| > 2
7	HEM	C	501	3	27,50,50	1.04	2 (7%)	17,82,82	1.81	4 (23%)
7	HEM	E	501	5	27,50,50	1.04	2 (7%)	17,82,82	1.74	5 (29%)
8	DEB	B	502	-	27,27,27	0.47	0	35,39,39	0.89	1 (2%)
8	DEB	A	502	-	27,27,27	0.48	0	35,39,39	0.97	2 (5%)
8	DEB	D	502	-	27,27,27	0.56	1 (3%)	35,39,39	0.90	1 (2%)
8	DEB	F	502	-	27,27,27	0.29	0	35,39,39	0.67	0
9	FMT	A	504	-	0,2,2	-	-	0,1,1	-	-
7	HEM	A	501	1	27,50,50	1.31	6 (22%)	17,82,82	2.39	8 (47%)
7	HEM	B	501	2	27,50,50	1.13	3 (11%)	17,82,82	1.87	7 (41%)
8	DEB	C	502	-	27,27,27	0.39	0	35,39,39	0.90	1 (2%)
9	FMT	H	1	-	0,2,2	-	-	0,1,1	-	-
9	FMT	H	3	-	0,2,2	-	-	0,1,1	-	-
9	FMT	A	505	-	0,2,2	-	-	0,1,1	-	-
9	FMT	H	2	-	0,2,2	-	-	0,1,1	-	-
9	FMT	A	503	-	0,2,2	-	-	0,1,1	-	-
8	DEB	E	502	-	27,27,27	0.37	0	35,39,39	0.73	1 (2%)
7	HEM	F	501	6	27,50,50	0.72	1 (3%)	17,82,82	1.11	1 (5%)
7	HEM	D	501	4	27,50,50	1.19	2 (7%)	17,82,82	1.63	6 (35%)

In the following table, the Chirals column lists the number of chiral outliers, the number of chiral centers analysed, the number of these observed in the model and the number defined in the Chemical Component Dictionary. Similar counts are reported in the Torsion and Rings columns. '-' means no outliers of that kind were identified.

Mol	Type	Chain	Res	Link	Chirals	Torsions	Rings
7	HEM	C	501	3	-	2/6/54/54	-
7	HEM	E	501	5	-	0/6/54/54	-
8	DEB	B	502	-	-	8/50/50/50	0/1/1/1
8	DEB	A	502	-	-	10/50/50/50	0/1/1/1
8	DEB	D	502	-	-	10/50/50/50	0/1/1/1
8	DEB	F	502	-	-	10/50/50/50	0/1/1/1
7	HEM	A	501	1	-	0/6/54/54	-
7	HEM	B	501	2	-	2/6/54/54	-
8	DEB	C	502	-	-	8/50/50/50	0/1/1/1
8	DEB	E	502	-	-	12/50/50/50	0/1/1/1
7	HEM	F	501	6	-	0/6/54/54	-
7	HEM	D	501	4	-	0/6/54/54	-

PRELIMINARY VALIDATION REPORT

All (17) bond length outliers are listed below:

Mol	Chain	Res	Type	Atoms	Z	Observed(Å)	Ideal(Å)
7	A	501	HEM	C3D-C4D	3.46	1.50	1.42
7	D	501	HEM	C3B-C2B	-3.44	1.35	1.40
7	E	501	HEM	C3B-C2B	-2.83	1.36	1.40
7	C	501	HEM	C3D-C4D	2.60	1.48	1.42
7	B	501	HEM	C4B-NB	-2.55	1.30	1.36
7	B	501	HEM	C3B-C2B	-2.54	1.36	1.40
7	A	501	HEM	C3D-C2D	-2.51	1.30	1.37
7	D	501	HEM	C4B-NB	-2.43	1.31	1.36
7	E	501	HEM	C3D-C4D	2.41	1.48	1.42
7	C	501	HEM	C3B-C2B	-2.36	1.37	1.40
7	A	501	HEM	CAA-C2A	2.28	1.55	1.52
7	A	501	HEM	C4B-NB	-2.18	1.31	1.36
7	A	501	HEM	C1A-CHA	-2.17	1.35	1.41
8	D	502	DEB	C2-C1	2.16	1.56	1.51
7	A	501	HEM	C1D-CHD	-2.12	1.35	1.41
7	B	501	HEM	C3D-C4D	2.05	1.47	1.42
7	F	501	HEM	C3D-C4D	2.05	1.47	1.42

All (37) bond angle outliers are listed below:

Mol	Chain	Res	Type	Atoms	Z	Observed(^{o})	Ideal(^{o})
7	A	501	HEM	C4A-C3A-C2A	4.75	110.30	107.00
7	C	501	HEM	CBA-CAA-C2A	3.95	119.77	112.49
7	A	501	HEM	CBA-CAA-C2A	3.90	119.69	112.49
7	A	501	HEM	C3B-C4B-NB	-3.57	104.59	109.21
7	E	501	HEM	C1D-C2D-C3D	-3.51	104.55	107.00
7	A	501	HEM	CAA-CBA-CGA	3.50	118.53	112.67
7	E	501	HEM	C4A-C3A-C2A	3.39	109.35	107.00
7	D	501	HEM	CMC-C2C-C3C	3.35	130.95	124.68
7	B	501	HEM	C4A-C3A-C2A	3.32	109.31	107.00
7	C	501	HEM	CMA-C3A-C4A	-3.12	123.67	128.46
7	B	501	HEM	CBA-CAA-C2A	3.09	118.18	112.49
7	C	501	HEM	C4A-C3A-C2A	3.03	109.11	107.00
7	C	501	HEM	CMC-C2C-C3C	3.03	130.34	124.68
7	D	501	HEM	CBA-CAA-C2A	3.02	118.06	112.49
7	E	501	HEM	CBD-CAD-C3D	-3.02	106.91	112.48
7	B	501	HEM	CBD-CAD-C3D	-2.97	107.00	112.48
7	A	501	HEM	C2C-C3C-C4C	-2.67	105.03	106.90
8	E	502	DEB	O21-C5-C6	-2.62	104.91	109.83
7	E	501	HEM	C3B-C4B-NB	-2.57	105.88	109.21
7	A	501	HEM	C1D-C2D-C3D	-2.55	105.22	107.00
7	B	501	HEM	CMC-C2C-C3C	2.51	129.38	124.68

Continued on next page...

PRELIMINARY VALIDATION REPORT

Continued from previous page...

Mol	Chain	Res	Type	Atoms	Z	Observed(°)	Ideal(°)
7	B	501	HEM	CMA-C3A-C4A	-2.36	124.84	128.46
7	B	501	HEM	CMB-C2B-C3B	2.33	129.04	124.68
7	B	501	HEM	CAA-C2A-C3A	-2.33	120.56	127.25
8	D	502	DEB	O16-C13-C14	2.32	110.63	106.92
7	D	501	HEM	CAD-CBD-CGD	-2.22	108.94	112.67
8	A	502	DEB	C25-C10-C9	2.22	111.94	108.08
7	E	501	HEM	CBA-CAA-C2A	2.22	116.58	112.49
8	A	502	DEB	C6-C5-C4	-2.22	112.84	116.27
8	B	502	DEB	O16-C13-C14	2.21	110.46	106.92
7	A	501	HEM	CAD-CBD-CGD	2.19	116.35	112.67
7	A	501	HEM	CMB-C2B-C3B	2.15	128.70	124.68
7	D	501	HEM	C2C-C3C-C4C	2.15	108.40	106.90
8	C	502	DEB	O19-C3-C2	2.14	113.79	108.82
7	D	501	HEM	C3C-C4C-NC	-2.09	106.99	110.94
7	F	501	HEM	CMC-C2C-C3C	2.06	128.53	124.68
7	D	501	HEM	CMA-C3A-C4A	-2.05	125.31	128.46

There are no chirality outliers.

All (62) torsion outliers are listed below:

Mol	Chain	Res	Type	Atoms
7	B	501	HEM	C1A-C2A-CAA-CBA
7	B	501	HEM	C3A-C2A-CAA-CBA
7	C	501	HEM	C1A-C2A-CAA-CBA
7	C	501	HEM	C3A-C2A-CAA-CBA
8	A	502	DEB	C12-C13-C14-C15
8	A	502	DEB	O16-C13-C14-C15
8	B	502	DEB	C12-C13-C14-C15
8	B	502	DEB	O16-C13-C14-C15
8	C	502	DEB	C12-C13-C14-C15
8	C	502	DEB	O16-C13-C14-C15
8	D	502	DEB	C12-C13-C14-C15
8	D	502	DEB	O16-C13-C14-C15
8	E	502	DEB	C18-C2-C3-C4
8	E	502	DEB	O16-C13-C14-C15
8	A	502	DEB	C3-C4-C5-O21
8	B	502	DEB	C3-C4-C5-O21
8	C	502	DEB	C3-C4-C5-O21
8	D	502	DEB	C3-C4-C5-O21
8	F	502	DEB	C3-C4-C5-O21
8	D	502	DEB	C20-C4-C5-O21
8	E	502	DEB	C3-C4-C5-O21

Continued on next page...

PRELIMINARY VALIDATION REPORT

Continued from previous page...

Mol	Chain	Res	Type	Atoms
8	A	502	DEB	C18-C2-C3-O19
8	C	502	DEB	C18-C2-C3-O19
8	E	502	DEB	C18-C2-C3-O19
8	F	502	DEB	C18-C2-C3-O19
8	A	502	DEB	C20-C4-C5-O21
8	F	502	DEB	C18-C2-C3-C4
8	B	502	DEB	C20-C4-C5-O21
8	C	502	DEB	C20-C4-C5-O21
8	E	502	DEB	C20-C4-C5-O21
8	F	502	DEB	C20-C4-C5-O21
8	D	502	DEB	C18-C2-C3-O19
8	D	502	DEB	C3-C4-C5-C6
8	A	502	DEB	C18-C2-C3-C4
8	B	502	DEB	C18-C2-C3-C4
8	C	502	DEB	C18-C2-C3-C4
8	D	502	DEB	C18-C2-C3-C4
8	D	502	DEB	C20-C4-C5-C6
8	B	502	DEB	C18-C2-C3-O19
8	A	502	DEB	C20-C4-C5-C6
8	C	502	DEB	C20-C4-C5-C6
8	E	502	DEB	C20-C4-C5-C6
8	B	502	DEB	C20-C4-C5-C6
8	E	502	DEB	C3-C4-C5-C6
8	A	502	DEB	C3-C4-C5-C6
8	B	502	DEB	C3-C4-C5-C6
8	C	502	DEB	C3-C4-C5-C6
8	F	502	DEB	C3-C4-C5-C6
8	E	502	DEB	C12-C13-C14-C15
8	E	502	DEB	C1-C2-C3-C4
8	E	502	DEB	C1-C2-C3-O19
8	F	502	DEB	C1-C2-C3-C4
8	F	502	DEB	C1-C2-C3-O19
8	F	502	DEB	C20-C4-C5-C6
8	A	502	DEB	C23-C8-C9-O24
8	D	502	DEB	C23-C8-C9-O24
8	E	502	DEB	C23-C8-C9-O24
8	F	502	DEB	C4-C5-C6-C22
8	A	502	DEB	C7-C8-C9-O24
8	D	502	DEB	C7-C8-C9-O24
8	E	502	DEB	C7-C8-C9-O24
8	F	502	DEB	C7-C8-C9-O24

There are no ring outliers.

PRELIMINARY VALIDATION REPORT

9 monomers are involved in 31 short contacts:

Mol	Chain	Res	Type	Clashes	Symm-Clashes
7	C	501	HEM	7	0
7	E	501	HEM	2	0
8	D	502	DEB	1	0
8	F	502	DEB	1	0
9	A	504	FMT	1	0
7	A	501	HEM	4	0
7	B	501	HEM	5	0
7	F	501	HEM	4	0
7	D	501	HEM	7	0

The following is a two-dimensional graphical depiction of Mogul quality analysis of bond lengths, bond angles, torsion angles, and ring geometry for all instances of the Ligand of Interest. In addition, ligands with molecular weight > 250 and outliers as shown on the validation Tables will also be included. For torsion angles, if less then 5% of the Mogul distribution of torsion angles is within 10 degrees of the torsion angle in question, then that torsion angle is considered an outlier. Any bond that is central to one or more torsion angles identified as an outlier by Mogul will be highlighted in the graph. For rings, the root-mean-square deviation (RMSD) between the ring in question and similar rings identified by Mogul is calculated over all ring torsion angles. If the average RMSD is greater than 60 degrees and the minimal RMSD between the ring in question and any Mogul-identified rings is also greater than 60 degrees, then that ring is considered an outlier. The outliers are highlighted in purple. The color gray indicates Mogul did not find sufficient equivalents in the CSD to analyse the geometry.

PRELIMINARY VALIDATION REPORT

Ligand HEM C 501

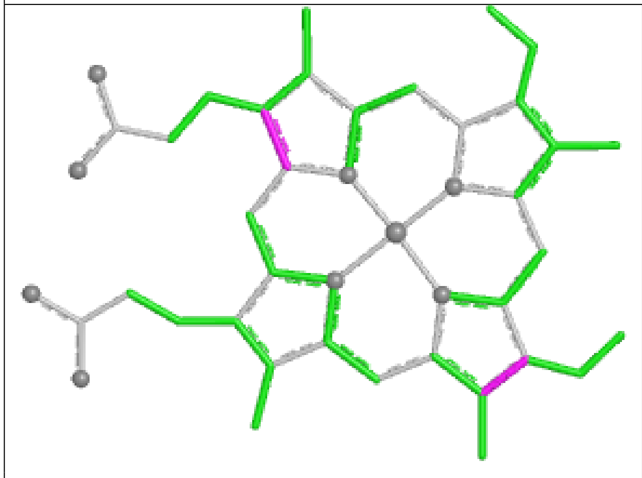

Bond lengths

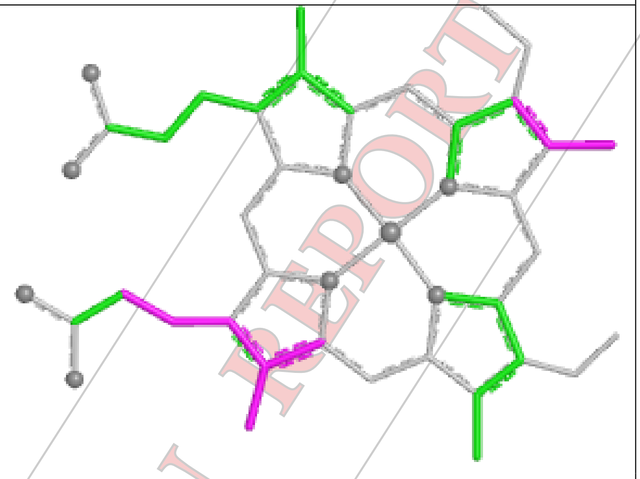

Bond angles

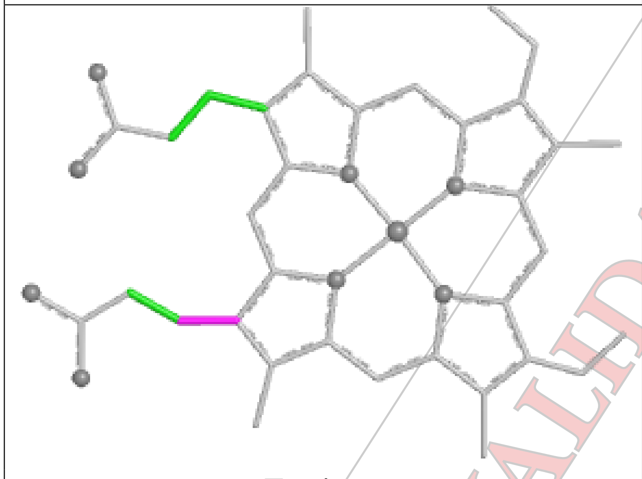

Torsions

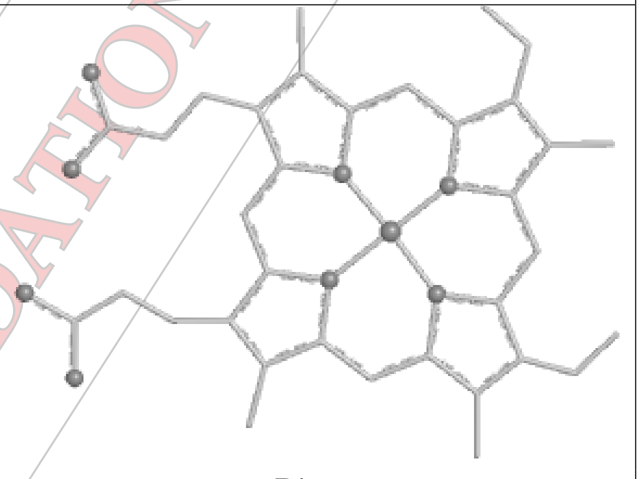

Rings

PRELIMINARY VALIDATION REPORT

Ligand HEM E 501

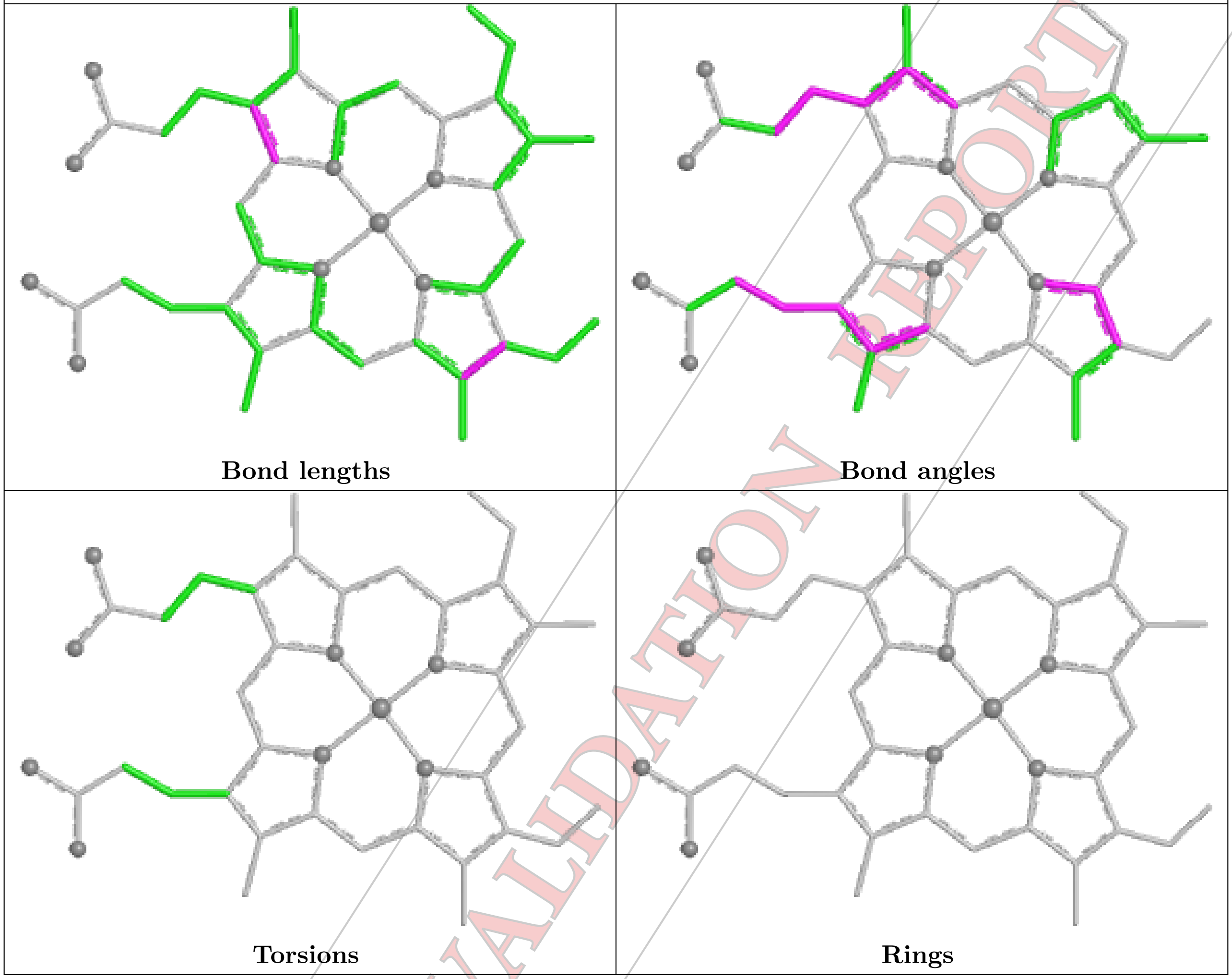

PRELIMINARY VALIDATION REPORT

Ligand DEB B 502

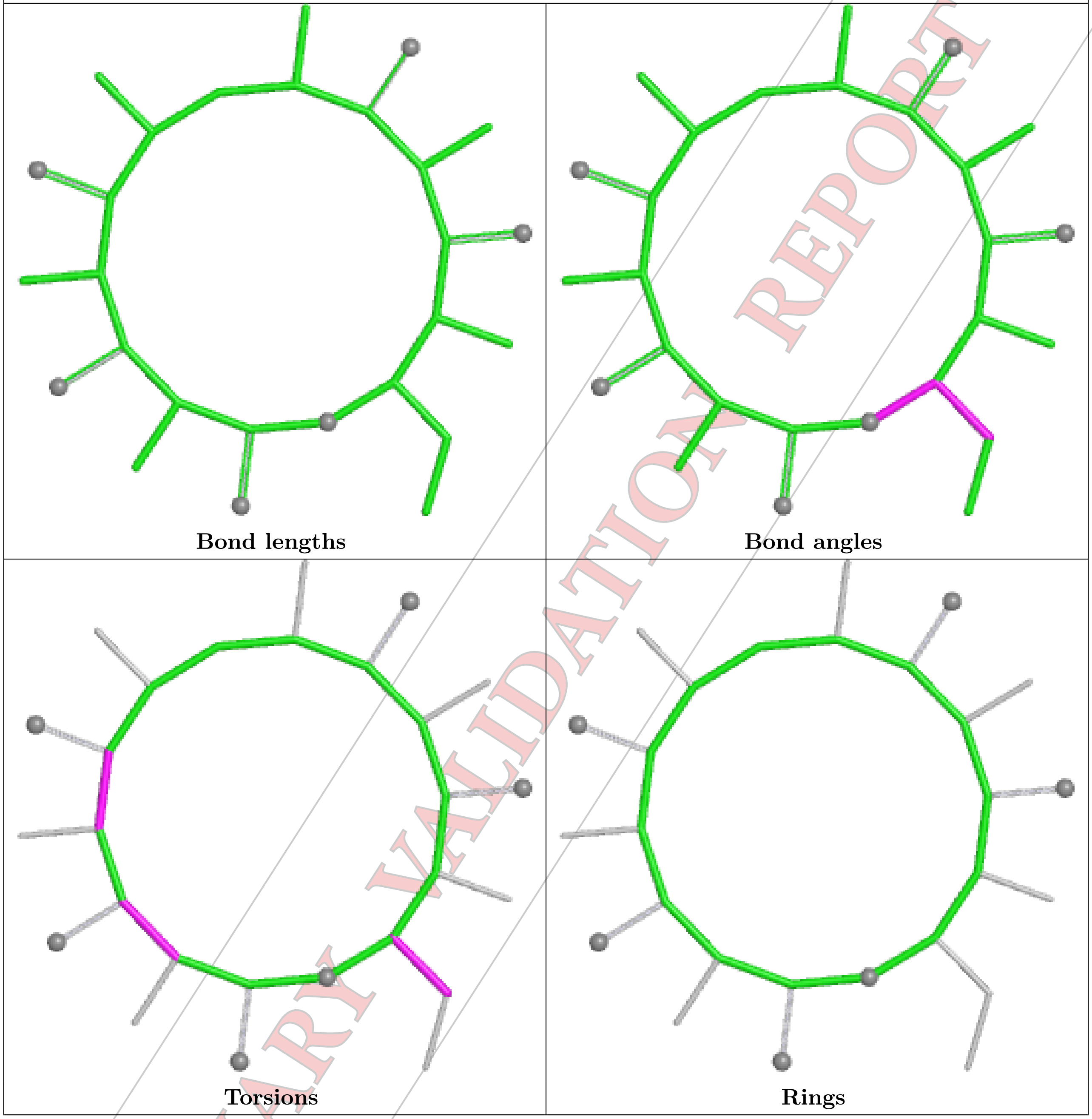

Bond lengths | Bond angles | Torsions | Rings

PRELIMINARY VALIDATION REPORT

Ligand DEB A 502	
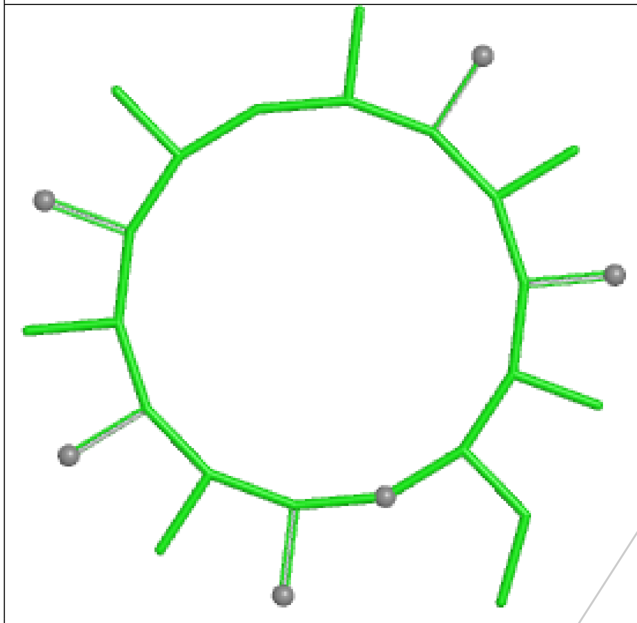 Bond lengths	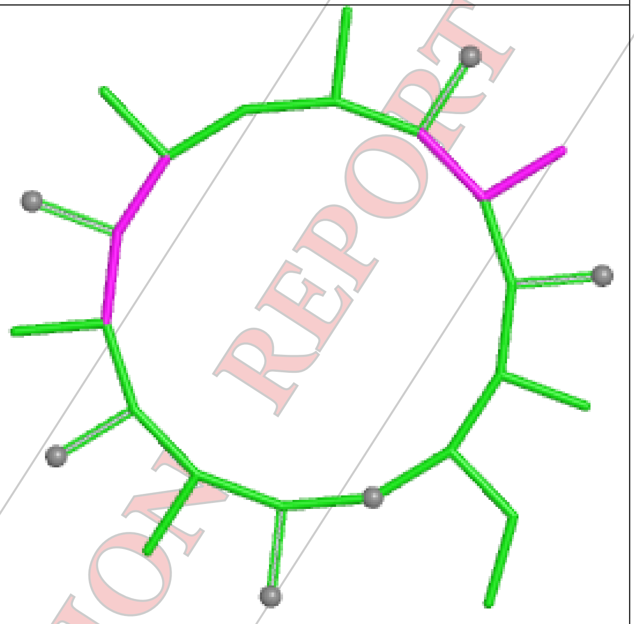 Bond angles
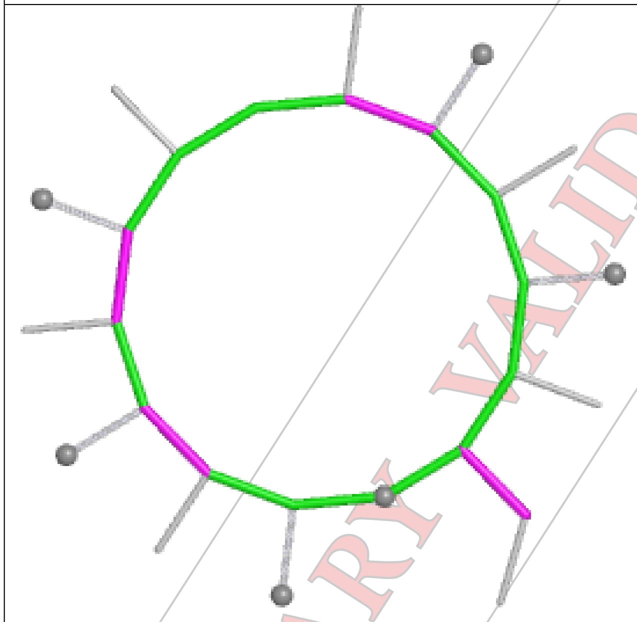 Torsions	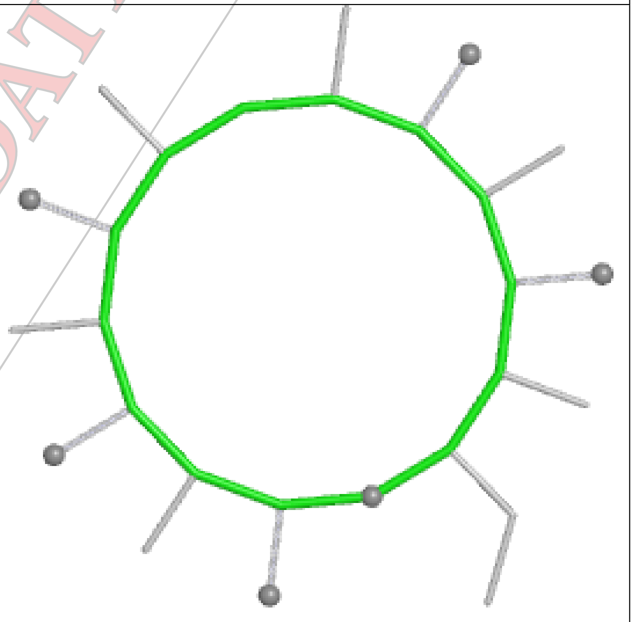 Rings

PRELIMINARY VALIDATION REPORT

Ligand DEB D 502

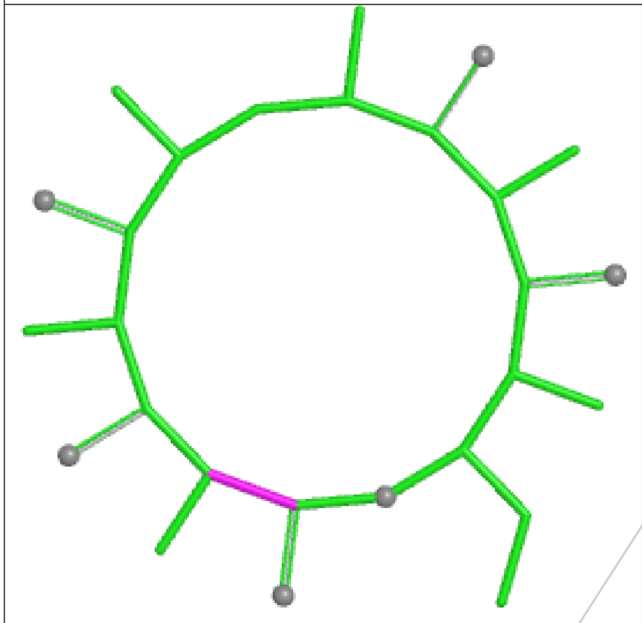 Bond lengths	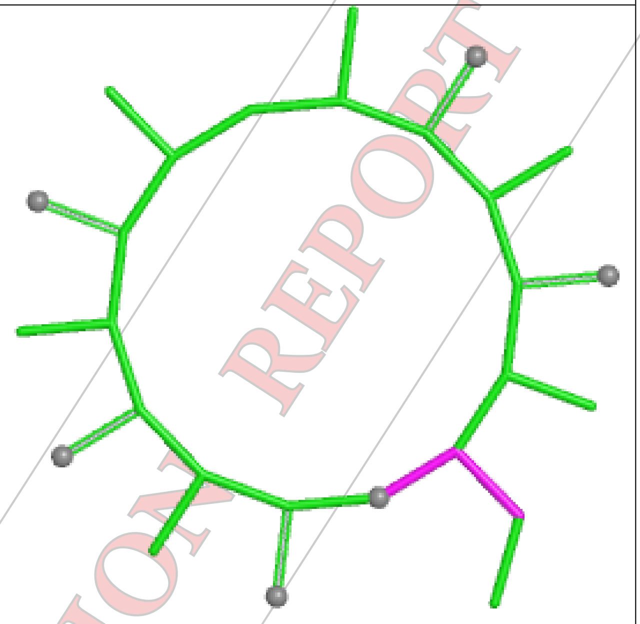 Bond angles
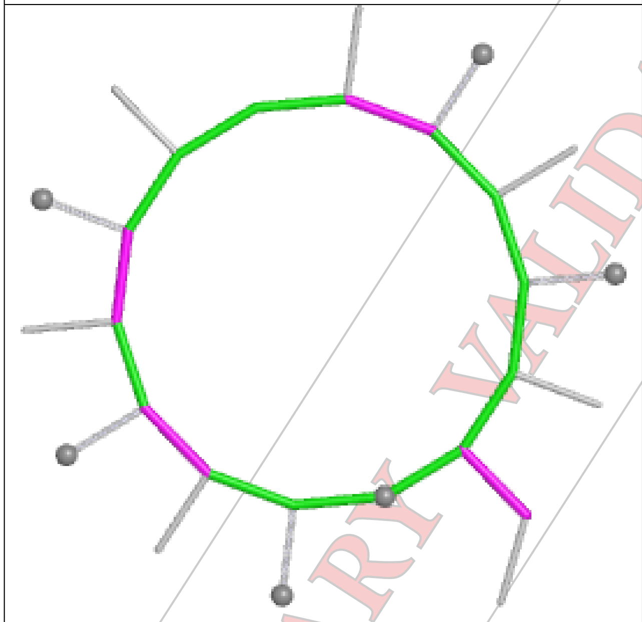 Torsions	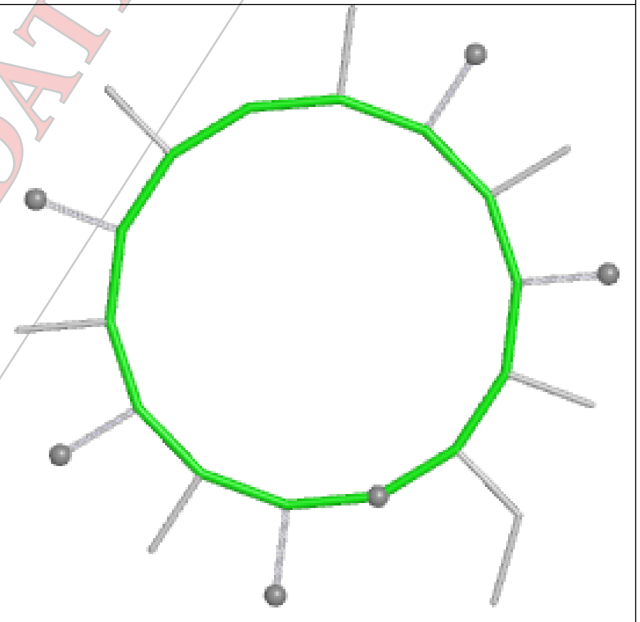 Rings

PRELIMINARY VALIDATION REPORT

Ligand DEB F 502

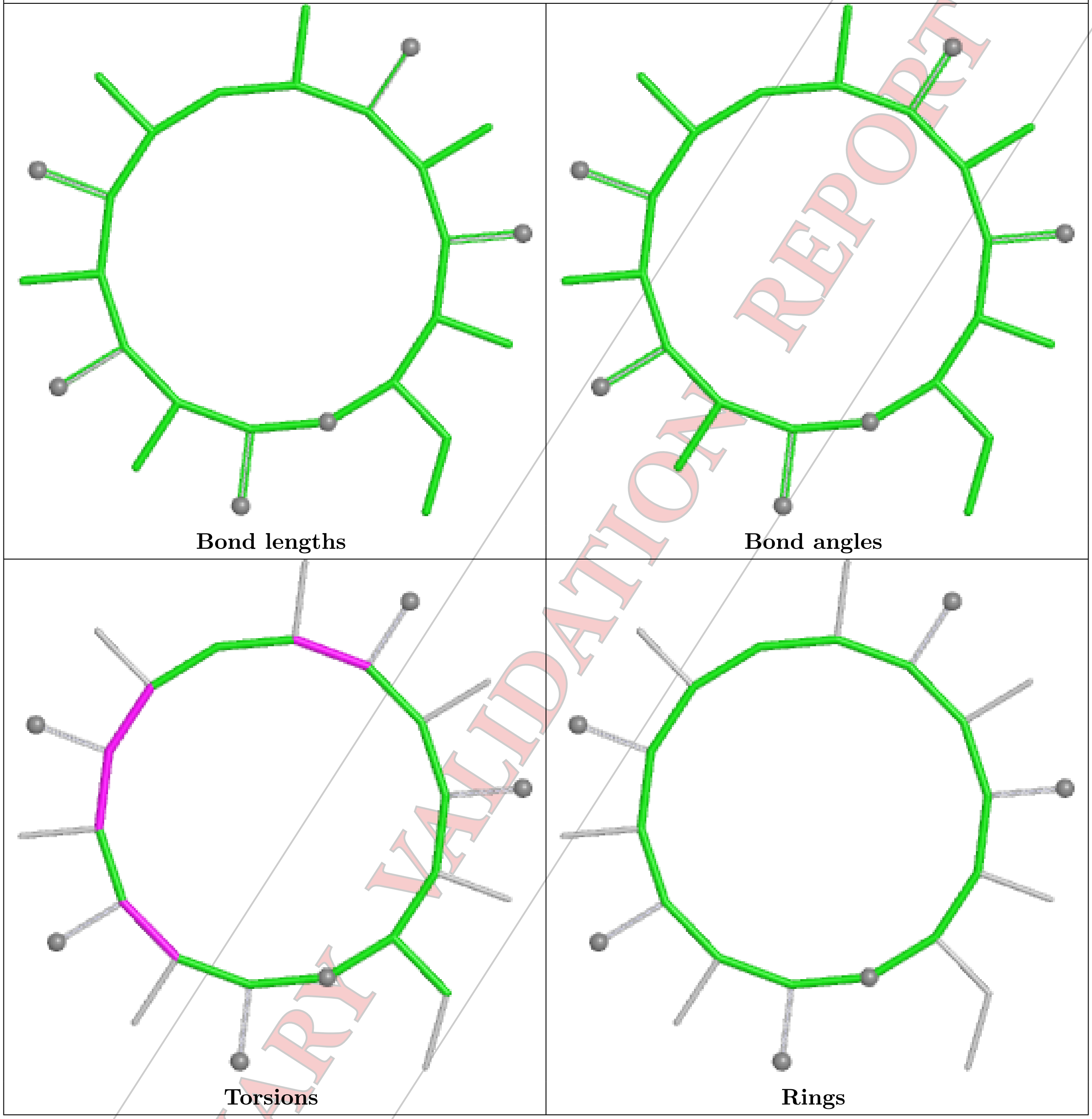

PRELIMINARY VALIDATION REPORT

Ligand HEM A 501

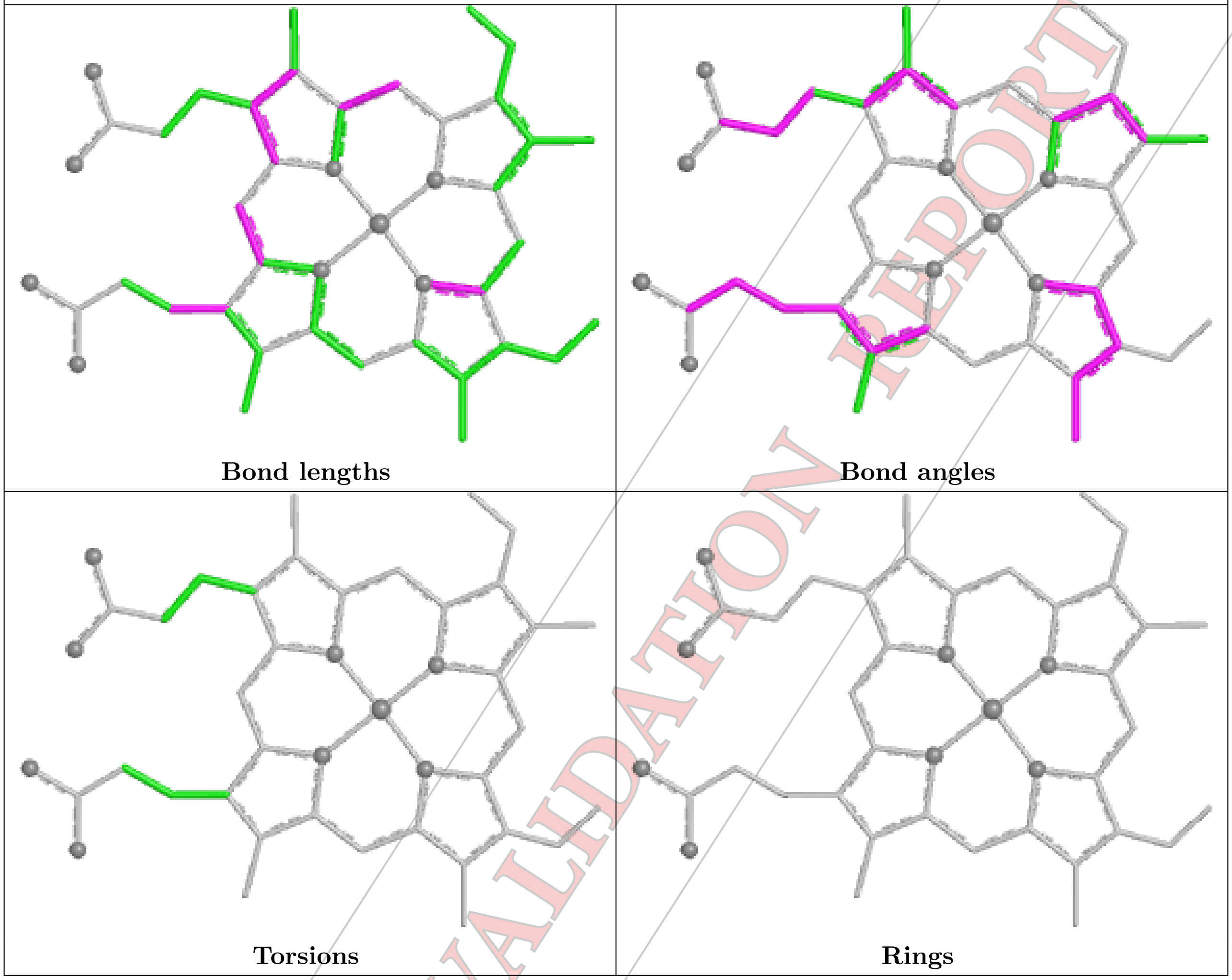

PRELIMINARY VALIDATION REPORT

Ligand HEM B 501

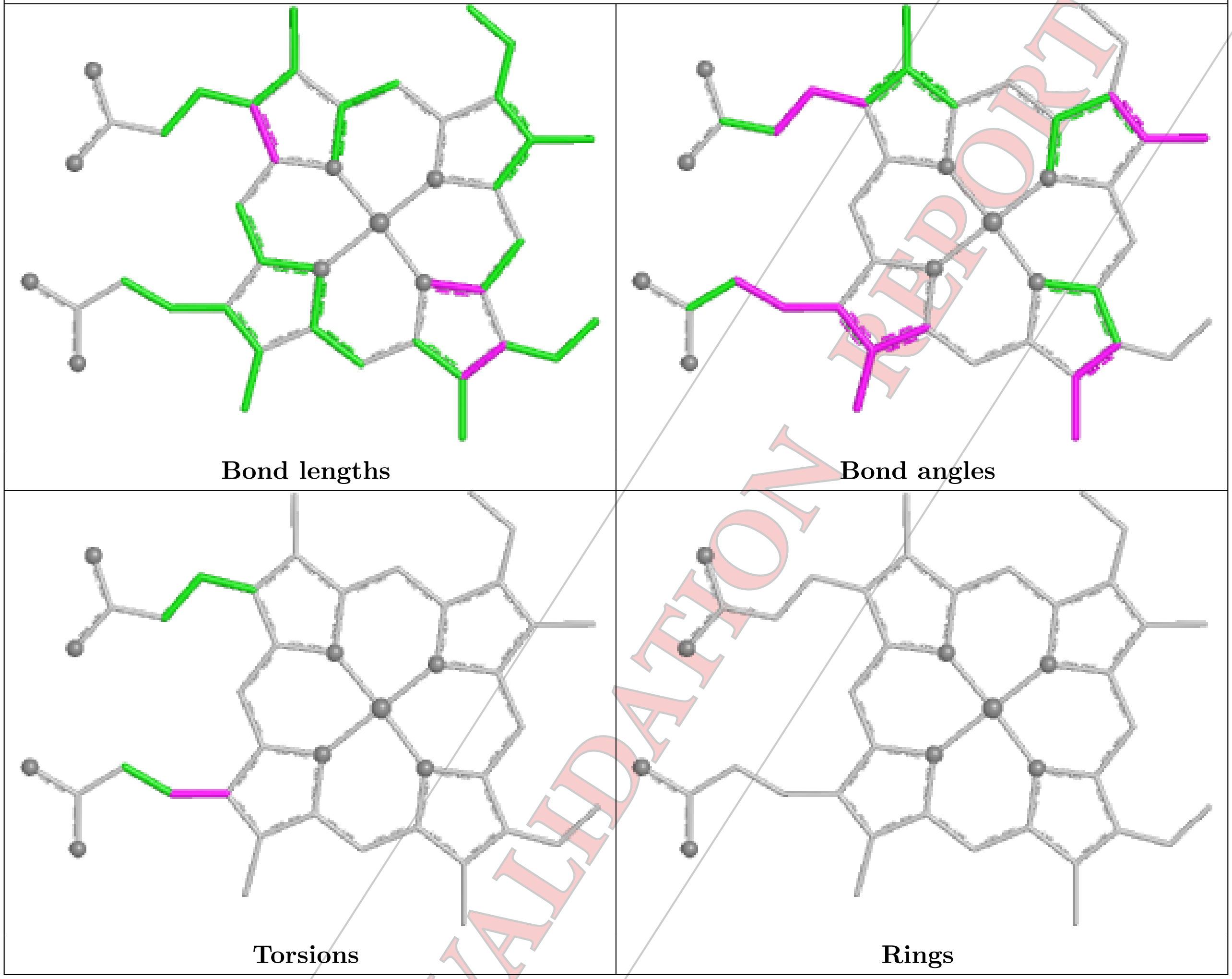

PRELIMINARY VALIDATION REPORT

WORLDWIDE PDB PROTEIN DATA BANK

Ligand DEB C 502

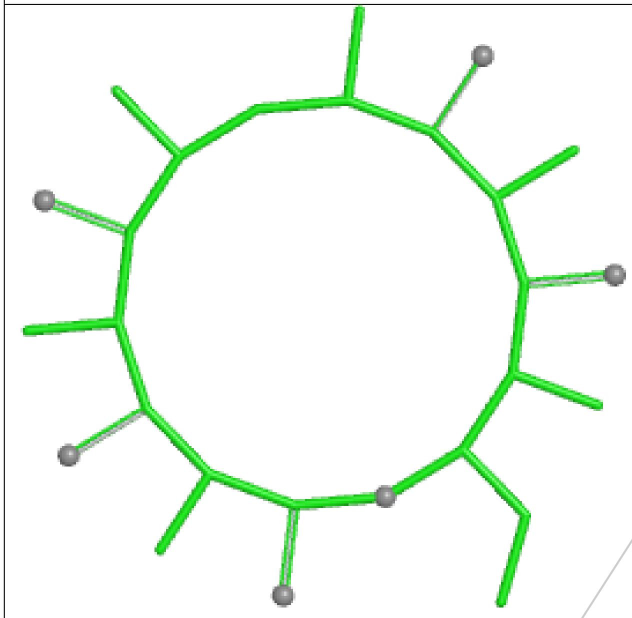

Bond lengths

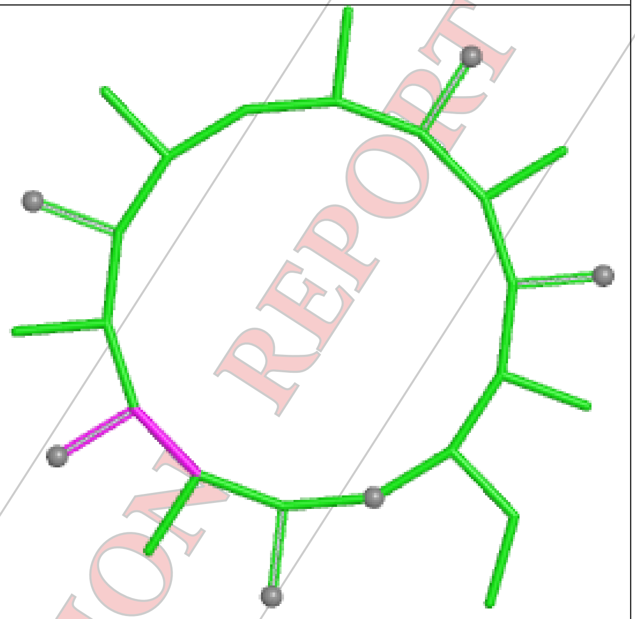

Bond angles

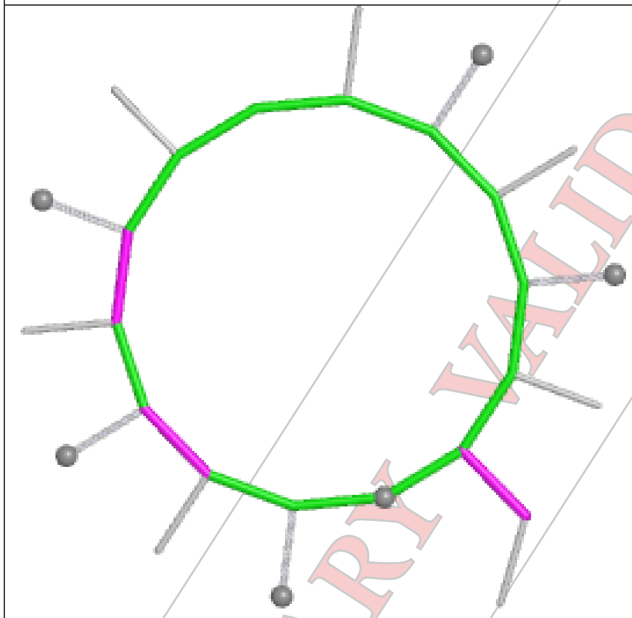

Torsions

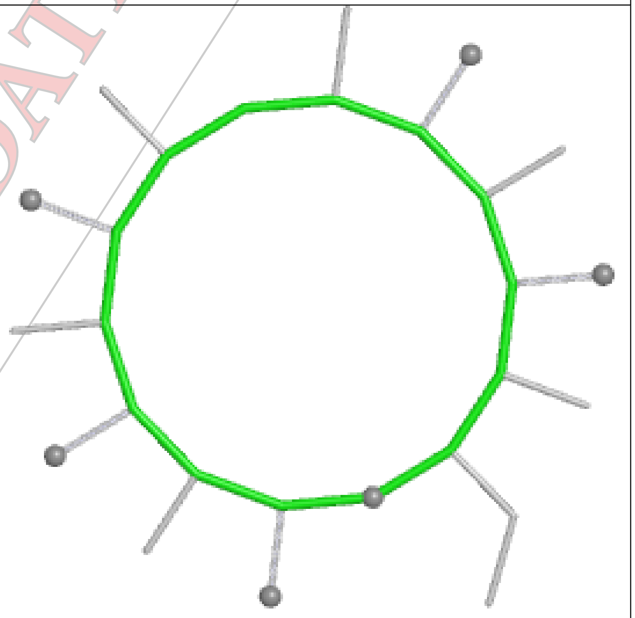

Rings

PRELIMINARY VALIDATION REPORT

Ligand DEB E 502

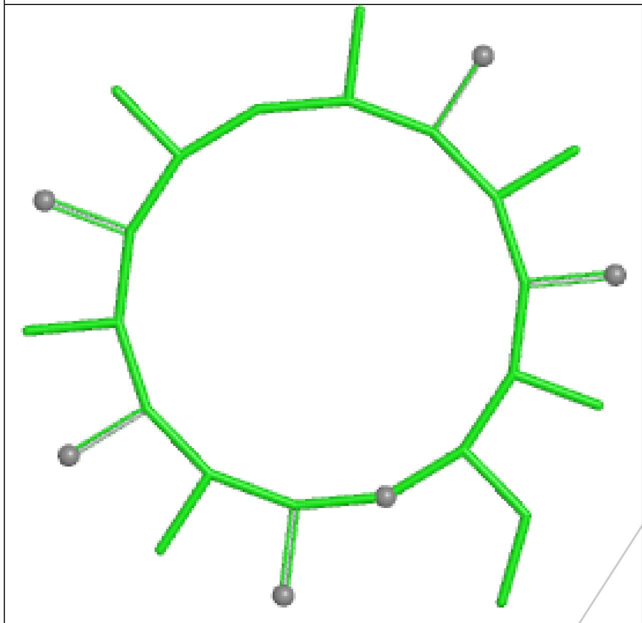

Bond lengths

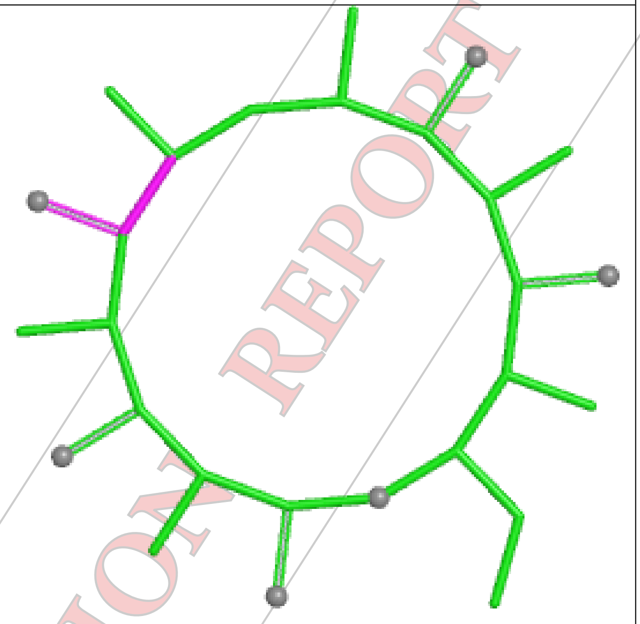

Bond angles

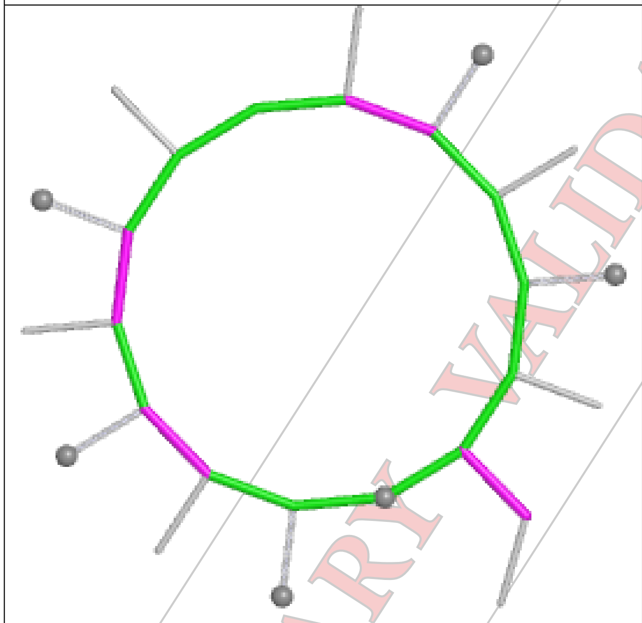

Torsions

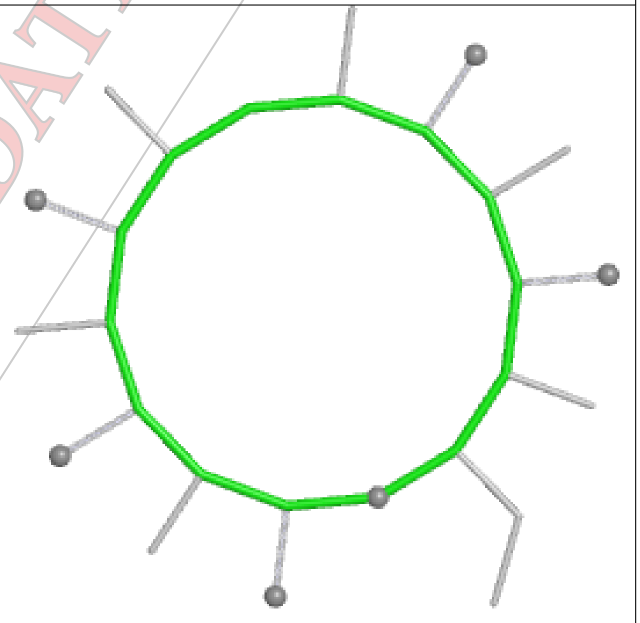

Rings

PRELIMINARY VALIDATION REPORT

Ligand HEM F 501

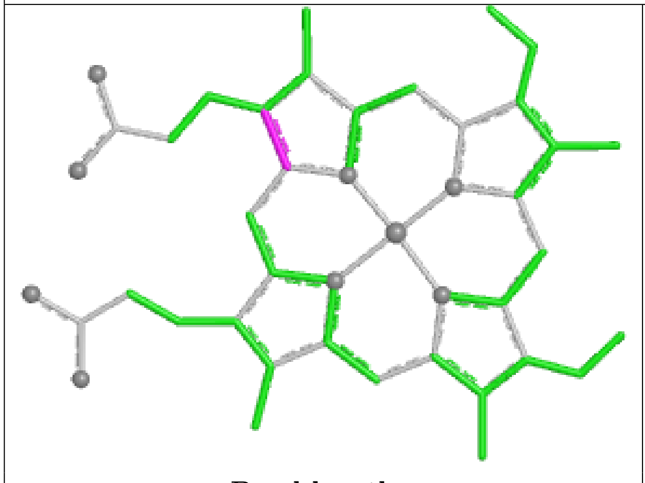

Bond lengths

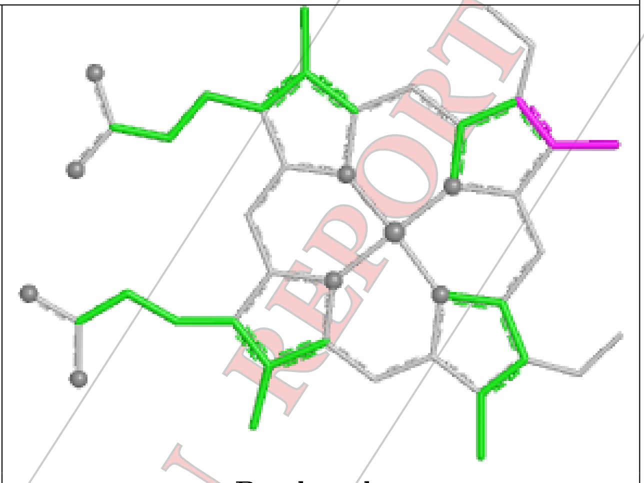

Bond angles

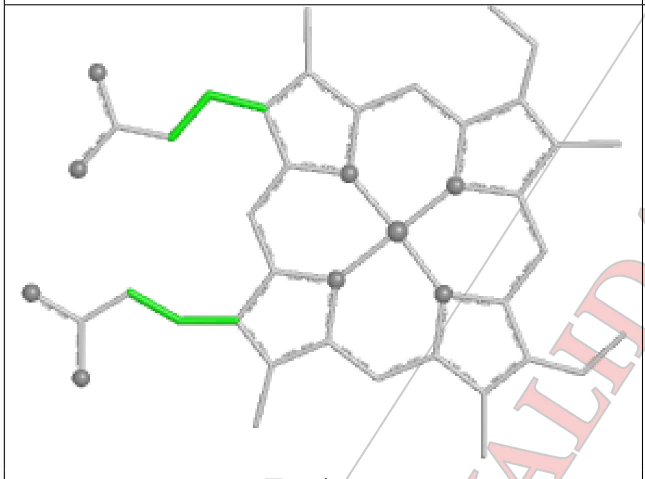

Torsions

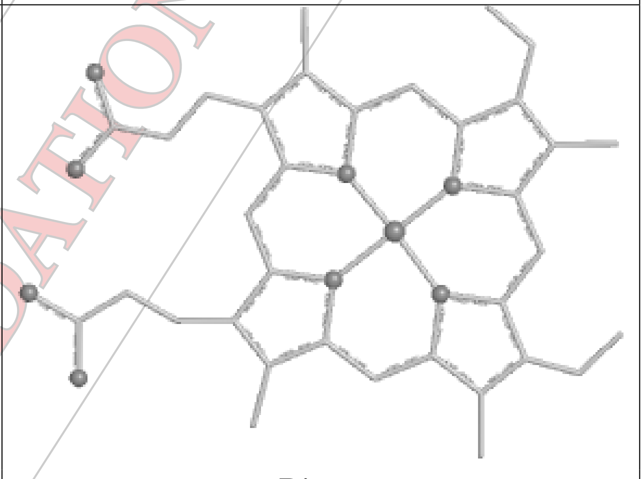

Rings

PRELIMINARY VALIDATION REPORT

Ligand HEM D 501

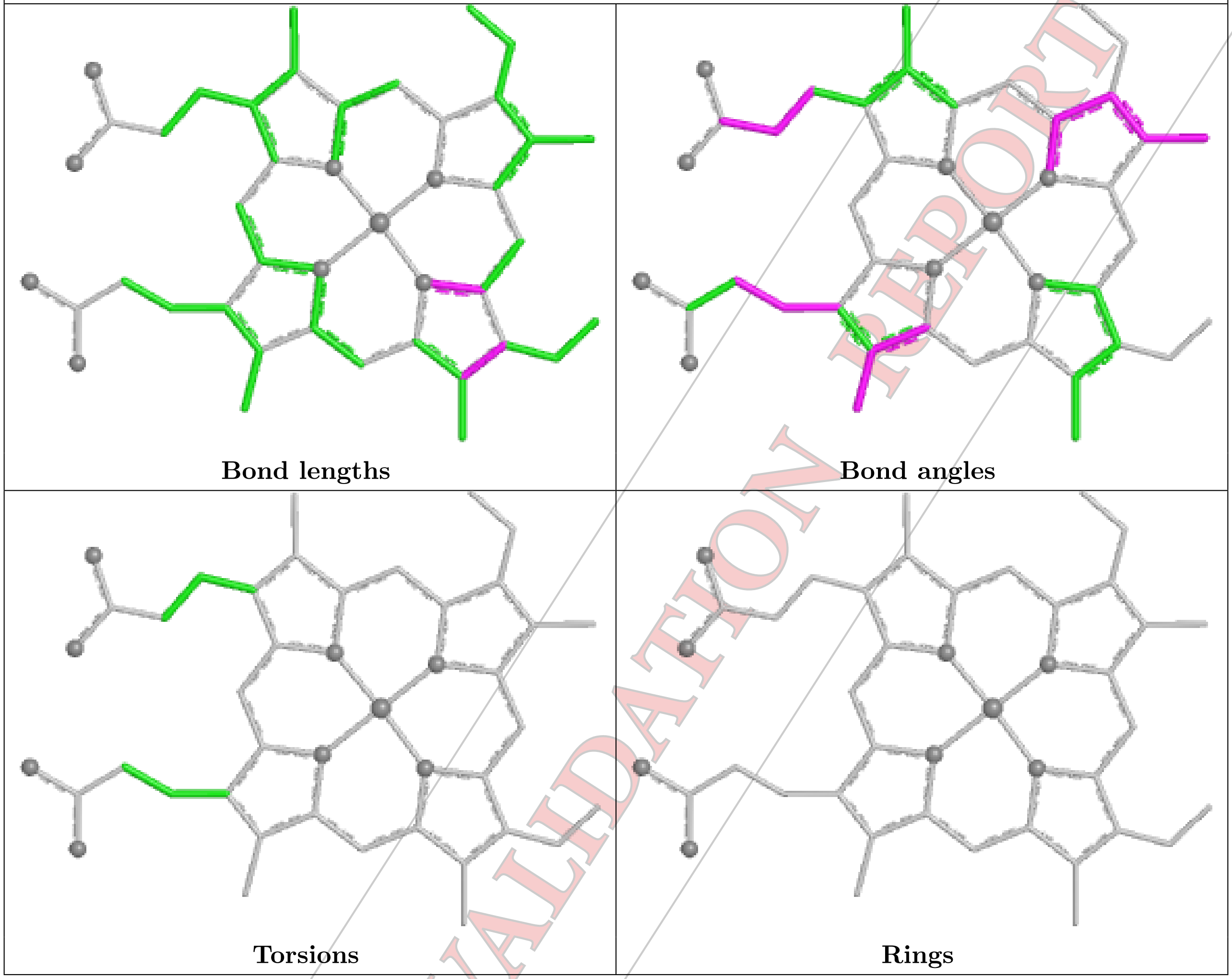

5.7 Other polymers (i)

There are no such residues in this entry.

5.8 Polymer linkage issues (i)

There are no chain breaks in this entry.

PRELIMINARY VALIDATION REPORT

6 Fit of model and data (i)

6.1 Protein, DNA and RNA chains (i)

In the following table, the column labelled '#RSRZ> 2' contains the number (and percentage) of RSRZ outliers, followed by percent RSRZ outliers for the chain as percentile scores relative to all X-ray entries and entries of similar resolution. The OWAB column contains the minimum, median, 95th percentile and maximum values of the occupancy-weighted average B-factor per residue. The column labelled 'Q< 0.9' lists the number of (and percentage) of residues with an average occupancy less than 0.9.

Mol	Chain	Analysed	<RSRZ>	#RSRZ>2			OWAB($Å^2$)	Q<0.9
1	A	397/397 (100%)	0.84	38 (9%)	8	6	40, 60, 85, 129	0
2	B	406/406 (100%)	0.78	36 (8%)	9	7	35, 59, 89, 168	0
3	C	399/399 (100%)	0.77	20 (5%)	28	27	41, 55, 75, 98	0
4	D	396/396 (100%)	1.07	63 (15%)	1	1	46, 73, 100, 148	0
5	E	395/395 (100%)	1.54	123 (31%)	0	0	51, 74, 104, 150	0
6	F	397/397 (100%)	1.93	134 (33%)	0	0	54, 80, 115, 135	0
All	All	2390/2390 (100%)	1.15	414 (17%)	1	1	35, 66, 103, 168	0

All (414) RSRZ outliers are listed below:

Mol	Chain	Res	Type	RSRZ
3	C	9[A]	ALA	20.1
3	C	10[A]	PRO	12.7
6	F	129[A]	ASP	11.9
6	F	405	VAL	10.1
6	F	277	VAL	9.8
6	F	125[A]	ARG	9.5
6	F	364	LEU	9.4
6	F	127[A]	LEU	9.2
6	F	262	THR	8.9
6	F	124	VAL	8.5
6	F	224[A]	ASP	8.4
4	D	223	THR	8.2
2	B	209[A]	ASP	8.2
5	E	23	HIS	8.1
5	E	212	THR	7.7
6	F	385	PRO	7.6
6	F	126	SER	7.4
6	F	328[A]	GLU	7.3
2	B	3	ALA	7.2

Continued on next page...

PRELIMINARY VALIDATION REPORT

Continued from previous page...

Mol	Chain	Res	Type	RSRZ
6	F	273	ALA	7.1
5	E	209	ASP	6.9
6	F	132	LEU	6.8
6	F	11	ALA	6.7
5	E	335[A]	ASP	6.6
5	E	265[A]	LYS	6.2
5	E	313	ALA	6.1
5	E	226[A]	ASP	6.0
6	F	270[A]	LEU	6.0
6	F	377	PHE	5.9
6	F	269[A]	SER	5.9
5	E	306	LEU	5.8
6	F	271	VAL	5.8
6	F	267	TYR	5.8
6	F	130	SER	5.8
6	F	327	ASP	5.8
6	F	376	ARG	5.7
6	F	141	PRO	5.6
5	E	312[A]	ARG	5.6
4	D	333	HIS	5.6
6	F	23[A]	HIS	5.6
2	B	211	PRO	5.5
5	E	207	ARG	5.5
4	D	208[A]	ARG	5.4
1	A	224[A]	ASP	5.3
4	D	227[B]	ASP	5.3
5	E	332	ASP	5.2
5	E	98	PRO	5.2
3	C	11[A]	ALA	5.2
6	F	248	THR	5.2
1	A	11	ALA	5.1
6	F	382	LEU	5.1
6	F	12	ASP	5.0
5	E	71	SER	5.0
6	F	123[A]	ARG	5.0
2	B	4	THR	5.0
5	E	90	PRO	5.0
5	E	24	ALA	5.0
6	F	263[A]	GLU	5.0
5	E	223	THR	4.9
6	F	406	SER	4.9
1	A	209[A]	ASP	4.9

Continued on next page...

PRELIMINARY VALIDATION REPORT

Continued from previous page...

Mol	Chain	Res	Type	RSRZ
6	F	400	LEU	4.8
4	D	263[A]	GLU	4.8
6	F	393[A]	GLN	4.8
6	F	272	ALA	4.7
6	F	307	SER	4.7
6	F	343[A]	ARG	4.7
6	F	58	MET	4.7
4	D	210	ALA	4.6
2	B	265[A]	LYS	4.6
6	F	332	ASP	4.6
5	E	308	THR	4.6
5	E	39	PRO	4.5
5	E	229	LEU	4.5
6	F	337[A]	LEU	4.5
4	D	205	ALA	4.4
4	D	115[A]	ARG	4.4
4	D	138[A]	HIS	4.4
5	E	305[A]	GLU	4.4
4	D	191[A]	ARG	4.4
5	E	13	ALA	4.4
6	F	340	HIS	4.4
6	F	38	GLU	4.3
5	E	60	ASP	4.3
6	F	349	PHE	4.3
6	F	402[A]	ARG	4.3
4	D	225[A]	ASN	4.3
6	F	365[A]	GLU	4.3
6	F	384[A]	GLU	4.2
5	E	327	ASP	4.2
2	B	225[A]	ASN	4.2
6	F	386	VAL	4.2
2	B	115[A]	ARG	4.2
4	D	406	SER	4.2
4	D	27[A]	LEU	4.2
5	E	211	PRO	4.1
2	B	228[A]	HIS	4.1
6	F	347	ILE	4.1
5	E	208	ARG	4.1
6	F	225[A]	ASN	4.1
5	E	64	VAL	4.1
6	F	308	THR	4.1
5	E	336[A]	GLU	4.1

Continued on next page...

PRELIMINARY VALIDATION REPORT

Continued from previous page...

Mol	Chain	Res	Type	RSRZ
4	D	137	ALA	4.1
2	B	333	HIS	4.0
4	D	224[A]	ASP	4.0
5	E	107[A]	LEU	4.0
5	E	261	LEU	4.0
4	D	209[A]	ASP	4.0
6	F	60	ASP	4.0
5	E	311	VAL	4.0
6	F	280	ALA	4.0
1	A	38[A]	GLU	4.0
2	B	272	ALA	4.0
2	B	332	ASP	4.0
6	F	131	LEU	4.0
6	F	333	HIS	3.9
6	F	108	VAL	3.9
6	F	208[A]	ARG	3.9
4	D	222	ALA	3.9
5	E	76	THR	3.9
2	B	2	ALA	3.8
6	F	388	GLY	3.8
5	E	213[A]	GLU	3.8
6	F	213[A]	GLU	3.8
5	E	110[A]	LYS	3.8
5	E	333	HIS	3.8
4	D	268	GLU	3.8
6	F	339	PHE	3.8
6	F	284[A]	MET	3.7
6	F	380	LEU	3.7
5	E	228[A]	HIS	3.7
6	F	276	LEU	3.7
6	F	140	SER	3.7
5	E	189	ILE	3.6
2	B	342[A]	GLU	3.6
6	F	37	ASP	3.6
6	F	188[A]	GLU	3.6
2	B	214	ASP	3.6
5	E	382	LEU	3.6
6	F	117	VAL	3.6
5	E	271	VAL	3.6
5	E	304	VAL	3.6
5	E	302[A]	GLU	3.6
6	F	375[A]	ARG	3.5

Continued on next page...

PRELIMINARY VALIDATION REPORT

Continued from previous page...

Mol	Chain	Res	Type	RSRZ
5	E	37	ASP	3.5
5	E	385	PRO	3.5
6	F	379	THR	3.5
4	D	270	LEU	3.5
2	B	222	ALA	3.5
4	D	266	ARG	3.4
1	A	267	TYR	3.4
1	A	220	ALA	3.4
3	C	222	ALA	3.4
6	F	128	VAL	3.4
4	D	123[A]	ARG	3.4
6	F	371	SER	3.3
5	E	383	ALA	3.3
4	D	271	VAL	3.3
6	F	244	ALA	3.3
3	C	328[A]	GLU	3.3
1	A	210	ALA	3.3
1	A	211	PRO	3.3
5	E	338	ASP	3.3
4	D	393[A]	GLN	3.3
4	D	332	ASP	3.3
5	E	309	VAL	3.3
5	E	22	PRO	3.3
1	A	262	THR	3.3
4	D	342[A]	GLU	3.3
5	E	106[A]	ARG	3.3
4	D	220	ALA	3.3
2	B	10	PRO	3.3
6	F	330	VAL	3.2
1	A	328[A]	GLU	3.2
1	A	271	VAL	3.2
6	F	309	VAL	3.2
5	E	210	ALA	3.2
6	F	215	LEU	3.2
5	E	73	ALA	3.2
6	F	342[A]	GLU	3.2
6	F	183[A]	ARG	3.2
5	E	287	TYR	3.2
4	D	141	PRO	3.1
5	E	342[A]	GLU	3.1
5	E	77	ASP	3.1
5	E	136	VAL	3.1

Continued on next page...

PRELIMINARY VALIDATION REPORT

Continued from previous page...

Mol	Chain	Res	Type	RSRZ
3	C	188[A]	GLU	3.1
6	F	36[A]	ARG	3.1
4	D	407	TRP	3.1
6	F	191[A]	ARG	3.1
2	B	136[A]	VAL	3.1
2	B	5	HIS	3.1
6	F	268[A]	GLU	3.1
6	F	142	ALA	3.1
6	F	146[A]	GLU	3.1
1	A	228[A]	HIS	3.1
5	E	268	GLU	3.0
6	F	61	ALA	3.0
6	F	287	TYR	3.0
5	E	163	VAL	3.0
5	E	25	LEU	3.0
6	F	165	LEU	3.0
6	F	383	ALA	3.0
6	F	118	GLU	3.0
4	D	107[A]	LEU	3.0
6	F	21[A]	LEU	3.0
6	F	329[A]	GLU	3.0
1	A	342[A]	GLU	3.0
6	F	245	GLY	3.0
6	F	278	PRO	3.0
2	B	406	SER	3.0
4	D	12	ALA	3.0
5	E	337	LEU	2.9
3	C	307	SER	2.9
1	A	13	ALA	2.9
5	E	264	ARG	2.9
6	F	173	THR	2.9
2	B	210	ALA	2.9
5	E	227[A]	ASP	2.9
1	A	141	PRO	2.9
5	E	307	SER	2.9
6	F	119[A]	GLU	2.9
1	A	123[A]	ARG	2.9
5	E	159	GLU	2.9
6	F	27	LEU	2.8
6	F	389	LEU	2.8
5	E	129	ASP	2.8
3	C	271	VAL	2.8

Continued on next page...

PRELIMINARY VALIDATION REPORT

Continued from previous page...

Mol	Chain	Res	Type	RSRZ
6	F	331	PHE	2.8
2	B	109	GLY	2.8
2	B	235	VAL	2.8
5	E	42	ARG	2.8
5	E	299	VAL	2.8
1	A	76	THR	2.8
3	C	223	THR	2.8
6	F	395	MET	2.8
1	A	206[A]	GLN	2.8
5	E	328[A]	GLU	2.8
2	B	327	ASP	2.8
1	A	221	LEU	2.8
4	D	219	LEU	2.8
6	F	110[A]	LYS	2.8
4	D	108	VAL	2.7
6	F	336[A]	GLU	2.7
4	D	261	LEU	2.7
5	E	389	LEU	2.7
1	A	329	GLU	2.7
6	F	261	LEU	2.7
5	E	141	PRO	2.7
6	F	346	HIS	2.7
5	E	33[A]	GLU	2.7
1	A	407	TRP	2.7
5	E	303	ASP	2.7
6	F	174	PHE	2.7
2	B	221	LEU	2.7
1	A	335[A]	ASP	2.7
1	A	334	ALA	2.7
6	F	229	LEU	2.7
3	C	343[A]	ARG	2.7
5	E	391	TRP	2.6
3	C	308	THR	2.6
3	C	332	ASP	2.6
6	F	274	ALA	2.6
5	E	277	VAL	2.6
5	E	257	VAL	2.6
5	E	34	LEU	2.6
4	D	327	ASP	2.6
3	C	36	ARG	2.6
4	D	334	ALA	2.6
1	A	12	ASP	2.6

Continued on next page...

PRELIMINARY VALIDATION REPORT

Continued from previous page...

Mol	Chain	Res	Type	RSRZ
4	D	183[A]	ARG	2.6
5	E	272	ALA	2.5
6	F	407	TRP	2.5
1	A	386	VAL	2.5
5	E	388	GLY	2.5
4	D	49[A]	GLU	2.5
2	B	357	ILE	2.5
5	E	244	ALA	2.5
6	F	260	LEU	2.5
4	D	182	THR	2.5
5	E	258	HIS	2.5
2	B	359	ALA	2.5
2	B	224[A]	ASP	2.5
6	F	288	THR	2.5
5	E	43	VAL	2.5
5	E	20	SER	2.5
6	F	83	MET	2.5
2	B	268	GLU	2.4
6	F	390[A]	LYS	2.4
6	F	206[A]	GLN	2.4
4	D	226[A]	ASP	2.4
5	E	318	VAL	2.4
5	E	339	PHE	2.4
5	E	69[A]	ARG	2.4
5	E	222	ALA	2.4
5	E	379	THR	2.4
1	A	377	PHE	2.4
6	F	370	LEU	2.4
4	D	267	TYR	2.4
5	E	27	LEU	2.4
6	F	148	LEU	2.4
3	C	407	TRP	2.4
4	D	44[A]	ARG	2.4
5	E	263	GLU	2.4
4	D	142	ALA	2.4
5	E	48	GLY	2.4
4	D	36[A]	ARG	2.4
4	D	120	MET	2.3
1	A	182	THR	2.3
5	E	387	ALA	2.3
5	E	66	GLY	2.3
4	D	147	PHE	2.3

Continued on next page...

PRELIMINARY VALIDATION REPORT

Continued from previous page...

Mol	Chain	Res	Type	RSRZ
3	C	333	HIS	2.3
6	F	46	PRO	2.3
6	F	338	ASP	2.3
5	E	300	ALA	2.3
6	F	264	ARG	2.3
4	D	336[A]	GLU	2.3
4	D	381	ASP	2.3
5	E	276	LEU	2.3
5	E	61	ALA	2.3
5	E	205	ALA	2.3
5	E	321	PHE	2.3
6	F	326	ARG	2.3
4	D	217	GLY	2.3
6	F	265[A]	LYS	2.3
4	D	38[A]	GLU	2.3
2	B	335[A]	ASP	2.3
3	C	184	LEU	2.3
5	E	406	SER	2.3
6	F	41	SER	2.3
2	B	226[A]	ASP	2.3
6	F	314	GLY	2.3
4	D	184	LEU	2.3
4	D	228[A]	HIS	2.3
5	E	142	ALA	2.3
3	C	208[A]	ARG	2.3
4	D	394	GLY	2.2
6	F	22	PRO	2.2
5	E	89	GLU	2.2
4	D	47	TYR	2.2
5	E	381	ASP	2.2
6	F	92	GLY	2.2
5	E	38	GLU	2.2
1	A	189	ILE	2.2
6	F	93	VAL	2.2
5	E	29	PRO	2.2
1	A	341	ARG	2.2
6	F	313	ALA	2.2
1	A	37	ASP	2.2
5	E	260	LEU	2.2
6	F	144[A]	LEU	2.2
2	B	11	ALA	2.2
3	C	272	ALA	2.2

Continued on next page...

PRELIMINARY VALIDATION REPORT

Continued from previous page...

Mol	Chain	Res	Type	RSRZ
4	D	50	GLY	2.2
6	F	404	ILE	2.2
5	E	298	ARG	2.2
3	C	52	ALA	2.2
5	E	134	ASP	2.2
6	F	177	ALA	2.2
6	F	182	THR	2.2
2	B	147	PHE	2.2
5	E	21	LEU	2.2
4	D	329[A]	GLU	2.2
4	D	202	GLY	2.2
6	F	210	ALA	2.2
2	B	208[A]	ARG	2.2
5	E	267	TYR	2.2
4	D	163	VAL	2.2
6	F	249	SER	2.2
5	E	57[A]	ARG	2.2
4	D	277	VAL	2.1
6	F	79	ALA	2.1
5	E	158	CYS	2.1
5	E	72	THR	2.1
4	D	189	ILE	2.1
5	E	31	TYR	2.1
6	F	31	TYR	2.1
5	E	301	THR	2.1
1	A	332	ASP	2.1
1	A	337	LEU	2.1
5	E	405	VAL	2.1
5	E	378	PRO	2.1
5	E	230	THR	2.1
4	D	231[A]	LYS	2.1
1	A	183	ARG	2.1
3	C	381	ASP	2.1
5	E	97	ASP	2.1
1	A	107	LEU	2.1
1	A	42[A]	ARG	2.1
5	E	273	ASP	2.1
6	F	150	VAL	2.1
6	F	293	ALA	2.1
1	A	115[A]	ARG	2.1
1	A	83	MET	2.1
2	B	227[A]	ASP	2.1

Continued on next page...

PRELIMINARY VALIDATION REPORT

Continued from previous page...

Mol	Chain	Res	Type	RSRZ
6	F	169[A]	ASP	2.1
6	F	34	LEU	2.0
2	B	116[A]	ARG	2.0
5	E	36[A]	ARG	2.0
4	D	262	THR	2.0
5	E	108	VAL	2.0
6	F	47	TYR	2.0
5	E	86	THR	2.0
5	E	407	TRP	2.0
5	E	215[A]	LEU	2.0
4	D	192	VAL	2.0
2	B	230	THR	2.0
4	D	216	LEU	2.0
5	E	340	HIS	2.0
1	A	192	VAL	2.0
5	E	204	VAL	2.0
5	E	386	VAL	2.0

6.2 Non-standard residues in protein, DNA, RNA chains (i)

There are no non-standard protein/DNA/RNA residues in this entry.

6.3 Carbohydrates (i)

There are no monosaccharides in this entry.

6.4 Ligands (i)

In the following table, the Atoms column lists the number of modelled atoms in the group and the number defined in the chemical component dictionary. The B-factors column lists the minimum, median, 95th percentile and maximum values of B factors of atoms in the group. The column labelled 'Q< 0.9' lists the number of atoms with occupancy less than 0.9.

Mol	Type	Chain	Res	Atoms	RSCC	RSR	B-factors(Å^2)	Q<0.9
9	FMT	H	3	3/?	0.51	0.49	81,81,93,93	0
9	FMT	H	1	3/?	0.59	0.31	96,96,97,100	0
9	FMT	A	504	3/?	0.61	0.42	91,91,94,98	0
9	FMT	H	2	3/?	0.65	0.35	98,98,101,102	0
8	DEB	F	502	27/?	0.73	0.32	66,74,81,87	0
9	FMT	A	503	3/?	0.76	0.17	87,87,92,93	0

Continued on next page...

PRELIMINARY VALIDATION REPORT

Continued from previous page...

Mol	Type	Chain	Res	Atoms	RSCC	RSR	B-factors($Å^2$)	Q<0.9
7	HEM	E	501	43/?	0.79	0.24	44,51,57,58	0
7	HEM	F	501	43/?	0.83	0.29	55,62,69,75	0
8	DEB	E	502	27/?	0.83	0.28	58,63,67,68	0
8	DEB	C	502	27/?	0.85	0.28	46,52,56,57	0
9	FMT	A	505	3/?	0.86	0.26	59,59,62,66	0
8	DEB	D	502	27/?	0.87	0.32	58,64,68,78	0
8	DEB	A	502	27/?	0.88	0.28	45,49,52,54	0
8	DEB	B	502	27/?	0.92	0.28	47,50,55,57	0
7	HEM	A	501	43/?	0.93	0.21	38,42,47,48	0
7	HEM	D	501	43/?	0.93	0.24	40,43,55,61	0
7	HEM	B	501	43/?	0.94	0.23	36,40,49,52	0
7	HEM	C	501	43/?	0.95	0.23	39,43,48,54	0

The following is a graphical depiction of the model fit to experimental electron density of all instances of the Ligand of Interest. In addition, ligands with molecular weight > 250 and outliers as shown on the geometry validation Tables will also be included. Each fit is shown from different orientation to approximate a three-dimensional view.

Electron density around DEB F 502:

2mF$_o$-DF$_c$ (at 0.7 rmsd) in gray
mF$_o$-DF$_c$ (at 3 rmsd) in purple (negative)
and green (positive)

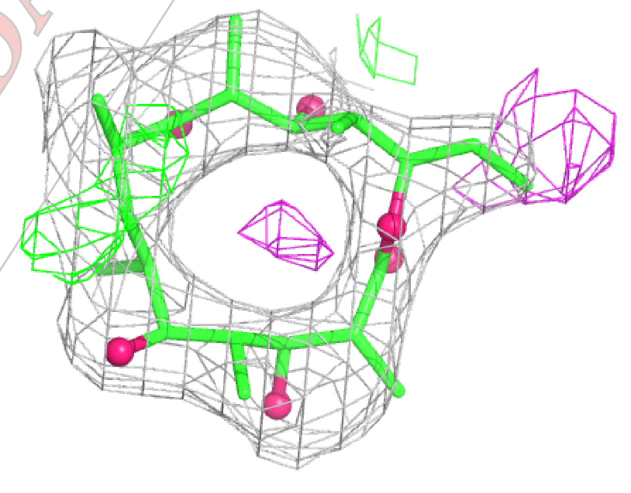

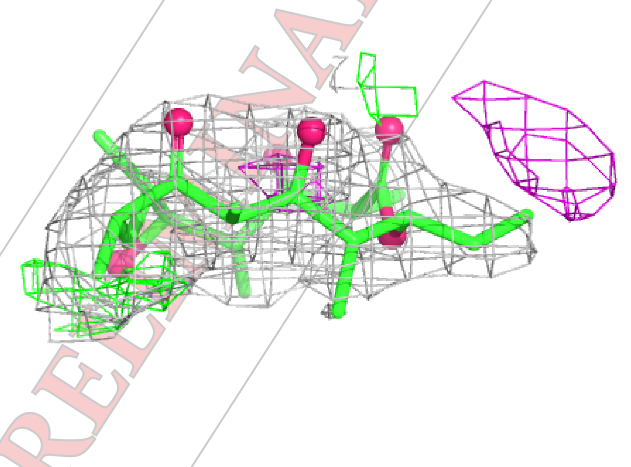

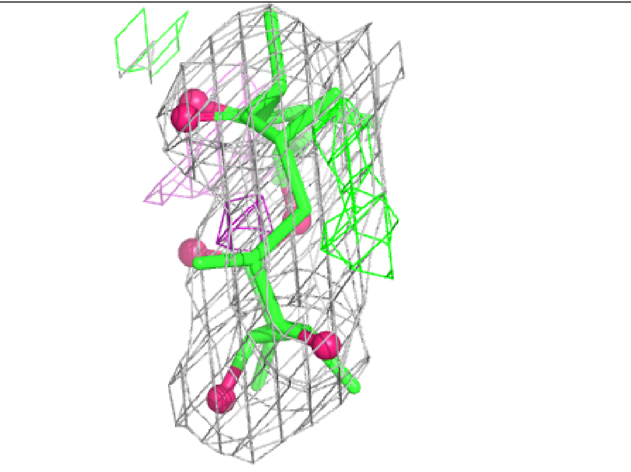

PRELIMINARY VALIDATION REPORT

Electron density around HEM E 501:

$2mF_o$-DF_c (at 0.7 rmsd) in gray
mF_o-DF_c (at 3 rmsd) in purple (negative)
and green (positive)

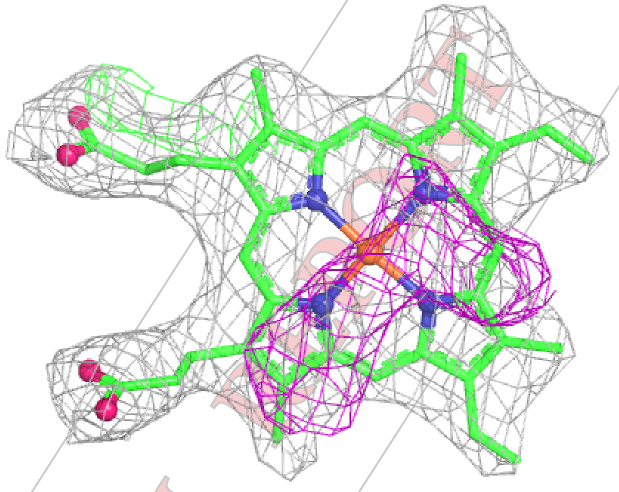

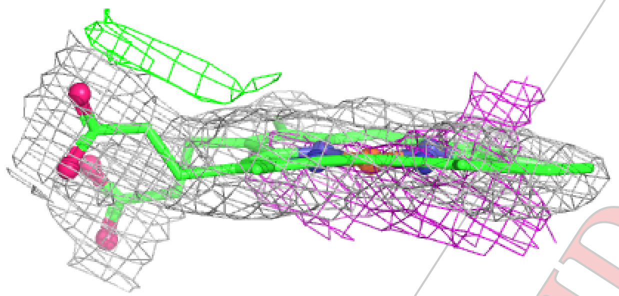

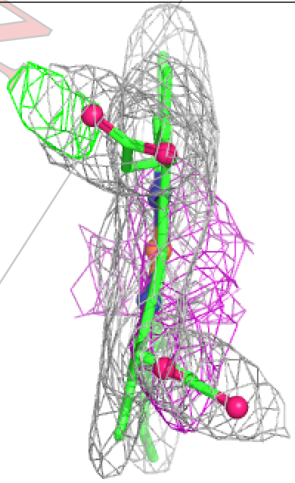

PRELIMINARY VALIDATION REPORT

Electron density around HEM F 501:

$2mF_o$-DF_c (at 0.7 rmsd) in gray
mF_o-DF_c (at 3 rmsd) in purple (negative)
and green (positive)

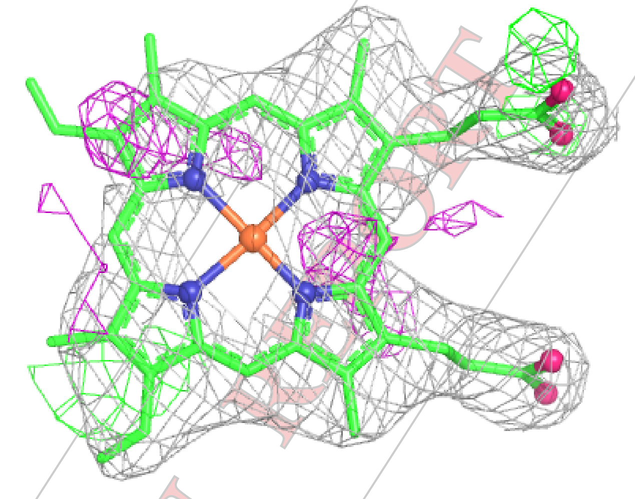

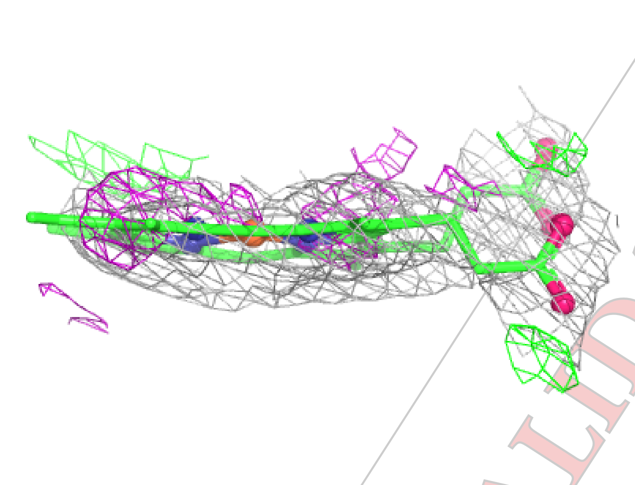

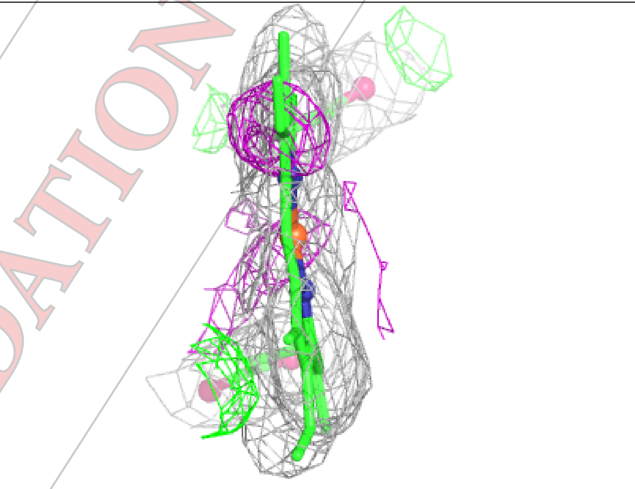

PRELIMINARY VALIDATION REPORT

Electron density around DEB E 502:

$2mF_o$-DF_c (at 0.7 rmsd) in gray
mF_o-DF_c (at 3 rmsd) in purple (negative)
and green (positive)

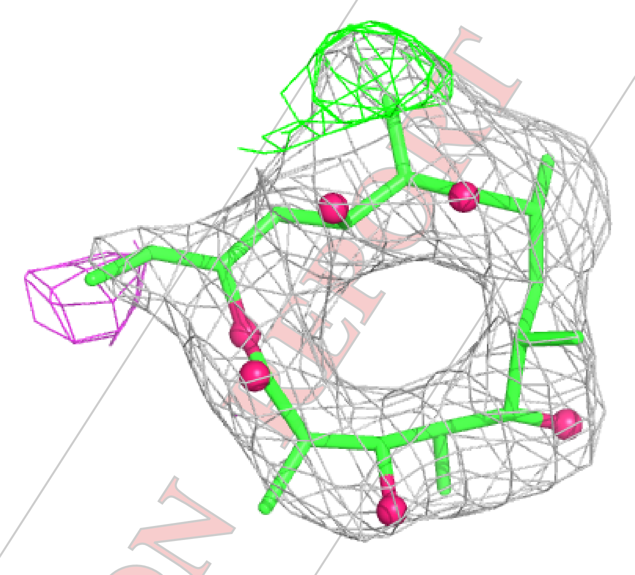

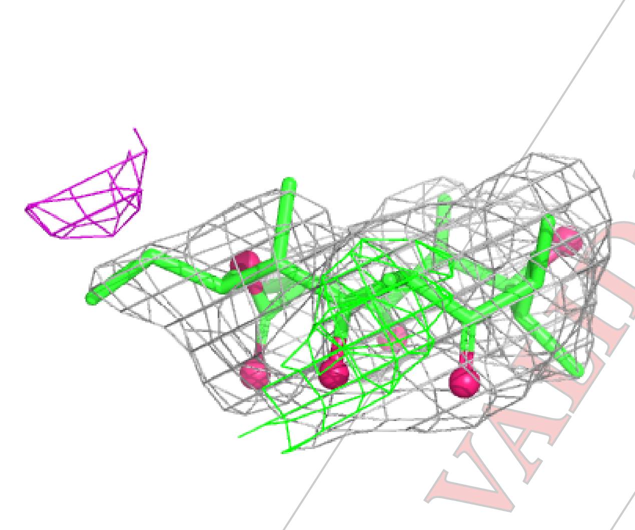

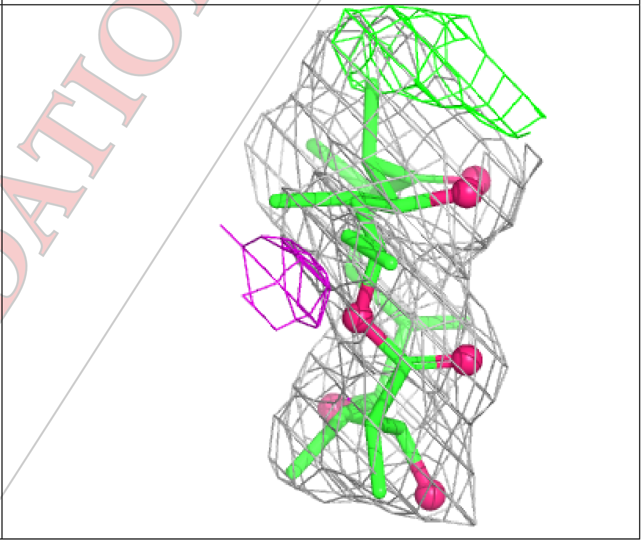

PRELIMINARY VALIDATION REPORT

Electron density around DEB C 502:

$2mF_o$-DF_c (at 0.7 rmsd) in gray
mF_o-DF_c (at 3 rmsd) in purple (negative)
and green (positive)

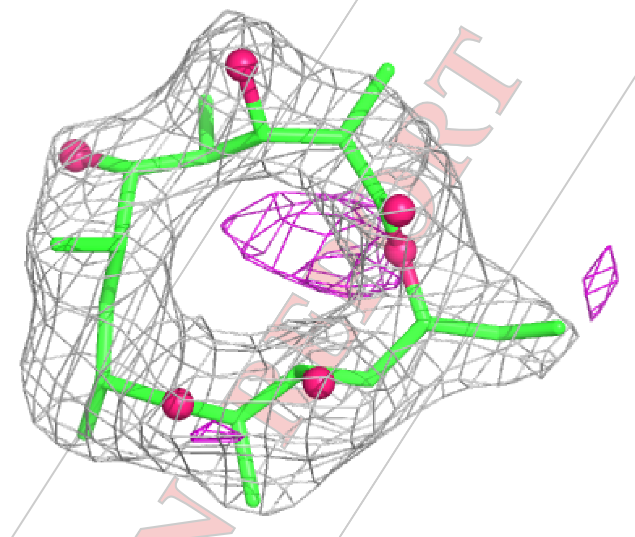

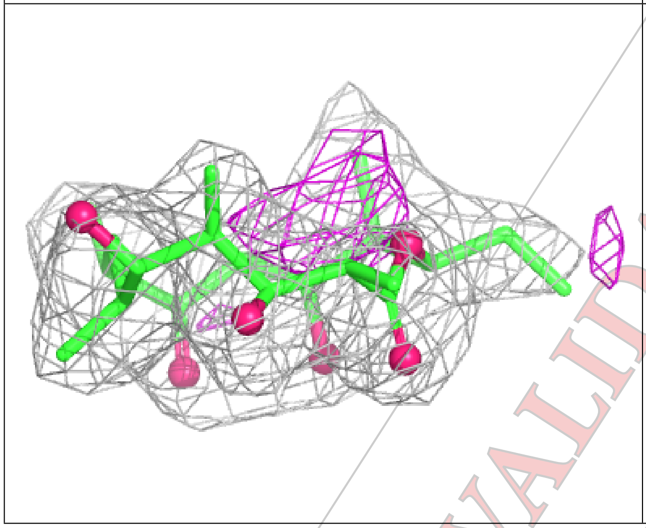

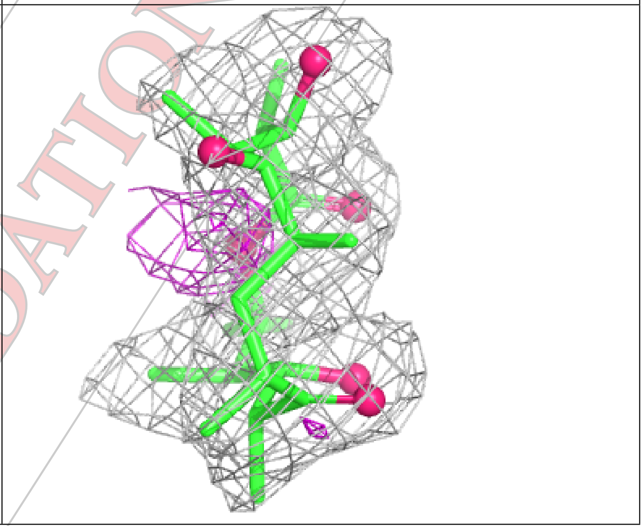

PRELIMINARY VALIDATION REPORT

Electron density around DEB D 502:

$2mF_o$-DF_c (at 0.7 rmsd) in gray
mF_o-DF_c (at 3 rmsd) in purple (negative)
and green (positive)

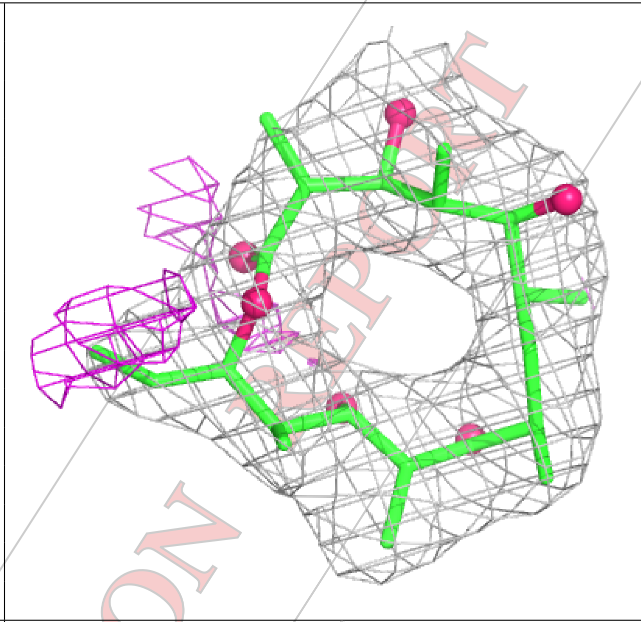

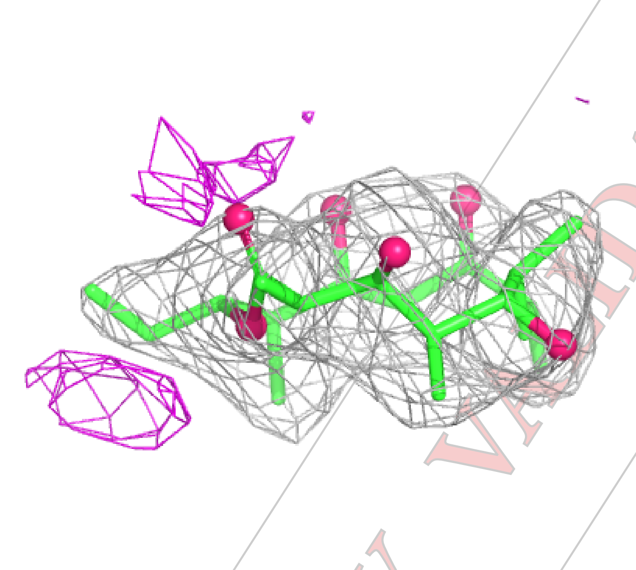

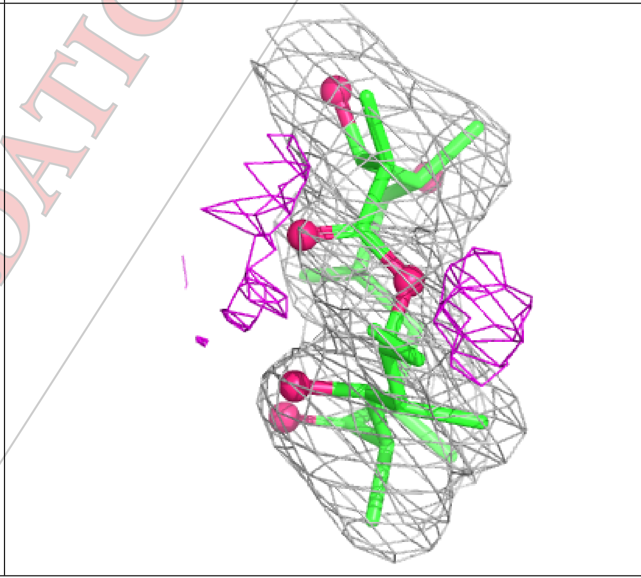

PRELIMINARY VALIDATION REPORT

Electron density around DEB A 502:

$2mF_o$-DF_c (at 0.7 rmsd) in gray
mF_o-DF_c (at 3 rmsd) in purple (negative)
and green (positive)

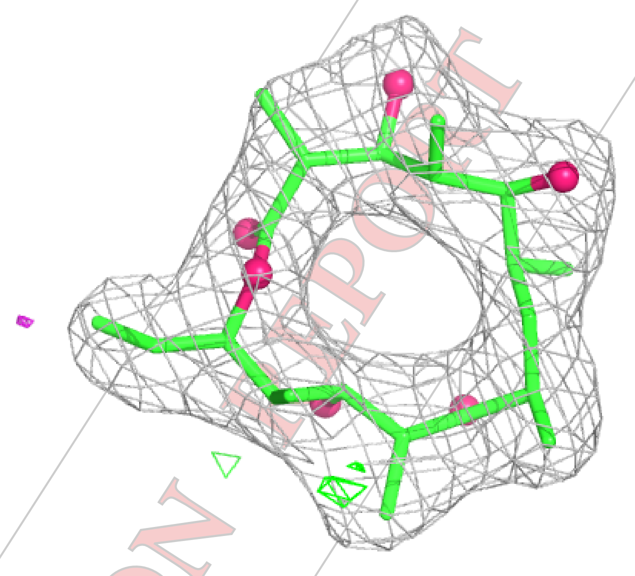

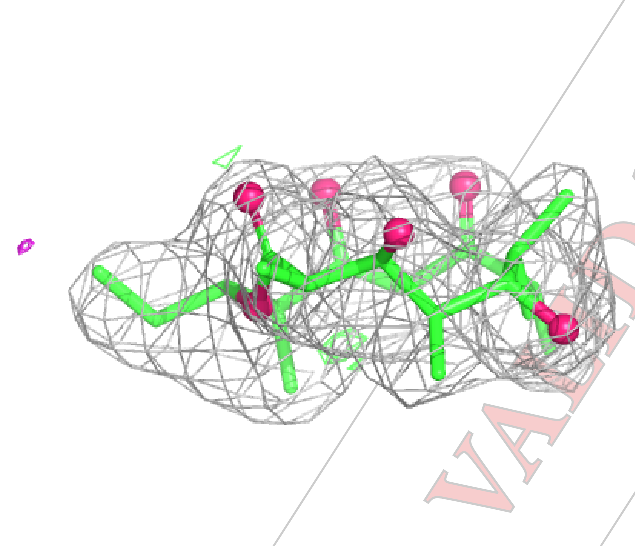

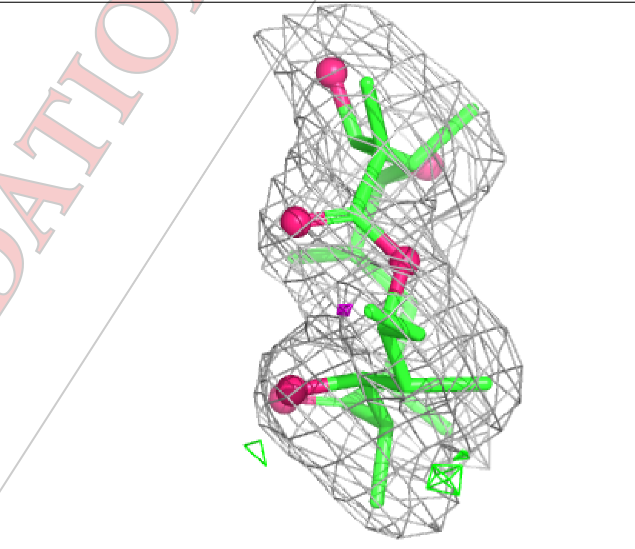

PRELIMINARY VALIDATION REPORT

Electron density around DEB B 502:

2mF$_o$-DF$_c$ (at 0.7 rmsd) in gray
mF$_o$-DF$_c$ (at 3 rmsd) in purple (negative)
and green (positive)

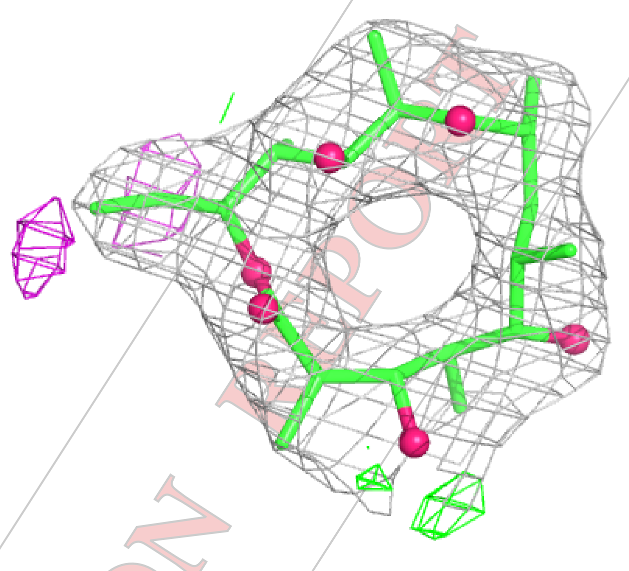

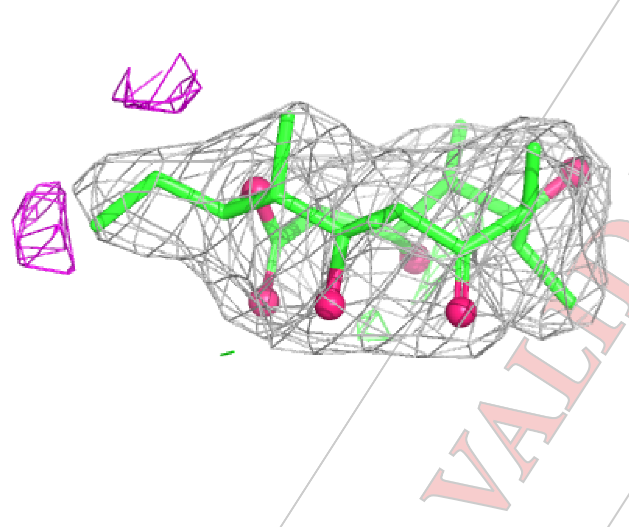

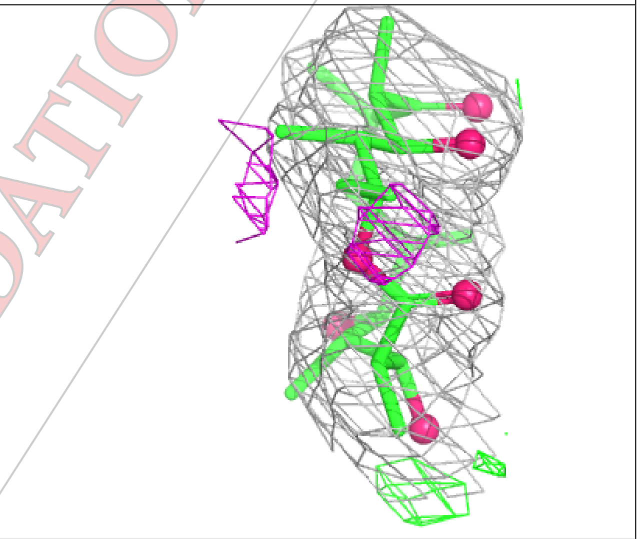

PRELIMINARY VALIDATION REPORT

Electron density around HEM A 501:

2mF$_o$-DF$_c$ (at 0.7 rmsd) in gray
mF$_o$-DF$_c$ (at 3 rmsd) in purple (negative)
and green (positive)

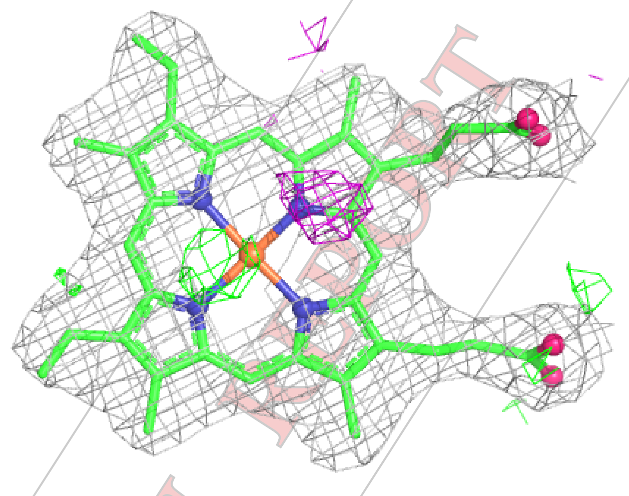

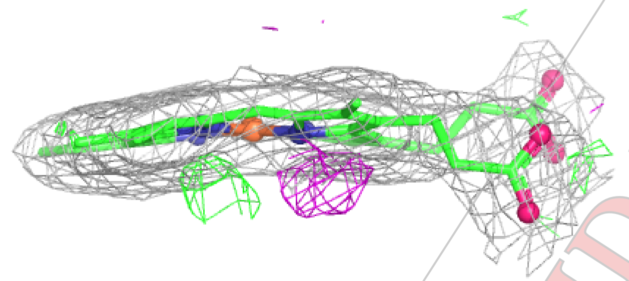

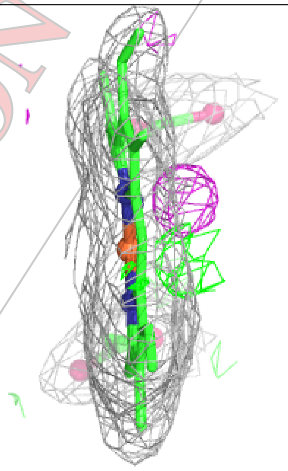

PRELIMINARY VALIDATION REPORT

Electron density around HEM D 501:

$2mF_o$-DF_c (at 0.7 rmsd) in gray
mF_o-DF_c (at 3 rmsd) in purple (negative)
and green (positive)

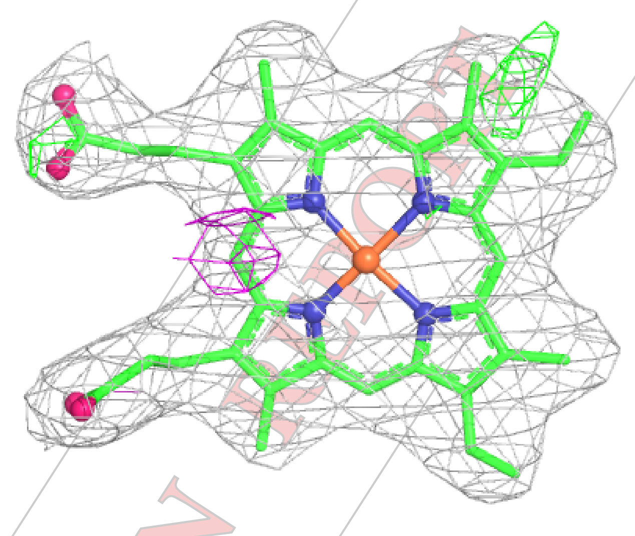

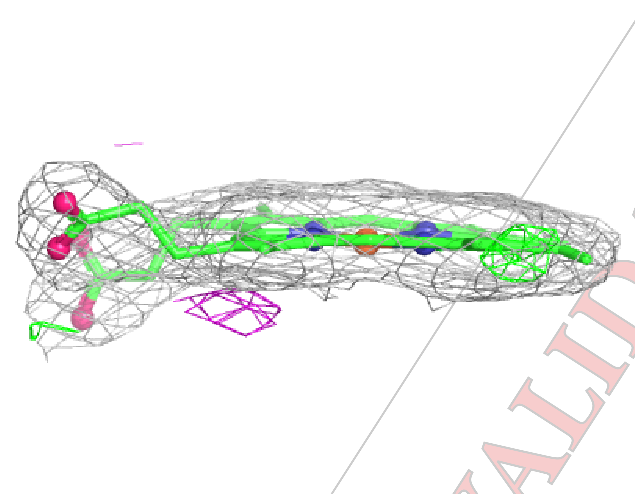

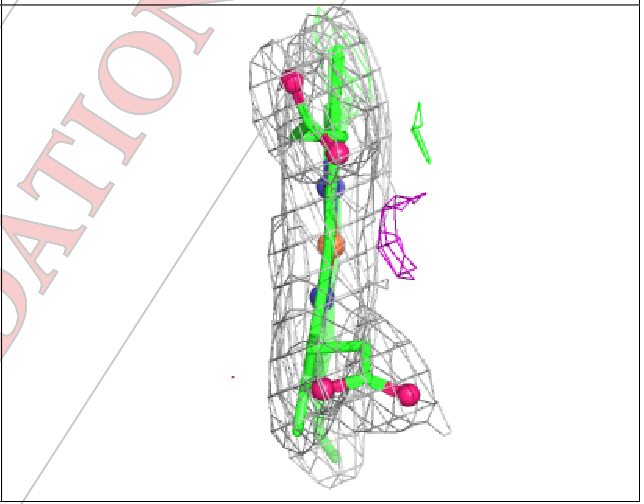

PRELIMINARY VALIDATION REPORT

**Electron density around HEM B 501:**<br><br>2mF$_o$-DF$_c$ (at 0.7 rmsd) in gray<br>mF$_o$-DF$_c$ (at 3 rmsd) in purple (negative)<br>and green (positive)	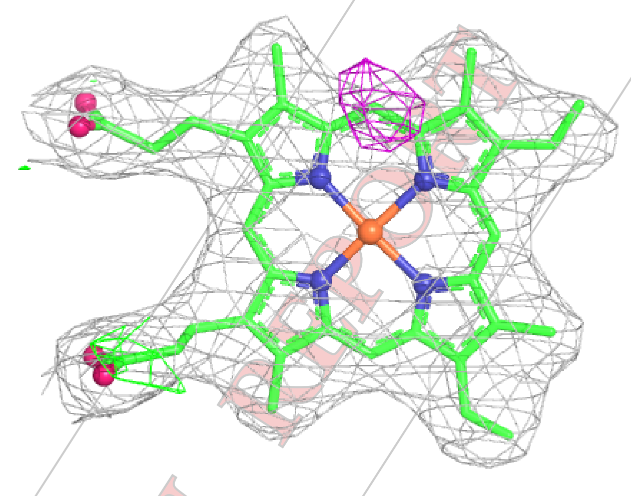
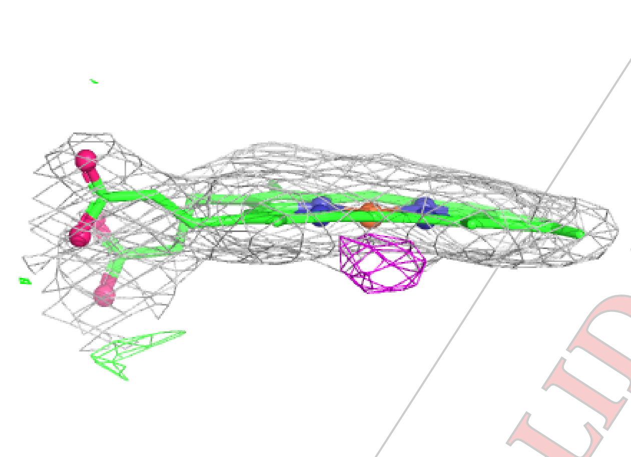	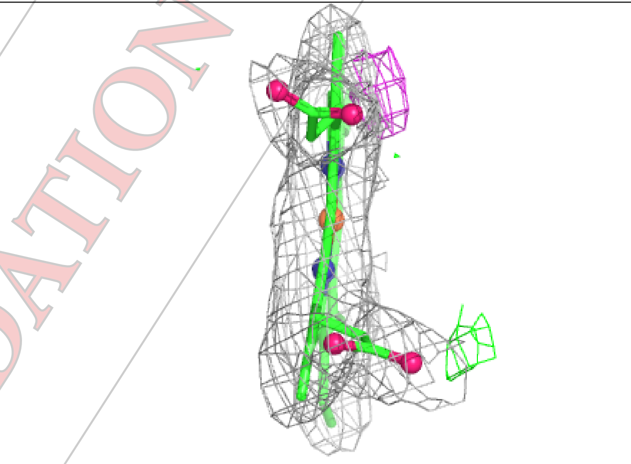

PRELIMINARY VALIDATION REPORT

Electron density around HEM C 501:

$2mF_o$-DF_c (at 0.7 rmsd) in gray
mF_o-DF_c (at 3 rmsd) in purple (negative)
and green (positive)

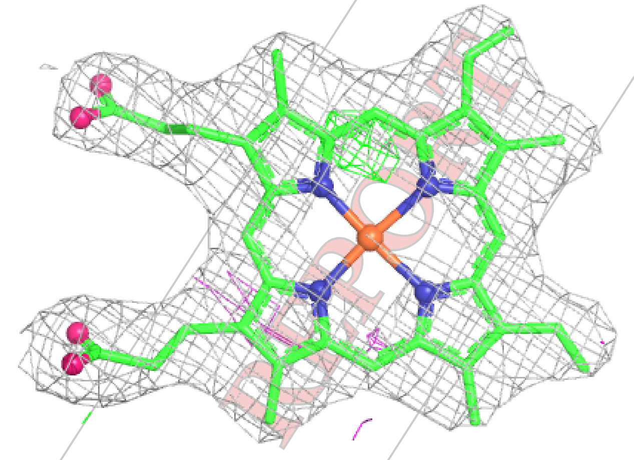

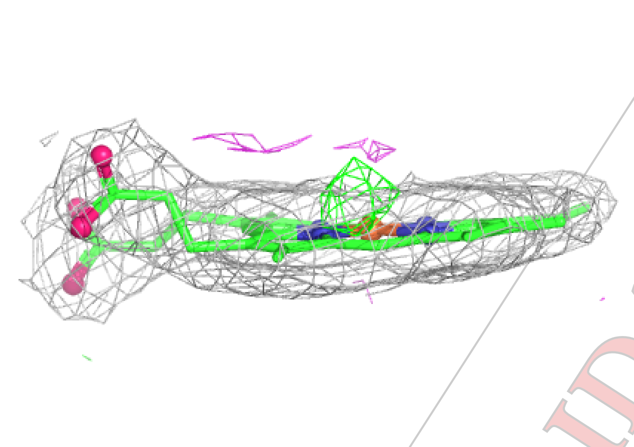

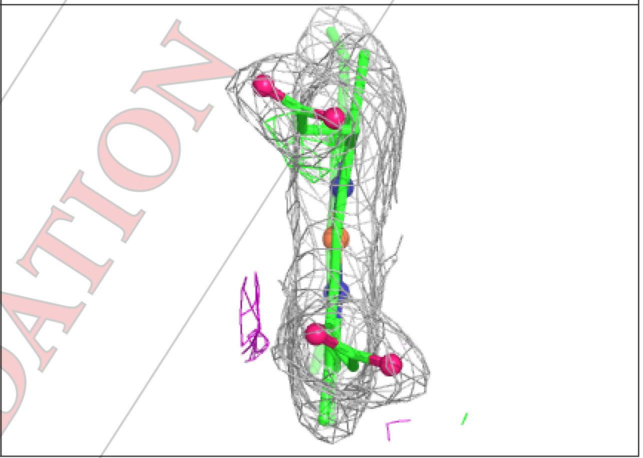

6.5 Other polymers (i)

There are no such residues in this entry.

PRELIMINARY VALIDATION REPORT
