# Supplementary material for: Point Mutations at a Key Site Alter the Cytochrome P450 OleP Structural Dynamics
Source: Biomolecules. 2021 Dec 31;12(1):55. doi: 10.3390/biom12010055 (PMC8774231; doi:10.3390/biom12010055)
Supplement: Supplementary file 1 [file biomolecules-12-00055-s001.zip › Supplementary_Files_Revised/g92w-6deb_D_1292119176_val-report-full_P1.pdf]

Preliminary Full wwPDB X-ray Structure Validation Report (i)

Dec 16, 2021 – 08:46 pm GMT

Deposition ID : D_1292119176

This is a Preliminary Full wwPDB X-ray Structure Validation Report.

This report is produced by the wwPDB Deposition System during initial deposition but before annotation of the structure.

We welcome your comments at *validation@mail.wwpdb.org*
A user guide is available at
https://www.wwpdb.org/validation/2017/XrayValidationReportHelp
with specific help available everywhere you see the (i) symbol.

PRELIMINARY VALIDATION REPORT

The following versions of software and data (see references (i)) were used in the production of this report:

MolProbity	:	4.02b-467
Mogul	:	1.8.4, CSD as541be (2020)
Xtriage (Phenix)	:	1.13
EDS	:	2.24
buster-report	:	1.1.7 (2018)
Percentile statistics	:	20191225.v01 (using entries in the PDB archive December 25th 2019)
Refmac	:	5.8.0267
CCP4	:	7.1.010 (Gargrove)
Ideal geometry (proteins)	:	Engh & Huber (2001)
Ideal geometry (DNA, RNA)	:	Parkinson et al. (1996)
Validation Pipeline (wwPDB-VP)	:	2.24

1 Overall quality at a glance (i)

The following experimental techniques were used to determine the structure:
X-RAY DIFFRACTION

The reported resolution of this entry is 2.08 Å.

Percentile scores (ranging between 0-100) for global validation metrics of the entry are shown in the following graphic. The table shows the number of entries on which the scores are based.

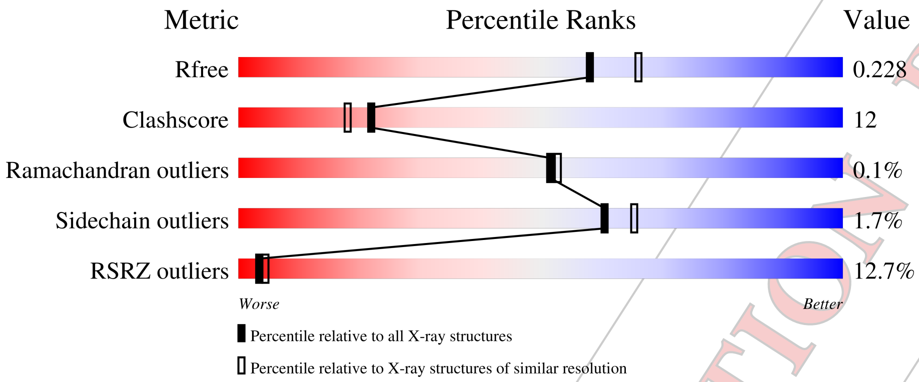

Metric	Whole archive (#Entries)	Similar resolution (#Entries, resolution range(Å))
R_{free}	130704	6189 (2.10-2.06)
Clashscore	141614	6738 (2.10-2.06)
Ramachandran outliers	138981	6663 (2.10-2.06)
Sidechain outliers	138945	6664 (2.10-2.06)
RSRZ outliers	127900	6057 (2.10-2.06)

The table below summarises the geometric issues observed across the polymeric chains and their fit to the electron density. The red, orange, yellow and green segments of the lower bar indicate the fraction of residues that contain outliers for >=3, 2, 1 and 0 types of geometric quality criteria respectively. A grey segment represents the fraction of residues that are not modelled. The numeric value for each fraction is indicated below the corresponding segment, with a dot representing fractions <=5% The upper red bar (where present) indicates the fraction of residues that have poor fit to the electron density. The numeric value is given above the bar.

Mol	Chain	Length	Quality of chain
1	A	396	7%<br>80% 18% •
1	D	396	10%<br>77% 23% •
1	F	396	26%<br>80% 19% •
2	B	402	7%<br>83% 16%
3	C	408	4%<br>88% 12%

Continued on next page...

PRELIMINARY VALIDATION REPORT

Continued from previous page...

Mol	Chain	Length	Quality of chain
4	E	395	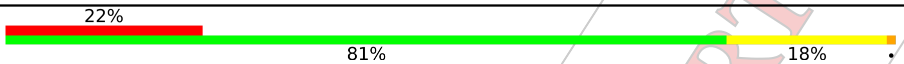

The following table lists non-polymeric compounds, carbohydrate monomers and non-standard residues in protein, DNA, RNA chains that are outliers for geometric or electron-density-fit criteria:

Mol	Type	Chain	Res	Chirality	Geometry	Clashes	Electron density
7	FMT	H	1004	-	-	-	X
7	FMT	H	1005	-	-	-	X
7	FMT	H	1020	-	-	-	X
7	FMT	H	1021	-	-	-	X
7	FMT	H	1024	-	-	X	-
7	FMT	H	1036	-	-	-	X
7	FMT	H	1040	-	-	X	-
7	FMT	H	1060	-	-	X	-
7	FMT	H	1061	-	-	X	-
7	FMT	H	19	-	-	X	-
7	FMT	H	31	-	-	X	-
7	FMT	H	33	-	-	X	-
7	FMT	H	57	-	-	-	X
7	FMT	H	62	-	-	-	X
7	FMT	H	66	-	-	X	-
7	FMT	H	71	-	-	X	-
7	FMT	H	72	-	-	-	X
7	FMT	H	902	-	-	X	-
7	FMT	H	917	-	-	-	X
7	FMT	H	928	-	-	-	X
7	FMT	H	929	-	-	-	X
7	FMT	H	940	-	-	-	X
7	FMT	H	950	-	-	X	-
7	FMT	H	951	-	-	X	-
7	FMT	H	960	-	-	X	-
7	FMT	H	970	-	-	X	-
7	FMT	H	976	-	-	-	X
7	FMT	H	984	-	-	-	X
7	FMT	H	998	-	-	-	X
9	GOL	K	11	-	-	-	X
9	GOL	K	4[A]	-	-	X	-
9	GOL	K	5	-	-	-	X
9	GOL	K	7[A]	-	-	X	-
9	GOL	K	7[B]	-	-	X	-

PRELIMINARY VALIDATION REPORT

2 Entry composition (i)

There are 10 unique types of molecules in this entry. The entry contains 21704 atoms, of which 0 are hydrogens and 0 are deuteriums.

In the tables below, the ZeroOcc column contains the number of atoms modelled with zero occupancy, the AltConf column contains the number of residues with at least one atom in alternate conformation and the Trace column contains the number of residues modelled with at most 2 atoms.

- Molecule 1 is a protein called Cytochrome P-450.

Mol	Chain	Residues	Atoms					ZeroOcc	AltConf	Trace
1	A	396	Total 3298	C 2092	N 595	O 596	S 15	0	32	0
1	D	396	Total 3186	C 2018	N 564	O 589	S 15	0	16	0
1	F	396	Total 3132	C 1976	N 556	O 584	S 16	0	6	0

There are 6 discrepancies between the modelled and reference sequences:

Chain	Residue	Modelled	Actual	Comment	Reference
A	0	HIS	-	expression tag	UNP Q59819
A	92	TRP	GLY	engineered mutation	UNP Q59819
D	0	HIS	-	expression tag	UNP Q59819
D	92	TRP	GLY	engineered mutation	UNP Q59819
F	0	HIS	-	expression tag	UNP Q59819
F	92	TRP	GLY	engineered mutation	UNP Q59819

- Molecule 2 is a protein called Cytochrome P-450.

Mol	Chain	Residues	Atoms					ZeroOcc	AltConf	Trace
2	B	402	Total 3331	C 2113	N 597	O 606	S 15	0	33	0

There are 2 discrepancies between the modelled and reference sequences:

Chain	Residue	Modelled	Actual	Comment	Reference
B	0	HIS	-	expression tag	UNP Q59819
B	92	TRP	GLY	engineered mutation	UNP Q59819

- Molecule 3 is a protein called Cytochrome P-450.

PRELIMINARY VALIDATION REPORT

Mol	Chain	Residues	Atoms					ZeroOcc	AltConf	Trace
3	C	408	Total<br>3354	C<br>2128	N<br>598	O<br>611	S<br>17	0	29	0

There are 2 discrepancies between the modelled and reference sequences:

Chain	Residue	Modelled	Actual	Comment	Reference
C	0	HIS	-	expression tag	UNP Q59819
C	92	TRP	GLY	engineered mutation	UNP Q59819

- Molecule 4 is a protein called Cytochrome P-450.

Mol	Chain	Residues	Atoms					ZeroOcc	AltConf	Trace
4	E	395	Total<br>3141	C<br>1985	N<br>558	O<br>580	S<br>18	0	9	0

There are 2 discrepancies between the modelled and reference sequences:

Chain	Residue	Modelled	Actual	Comment	Reference
E	0	HIS	-	expression tag	UNP Q59819
E	92	TRP	GLY	engineered mutation	UNP Q59819

- Molecule 5 is PROTOPORPHYRIN IX CONTAINING FE (three-letter code: HEM) (formula: $C_{34}H_{32}FeN_4O_4$).

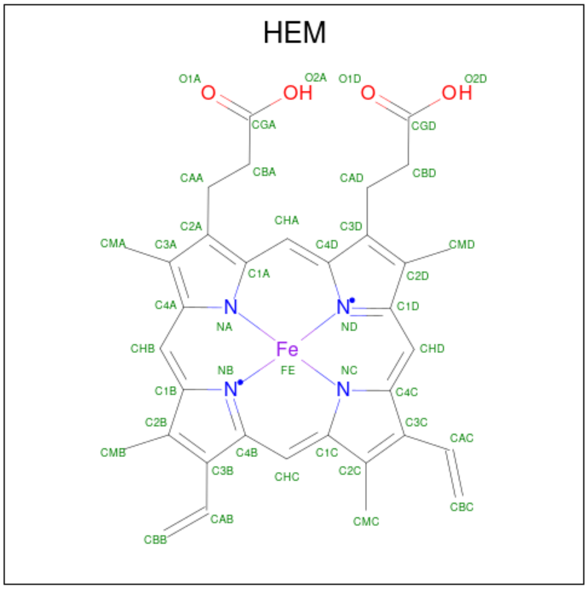

Mol	Chain	Residues	Atoms					ZeroOcc	AltConf
5	A	1	Total<br>43	C<br>34	Fe<br>1	N<br>4	O<br>4	0	0

Continued on next page...

Continued from previous page...

Mol	Chain	Residues	Atoms					ZeroOcc	AltConf
5	B	1	Total 43	C 34	Fe 1	N 4	O 4	0	0
5	C	1	Total 43	C 34	Fe 1	N 4	O 4	0	0
5	D	1	Total 43	C 34	Fe 1	N 4	O 4	0	0
5	E	1	Total 43	C 34	Fe 1	N 4	O 4	0	0
5	F	1	Total 43	C 34	Fe 1	N 4	O 4	0	0

- Molecule 6 is 6-DEOXYERYTHRONOLIDE B (three-letter code: DEB) (formula: $C_{21}H_{38}O_6$).

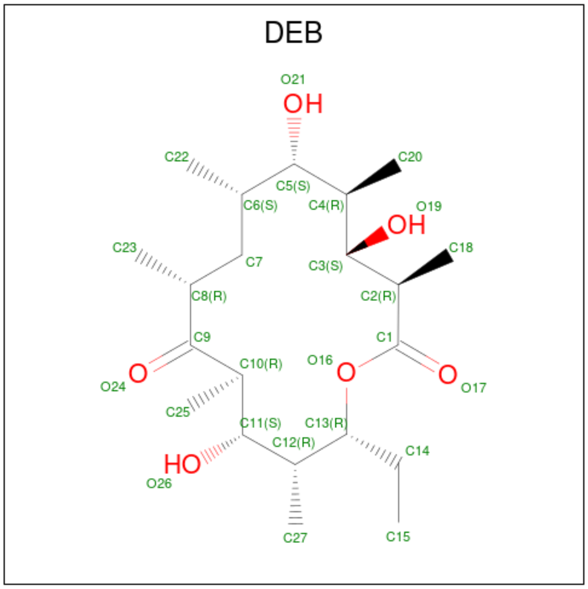

Mol	Chain	Residues	Atoms			ZeroOcc	AltConf
6	A	1	Total 27	C 21	O 6	0	0
6	B	1	Total 27	C 21	O 6	0	0
6	D	1	Total 27	C 21	O 6	0	0
6	E	1	Total 27	C 21	O 6	0	0
6	F	1	Total 27	C 21	O 6	0	0
6	Z	1	Total 27	C 21	O 6	0	0

PRELIMINARY VALIDATION REPORT

- Molecule 7 is FORMIC ACID (three-letter code: FMT) (formula: CH_2O_2).

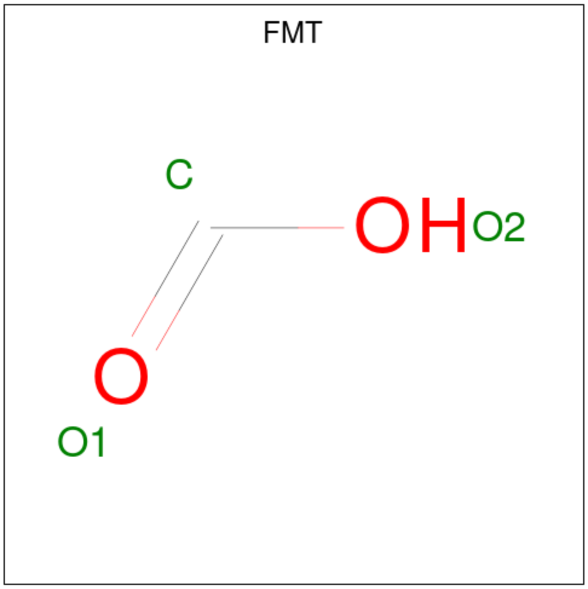

Mol	Chain	Residues	Atoms	ZeroOcc	AltConf
7	H	1	Total C O<br>3 1 2	0	0
7	H	1	Total C O<br>3 1 2	0	0
7	H	1	Total C O<br>3 1 2	0	0
7	H	1	Total C O<br>3 1 2	0	0
7	H	1	Total C O<br>3 1 2	0	0
7	H	1	Total C O<br>3 1 2	0	0
7	H	1	Total C O<br>3 1 2	0	0
7	H	1	Total C O<br>3 1 2	0	0
7	H	1	Total C O<br>3 1 2	0	0
7	H	1	Total C O<br>3 1 2	0	0
7	H	1	Total C O<br>3 1 2	0	0
7	H	1	Total C O<br>3 1 2	0	0
7	H	1	Total C O<br>3 1 2	0	0

Continued on next page...

PRELIMINARY VALIDATION REPORT

Continued from previous page...

Mol	Chain	Residues	Atoms	ZeroOcc	AltConf
7	H	1	Total C O<br>3 1 2	0	0
7	H	1	Total C O<br>3 1 2	0	0
7	H	1	Total C O<br>3 1 2	0	0
7	H	1	Total C O<br>3 1 2	0	0
7	H	1	Total C O<br>3 1 2	0	0
7	H	1	Total C O<br>3 1 2	0	0
7	H	1	Total C O<br>3 1 2	0	0
7	H	1	Total C O<br>3 1 2	0	0
7	H	1	Total C O<br>3 1 2	0	0
7	H	1	Total C O<br>3 1 2	0	0
7	H	1	Total C O<br>3 1 2	0	0
7	H	1	Total C O<br>3 1 2	0	0
7	H	1	Total C O<br>3 1 2	0	0
7	H	1	Total C O<br>3 1 2	0	0
7	H	1	Total C O<br>3 1 2	0	0
7	H	1	Total C O<br>3 1 2	0	0
7	H	1	Total C O<br>3 1 2	0	0
7	H	1	Total C O<br>3 1 2	0	0
7	H	1	Total C O<br>3 1 2	0	0
7	H	1	Total C O<br>3 1 2	0	0
7	H	1	Total C O<br>3 1 2	0	0

Continued on next page...

PRELIMINARY VALIDATION REPORT

Continued from previous page...

Mol	Chain	Residues	Atoms	ZeroOcc	AltConf
7	H	1	Total C O<br>3 1 2	0	0
7	H	1	Total C O<br>3 1 2	0	0
7	H	1	Total C O<br>3 1 2	0	0
7	H	1	Total C O<br>3 1 2	0	0
7	H	1	Total C O<br>3 1 2	0	0
7	H	1	Total C O<br>3 1 2	0	0
7	H	1	Total C O<br>3 1 2	0	0
7	H	1	Total C O<br>3 1 2	0	0
7	H	1	Total C O<br>3 1 2	0	0
7	H	1	Total C O<br>3 1 2	0	0
7	H	1	Total C O<br>3 1 2	0	0
7	H	1	Total C O<br>3 1 2	0	0
7	H	1	Total C O<br>3 1 2	0	0
7	H	1	Total C O<br>3 1 2	0	0
7	H	1	Total C O<br>3 1 2	0	0
7	H	1	Total C O<br>3 1 2	0	0
7	H	1	Total C O<br>3 1 2	0	0
7	H	1	Total C O<br>3 1 2	0	0
7	H	1	Total C O<br>3 1 2	0	0
7	H	1	Total C O<br>3 1 2	0	0
7	H	1	Total C O<br>3 1 2	0	0

Continued on next page...

PRELIMINARY VALIDATION REPORT

Continued from previous page...

Mol	Chain	Residues	Atoms	ZeroOcc	AltConf
7	H	1	Total C O<br>3 1 2	0	0
7	H	1	Total C O<br>3 1 2	0	0
7	H	1	Total C O<br>3 1 2	0	0
7	H	1	Total C O<br>3 1 2	0	0
7	H	1	Total C O<br>3 1 2	0	0
7	H	1	Total C O<br>3 1 2	0	0
7	H	1	Total C O<br>3 1 2	0	0
7	H	1	Total C O<br>3 1 2	0	0
7	H	1	Total C O<br>3 1 2	0	0
7	H	1	Total C O<br>3 1 2	0	0
7	H	1	Total C O<br>3 1 2	0	0
7	H	1	Total C O<br>3 1 2	0	0
7	H	1	Total C O<br>3 1 2	0	0
7	H	1	Total C O<br>3 1 2	0	0
7	H	1	Total C O<br>3 1 2	0	0
7	H	1	Total C O<br>3 1 2	0	0
7	H	1	Total C O<br>3 1 2	0	0
7	H	1	Total C O<br>3 1 2	0	0
7	H	1	Total C O<br>3 1 2	0	0
7	H	1	Total C O<br>3 1 2	0	0
7	H	1	Total C O<br>3 1 2	0	0

Continued on next page...

PRELIMINARY VALIDATION REPORT

Continued from previous page...

Mol	Chain	Residues	Atoms	ZeroOcc	AltConf
7	H	1	Total C O<br>3 1 2	0	0
7	H	1	Total C O<br>3 1 2	0	0
7	H	1	Total C O<br>3 1 2	0	0
7	H	1	Total C O<br>3 1 2	0	0
7	H	1	Total C O<br>3 1 2	0	0
7	H	1	Total C O<br>3 1 2	0	0
7	H	1	Total C O<br>3 1 2	0	0
7	H	1	Total C O<br>3 1 2	0	0
7	H	1	Total C O<br>3 1 2	0	0
7	H	1	Total C O<br>3 1 2	0	0
7	H	1	Total C O<br>3 1 2	0	0
7	H	1	Total C O<br>3 1 2	0	0
7	H	1	Total C O<br>3 1 2	0	0
7	H	1	Total C O<br>3 1 2	0	0
7	H	1	Total C O<br>3 1 2	0	0
7	H	1	Total C O<br>3 1 2	0	0
7	H	1	Total C O<br>3 1 2	0	0
7	H	1	Total C O<br>3 1 2	0	0
7	H	1	Total C O<br>3 1 2	0	0
7	H	1	Total C O<br>3 1 2	0	0
7	H	1	Total C O<br>3 1 2	0	0

Continued on next page...

PRELIMINARY VALIDATION REPORT

Continued from previous page...

Mol	Chain	Residues	Atoms	ZeroOcc	AltConf
7	H	1	Total C O<br>3 1 2	0	0
7	H	1	Total C O<br>3 1 2	0	0
7	H	1	Total C O<br>3 1 2	0	0
7	H	1	Total C O<br>3 1 2	0	0
7	H	1	Total C O<br>3 1 2	0	0
7	H	1	Total C O<br>3 1 2	0	0
7	H	1	Total C O<br>3 1 2	0	0
7	H	1	Total C O<br>3 1 2	0	0
7	H	1	Total C O<br>3 1 2	0	0
7	H	1	Total C O<br>3 1 2	0	0
7	H	1	Total C O<br>3 1 2	0	0
7	H	1	Total C O<br>3 1 2	0	0
7	H	1	Total C O<br>3 1 2	0	0
7	H	1	Total C O<br>3 1 2	0	0
7	H	1	Total C O<br>3 1 2	0	0
7	H	1	Total C O<br>3 1 2	0	0
7	H	1	Total C O<br>3 1 2	0	0
7	H	1	Total C O<br>3 1 2	0	0
7	H	1	Total C O<br>3 1 2	0	0
7	H	1	Total C O<br>3 1 2	0	0
7	H	1	Total C O<br>3 1 2	0	0

Continued on next page...

PRELIMINARY VALIDATION REPORT

Continued from previous page...

Mol	Chain	Residues	Atoms	ZeroOcc	AltConf
7	H	1	Total C O<br>3 1 2	0	0
7	H	1	Total C O<br>3 1 2	0	0
7	H	1	Total C O<br>3 1 2	0	0
7	H	1	Total C O<br>3 1 2	0	0
7	H	1	Total C O<br>3 1 2	0	0
7	H	1	Total C O<br>3 1 2	0	0
7	H	1	Total C O<br>3 1 2	0	0
7	H	1	Total C O<br>3 1 2	0	0
7	H	1	Total C O<br>3 1 2	0	0
7	H	1	Total C O<br>3 1 2	0	0
7	H	1	Total C O<br>3 1 2	0	0
7	H	1	Total C O<br>3 1 2	0	0
7	H	1	Total C O<br>3 1 2	0	0
7	H	1	Total C O<br>3 1 2	0	0
7	H	1	Total C O<br>3 1 2	0	0
7	H	1	Total C O<br>3 1 2	0	0
7	H	1	Total C O<br>3 1 2	0	0
7	H	1	Total C O<br>3 1 2	0	0
7	H	1	Total C O<br>3 1 2	0	0
7	H	1	Total C O<br>3 1 2	0	0
7	H	1	Total C O<br>3 1 2	0	0

PRELIMINARY VALIDATION REPORT

- Molecule 8 is SODIUM ION (three-letter code: NA) (formula: Na).

Mol	Chain	Residues	Atoms	ZeroOcc	AltConf
8	I	1	Total Na<br>1 1	0	0
8	I	1	Total Na<br>1 1	0	0
8	I	1	Total Na<br>1 1	0	0
8	I	1	Total Na<br>1 1	0	0
8	I	1	Total Na<br>1 1	0	0
8	I	1	Total Na<br>1 1	0	0
8	I	1	Total Na<br>1 1	0	0
8	I	1	Total Na<br>1 1	0	0

- Molecule 9 is GLYCEROL (three-letter code: GOL) (formula: $C_3H_8O_3$).

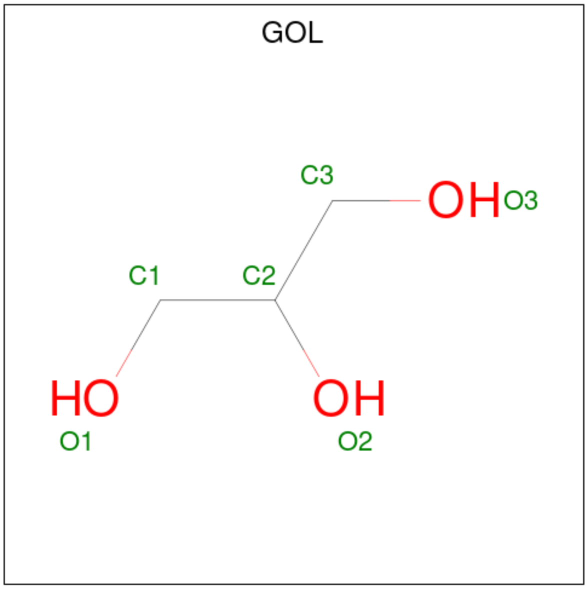

Mol	Chain	Residues	Atoms	ZeroOcc	AltConf
9	K	1	Total C O<br>12 6 6	0	1
9	K	1	Total C O<br>6 3 3	0	0
9	K	1	Total C O<br>12 6 6	0	1

Continued on next page...

PRELIMINARY VALIDATION REPORT

Continued from previous page...

Mol	Chain	Residues	Atoms	ZeroOcc	AltConf
9	K	1	Total C O<br>6 3 3	0	0
9	K	1	Total C O<br>12 6 6	0	1
9	K	1	Total C O<br>12 6 6	0	1
9	K	1	Total C O<br>6 3 3	0	0
9	K	1	Total C O<br>6 3 3	0	0
9	K	1	Total C O<br>6 3 3	0	0

- Molecule 10 is water.

Mol	Chain	Residues	Atoms	ZeroOcc	AltConf
10	G	1339	Total O<br>1339 1339	0	0

PRELIMINARY VALIDATION REPORT

3 Residue-property plots (i)

These plots are drawn for all protein, RNA, DNA and oligosaccharide chains in the entry. The first graphic for a chain summarises the proportions of the various outlier classes displayed in the second graphic. The second graphic shows the sequence view annotated by issues in geometry and electron density. Residues are color-coded according to the number of geometric quality criteria for which they contain at least one outlier: green = 0, yellow = 1, orange = 2 and red = 3 or more. A red dot above a residue indicates a poor fit to the electron density (RSRZ > 2). Stretches of 2 or more consecutive residues without any outlier are shown as a green connector. Residues present in the sample, but not in the model, are shown in grey.

- Molecule 1: Cytochrome P-450

Chain A:
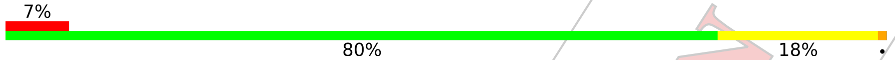

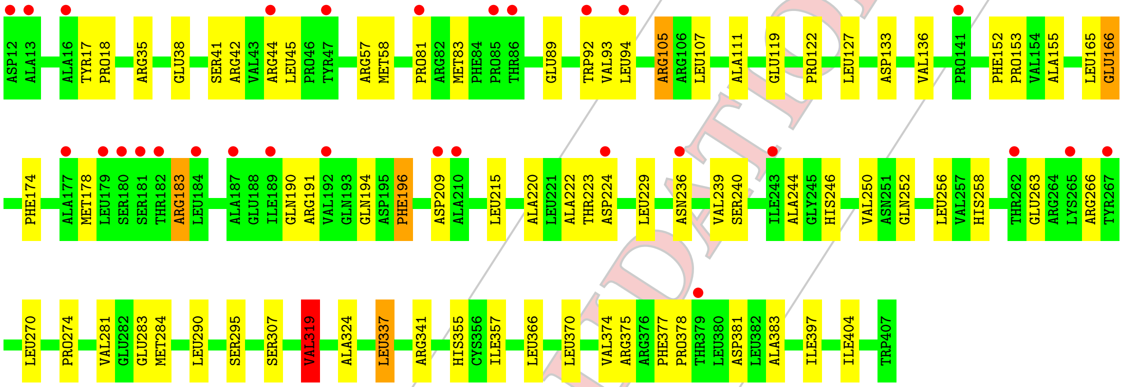

- Molecule 1: Cytochrome P-450

Chain D:
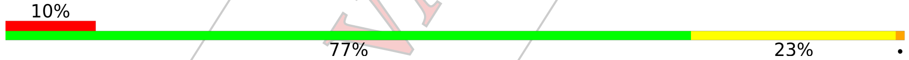

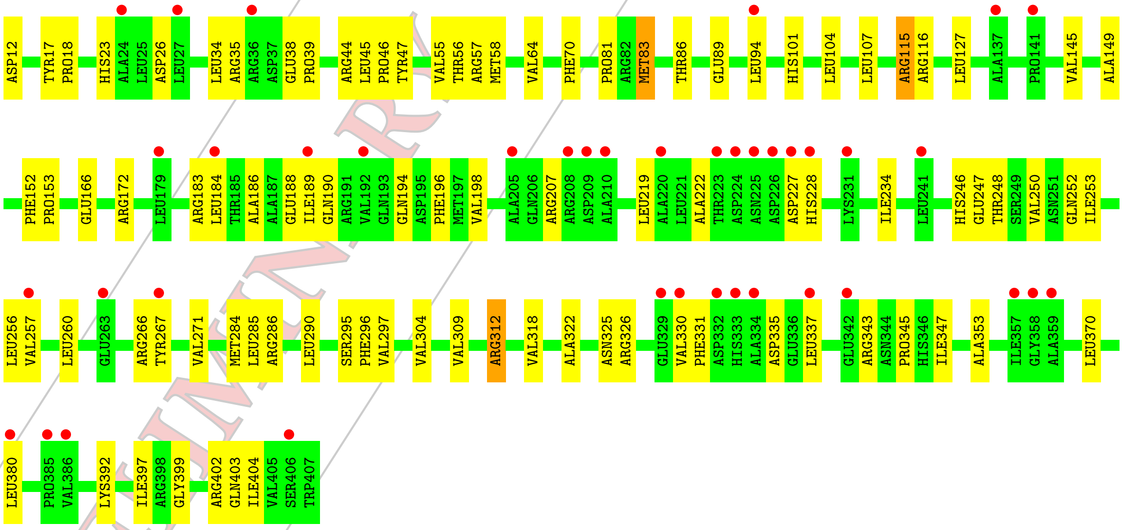

- Molecule 1: Cytochrome P-450

Chain F:
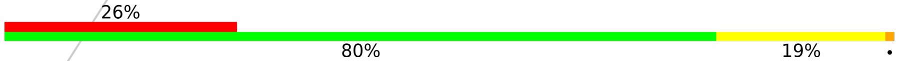

PRELIMINARY VALIDATION REPORT

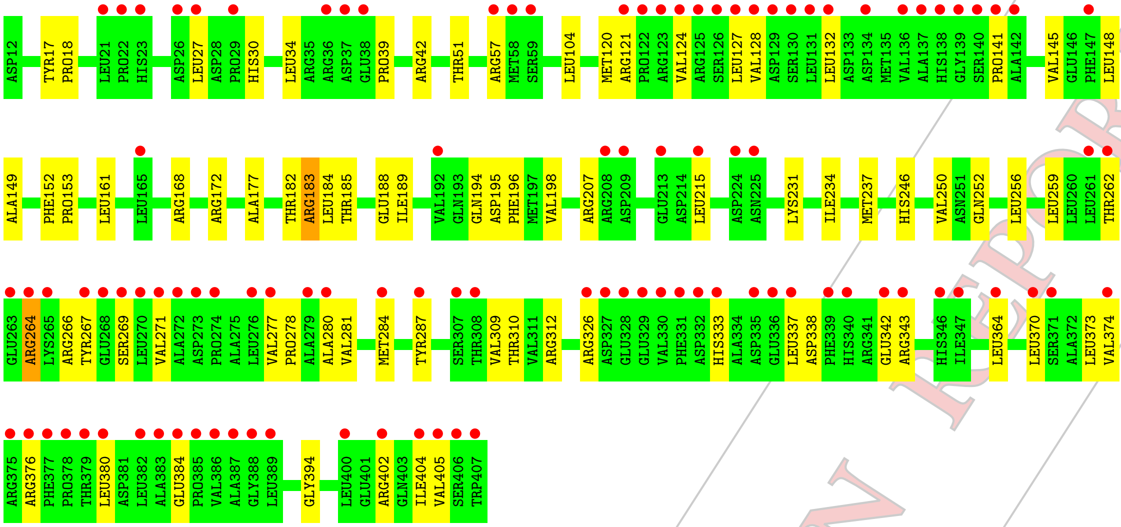

• Molecule 2: Cytochrome P-450

Chain B:

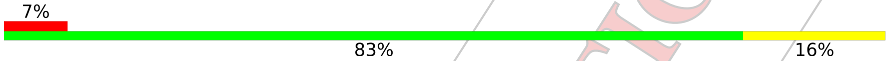

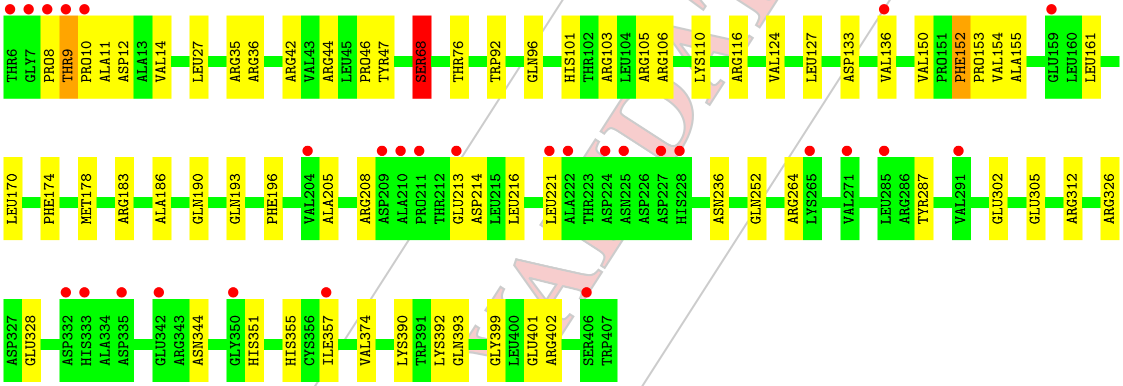

• Molecule 3: Cytochrome P-450

Chain C:

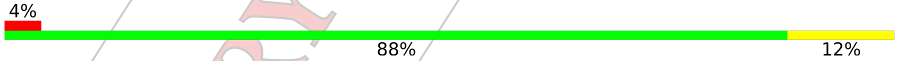

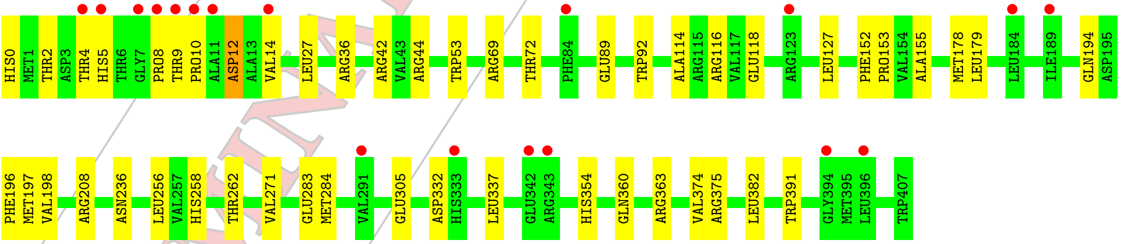

• Molecule 4: Cytochrome P-450

Chain E:

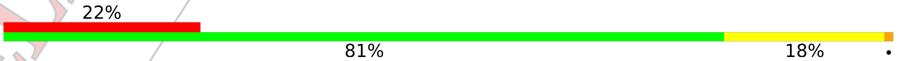

PRELIMINARY VALIDATION REPORT

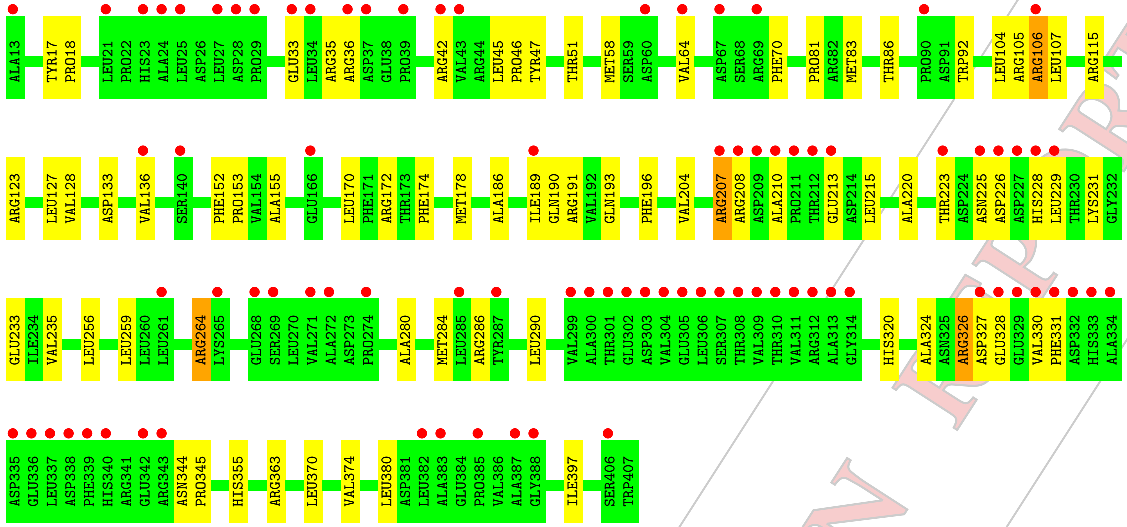

PRELIMINARY VALIDATION REPORT

4 Data and refinement statistics (i)

Property	Value	Source
Space group	C 1 2 1	Depositor
Cell constants<br>a, b, c, α, β, γ	247.76Å 110.49Å 159.82Å<br>90.00° 129.66° 90.00°	Depositor
Resolution (Å)	48.03 – 2.08<br>47.98 – 1.96	Depositor<br>EDS
% Data completeness<br>(in resolution range)	99.8 (48.03-2.08)<br>99.7 (47.98-1.96)	Depositor<br>EDS
R_{merge}	0.08	Depositor
R_{sym}	(Not available)	Depositor
$< I/\sigma(I) >$ [1]	1.36 (at 1.97Å)	Xtriage
Refinement program	REFMAC 5.8.0267	Depositor
R, R_{free}	0.170 , 0.228<br>0.181 , 0.228	Depositor<br>DCC
R_{free} test set	11825 reflections (5.00%)	wwPDB-VP
Wilson B-factor (Å^2)	50.9	Xtriage
Anisotropy	0.025	Xtriage
Bulk solvent k_{sol}(e/Å^3), B_{sol}(Å^2)	(Not available) , (Not available)	EDS
L-test for twinning[2]	$< \|L\| > = 0.50$, $< L^2 > = 0.33$	Xtriage
Estimated twinning fraction	0.010 for -h-2*l,-k,l	Xtriage
F_o,F_c correlation	0.97	EDS
Total number of atoms	21704	wwPDB-VP
Average B, all atoms (Å^2)	59.0	wwPDB-VP

Xtriage's analysis on translational NCS is as follows: *The analyses of the Patterson function reveals a significant off-origin peak that is 37.94 % of the origin peak, indicating pseudo-translational symmetry. The chance of finding a peak of this or larger height randomly in a structure without pseudo-translational symmetry is equal to 3.9742e-04. The detected translational NCS is most likely also responsible for the elevated intensity ratio.*

[1] Intensities estimated from amplitudes.
[2] Theoretical values of $< |L| >$, $< L^2 >$ for acentric reflections are 0.5, 0.333 respectively for untwinned datasets, and 0.375, 0.2 for perfectly twinned datasets.

PRELIMINARY VALIDATION REPORT

5 Model quality (i)

5.1 Standard geometry (i)

Bond lengths and bond angles in the following residue types are not validated in this section: DEB, GOL, FMT, HEM, NA

The Z score for a bond length (or angle) is the number of standard deviations the observed value is removed from the expected value. A bond length (or angle) with $|Z| > 5$ is considered an outlier worth inspection. RMSZ is the root-mean-square of all Z scores of the bond lengths (or angles).

Mol	Chain	Bond lengths		Bond angles	
		RMSZ	#|Z| >5	RMSZ	#|Z| >5
1	A	0.80	0/3456	0.98	9/4701 (0.2%)
1	D	0.82	0/3303	0.99	3/4499 (0.1%)
1	F	0.74	0/3213	0.90	0/4378
2	B	0.80	0/3491	0.94	2/4750 (0.0%)
3	C	0.80	0/3516	0.94	1/4785 (0.0%)
4	E	0.75	0/3231	0.90	2/4400 (0.0%)
All	All	0.79	0/20210	0.94	17/27513 (0.1%)

Chiral center outliers are detected by calculating the chiral volume of a chiral center and verifying if the center is modelled as a planar moiety or with the opposite hand.A planarity outlier is detected by checking planarity of atoms in a peptide group, atoms in a mainchain group or atoms of a sidechain that are expected to be planar.

Mol	Chain	#Chirality outliers	#Planarity outliers
1	A	0	3
2	B	0	1
All	All	0	4

There are no bond length outliers.

All (17) bond angle outliers are listed below:

Mol	Chain	Res	Type	Atoms	Z	Observed(^{o})	Ideal(^{o})
1	A	319[A]	VAL	CA-C-O	-8.17	102.95	120.10
1	A	319[B]	VAL	CA-C-O	-8.17	102.95	120.10
2	B	68	SER	N-CA-C	7.91	132.36	111.00
1	A	319[A]	VAL	CA-C-N	6.10	130.62	117.20
1	A	319[B]	VAL	CA-C-N	6.10	130.62	117.20
4	E	207	ARG	NE-CZ-NH1	-5.94	117.33	120.30
1	A	105[A]	ARG	NE-CZ-NH2	-5.77	117.41	120.30
1	A	105[B]	ARG	NE-CZ-NH2	-5.77	117.41	120.30

Continued on next page...

PRELIMINARY VALIDATION REPORT

Continued from previous page...

Mol	Chain	Res	Type	Atoms	Z	Observed(^{o})	Ideal(^{o})
1	A	105[A]	ARG	NE-CZ-NH1	5.58	123.09	120.30
1	A	105[B]	ARG	NE-CZ-NH1	5.58	123.09	120.30
1	D	83[A]	MET	CA-C-O	5.34	131.31	120.10
1	D	83[B]	MET	CA-C-O	5.34	131.31	120.10
1	D	172	ARG	NE-CZ-NH2	-5.33	117.64	120.30
2	B	152	PHE	CB-CA-C	5.28	120.96	110.40
3	C	363	ARG	NE-CZ-NH1	5.23	122.91	120.30
1	A	209	ASP	CB-CA-C	5.12	120.64	110.40
4	E	363	ARG	NE-CZ-NH2	-5.08	117.76	120.30

There are no chirality outliers.

All (4) planarity outliers are listed below:

Mol	Chain	Res	Type	Group
1	A	319[A]	VAL	Mainchain
1	A	319[B]	VAL	Mainchain
1	A	42	ARG	Sidechain
2	B	68	SER	Mainchain

5.2 Too-close contacts (i)

In the following table, the Non-H and H(model) columns list the number of non-hydrogen atoms and hydrogen atoms in the chain respectively. The H(added) column lists the number of hydrogen atoms added and optimized by MolProbity. The Clashes column lists the number of clashes within the asymmetric unit, whereas Symm-Clashes lists symmetry-related clashes.

Mol	Chain	Non-H	H(model)	H(added)	Clashes	Symm-Clashes
1	A	3298	0	3371	98	0
1	D	3186	0	3212	88	0
1	F	3132	0	3113	68	0
2	B	3331	0	3408	94	1
3	C	3354	0	3421	59	1
4	E	3141	0	3143	70	0
5	A	43	0	30	3	0
5	B	43	0	30	1	0
5	C	43	0	30	0	0
5	D	43	0	30	4	0
5	E	43	0	30	2	0
5	F	43	0	30	3	0
6	A	27	0	38	8	0
6	B	27	0	38	2	0

Continued on next page...

PRELIMINARY VALIDATION REPORT

Continued from previous page...

Mol	Chain	Non-H	H(model)	H(added)	Clashes	Symm-Clashes
6	D	27	0	38	0	0
6	E	27	0	38	0	0
6	F	27	0	38	0	0
6	Z	27	0	38	6	0
7	H	417	0	139	47	1
8	I	8	0	0	0	0
9	K	78	0	101	31	0
10	G	1339	0	0	23	1
All	All	21704	0	20316	481	2

The all-atom clashscore is defined as the number of clashes found per 1000 atoms (including hydrogen atoms). The all-atom clashscore for this structure is 12.

All (481) close contacts within the same asymmetric unit are listed below, sorted by their clash magnitude.

Atom-1	Atom-2	Interatomic distance (Å)	Clash overlap (Å)
2:B:236[A]:ASN:OD1	9:K:7[A]:GOL:H2	1.22	1.24
2:B:236[A]:ASN:OD1	9:K:7[A]:GOL:C2	2.04	1.05
1:D:115[A]:ARG:HH11	1:D:115[A]:ARG:CG	1.70	1.04
1:A:92[B]:TRP:HH2	6:A:502:DEB:H223	1.25	1.02
2:B:92:TRP:HA	2:B:236[A]:ASN:ND2	1.74	1.01
3:C:89[B]:GLU:OE2	3:C:92[B]:TRP:CG	2.16	0.98
2:B:161:LEU:O	2:B:216[B]:LEU:HD13	1.65	0.96
2:B:92:TRP:HA	2:B:236[A]:ASN:HD22	1.27	0.96
2:B:236[A]:ASN:CG	9:K:7[A]:GOL:H2	1.86	0.95
1:A:92[B]:TRP:HZ3	1:A:240:SER:HG	0.98	0.92
4:E:106[A]:ARG:HH21	4:E:106[A]:ARG:HG3	1.34	0.91
1:F:266:ARG:NE	1:F:337:LEU:HD12	1.85	0.91
1:A:122:PRO:HB3	1:D:309:VAL:HG22	1.53	0.90
4:E:45:LEU:HD12	4:E:81:PRO:HB2	1.52	0.90
1:A:92[B]:TRP:CH2	6:A:502:DEB:H223	2.07	0.89
4:E:226:ASP:OD2	4:E:229:LEU:HB2	1.71	0.89
2:B:9[A]:THR:HG23	2:B:10[A]:PRO:CD	2.02	0.89
1:A:35[B]:ARG:HE	9:K:13:GOL:H32	1.36	0.88
1:D:267:TYR:CE1	1:D:380:LEU:HD23	2.09	0.88
3:C:89[B]:GLU:OE2	3:C:92[B]:TRP:CD2	2.27	0.87
1:A:92[B]:TRP:CZ2	1:A:94:LEU:HD12	2.10	0.87
2:B:161:LEU:HB3	2:B:216[B]:LEU:CD1	2.05	0.86
1:D:17:TYR:HD2	1:D:83[B]:MET:HE1	1.40	0.85
1:A:370:LEU:O	1:A:374:VAL:HG23	1.75	0.85
2:B:9[A]:THR:HG23	2:B:10[A]:PRO:HD2	1.57	0.84

Continued on next page...

PRELIMINARY VALIDATION REPORT

Continued from previous page...

Atom-1	Atom-2	Interatomic distance (Å)	Clash overlap (Å)
1:A:92[B]:TRP:HZ3	1:A:240:SER:OG	1.60	0.84
4:E:106[A]:ARG:HH21	4:E:106[A]:ARG:CG	1.90	0.84
1:D:115[A]:ARG:HH11	1:D:115[A]:ARG:HG2	1.42	0.83
1:F:185:THR:OG1	1:F:188:GLU:HG3	1.77	0.83
1:D:115[A]:ARG:HH11	1:D:115[A]:ARG:HG3	1.42	0.82
3:C:92[B]:TRP:HH2	3:C:178:MET:HE1	1.44	0.82
2:B:161:LEU:HB3	2:B:216[B]:LEU:HD11	1.61	0.82
1:F:266:ARG:CZ	1:F:337:LEU:HD12	2.09	0.81
4:E:280:ALA:O	4:E:284[A]:MET:HG3	1.81	0.81
1:F:277:VAL:HG23	1:F:278:PRO:HD3	1.63	0.81
1:D:257:VAL:HG23	1:D:403:GLN:HE22	1.46	0.81
1:F:161:LEU:HD23	1:F:215:LEU:HD12	1.63	0.80
1:A:45:LEU:HD12	1:A:81:PRO:HB2	1.62	0.80
1:A:92[B]:TRP:HZ2	6:A:502:DEB:HO9	1.30	0.79
1:F:128:VAL:O	1:F:132:LEU:HG	1.82	0.79
1:F:195:ASP:O	1:F:198:VAL:HG22	1.81	0.79
3:C:179[B]:LEU:HD11	6:Z:502:DEB:H201	1.64	0.79
2:B:10[B]:PRO:HD3	3:C:360[B]:GLN:OE1	1.83	0.78
4:E:229:LEU:HA	4:E:233[B]:GLU:OE1	1.84	0.77
6:A:502:DEB:H5	9:K:8[A]:GOL:H2	1.66	0.77
1:D:115[A]:ARG:HG3	1:D:115[A]:ARG:NH1	1.98	0.77
1:A:45:LEU:HD12	1:A:81:PRO:CB	2.14	0.76
4:E:45:LEU:HD12	4:E:81:PRO:CB	2.15	0.76
4:E:51:THR:HG22	7:H:976:FMT:O2	1.85	0.76
3:C:92[B]:TRP:CH2	10:G:1347:HOH:O	2.37	0.75
4:E:133:ASP:O	4:E:136:VAL:HG22	1.86	0.75
1:F:333:HIS:HE1	1:F:338:ASP:OD2	1.69	0.75
2:B:105[A]:ARG:HD3	2:B:357:ILE:HD12	1.69	0.75
1:F:256:LEU:HD23	1:F:284[B]:MET:HE1	1.69	0.75
4:E:256:LEU:HD22	4:E:284[B]:MET:HB3	1.67	0.74
9:K:1[A]:GOL:O3	9:K:1[A]:GOL:O1	2.02	0.74
4:E:370:LEU:O	4:E:374:VAL:HG23	1.87	0.74
1:D:166[B]:GLU:HG3	4:E:228:HIS:NE2	2.04	0.73
1:F:51:THR:O	7:H:938:FMT:O1	2.07	0.73
4:E:106[A]:ARG:HG3	4:E:106[A]:ARG:NH2	1.96	0.72
1:D:297:VAL:HG22	1:D:318:VAL:HG22	1.72	0.72
2:B:10[B]:PRO:CD	3:C:360[B]:GLN:OE1	2.37	0.72
3:C:92[B]:TRP:CZ2	10:G:1347:HOH:O	2.42	0.72
1:A:92[B]:TRP:HZ2	1:A:94:LEU:HD12	1.52	0.71
2:B:178[B]:MET:SD	2:B:193:GLN:HG2	2.30	0.71
4:E:178[A]:MET:CE	9:K:4[A]:GOL:H32	2.21	0.71

Continued on next page...

PRELIMINARY VALIDATION REPORT

Continued from previous page...

Atom-1	Atom-2	Interatomic distance (Å)	Clash overlap (Å)
2:B:161:LEU:C	2:B:216[B]:LEU:HD13	2.10	0.70
1:A:119:GLU:OE2	7:H:966:FMT:O2	2.09	0.70
2:B:351:HIS:HD1	7:H:33:FMT:H	1.56	0.70
4:E:207:ARG:HA	4:E:210:ALA:HB3	1.74	0.70
1:A:92[B]:TRP:CE3	9:K:8[B]:GOL:H2	2.27	0.70
3:C:256:LEU:HD22	3:C:284:MET:HB3	1.74	0.70
4:E:178[A]:MET:HE1	9:K:4[A]:GOL:H32	1.71	0.70
3:C:92[B]:TRP:HH2	3:C:178:MET:CE	2.04	0.70
6:B:502:DEB:O19	9:K:7[B]:GOL:H31	1.93	0.69
1:D:86[A]:THR:HG21	1:D:189:ILE:CG2	2.23	0.69
1:D:115[A]:ARG:CG	1:D:115[A]:ARG:NH1	2.41	0.69
1:A:58[B]:MET:SD	1:A:324:ALA:HB1	2.33	0.69
1:A:92[B]:TRP:CH2	6:A:502:DEB:H5	2.28	0.69
3:C:89[B]:GLU:CD	3:C:92[B]:TRP:CE3	2.67	0.69
2:B:9[A]:THR:HG23	2:B:10[A]:PRO:HD3	1.73	0.68
1:D:326:ARG:HD3	10:G:254:HOH:O	1.93	0.68
2:B:105[A]:ARG:NH2	2:B:355:HIS:O	2.26	0.68
5:D:501:HEM:HBB2	5:D:501:HEM:HMB2	1.76	0.68
1:A:127[A]:LEU:HD21	1:A:155:ALA:HB3	1.76	0.67
2:B:236[A]:ASN:OD1	9:K:7[A]:GOL:C1	2.42	0.67
1:D:45:LEU:HD12	1:D:81:PRO:HB2	1.76	0.67
1:F:271:VAL:HG22	1:F:374:VAL:HG13	1.77	0.67
1:A:105[A]:ARG:NH2	1:A:355:HIS:O	2.27	0.67
1:A:92[B]:TRP:HH2	6:A:502:DEB:C22	2.05	0.66
1:A:92[B]:TRP:HE3	1:A:92[B]:TRP:HA	1.59	0.66
5:E:501:HEM:HHC	5:E:501:HEM:HBB2	1.75	0.66
1:F:39:PRO:HB3	1:F:57:ARG:HD2	1.78	0.65
2:B:208:ARG:NH2	10:G:559:HOH:O	2.30	0.65
1:D:17:TYR:CD2	1:D:83[B]:MET:HE1	2.28	0.65
1:A:122:PRO:CB	1:D:309:VAL:HG22	2.26	0.65
1:D:12:ASP:CG	1:D:44:ARG:HH21	2.00	0.64
1:F:277:VAL:CG2	1:F:278:PRO:HD3	2.27	0.64
1:F:287:TYR:CG	1:F:337:LEU:HD21	2.31	0.64
3:C:179[B]:LEU:HD11	6:Z:502:DEB:C20	2.27	0.64
5:A:501:HEM:HBB2	5:A:501:HEM:HMB2	1.79	0.64
1:A:92[B]:TRP:CE3	1:A:92[B]:TRP:HA	2.33	0.64
1:A:283:GLU:HG3	1:A:337:LEU:CD2	2.27	0.63
4:E:64:VAL:HG12	4:E:70:PHE:CZ	2.33	0.63
1:A:107:LEU:HD11	1:A:222:ALA:CB	2.29	0.63
1:A:281:VAL:CG2	1:A:370:LEU:HD12	2.29	0.63
1:D:35[B]:ARG:HG3	1:D:57[B]:ARG:HG2	1.81	0.63

Continued on next page...

PRELIMINARY VALIDATION REPORT

Continued from previous page...

Atom-1	Atom-2	Interatomic distance (Å)	Clash overlap (Å)
1:D:45:LEU:HD11	1:D:318:VAL:HG21	1.79	0.63
1:A:92[B]:TRP:CZ3	9:K:8[B]:GOL:H2	2.35	0.62
1:A:283:GLU:HG3	1:A:337:LEU:HD22	1.80	0.62
4:E:115:ARG:HB3	7:H:66:FMT:H	1.82	0.62
1:D:184:LEU:HB2	1:D:189:ILE:HD11	1.81	0.62
4:E:327:ASP:HB3	4:E:330:VAL:HG12	1.80	0.62
1:D:116:ARG:HB3	1:D:116:ARG:NH1	2.14	0.61
1:D:257:VAL:HG23	1:D:403:GLN:NE2	2.14	0.61
1:A:174:PHE:O	1:A:178[B]:MET:HG2	2.01	0.61
1:F:184:LEU:HB2	1:F:189:ILE:HD11	1.82	0.61
2:B:328[A]:GLU:H	7:H:19:FMT:C	2.14	0.61
2:B:328[B]:GLU:H	7:H:19:FMT:C	2.14	0.61
1:A:256:LEU:HD22	1:A:284:MET:HB3	1.81	0.61
4:E:264:ARG:NH2	4:E:380:LEU:O	2.33	0.61
2:B:96:GLN:OE1	7:H:1009:FMT:H	2.00	0.61
4:E:213:GLU:N	4:E:213:GLU:OE1	2.34	0.61
1:A:236[B]:ASN:OD1	7:H:1053:FMT:H	2.01	0.61
2:B:236[A]:ASN:ND2	9:K:7[A]:GOL:H2	2.16	0.60
1:D:186:ALA:O	1:D:190:GLN:HG3	2.01	0.60
3:C:391:TRP:HB2	7:H:1040:FMT:H	1.81	0.60
2:B:9[A]:THR:CG2	2:B:10[A]:PRO:CD	2.78	0.60
1:A:194[B]:GLN:NE2	10:G:701:HOH:O	2.35	0.60
1:A:256:LEU:HD12	1:A:370:LEU:HD11	1.84	0.60
4:E:152:PHE:HB3	4:E:153:PRO:HD3	1.84	0.60
1:A:355:HIS:NE2	7:H:951:FMT:O1	2.35	0.60
2:B:150:VAL:O	2:B:154[A]:VAL:HG12	2.01	0.60
1:D:252[A]:GLN:HE21	1:D:285:LEU:HD23	1.66	0.60
2:B:76:THR:OG1	7:H:20:FMT:O2	2.20	0.59
3:C:89[B]:GLU:CD	3:C:92[B]:TRP:CD2	2.76	0.59
1:D:256:LEU:HD12	1:D:370[A]:LEU:HD11	1.84	0.59
4:E:104:LEU:HD12	4:E:107:LEU:HD12	1.83	0.59
3:C:14:VAL:HG23	3:C:44[A]:ARG:CG	2.32	0.59
1:D:45:LEU:HD22	1:D:83[B]:MET:CE	2.33	0.59
1:F:280:ALA:O	1:F:284[A]:MET:HG3	2.02	0.59
1:F:145:VAL:HA	1:F:149:ALA:HB3	1.85	0.59
1:A:92[B]:TRP:CZ3	1:A:93:VAL:HG12	2.37	0.59
1:F:182:THR:CG2	1:F:394:GLY:HA2	2.33	0.59
3:C:0:HIS:HD2	3:C:2:THR:OG1	1.86	0.59
1:F:182:THR:HG22	1:F:394:GLY:HA2	1.84	0.59
3:C:89[B]:GLU:OE2	3:C:92[B]:TRP:CB	2.51	0.58
1:D:35[B]:ARG:CG	1:D:57[B]:ARG:HG2	2.33	0.58

Continued on next page...

PRELIMINARY VALIDATION REPORT

Continued from previous page...

Atom-1	Atom-2	Interatomic distance (Å)	Clash overlap (Å)
1:D:86[A]:THR:HG21	1:D:189:ILE:HG21	1.85	0.58
1:D:46:PRO:HB2	1:D:47:TYR:CD1	2.39	0.58
1:D:183:ARG:HB3	1:D:184:LEU:HD12	1.84	0.58
4:E:213:GLU:O	4:E:213:GLU:HG2	2.03	0.58
1:D:107:LEU:HD11	1:D:234:ILE:HG12	1.84	0.58
1:D:330:VAL:HG23	1:D:331:PHE:CD2	2.39	0.58
4:E:256:LEU:HD22	4:E:284[A]:MET:HB3	1.84	0.58
2:B:9[B]:THR:O	3:C:114:ALA:HB1	2.03	0.58
2:B:9[A]:THR:CG2	2:B:10[A]:PRO:HD3	2.34	0.57
1:A:111:ALA:CB	1:A:215[A]:LEU:HD21	2.35	0.57
1:A:107:LEU:HD13	1:A:229:LEU:CD1	2.35	0.57
1:A:111:ALA:HB2	1:A:215[A]:LEU:HD21	1.85	0.57
1:A:281:VAL:HG22	1:A:370:LEU:HD12	1.85	0.57
2:B:103:ARG:HH12	7:H:1061:FMT:H	1.69	0.57
1:A:57[A]:ARG:NH1	1:A:307:SER:CB	2.68	0.57
3:C:14:VAL:HG23	3:C:44[A]:ARG:HG2	1.85	0.57
1:F:30:HIS:O	1:F:34:LEU:HG	2.05	0.57
1:D:152:PHE:HB3	1:D:153:PRO:HD3	1.87	0.56
1:F:195:ASP:O	1:F:198:VAL:CG2	2.52	0.56
4:E:178[A]:MET:CE	9:K:4[A]:GOL:C3	2.84	0.56
4:E:191:ARG:HG2	7:H:71:FMT:H	1.86	0.56
1:F:342:GLU:O	1:F:343:ARG:HG3	2.06	0.56
1:D:246:HIS:O	1:D:250:VAL:HG23	2.06	0.56
1:D:17:TYR:HD2	1:D:83[B]:MET:CE	2.17	0.56
1:A:92[B]:TRP:CZ3	1:A:240:SER:OG	2.46	0.56
3:C:89[B]:GLU:OE2	3:C:92[B]:TRP:HB2	2.05	0.56
3:C:9:THR:N	3:C:10:PRO:CD	2.68	0.55
1:F:287:TYR:CD2	1:F:337:LEU:HD23	2.40	0.55
4:E:326:ARG:NH1	10:G:268:HOH:O	2.38	0.55
1:F:370:LEU:O	1:F:374:VAL:HG23	2.06	0.55
1:D:35[A]:ARG:HD3	1:D:56:THR:O	2.05	0.55
5:A:501:HEM:HBB2	5:A:501:HEM:CMB	2.37	0.55
1:F:287:TYR:CD2	1:F:337:LEU:CD2	2.90	0.55
1:A:57[A]:ARG:NH1	1:A:307:SER:OG	2.40	0.55
3:C:14:VAL:CG2	3:C:44[A]:ARG:HG2	2.36	0.55
1:F:184:LEU:CB	1:F:189:ILE:HD11	2.36	0.55
1:A:105[A]:ARG:CD	1:A:357[A]:ILE:HD13	2.37	0.54
3:C:92[B]:TRP:HZ2	6:Z:502:DEB:O19	1.90	0.54
1:D:295:SER:OG	1:D:296:PHE:O	2.20	0.54
1:A:35[B]:ARG:HE	9:K:13:GOL:C3	2.15	0.54
2:B:236[A]:ASN:OD1	9:K:7[A]:GOL:H11	2.07	0.54

Continued on next page...

PRELIMINARY VALIDATION REPORT

Continued from previous page...

Atom-1	Atom-2	Interatomic distance (Å)	Clash overlap (Å)
4:E:45:LEU:HD22	4:E:83:MET:SD	2.48	0.54
3:C:4[A]:THR:HG22	3:C:5:HIS:O	2.08	0.54
3:C:92[B]:TRP:CH2	3:C:178:MET:CE	2.87	0.54
1:A:105[B]:ARG:HG2	1:A:357[B]:ILE:HD11	1.88	0.54
2:B:92:TRP:CD2	9:K:7[B]:GOL:H32	2.43	0.53
1:D:166[B]:GLU:HG3	4:E:228:HIS:CD2	2.44	0.53
3:C:194:GLN:O	3:C:198:VAL:HG23	2.09	0.53
5:F:501:HEM:HMB2	5:F:501:HEM:HBB2	1.90	0.53
3:C:179[B]:LEU:CD1	6:Z:502:DEB:C20	2.87	0.53
1:D:17:TYR:CD2	1:D:83[B]:MET:CE	2.92	0.53
1:A:58[B]:MET:HG2	1:A:324:ALA:O	2.09	0.53
1:A:92[B]:TRP:CE3	1:A:93:VAL:N	2.77	0.53
2:B:161:LEU:HB3	2:B:216[B]:LEU:HD13	1.90	0.53
1:A:92[B]:TRP:CZ2	6:A:502:DEB:O19	2.48	0.53
5:E:501:HEM:HBC2	5:E:501:HEM:HMC2	1.92	0.53
1:A:57[A]:ARG:HH11	1:A:307:SER:CB	2.22	0.52
1:A:166:GLU:H	1:A:166:GLU:CD	2.11	0.52
1:D:145:VAL:HA	1:D:149:ALA:HB3	1.89	0.52
2:B:101:HIS:CE1	2:B:105[B]:ARG:HD2	2.44	0.52
1:F:185:THR:O	1:F:189:ILE:HG12	2.09	0.52
3:C:89[B]:GLU:CD	3:C:89[B]:GLU:H	2.13	0.52
2:B:127[A]:LEU:HD21	2:B:155:ALA:HB3	1.90	0.52
1:D:330:VAL:HG23	1:D:331:PHE:CE2	2.45	0.52
1:A:45:LEU:HD12	1:A:81:PRO:HB3	1.91	0.52
2:B:105[A]:ARG:CD	2:B:357:ILE:HD12	2.39	0.52
1:F:185:THR:HG1	1:F:188:GLU:HG3	1.74	0.52
1:A:107:LEU:HD11	1:A:222:ALA:HB1	1.91	0.52
2:B:46:PRO:HB2	2:B:47:TYR:CD1	2.44	0.51
1:A:92[B]:TRP:CE3	1:A:92[B]:TRP:CA	2.93	0.51
1:F:266:ARG:O	1:F:269:SER:HB3	2.11	0.51
1:A:92[B]:TRP:CE2	1:A:94:LEU:HB2	2.45	0.51
1:D:17:TYR:CD1	1:D:18:PRO:HA	2.44	0.51
1:D:246:HIS:NE2	1:D:247[B]:GLU:OE2	2.43	0.51
1:F:104:LEU:HB3	1:F:237[A]:MET:CE	2.40	0.51
4:E:35:ARG:NH1	4:E:326:ARG:O	2.44	0.51
3:C:89[B]:GLU:HB2	10:G:205:HOH:O	2.10	0.51
3:C:283:GLU:HG3	3:C:337:LEU:HD22	1.93	0.51
2:B:9[B]:THR:HA	3:C:360[B]:GLN:OE1	2.10	0.51
1:A:45:LEU:HD22	1:A:83:MET:SD	2.51	0.51
2:B:9[A]:THR:CB	2:B:10[A]:PRO:CD	2.89	0.51
1:D:194:GLN:O	1:D:198:VAL:HG13	2.11	0.51

Continued on next page...

PRELIMINARY VALIDATION REPORT

Continued from previous page...

Atom-1	Atom-2	Interatomic distance (Å)	Clash overlap (Å)
1:F:252:GLN:O	1:F:256:LEU:HD13	2.10	0.51
2:B:351:HIS:ND1	7:H:33:FMT:H	2.24	0.51
1:A:105[A]:ARG:HD3	1:A:357[A]:ILE:HD13	1.93	0.51
1:D:104:LEU:O	1:D:107:LEU:HB2	2.10	0.51
1:A:274:PRO:HB2	1:A:375[A]:ARG:NH2	2.26	0.50
2:B:106[A]:ARG:NH1	7:H:1060:FMT:C	2.74	0.50
1:D:256:LEU:HD22	1:D:284:MET:HB3	1.93	0.50
2:B:9[B]:THR:HA	3:C:360[B]:GLN:CD	2.32	0.50
2:B:10[B]:PRO:N	3:C:360[B]:GLN:OE1	2.44	0.50
4:E:178[B]:MET:SD	4:E:193:GLN:CG	3.00	0.50
2:B:101:HIS:CE1	2:B:105[B]:ARG:CD	2.95	0.50
1:D:253:ILE:O	1:D:257:VAL:HG22	2.11	0.50
1:F:267:TYR:OH	1:F:373:LEU:O	2.29	0.50
1:D:312:ARG:HD2	10:G:968:HOH:O	2.11	0.50
4:E:172:ARG:NH1	10:G:809:HOH:O	2.38	0.50
2:B:213:GLU:O	2:B:214[A]:ASP:CG	2.50	0.50
2:B:236[A]:ASN:HD21	9:K:7[A]:GOL:C2	2.25	0.50
1:F:264:ARG:NH2	1:F:380:LEU:O	2.41	0.50
2:B:9[A]:THR:OG1	2:B:10[A]:PRO:HD3	2.12	0.49
2:B:393[A]:GLN:NE2	10:G:367:HOH:O	2.44	0.49
1:F:234:ILE:O	1:F:237[B]:MET:HB2	2.12	0.49
3:C:36:ARG:HD2	10:G:1043:HOH:O	2.11	0.49
4:E:331:PHE:CE1	4:E:345:PRO:HD2	2.47	0.49
1:F:195:ASP:C	1:F:198:VAL:HG22	2.31	0.49
4:E:178[B]:MET:SD	4:E:193:GLN:HG2	2.53	0.49
1:A:122:PRO:HB3	1:D:309:VAL:CG2	2.36	0.49
2:B:355:HIS:HE2	7:H:31:FMT:C	2.25	0.49
1:D:45:LEU:HD22	1:D:83[B]:MET:HE1	1.94	0.49
1:D:116:ARG:NH1	10:G:627:HOH:O	2.44	0.49
4:E:106[A]:ARG:HH21	4:E:106[A]:ARG:CB	2.26	0.49
2:B:14:VAL:HG12	3:C:118:GLU:HG2	1.94	0.49
2:B:183:ARG:HH11	7:H:1018:FMT:C	2.26	0.49
1:A:105[B]:ARG:NH1	1:A:357[B]:ILE:HG13	2.28	0.48
2:B:178[B]:MET:HB3	2:B:178[B]:MET:HE2	1.65	0.48
3:C:197[B]:MET:SD	3:C:236:ASN:ND2	2.86	0.48
4:E:123:ARG:NH2	4:E:127[A]:LEU:HD21	2.28	0.48
1:A:281:VAL:HG22	1:A:370:LEU:CD1	2.42	0.48
1:A:17:TYR:HA	1:A:18:PRO:C	2.33	0.48
7:H:902:FMT:H	10:G:8:HOH:O	2.13	0.48
1:A:38[A]:GLU:HG2	1:A:41:SER:HB3	1.94	0.48
1:A:375[B]:ARG:HH21	1:A:375[B]:ARG:HG3	1.79	0.48

Continued on next page...

PRELIMINARY VALIDATION REPORT

Continued from previous page...

Atom-1	Atom-2	Interatomic distance (Å)	Clash overlap (Å)
2:B:236[A]:ASN:ND2	9:K:7[A]:GOL:C2	2.76	0.48
4:E:191:ARG:HG2	7:H:71:FMT:C	2.44	0.48
1:A:366:LEU:O	1:A:370:LEU:HG	2.13	0.48
2:B:186:ALA:O	2:B:190:GLN:HG3	2.14	0.48
1:D:39:PRO:HG3	1:D:57[A]:ARG:CZ	2.43	0.48
1:D:248:THR:O	1:D:252[B]:GLN:HG2	2.13	0.48
4:E:178[A]:MET:HE2	9:K:4[A]:GOL:H32	1.95	0.48
1:F:121:ARG:HG3	1:F:364:LEU:HD11	1.96	0.48
1:A:270:LEU:HB3	1:A:374:VAL:HG11	1.95	0.48
3:C:92[B]:TRP:CE3	3:C:92[B]:TRP:HA	2.49	0.48
1:D:266:ARG:CZ	1:D:337[A]:LEU:HD12	2.44	0.47
1:D:267:TYR:O	1:D:271:VAL:HG23	2.13	0.47
1:F:127:LEU:HD12	1:F:127:LEU:O	2.14	0.47
1:F:287:TYR:CG	1:F:337:LEU:CD2	2.97	0.47
3:C:354:HIS:NE2	9:K:1[B]:GOL:O3	2.33	0.47
4:E:115:ARG:HB3	7:H:66:FMT:C	2.43	0.47
1:A:290:LEU:O	1:A:397:ILE:HA	2.15	0.47
2:B:103:ARG:O	7:H:1060:FMT:H	2.15	0.47
1:D:38:GLU:OE2	7:H:906:FMT:O2	2.32	0.47
1:D:70:PHE:HE2	1:D:304:VAL:HG11	1.79	0.47
1:D:252[B]:GLN:HG3	5:D:501:HEM:CBB	2.44	0.47
1:D:35[A]:ARG:NH2	7:H:960:FMT:O1	2.47	0.47
4:E:17:TYR:HA	4:E:18:PRO:C	2.34	0.47
2:B:178[B]:MET:SD	2:B:193:GLN:CG	3.03	0.47
2:B:35:ARG:HE	7:H:19:FMT:H	1.79	0.47
3:C:92[B]:TRP:CZ2	6:Z:502:DEB:O19	2.62	0.47
1:D:183:ARG:C	1:D:184:LEU:HD12	2.34	0.47
1:D:86[A]:THR:CG2	1:D:189:ILE:HB	2.45	0.47
3:C:152:PHE:HB3	3:C:153:PRO:HD3	1.96	0.47
4:E:178[A]:MET:HE1	9:K:4[A]:GOL:C3	2.44	0.47
1:F:177:ALA:O	1:F:189:ILE:HD12	2.15	0.46
7:H:1004:FMT:C	9:K:3:GOL:H31	2.45	0.46
1:A:239:VAL:HB	9:K:8[B]:GOL:O1	2.15	0.46
1:A:341[B]:ARG:NH1	1:A:341[B]:ARG:HB3	2.30	0.46
1:D:260:LEU:HD11	1:D:370[B]:LEU:HD21	1.97	0.46
4:E:127[B]:LEU:HD21	4:E:155:ALA:HB3	1.98	0.46
2:B:103:ARG:HH12	7:H:1061:FMT:C	2.29	0.46
2:B:133:ASP:O	2:B:136:VAL:HG22	2.16	0.46
1:A:44[A]:ARG:NH2	7:H:943:FMT:O2	2.43	0.46
1:D:402:ARG:HD3	1:D:404:ILE:HG13	1.98	0.46
2:B:110[B]:LYS:HG3	2:B:116:ARG:NH1	2.30	0.46

Continued on next page...

PRELIMINARY VALIDATION REPORT

Continued from previous page...

Atom-1	Atom-2	Interatomic distance (Å)	Clash overlap (Å)
1:D:46:PRO:HB2	1:D:47:TYR:CE1	2.50	0.46
4:E:213:GLU:O	4:E:213:GLU:CG	2.64	0.46
4:E:320:HIS:HD2	10:G:270:HOH:O	1.99	0.46
1:A:355:HIS:ND1	7:H:950:FMT:C	2.79	0.46
1:F:183:ARG:HB3	1:F:394:GLY:HA3	1.98	0.46
1:F:207:ARG:NH1	10:G:296:HOH:O	2.49	0.46
5:F:501:HEM:HMC2	5:F:501:HEM:HBC2	1.97	0.46
2:B:35:ARG:HE	7:H:19:FMT:C	2.29	0.45
3:C:12:ASP:OD1	3:C:12:ASP:N	2.49	0.45
1:D:55:VAL:HG21	1:D:64:VAL:HG21	1.99	0.45
4:E:170:LEU:HD22	4:E:174:PHE:CZ	2.50	0.45
5:F:501:HEM:HBB2	5:F:501:HEM:CMB	2.45	0.45
7:H:902:FMT:C	10:G:8:HOH:O	2.64	0.45
1:D:34:LEU:O	1:D:38:GLU:C	2.54	0.45
4:E:92:TRP:CG	9:K:4[A]:GOL:H12	2.50	0.45
4:E:178[B]:MET:SD	4:E:193:GLN:HG3	2.56	0.45
1:F:39:PRO:CB	1:F:57:ARG:HD2	2.45	0.45
2:B:42[B]:ARG:H	7:H:970:FMT:C	2.29	0.45
3:C:127:LEU:HD21	3:C:155:ALA:HB3	1.99	0.45
5:D:501:HEM:HAB	5:D:501:HEM:HHC	1.56	0.45
1:F:194:GLN:O	1:F:198:VAL:HG13	2.17	0.45
2:B:42[A]:ARG:H	7:H:970:FMT:C	2.29	0.45
1:D:127:LEU:HD23	1:D:152:PHE:CD1	2.52	0.45
1:F:259:LEU:HB2	1:F:284[A]:MET:CE	2.46	0.45
3:C:72:THR:HG23	9:K:1[B]:GOL:H11	1.99	0.45
4:E:215:LEU:HD23	4:E:215:LEU:HA	1.86	0.45
4:E:186:ALA:O	4:E:190:GLN:HG3	2.17	0.45
1:D:252[B]:GLN:OE1	1:D:252[B]:GLN:HA	2.16	0.45
3:C:92[B]:TRP:HE1	6:Z:502:DEB:HO9	1.64	0.45
4:E:33:GLU:HA	4:E:36:ARG:NH2	2.32	0.45
2:B:312[A]:ARG:HB3	3:C:375:ARG:HH21	1.81	0.45
2:B:344:ASN:N	10:G:1035:HOH:O	2.24	0.45
4:E:178[A]:MET:HE2	9:K:4[A]:GOL:C3	2.46	0.45
1:F:246:HIS:O	1:F:250:VAL:HG23	2.17	0.45
2:B:205:ALA:O	2:B:208:ARG:HG2	2.16	0.44
3:C:283:GLU:HG3	3:C:337:LEU:CD2	2.47	0.44
3:C:305[B]:GLU:HG3	7:H:1024:FMT:O1	2.17	0.44
1:A:174:PHE:HB3	1:A:196:PHE:CD2	2.53	0.44
1:D:35[B]:ARG:HG3	1:D:57[B]:ARG:CG	2.44	0.44
1:F:333:HIS:CE1	1:F:338:ASP:OD2	2.60	0.44
1:A:220:ALA:O	1:A:223:THR:HB	2.18	0.44

Continued on next page...

PRELIMINARY VALIDATION REPORT

Continued from previous page...

Atom-1	Atom-2	Interatomic distance (Å)	Clash overlap (Å)
2:B:92:TRP:HA	2:B:236[A]:ASN:HD21	1.75	0.44
2:B:178[B]:MET:CE	2:B:193:GLN:HE21	2.30	0.44
2:B:312[A]:ARG:HB3	3:C:375:ARG:NH2	2.33	0.44
2:B:305:GLU:HG3	7:H:1008:FMT:H	1.99	0.44
1:D:267:TYR:CZ	1:D:380:LEU:HD23	2.50	0.44
1:D:335:ASP:OD1	7:H:960:FMT:O2	2.36	0.44
1:F:168:ARG:O	1:F:172:ARG:HG3	2.17	0.44
1:A:107:LEU:HD11	1:A:222:ALA:HB2	1.99	0.44
2:B:42[A]:ARG:NH2	7:H:915:FMT:O2	2.51	0.44
1:A:92[B]:TRP:CZ2	6:A:502:DEB:H5	2.53	0.44
1:A:383:ALA:HB3	1:A:404:ILE:HG22	1.99	0.44
2:B:236[A]:ASN:CG	9:K:7[A]:GOL:C2	2.71	0.44
2:B:106[A]:ARG:NH2	10:G:1286:HOH:O	2.50	0.44
2:B:392:LYS:HG2	2:B:401:GLU:HG3	2.00	0.44
1:F:145:VAL:HG21	1:F:402:ARG:HA	1.99	0.44
1:A:246:HIS:O	1:A:250:VAL:HG23	2.17	0.43
3:C:69:ARG:HH12	7:H:1024:FMT:C	2.31	0.43
1:A:133:ASP:HA	1:A:136:VAL:HG22	2.00	0.43
3:C:271:VAL:HA	3:C:374:VAL:CG1	2.49	0.43
4:E:259:LEU:HB2	4:E:284[A]:MET:CE	2.48	0.43
1:F:267:TYR:CE1	1:F:374:VAL:HG22	2.53	0.43
1:F:380:LEU:HG	1:F:405:VAL:CG1	2.48	0.43
1:A:58[B]:MET:SD	1:A:324:ALA:O	2.75	0.43
1:D:58[B]:MET:HE1	1:D:347:ILE:HG21	1.99	0.43
1:F:384:GLU:OE1	1:F:402:ARG:NH1	2.51	0.43
2:B:110[B]:LYS:HD2	2:B:110[B]:LYS:HA	1.63	0.43
1:F:148:LEU:O	1:F:152:PHE:HB3	2.18	0.43
1:A:57[A]:ARG:NH1	1:A:307:SER:HB2	2.34	0.43
2:B:27:LEU:HD12	2:B:326[A]:ARG:CZ	2.49	0.43
2:B:152:PHE:HB3	2:B:153:PRO:HD3	2.01	0.43
6:B:502:DEB:O19	9:K:7[B]:GOL:C3	2.65	0.43
5:D:501:HEM:HBC2	5:D:501:HEM:HMC2	2.00	0.43
4:E:107:LEU:HD11	4:E:229:LEU:HD13	2.00	0.43
1:F:284[B]:MET:HE1	1:F:370:LEU:HD11	2.01	0.43
1:A:263:GLU:HB2	1:A:266:ARG:HD2	2.00	0.43
1:F:27:LEU:HD12	1:F:326:ARG:NH1	2.33	0.43
1:F:141:PRO:HB2	1:F:404:ILE:HG22	1.99	0.43
1:A:152:PHE:HB3	1:A:153:PRO:HD3	2.01	0.43
1:A:377:PHE:N	1:A:378:PRO:HD3	2.34	0.43
3:C:258:HIS:CE1	3:C:262:THR:HG21	2.54	0.43
1:D:343:ARG:HG2	1:D:345:PRO:HD3	1.99	0.43

Continued on next page...

PRELIMINARY VALIDATION REPORT

Continued from previous page...

Atom-1	Atom-2	Interatomic distance (Å)	Clash overlap (Å)
4:E:231:LYS:O	4:E:235:VAL:HG23	2.18	0.43
1:F:266:ARG:HE	1:F:337:LEU:HD12	1.75	0.43
1:A:35[B]:ARG:HH21	9:K:13:GOL:C1	2.32	0.43
4:E:286:ARG:HD3	4:E:344:ASN:OD1	2.19	0.43
1:F:17:TYR:CD1	1:F:18:PRO:HA	2.53	0.43
4:E:58[B]:MET:HA	4:E:324:ALA:HB1	1.99	0.43
2:B:355:HIS:NE2	7:H:31:FMT:O1	2.46	0.43
4:E:106[A]:ARG:HH21	4:E:106[A]:ARG:HB3	1.84	0.43
4:E:128:VAL:HG23	4:E:152:PHE:CE1	2.54	0.43
1:F:120:MET:O	1:F:124:VAL:HG23	2.19	0.43
1:F:342:GLU:C	1:F:343:ARG:HG3	2.39	0.43
1:A:89[B]:GLU:HG2	1:A:92[B]:TRP:HB2	2.01	0.42
1:A:105[B]:ARG:NH2	7:H:950:FMT:C	2.82	0.42
4:E:46:PRO:HB2	4:E:47:TYR:CD2	2.54	0.42
2:B:392:LYS:HG3	2:B:399:GLY:O	2.19	0.42
3:C:44[A]:ARG:NE	10:G:1047:HOH:O	2.27	0.42
3:C:332:ASP:HB3	7:H:928:FMT:C	2.48	0.42
1:D:166[B]:GLU:CG	4:E:228:HIS:NE2	2.78	0.42
1:D:227:ASP:O	1:D:228:HIS:HB2	2.19	0.42
1:D:392:LYS:HG3	1:D:399:GLY:O	2.19	0.42
4:E:104:LEU:HD12	4:E:107:LEU:CD1	2.49	0.42
1:F:309[A]:VAL:HG12	1:F:310:THR:N	2.34	0.42
2:B:92:TRP:CE3	9:K:7[B]:GOL:H32	2.55	0.42
2:B:106[A]:ARG:CZ	7:H:1060:FMT:C	2.97	0.42
1:D:322:ALA:O	1:D:326:ARG:HG2	2.20	0.42
4:E:290:LEU:O	4:E:397:ILE:HA	2.18	0.42
1:A:256:LEU:HD22	1:A:284:MET:CB	2.47	0.42
1:D:83[A]:MET:HE3	10:G:243:HOH:O	2.19	0.42
1:D:101:HIS:HE1	1:D:353:ALA:O	2.02	0.42
4:E:105:ARG:NH2	4:E:355:HIS:O	2.28	0.42
4:E:259:LEU:HB2	4:E:284[A]:MET:HE1	2.01	0.42
2:B:8[A]:PRO:HB3	2:B:12:ASP:HB2	2.00	0.42
2:B:390:LYS:HE3	2:B:402[A]:ARG:HH21	1.84	0.42
1:D:219:LEU:O	1:D:222:ALA:HB3	2.20	0.42
1:F:376:ARG:HE	1:F:376:ARG:HB3	1.70	0.42
1:A:375[B]:ARG:HG3	1:A:375[B]:ARG:NH2	2.35	0.42
2:B:174:PHE:O	2:B:178[B]:MET:HG2	2.20	0.42
3:C:8:PRO:C	3:C:10:PRO:HD2	2.40	0.42
1:A:295:SER:CB	1:A:319[A]:VAL:HG22	2.49	0.42
2:B:9[A]:THR:OG1	2:B:10[A]:PRO:CD	2.68	0.42
2:B:161:LEU:O	2:B:216[B]:LEU:CD1	2.54	0.42

Continued on next page...

PRELIMINARY VALIDATION REPORT

Continued from previous page...

Atom-1	Atom-2	Interatomic distance (Å)	Clash overlap (Å)
1:A:165:LEU:HD12	1:A:165:LEU:HA	1.79	0.41
4:E:328:GLU:HB2	10:G:273:HOH:O	2.19	0.41
1:A:38[A]:GLU:CD	1:A:41:SER:HB3	2.41	0.41
1:A:355:HIS:HE2	7:H:951:FMT:C	2.32	0.41
5:B:501:HEM:HBB2	5:B:501:HEM:HMB2	2.02	0.41
1:A:244:ALA:HB1	5:A:501:HEM:C4C	2.55	0.41
2:B:287:TYR:O	2:B:326[A]:ARG:NH1	2.54	0.41
1:D:188:GLU:OE1	7:H:66:FMT:O1	2.37	0.41
1:A:58[B]:MET:CG	1:A:324:ALA:O	2.68	0.41
2:B:161:LEU:CA	2:B:216[B]:LEU:HD13	2.50	0.41
1:F:271:VAL:CG2	1:F:374:VAL:HG13	2.48	0.41
1:F:152:PHE:HB3	1:F:153:PRO:HD3	2.02	0.41
1:F:259:LEU:HA	1:F:262:THR:HG22	2.03	0.41
1:D:256:LEU:HD12	1:D:370[A]:LEU:CD1	2.48	0.41
4:E:86:THR:HG21	4:E:189:ILE:HG22	2.02	0.41
1:F:280:ALA:O	1:F:284[B]:MET:HG3	2.19	0.41
1:A:183:ARG:HD2	7:H:911:FMT:O1	2.21	0.41
2:B:101:HIS:CE1	2:B:105[B]:ARG:HD3	2.56	0.41
2:B:124:VAL:HG13	2:B:152:PHE:HE1	1.86	0.41
1:D:256:LEU:CD1	1:D:370[A]:LEU:HD11	2.51	0.41
4:E:208:ARG:NH1	4:E:223:THR:HG22	2.36	0.41
2:B:11[A]:ALA:O	2:B:44:ARG:NH2	2.54	0.41
3:C:382:LEU:HD12	3:C:382:LEU:HA	1.97	0.41
7:H:1040:FMT:H	10:G:1040:HOH:O	2.20	0.41
1:A:252:GLN:OE1	1:A:252:GLN:HA	2.21	0.40
1:A:258:HIS:HD2	10:G:721:HOH:O	2.03	0.40
1:D:83[B]:MET:HE2	1:D:318:VAL:HG11	2.02	0.40
4:E:178[B]:MET:CE	4:E:178[B]:MET:HA	2.51	0.40
1:F:161:LEU:CD2	1:F:215:LEU:HD12	2.43	0.40
1:D:23:HIS:O	1:D:26:ASP:HB2	2.21	0.40
1:A:337:LEU:HD23	1:A:337:LEU:HA	1.91	0.40
3:C:92[B]:TRP:HZ3	7:H:1046:FMT:O1	2.04	0.40
1:D:58[B]:MET:HA	1:D:58[B]:MET:CE	2.51	0.40
1:D:290:LEU:O	1:D:397:ILE:HA	2.21	0.40
4:E:204:VAL:HG13	4:E:220:ALA:HB2	2.04	0.40
1:F:281:VAL:HA	1:F:284[B]:MET:HE3	2.02	0.40
1:F:373:LEU:HD23	1:F:373:LEU:HA	1.91	0.40
2:B:252:GLN:HA	2:B:252:GLN:OE1	2.21	0.40
1:A:256:LEU:CD1	1:A:370:LEU:HD11	2.51	0.40
2:B:170:LEU:HD12	2:B:170:LEU:HA	1.96	0.40
3:C:42[A]:ARG:HG2	3:C:53:TRP:CE3	2.57	0.40

Continued on next page...

PRELIMINARY VALIDATION REPORT

Continued from previous page...

Atom-1	Atom-2	Interatomic distance (Å)	Clash overlap (Å)
1:D:286:ARG:HG3	1:D:325:ASN:HB3	2.03	0.40

All (2) symmetry-related close contacts are listed below. The label for Atom-2 includes the symmetry operator and encoded unit-cell translations to be applied.

Atom-1	Atom-2	Interatomic distance (Å)	Clash overlap (Å)
7:H:32:FMT:O2	10:G:680:HOH:O[3_555]	2.11	0.09
2:B:302:GLU:OE2	3:C:208:ARG:NH2[4_556]	2.14	0.06

5.3 Torsion angles (i)

5.3.1 Protein backbone (i)

In the following table, the Percentiles column shows the percent Ramachandran outliers of the chain as a percentile score with respect to all X-ray entries followed by that with respect to entries of similar resolution.

The Analysed column shows the number of residues for which the backbone conformation was analysed, and the total number of residues.

Mol	Chain	Analysed	Favoured	Allowed	Outliers	Percentiles	
1	A	426/396 (108%)	413 (97%)	13 (3%)	0	100	100
1	D	410/396 (104%)	388 (95%)	22 (5%)	0	100	100
1	F	400/396 (101%)	375 (94%)	24 (6%)	1 (0%)	41	39
2	B	433/402 (108%)	415 (96%)	17 (4%)	1 (0%)	47	47
3	C	435/408 (107%)	426 (98%)	9 (2%)	0	100	100
4	E	402/395 (102%)	385 (96%)	17 (4%)	0	100	100
All	All	2506/2393 (105%)	2402 (96%)	102 (4%)	2 (0%)	51	53

All (2) Ramachandran outliers are listed below:

Mol	Chain	Res	Type
1	F	264	ARG
2	B	68	SER

PRELIMINARY VALIDATION REPORT

5.3.2 Protein sidechains (i)

In the following table, the Percentiles column shows the percent sidechain outliers of the chain as a percentile score with respect to all X-ray entries followed by that with respect to entries of similar resolution.

The Analysed column shows the number of residues for which the sidechain conformation was analysed, and the total number of residues.

Mol	Chain	Analysed	Rotameric	Outliers	Percentiles	
1	A	365/333 (110%)	356 (98%)	9 (2%)	47	50
1	D	349/333 (105%)	342 (98%)	7 (2%)	55	59
1	F	339/333 (102%)	334 (98%)	5 (2%)	65	69
2	B	369/337 (110%)	362 (98%)	7 (2%)	57	61
3	C	372/343 (108%)	367 (99%)	5 (1%)	69	74
4	E	341/332 (103%)	334 (98%)	7 (2%)	53	57
All	All	2135/2011 (106%)	2095 (98%)	40 (2%)	60	61

All (40) residues with a non-rotameric sidechain are listed below:

Mol	Chain	Res	Type
1	A	166	GLU
1	A	183	ARG
1	A	190	GLN
1	A	191[A]	ARG
1	A	191[B]	ARG
1	A	196	PHE
1	A	224	ASP
1	A	337	LEU
1	A	381	ASP
2	B	9[A]	THR
2	B	9[B]	THR
2	B	36	ARG
2	B	196	PHE
2	B	221	LEU
2	B	264[A]	ARG
2	B	264[B]	ARG
3	C	12	ASP
3	C	27[A]	LEU
3	C	27[B]	LEU
3	C	116	ARG
3	C	196	PHE
1	D	89	GLU

Continued on next page...

PRELIMINARY VALIDATION REPORT

Continued from previous page...

Mol	Chain	Res	Type
1	D	94	LEU
1	D	115[A]	ARG
1	D	115[B]	ARG
1	D	196	PHE
1	D	207	ARG
1	D	312	ARG
4	E	42	ARG
4	E	106[A]	ARG
4	E	106[B]	ARG
4	E	196	PHE
4	E	225	ASN
4	E	264	ARG
4	E	326	ARG
1	F	42	ARG
1	F	183	ARG
1	F	196	PHE
1	F	231	LYS
1	F	312	ARG

Sometimes sidechains can be flipped to improve hydrogen bonding and reduce clashes. All (7) such sidechains are listed below:

Mol	Chain	Res	Type
1	A	228	HIS
2	B	193	GLN
3	C	0	HIS
3	C	190	GLN
3	C	236	ASN
4	E	225	ASN
1	F	333	HIS

5.3.3 RNA ⓘ

There are no RNA molecules in this entry.

5.4 Non-standard residues in protein, DNA, RNA chains ⓘ

There are no non-standard protein/DNA/RNA residues in this entry.

PRELIMINARY VALIDATION REPORT

5.5 Carbohydrates (i)

There are no monosaccharides in this entry.

5.6 Ligand geometry (i)

Of 172 ligands modelled in this entry, 8 are monoatomic - leaving 164 for Mogul analysis.

In the following table, the Counts columns list the number of bonds (or angles) for which Mogul statistics could be retrieved, the number of bonds (or angles) that are observed in the model and the number of bonds (or angles) that are defined in the Chemical Component Dictionary. The Link column lists molecule types, if any, to which the group is linked. The Z score for a bond length (or angle) is the number of standard deviations the observed value is removed from the expected value. A bond length (or angle) with $|Z| > 2$ is considered an outlier worth inspection. RMSZ is the root-mean-square of all Z scores of the bond lengths (or angles).

Mol	Type	Chain	Res	Link	Bond lengths			Bond angles		
					Counts	RMSZ	$\#\|Z\| > 2$	Counts	RMSZ	$\#\|Z\| > 2$
6	DEB	E	502	-	27,27,27	0.50	0	35,39,39	0.77	0
7	FMT	H	62	-	0,2,2	-	-	0,1,1	-	-
7	FMT	H	905	-	0,2,2	-	-	0,1,1	-	-
7	FMT	H	929	-	0,2,2	-	-	0,1,1	-	-
7	FMT	H	1030	-	0,2,2	-	-	0,1,1	-	-
7	FMT	H	93	-	0,2,2	-	-	0,1,1	-	-
7	FMT	H	1015	-	0,2,2	-	-	0,1,1	-	-
7	FMT	H	920	-	0,2,2	-	-	0,1,1	-	-
7	FMT	H	31	-	0,2,2	-	-	0,1,1	-	-
7	FMT	H	1047	-	0,2,2	-	-	0,1,1	-	-
7	FMT	H	904	-	0,2,2	-	-	0,1,1	-	-
7	FMT	H	1018	-	0,2,2	-	-	0,1,1	-	-
7	FMT	H	12	-	0,2,2	-	-	0,1,1	-	-
7	FMT	H	1023	-	0,2,2	-	-	0,1,1	-	-
7	FMT	H	961	-	0,2,2	-	-	0,1,1	-	-
7	FMT	H	979	-	0,2,2	-	-	0,1,1	-	-
7	FMT	H	906	-	0,2,2	-	-	0,1,1	-	-
7	FMT	H	1069	-	0,2,2	-	-	0,1,1	-	-
7	FMT	H	989	-	0,2,2	-	-	0,1,1	-	-
7	FMT	H	969	-	0,2,2	-	-	0,1,1	-	-
7	FMT	H	995	-	0,2,2	-	-	0,1,1	-	-
7	FMT	H	65	-	0,2,2	-	-	0,1,1	-	-
7	FMT	H	1065	-	0,2,2	-	-	0,1,1	-	-
7	FMT	H	915	-	0,2,2	-	-	0,1,1	-	-
5	HEM	F	501	1	27,50,50	0.90	1 (3%)	17,82,82	1.64	4 (23%)
7	FMT	H	945	-	0,2,2	-	-	0,1,1	-	-
7	FMT	H	1061	-	0,2,2	-	-	0,1,1	-	-

PRELIMINARY VALIDATION REPORT

Mol	Type	Chain	Res	Link	Bond lengths			Bond angles		
					Counts	RMSZ	#|Z| > 2	Counts	RMSZ	#|Z| > 2
7	FMT	H	41	-	0,2,2	-	-	0,1,1	-	-
7	FMT	H	982	-	0,2,2	-	-	0,1,1	-	-
7	FMT	H	1058	-	0,2,2	-	-	0,1,1	-	-
7	FMT	H	981	-	0,2,2	-	-	0,1,1	-	-
7	FMT	H	1025	-	0,2,2	-	-	0,1,1	-	-
5	HEM	E	501	4	27,50,50	1.44	5 (18%)	17,82,82	2.35	7 (41%)
7	FMT	H	1021	-	0,2,2	-	-	0,1,1	-	-
6	DEB	A	502	-	27,27,27	0.26	0	35,39,39	0.51	0
7	FMT	H	972	-	0,2,2	-	-	0,1,1	-	-
9	GOL	K	13	-	5,5,5	0.20	0	5,5,5	0.65	0
9	GOL	K	7[B]	-	5,5,5	0.23	0	5,5,5	0.43	0
7	FMT	H	58	-	0,2,2	-	-	0,1,1	-	-
7	FMT	H	946	-	0,2,2	-	-	0,1,1	-	-
7	FMT	H	95	-	0,2,2	-	-	0,1,1	-	-
7	FMT	H	74	-	0,2,2	-	-	0,1,1	-	-
7	FMT	H	1008	-	0,2,2	-	-	0,1,1	-	-
7	FMT	H	965	-	0,2,2	-	-	0,1,1	-	-
7	FMT	H	96	-	0,2,2	-	-	0,1,1	-	-
9	GOL	K	8[B]	-	5,5,5	0.10	0	5,5,5	0.28	0
7	FMT	H	998	-	0,2,2	-	-	0,1,1	-	-
7	FMT	H	933	-	0,2,2	-	-	0,1,1	-	-
7	FMT	H	6	-	0,2,2	-	-	0,1,1	-	-
7	FMT	H	941	-	0,2,2	-	-	0,1,1	-	-
7	FMT	H	960	-	0,2,2	-	-	0,1,1	-	-
7	FMT	H	1045	-	0,2,2	-	-	0,1,1	-	-
7	FMT	H	33	-	0,2,2	-	-	0,1,1	-	-
7	FMT	H	66	-	0,2,2	-	-	0,1,1	-	-
9	GOL	K	1[A]	-	5,5,5	0.09	0	5,5,5	0.41	0
9	GOL	K	11	-	5,5,5	0.14	0	5,5,5	0.32	0
7	FMT	H	1053	-	0,2,2	-	-	0,1,1	-	-
7	FMT	H	56	-	0,2,2	-	-	0,1,1	-	-
7	FMT	H	938	-	0,2,2	-	-	0,1,1	-	-
7	FMT	H	1019	-	0,2,2	-	-	0,1,1	-	-
7	FMT	H	94	-	0,2,2	-	-	0,1,1	-	-
7	FMT	H	7	-	0,2,2	-	-	0,1,1	-	-
7	FMT	H	1020	-	0,2,2	-	-	0,1,1	-	-
7	FMT	H	996	-	0,2,2	-	-	0,1,1	-	-
7	FMT	H	1046	-	0,2,2	-	-	0,1,1	-	-
7	FMT	H	20	-	0,2,2	-	-	0,1,1	-	-
7	FMT	H	27	-	0,2,2	-	-	0,1,1	-	-
9	GOL	K	4[A]	-	5,5,5	0.09	0	5,5,5	0.30	0
7	FMT	H	971	-	0,2,2	-	-	0,1,1	-	-
7	FMT	H	1003	-	0,2,2	-	-	0,1,1	-	-

PRELIMINARY VALIDATION REPORT

Mol	Type	Chain	Res	Link	Bond lengths			Bond angles		
					Counts	RMSZ	#|Z| > 2	Counts	RMSZ	#|Z| > 2
7	FMT	H	901	-	0,2,2	-	-	0,1,1	-	-
7	FMT	H	968	-	0,2,2	-	-	0,1,1	-	-
7	FMT	H	910	-	0,2,2	-	-	0,1,1	-	-
7	FMT	H	1036	-	0,2,2	-	-	0,1,1	-	-
7	FMT	H	1028	-	0,2,2	-	-	0,1,1	-	-
7	FMT	H	986	-	0,2,2	-	-	0,1,1	-	-
7	FMT	H	1001	-	0,2,2	-	-	0,1,1	-	-
7	FMT	H	937	-	0,2,2	-	-	0,1,1	-	-
9	GOL	K	9	-	5,5,5	0.26	0	5,5,5	0.56	0
9	GOL	K	7[A]	-	5,5,5	0.32	0	5,5,5	0.38	0
7	FMT	H	1033	-	0,2,2	-	-	0,1,1	-	-
7	FMT	H	1010	-	0,2,2	-	-	0,1,1	-	-
7	FMT	H	912	-	0,2,2	-	-	0,1,1	-	-
7	FMT	H	70	-	0,2,2	-	-	0,1,1	-	-
7	FMT	H	57	-	0,2,2	-	-	0,1,1	-	-
7	FMT	H	976	-	0,2,2	-	-	0,1,1	-	-
7	FMT	H	911	-	0,2,2	-	-	0,1,1	-	-
7	FMT	H	914	-	0,2,2	-	-	0,1,1	-	-
7	FMT	H	925	-	0,2,2	-	-	0,1,1	-	-
7	FMT	H	1009	-	0,2,2	-	-	0,1,1	-	-
9	GOL	K	3	-	5,5,5	0.22	0	5,5,5	0.59	0
7	FMT	H	934	-	0,2,2	-	-	0,1,1	-	-
7	FMT	H	980	-	0,2,2	-	-	0,1,1	-	-
7	FMT	H	1044	-	0,2,2	-	-	0,1,1	-	-
9	GOL	K	5	-	5,5,5	0.13	0	5,5,5	0.31	0
7	FMT	H	928	-	0,2,2	-	-	0,1,1	-	-
7	FMT	H	931	-	0,2,2	-	-	0,1,1	-	-
7	FMT	H	1068	-	0,2,2	-	-	0,1,1	-	-
7	FMT	H	951	-	0,2,2	-	-	0,1,1	-	-
7	FMT	H	984	-	0,2,2	-	-	0,1,1	-	-
7	FMT	H	13	-	0,2,2	-	-	0,1,1	-	-
7	FMT	H	10	-	0,2,2	-	-	0,1,1	-	-
7	FMT	H	921	-	0,2,2	-	-	0,1,1	-	-
5	HEM	C	501	3	27,50,50	1.15	4 (14%)	17,82,82	1.77	6 (35%)
6	DEB	D	502	-	27,27,27	0.22	0	35,39,39	0.47	0
7	FMT	H	923	-	0,2,2	-	-	0,1,1	-	-
7	FMT	H	1064	-	0,2,2	-	-	0,1,1	-	-
7	FMT	H	902	8	0,2,2	-	-	0,1,1	-	-
7	FMT	H	8	-	0,2,2	-	-	0,1,1	-	-
7	FMT	H	1060	-	0,2,2	-	-	0,1,1	-	-
7	FMT	H	72	-	0,2,2	-	-	0,1,1	-	-
7	FMT	H	947	-	0,2,2	-	-	0,1,1	-	-

PRELIMINARY VALIDATION REPORT

Mol	Type	Chain	Res	Link	Bond lengths			Bond angles		
					Counts	RMSZ	#|Z| > 2	Counts	RMSZ	#|Z| > 2
6	DEB	B	502	-	27,27,27	0.51	0	35,39,39	1.08	2 (5%)
7	FMT	H	926	-	0,2,2	-	-	0,1,1	-	-
7	FMT	H	1038	-	0,2,2	-	-	0,1,1	-	-
7	FMT	H	922	-	0,2,2	-	-	0,1,1	-	-
7	FMT	H	935	-	0,2,2	-	-	0,1,1	-	-
7	FMT	H	1014	-	0,2,2	-	-	0,1,1	-	-
7	FMT	H	1040	-	0,2,2	-	-	0,1,1	-	-
7	FMT	H	67	-	0,2,2	-	-	0,1,1	-	-
7	FMT	H	918	-	0,2,2	-	-	0,1,1	-	-
7	FMT	H	601	-	0,2,2	-	-	0,1,1	-	-
7	FMT	H	943	-	0,2,2	-	-	0,1,1	-	-
7	FMT	H	944	-	0,2,2	-	-	0,1,1	-	-
6	DEB	Z	502	-	27,27,27	0.27	0	35,39,39	0.61	1 (2%)
7	FMT	H	1006	-	0,2,2	-	-	0,1,1	-	-
7	FMT	H	1005	-	0,2,2	-	-	0,1,1	-	-
7	FMT	H	1048	-	0,2,2	-	-	0,1,1	-	-
9	GOL	K	8[A]	-	5,5,5	0.24	0	5,5,5	0.48	0
7	FMT	H	917	-	0,2,2	-	-	0,1,1	-	-
9	GOL	K	1[B]	-	5,5,5	0.22	0	5,5,5	0.65	0
7	FMT	H	14	-	0,2,2	-	-	0,1,1	-	-
7	FMT	H	913	-	0,2,2	-	-	0,1,1	-	-
7	FMT	H	11	-	0,2,2	-	-	0,1,1	-	-
7	FMT	H	29	-	0,2,2	-	-	0,1,1	-	-
7	FMT	H	32	-	0,2,2	-	-	0,1,1	-	-
7	FMT	H	909	-	0,2,2	-	-	0,1,1	-	-
7	FMT	H	939	-	0,2,2	-	-	0,1,1	-	-
7	FMT	H	1007	-	0,2,2	-	-	0,1,1	-	-
7	FMT	H	64	-	0,2,2	-	-	0,1,1	-	-
7	FMT	H	966	-	0,2,2	-	-	0,1,1	-	-
7	FMT	H	1024	-	0,2,2	-	-	0,1,1	-	-
9	GOL	K	4[B]	-	5,5,5	0.09	0	5,5,5	0.29	0
7	FMT	H	21	-	0,2,2	-	-	0,1,1	-	-
7	FMT	H	40	-	0,2,2	-	-	0,1,1	-	-
7	FMT	H	948	-	0,2,2	-	-	0,1,1	-	-
7	FMT	H	19	-	0,2,2	-	-	0,1,1	-	-
7	FMT	H	1004	-	0,2,2	-	-	0,1,1	-	-
7	FMT	H	1062	-	0,2,2	-	-	0,1,1	-	-
5	HEM	B	501	2	27,50,50	0.99	2 (7%)	17,82,82	2.56	8 (47%)
6	DEB	F	502	-	27,27,27	0.59	1 (3%)	35,39,39	0.95	1 (2%)
5	HEM	A	501	1	27,50,50	1.21	3 (11%)	17,82,82	1.65	5 (29%)
5	HEM	D	501	1	27,50,50	1.47	2 (7%)	17,82,82	2.10	7 (41%)
7	FMT	H	970	-	0,2,2	-	-	0,1,1	-	-

PRELIMINARY VALIDATION REPORT

Mol	Type	Chain	Res	Link	Bond lengths			Bond angles		
					Counts	RMSZ	#|Z| > 2	Counts	RMSZ	#|Z| > 2
7	FMT	H	908	-	0,2,2	-	-	0,1,1	-	-
7	FMT	H	71	-	0,2,2	-	-	0,1,1	-	-
7	FMT	H	950	-	0,2,2	-	-	0,1,1	-	-
7	FMT	H	997	-	0,2,2	-	-	0,1,1	-	-
7	FMT	H	940	-	0,2,2	-	-	0,1,1	-	-
7	FMT	H	15	-	0,2,2	-	-	0,1,1	-	-
7	FMT	H	932	-	0,2,2	-	-	0,1,1	-	-
7	FMT	H	955	-	0,2,2	-	-	0,1,1	-	-
7	FMT	H	942	-	0,2,2	-	-	0,1,1	-	-
7	FMT	H	919	-	0,2,2	-	-	0,1,1	-	-

In the following table, the Chirals column lists the number of chiral outliers, the number of chiral centers analysed, the number of these observed in the model and the number defined in the Chemical Component Dictionary. Similar counts are reported in the Torsion and Rings columns. '-' means no outliers of that kind were identified.

Mol	Type	Chain	Res	Link	Chirals	Torsions	Rings
6	DEB	E	502	-	-	9/50/50/50	0/1/1/1
9	GOL	K	8[B]	-	-	2/4/4/4	-
9	GOL	K	8[A]	-	-	4/4/4/4	-
9	GOL	K	1[B]	-	-	1/4/4/4	-
9	GOL	K	3	-	-	4/4/4/4	-
9	GOL	K	11	-	-	3/4/4/4	-
9	GOL	K	1[A]	-	-	3/4/4/4	-
9	GOL	K	5	-	-	2/4/4/4	-
9	GOL	K	4[B]	-	-	4/4/4/4	-
5	HEM	C	501	3	-	0/6/54/54	-
6	DEB	D	502	-	-	8/50/50/50	0/1/1/1
9	GOL	K	4[A]	-	-	2/4/4/4	-
6	DEB	B	502	-	-	7/50/50/50	0/1/1/1
5	HEM	F	501	1	-	0/6/54/54	-
5	HEM	B	501	2	-	0/6/54/54	-
6	DEB	F	502	-	-	8/50/50/50	0/1/1/1
5	HEM	A	501	1	-	0/6/54/54	-
5	HEM	D	501	1	-	0/6/54/54	-
5	HEM	E	501	4	-	0/6/54/54	-
6	DEB	A	502	-	-	8/50/50/50	0/1/1/1
9	GOL	K	13	-	-	2/4/4/4	-
9	GOL	K	7[B]	-	-	1/4/4/4	-

Continued on next page...

PRELIMINARY VALIDATION REPORT

Continued from previous page...

Mol	Type	Chain	Res	Link	Chirals	Torsions	Rings
9	GOL	K	9	-	-	2/4/4/4	-
9	GOL	K	7[A]	-	-	4/4/4/4	-
6	DEB	Z	502	-	-	10/50/50/50	0/1/1/1

All (18) bond length outliers are listed below:

Mol	Chain	Res	Type	Atoms	Z	Observed(Å)	Ideal(Å)
5	D	501	HEM	C3B-C2B	-4.22	1.34	1.40
5	E	501	HEM	C3B-C2B	-3.78	1.35	1.40
5	E	501	HEM	C3D-C4D	3.55	1.50	1.42
5	E	501	HEM	CAA-C2A	2.78	1.56	1.52
5	D	501	HEM	C1D-ND	-2.74	1.30	1.36
5	E	501	HEM	C1A-NA	2.74	1.41	1.36
5	B	501	HEM	C3D-C2D	-2.67	1.29	1.37
5	C	501	HEM	C3D-C4D	2.63	1.48	1.42
5	A	501	HEM	C3B-C2B	-2.49	1.36	1.40
6	F	502	DEB	O24-C9	2.40	1.25	1.21
5	A	501	HEM	C3D-C4D	2.38	1.47	1.42
5	C	501	HEM	C3D-C2D	-2.29	1.30	1.37
5	A	501	HEM	CMC-C2C	2.23	1.56	1.51
5	E	501	HEM	C3C-C2C	-2.22	1.37	1.40
5	C	501	HEM	CAA-C2A	2.21	1.55	1.52
5	C	501	HEM	C4A-NA	2.15	1.40	1.36
5	B	501	HEM	C1D-ND	-2.09	1.31	1.36
5	F	501	HEM	C3D-C4D	2.02	1.47	1.42

All (41) bond angle outliers are listed below:

Mol	Chain	Res	Type	Atoms	Z	Observed(^{o})	Ideal(^{o})
5	B	501	HEM	CMA-C3A-C4A	-5.43	120.12	128.46
5	E	501	HEM	C4A-C3A-C2A	5.11	110.55	107.00
5	B	501	HEM	CAD-CBD-CGD	4.30	119.89	112.67
5	B	501	HEM	CMC-C2C-C3C	4.05	132.25	124.68
5	C	501	HEM	C4A-C3A-C2A	3.88	109.69	107.00
5	D	501	HEM	CMC-C2C-C3C	3.83	131.85	124.68
5	E	501	HEM	C2C-C3C-C4C	3.69	109.47	106.90
5	A	501	HEM	C2C-C3C-C4C	3.59	109.41	106.90
5	B	501	HEM	CMA-C3A-C2A	3.45	131.45	124.94
5	E	501	HEM	C1D-C2D-C3D	-3.33	104.68	107.00
5	D	501	HEM	CMD-C2D-C3D	-3.32	118.68	124.94
5	D	501	HEM	CAD-CBD-CGD	3.28	118.17	112.67

Continued on next page...

PRELIMINARY VALIDATION REPORT

Continued from previous page...

Mol	Chain	Res	Type	Atoms	Z	Observed(°)	Ideal(°)
5	F	501	HEM	CBD-CAD-C3D	-3.22	106.55	112.48
5	F	501	HEM	CMC-C2C-C3C	3.14	130.56	124.68
5	E	501	HEM	CBD-CAD-C3D	-3.03	106.89	112.48
5	E	501	HEM	CBA-CAA-C2A	3.00	118.01	112.49
6	F	502	DEB	C10-C11-C12	2.86	120.30	114.41
5	D	501	HEM	C1D-C2D-C3D	2.84	108.97	107.00
5	C	501	HEM	C2C-C3C-C4C	2.81	108.86	106.90
5	B	501	HEM	CMD-C2D-C1D	2.77	132.72	128.46
5	D	501	HEM	CBA-CAA-C2A	2.73	117.53	112.49
5	A	501	HEM	C4A-C3A-C2A	2.73	108.89	107.00
5	B	501	HEM	CBA-CAA-C2A	2.71	117.48	112.49
5	D	501	HEM	C4A-C3A-C2A	2.59	108.80	107.00
5	C	501	HEM	CMA-C3A-C4A	-2.45	124.69	128.46
5	A	501	HEM	CAD-CBD-CGD	2.43	116.75	112.67
5	C	501	HEM	CMC-C2C-C3C	2.39	129.15	124.68
5	E	501	HEM	C3B-C4B-NB	-2.36	106.16	109.21
5	B	501	HEM	CMD-C2D-C3D	-2.35	120.50	124.94
5	B	501	HEM	CBD-CAD-C3D	-2.33	108.18	112.48
5	A	501	HEM	C3C-C4C-NC	-2.32	106.56	110.94
5	E	501	HEM	CMD-C2D-C3D	2.29	129.26	124.94
6	B	502	DEB	O21-C5-C6	-2.27	105.56	109.83
5	A	501	HEM	CMC-C2C-C3C	2.24	128.87	124.68
5	C	501	HEM	CBD-CAD-C3D	-2.22	108.38	112.48
5	F	501	HEM	C2C-C3C-C4C	2.13	108.39	106.90
5	D	501	HEM	CMD-C2D-C1D	2.12	131.72	128.46
6	Z	502	DEB	C10-C11-C12	2.11	118.77	114.41
5	F	501	HEM	CAD-CBD-CGD	2.11	116.20	112.67
5	C	501	HEM	CBA-CAA-C2A	2.10	116.36	112.49
6	B	502	DEB	O26-C11-C10	2.10	113.70	108.82

There are no chirality outliers.

All (84) torsion outliers are listed below:

Mol	Chain	Res	Type	Atoms
6	Z	502	DEB	C3-C4-C5-O21
6	Z	502	DEB	C20-C4-C5-O21
6	Z	502	DEB	O16-C13-C14-C15
9	K	1[A]	GOL	O1-C1-C2-C3
9	K	4[B]	GOL	O1-C1-C2-C3
9	K	4[B]	GOL	C1-C2-C3-O3
9	K	5	GOL	C1-C2-C3-O3
9	K	5	GOL	O2-C2-C3-O3

Continued on next page...

PRELIMINARY VALIDATION REPORT

Continued from previous page...

Mol	Chain	Res	Type	Atoms
9	K	8[A]	GOL	O1-C1-C2-C3
9	K	8[A]	GOL	C1-C2-C3-O3
9	K	8[B]	GOL	O1-C1-C2-C3
9	K	11	GOL	C1-C2-C3-O3
9	K	13	GOL	O1-C1-C2-C3
6	A	502	DEB	C3-C4-C5-O21
6	D	502	DEB	C3-C4-C5-O21
6	Z	502	DEB	C20-C4-C5-C6
6	Z	502	DEB	C3-C4-C5-C6
9	K	1[A]	GOL	O1-C1-C2-O2
9	K	4[A]	GOL	O1-C1-C2-O2
9	K	4[B]	GOL	O2-C2-C3-O3
9	K	8[B]	GOL	O1-C1-C2-O2
6	A	502	DEB	C20-C4-C5-O21
6	D	502	DEB	C20-C4-C5-O21
9	K	1[A]	GOL	C1-C2-C3-O3
9	K	3	GOL	O1-C1-C2-C3
9	K	3	GOL	C1-C2-C3-O3
9	K	4[A]	GOL	O1-C1-C2-C3
9	K	7[A]	GOL	O1-C1-C2-C3
9	K	7[A]	GOL	C1-C2-C3-O3
9	K	9	GOL	O1-C1-C2-C3
9	K	4[B]	GOL	O1-C1-C2-O2
6	E	502	DEB	C18-C2-C3-O19
6	F	502	DEB	C18-C2-C3-O19
6	A	502	DEB	C18-C2-C3-C4
6	B	502	DEB	C18-C2-C3-C4
6	D	502	DEB	C18-C2-C3-C4
6	E	502	DEB	C18-C2-C3-C4
6	F	502	DEB	C18-C2-C3-C4
6	Z	502	DEB	C18-C2-C3-C4
9	K	3	GOL	O2-C2-C3-O3
9	K	8[A]	GOL	O1-C1-C2-O2
6	E	502	DEB	C3-C4-C5-O21
6	A	502	DEB	C18-C2-C3-O19
6	B	502	DEB	C18-C2-C3-O19
6	D	502	DEB	C18-C2-C3-O19
6	Z	502	DEB	C18-C2-C3-O19
9	K	1[B]	GOL	O1-C1-C2-O2
9	K	7[A]	GOL	O2-C2-C3-O3
9	K	9	GOL	O1-C1-C2-O2
9	K	13	GOL	O1-C1-C2-O2

Continued on next page...

PRELIMINARY VALIDATION REPORT

Continued from previous page...

Mol	Chain	Res	Type	Atoms
9	K	3	GOL	O1-C1-C2-O2
9	K	7[A]	GOL	O1-C1-C2-O2
6	F	502	DEB	C3-C4-C5-O21
6	D	502	DEB	C3-C4-C5-C6
6	Z	502	DEB	C12-C13-C14-C15
6	E	502	DEB	C23-C8-C9-O24
6	B	502	DEB	C3-C4-C5-O21
6	A	502	DEB	C1-C2-C3-C4
6	A	502	DEB	C1-C2-C3-O19
6	B	502	DEB	C1-C2-C3-C4
6	B	502	DEB	C1-C2-C3-O19
6	D	502	DEB	C1-C2-C3-C4
6	D	502	DEB	C1-C2-C3-O19
6	E	502	DEB	C1-C2-C3-C4
6	E	502	DEB	C1-C2-C3-O19
6	F	502	DEB	C1-C2-C3-C4
6	F	502	DEB	C1-C2-C3-O19
6	Z	502	DEB	C1-C2-C3-C4
6	Z	502	DEB	C1-C2-C3-O19
9	K	11	GOL	O2-C2-C3-O3
6	D	502	DEB	C20-C4-C5-C6
6	E	502	DEB	C20-C4-C5-O21
6	A	502	DEB	C3-C4-C5-C6
6	A	502	DEB	C20-C4-C5-C6
6	F	502	DEB	C2-C1-O16-C13
6	E	502	DEB	C2-C1-O16-C13
9	K	8[A]	GOL	O2-C2-C3-O3
6	B	502	DEB	O16-C13-C14-C15
9	K	7[B]	GOL	C1-C2-C3-O3
9	K	11	GOL	O1-C1-C2-C3
6	B	502	DEB	C3-C4-C5-C6
6	F	502	DEB	C23-C8-C9-O24
6	E	502	DEB	C7-C8-C9-O24
6	F	502	DEB	C7-C8-C9-O24

There are no ring outliers.

47 monomers are involved in 104 short contacts:

Mol	Chain	Res	Type	Clashes	Symm-Clashes
7	H	31	FMT	2	0
7	H	1018	FMT	1	0
7	H	906	FMT	1	0

Continued on next page...

PRELIMINARY VALIDATION REPORT

Continued from previous page...

Mol	Chain	Res	Type	Clashes	Symm-Clashes
7	H	915	FMT	1	0
5	F	501	HEM	3	0
7	H	1061	FMT	2	0
5	E	501	HEM	2	0
6	A	502	DEB	8	0
9	K	13	GOL	3	0
9	K	7[B]	GOL	4	0
7	H	1008	FMT	1	0
9	K	8[B]	GOL	3	0
7	H	960	FMT	2	0
7	H	33	FMT	2	0
7	H	66	FMT	3	0
9	K	1[A]	GOL	1	0
7	H	1053	FMT	1	0
7	H	938	FMT	1	0
7	H	1046	FMT	1	0
7	H	20	FMT	1	0
9	K	4[A]	GOL	7	0
9	K	7[A]	GOL	9	0
7	H	976	FMT	1	0
7	H	911	FMT	1	0
7	H	1009	FMT	1	0
9	K	3	GOL	1	0
7	H	928	FMT	1	0
7	H	951	FMT	2	0
7	H	902	FMT	2	0
7	H	1060	FMT	3	0
6	B	502	DEB	2	0
7	H	1040	FMT	2	0
7	H	943	FMT	1	0
6	Z	502	DEB	6	0
9	K	8[A]	GOL	1	0
9	K	1[B]	GOL	2	0
7	H	32	FMT	0	1
7	H	966	FMT	1	0
7	H	1024	FMT	2	0
7	H	19	FMT	4	0
7	H	1004	FMT	1	0
5	B	501	HEM	1	0
5	A	501	HEM	3	0
5	D	501	HEM	4	0
7	H	970	FMT	2	0

Continued on next page...

PRELIMINARY VALIDATION REPORT

Continued from previous page...

Mol	Chain	Res	Type	Clashes	Symm-Clashes
7	H	71	FMT	2	0
7	H	950	FMT	2	0

The following is a two-dimensional graphical depiction of Mogul quality analysis of bond lengths, bond angles, torsion angles, and ring geometry for all instances of the Ligand of Interest. In addition, ligands with molecular weight > 250 and outliers as shown on the validation Tables will also be included. For torsion angles, if less then 5% of the Mogul distribution of torsion angles is within 10 degrees of the torsion angle in question, then that torsion angle is considered an outlier. Any bond that is central to one or more torsion angles identified as an outlier by Mogul will be highlighted in the graph. For rings, the root-mean-square deviation (RMSD) between the ring in question and similar rings identified by Mogul is calculated over all ring torsion angles. If the average RMSD is greater than 60 degrees and the minimal RMSD between the ring in question and any Mogul-identified rings is also greater than 60 degrees, then that ring is considered an outlier. The outliers are highlighted in purple. The color gray indicates Mogul did not find sufficient equivalents in the CSD to analyse the geometry.

PRELIMINARY VALIDATION REPORT

Ligand DEB E 502

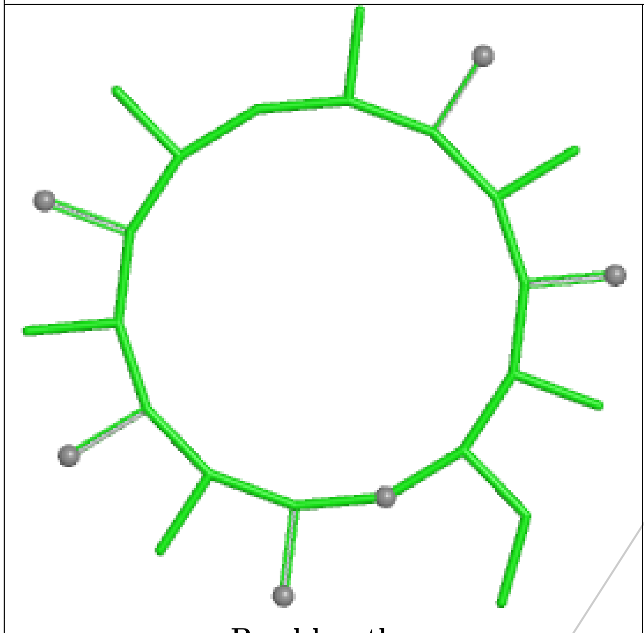

Bond lengths

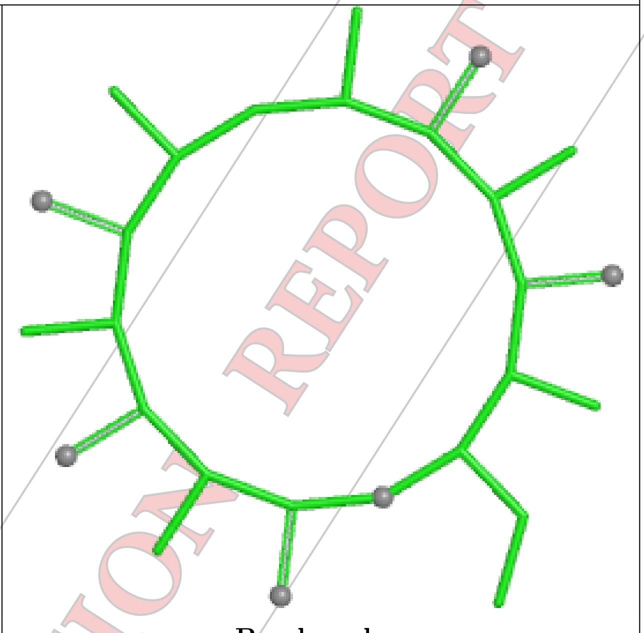

Bond angles

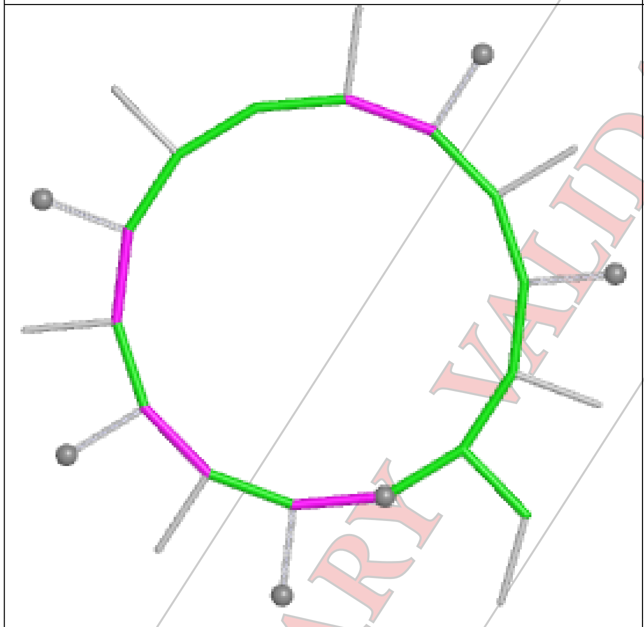

Torsions

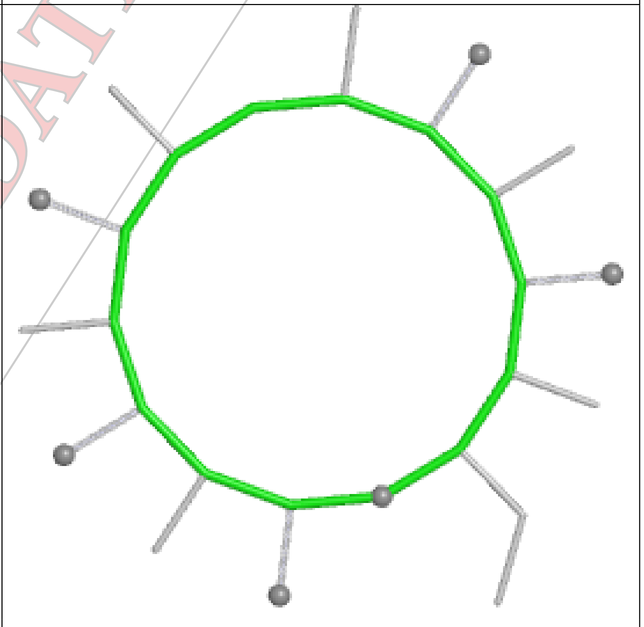

Rings

PRELIMINARY VALIDATION REPORT

Ligand HEM F 501

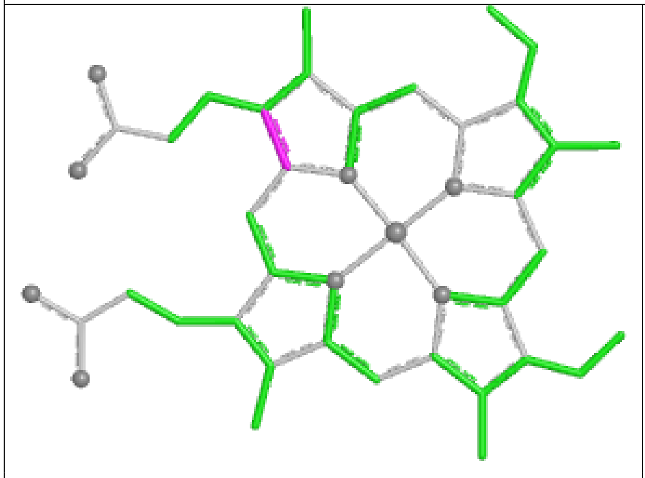

Bond lengths

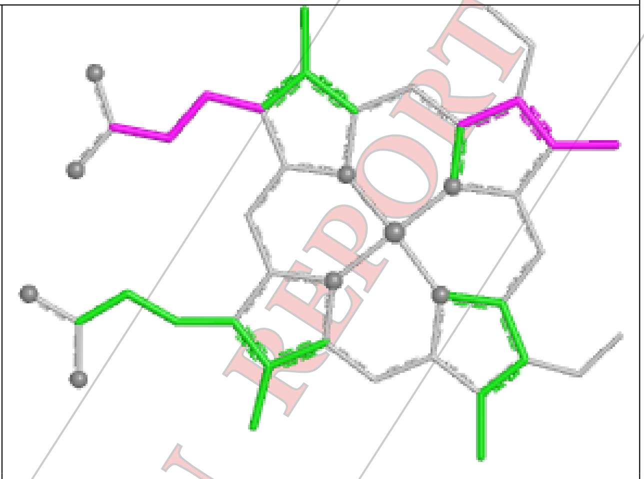

Bond angles

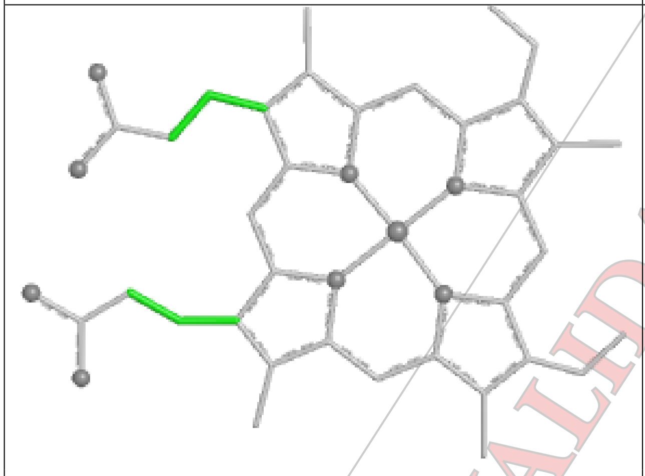

Torsions

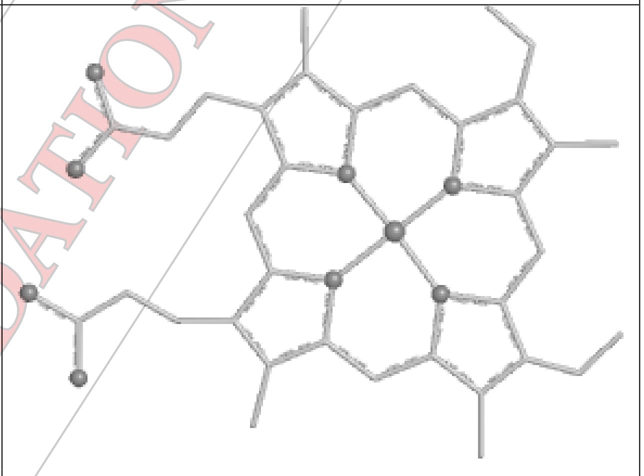

Rings

PRELIMINARY VALIDATION REPORT

Ligand HEM E 501	
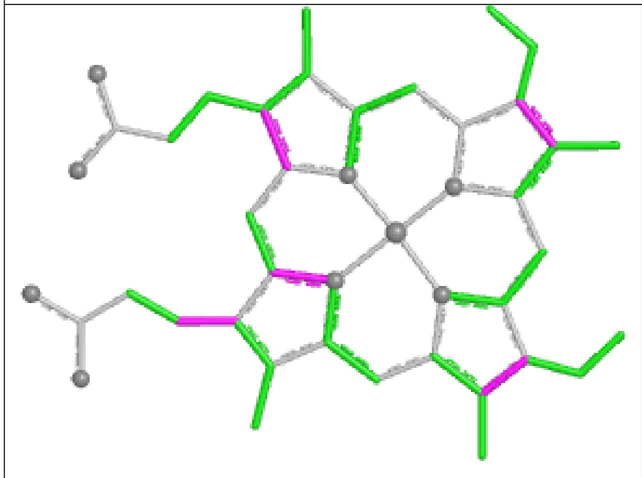 Bond lengths	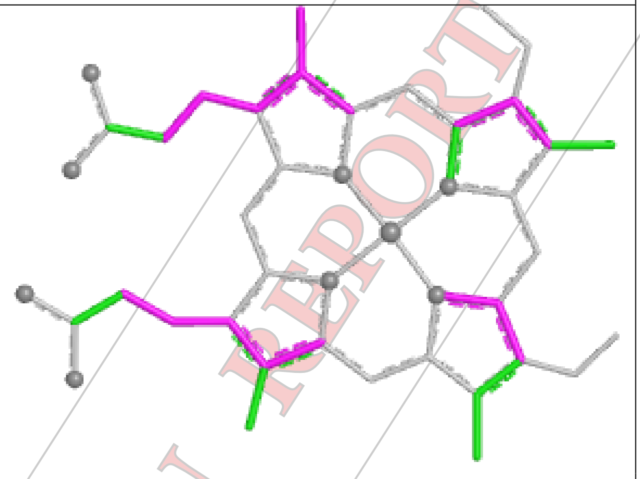 Bond angles
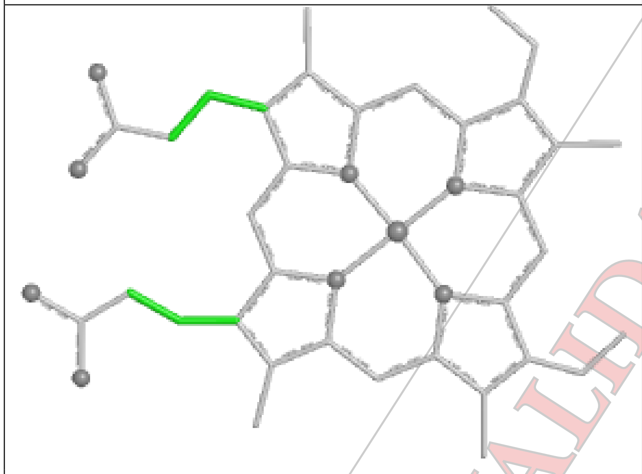 Torsions	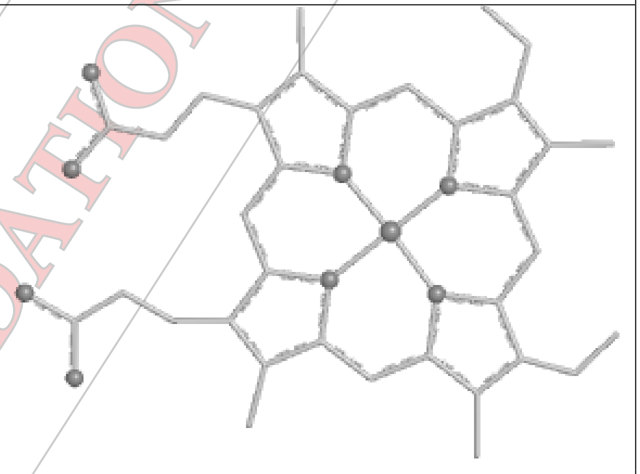 Rings

PRELIMINARY VALIDATION REPORT

Ligand DEB A 502

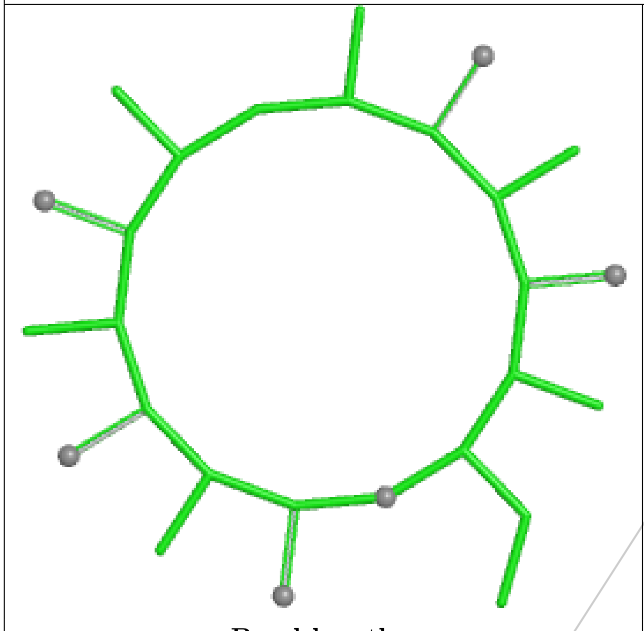

Bond lengths

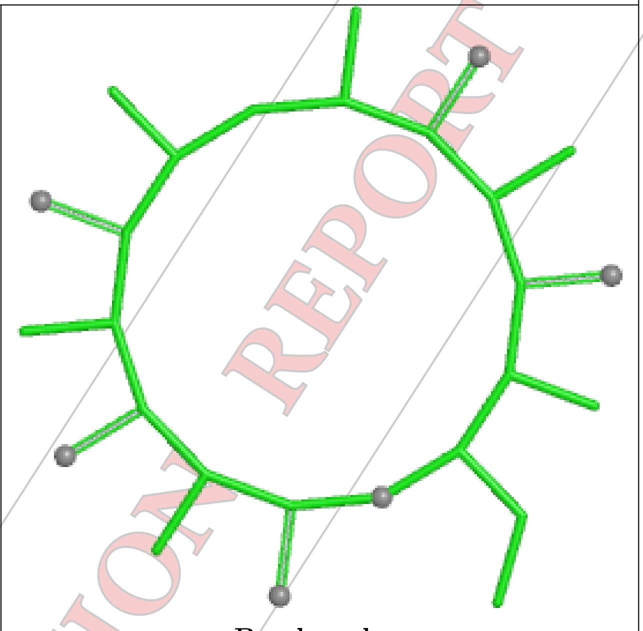

Bond angles

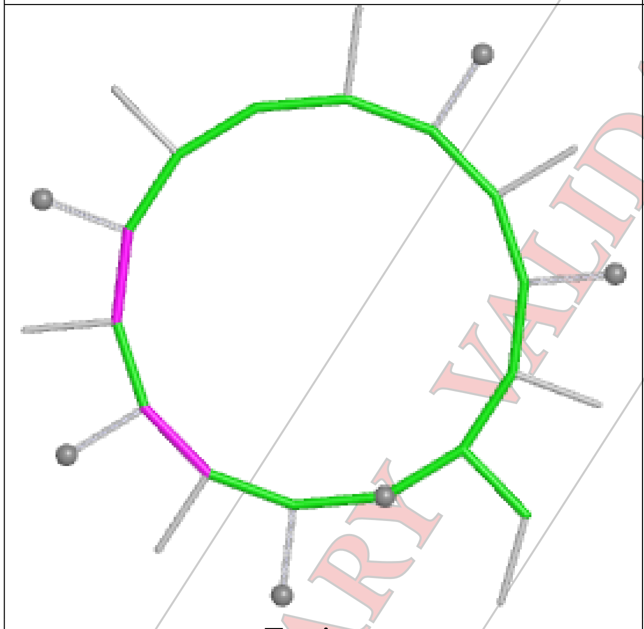

Torsions

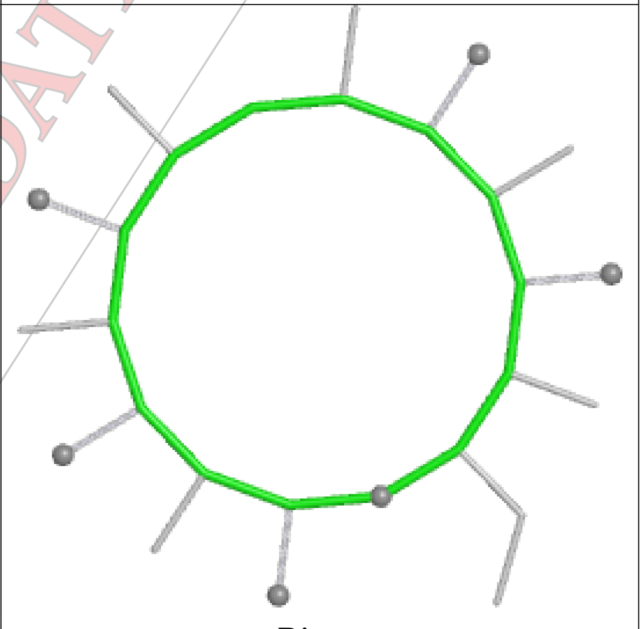

Rings

PRELIMINARY VALIDATION REPORT

Ligand HEM C 501

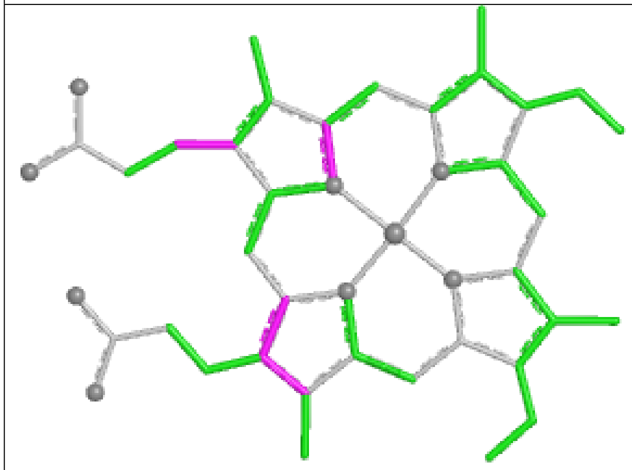

Bond lengths

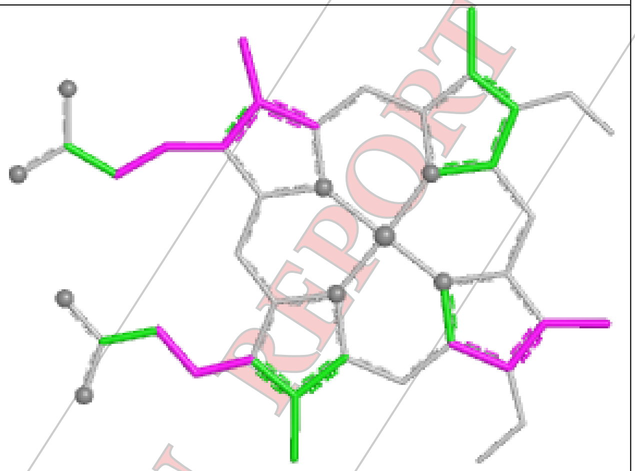

Bond angles

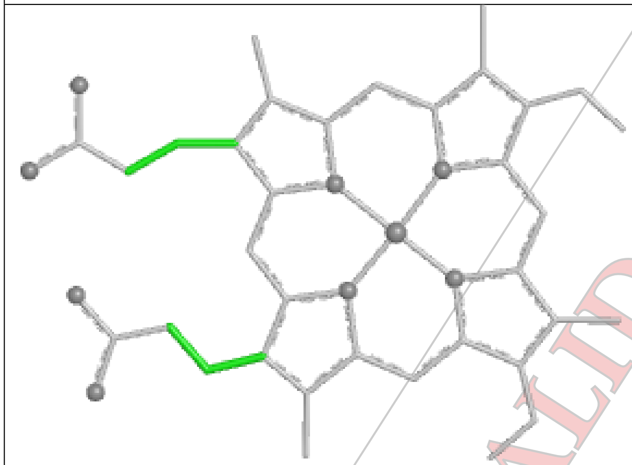

Torsions

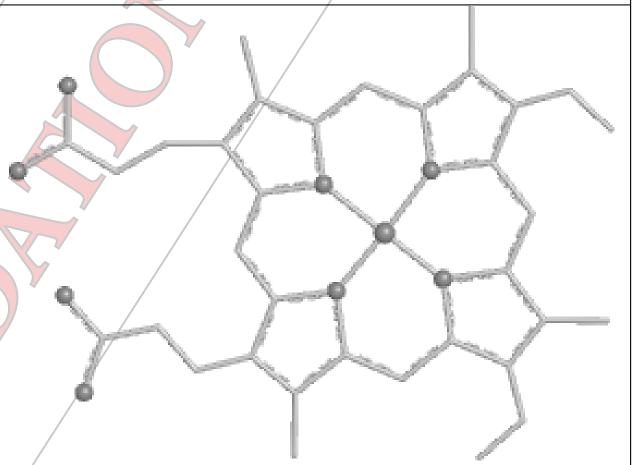

Rings

PRELIMINARY VALIDATION REPORT

Ligand DEB D 502

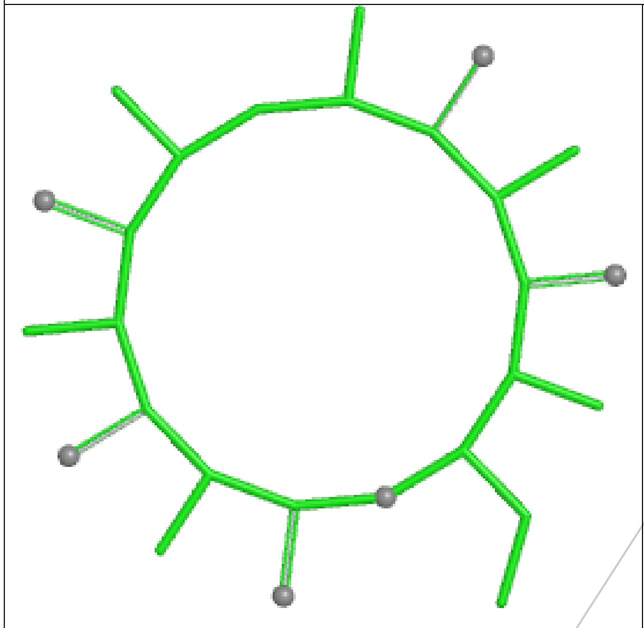

Bond lengths

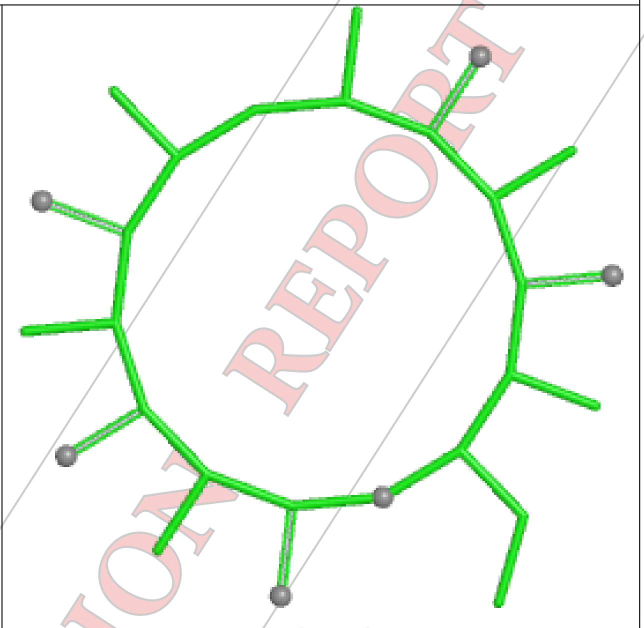

Bond angles

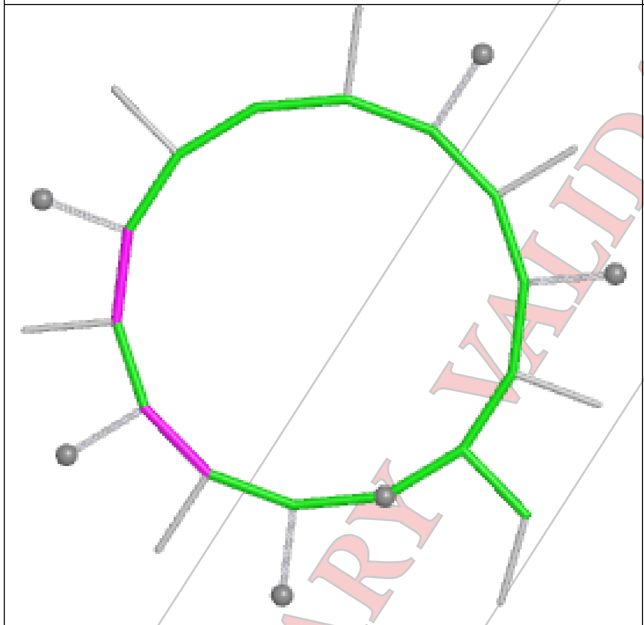

Torsions

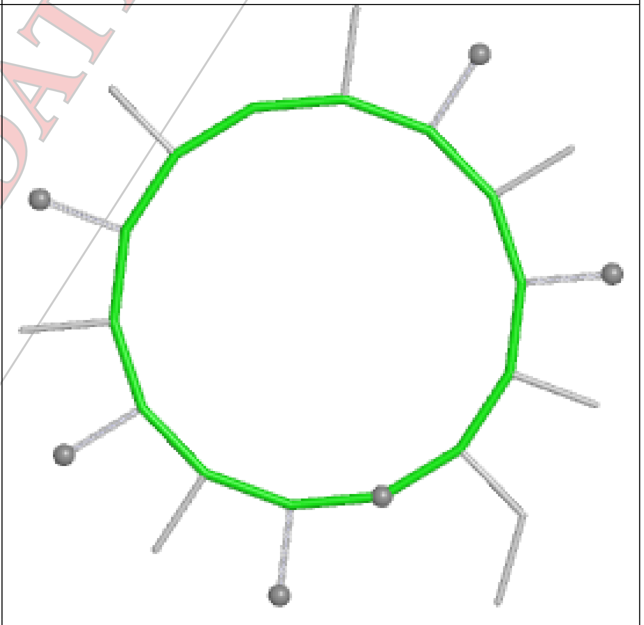

Rings

PRELIMINARY VALIDATION REPORT

Ligand DEB B 502	
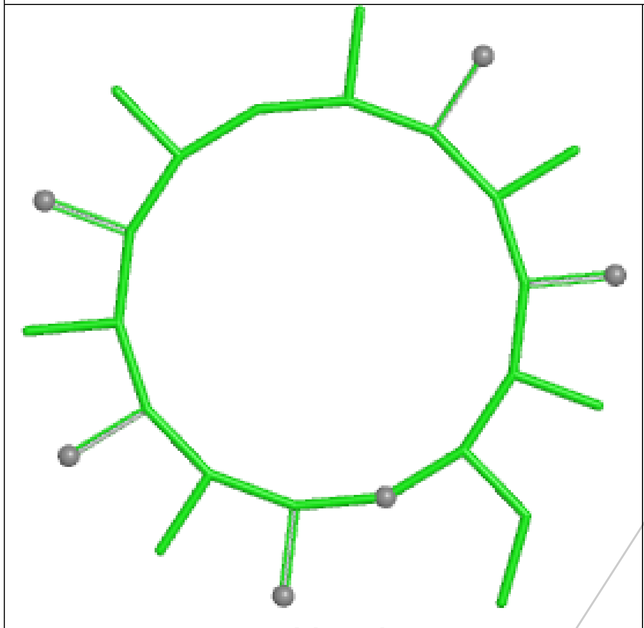 Bond lengths	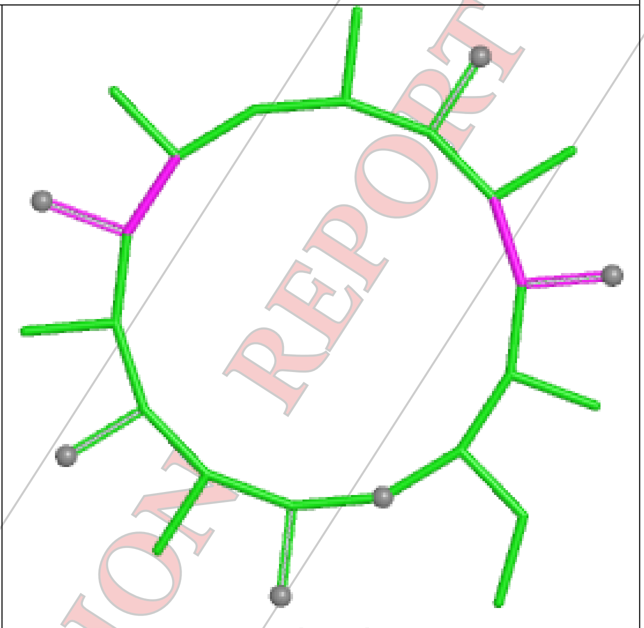 Bond angles
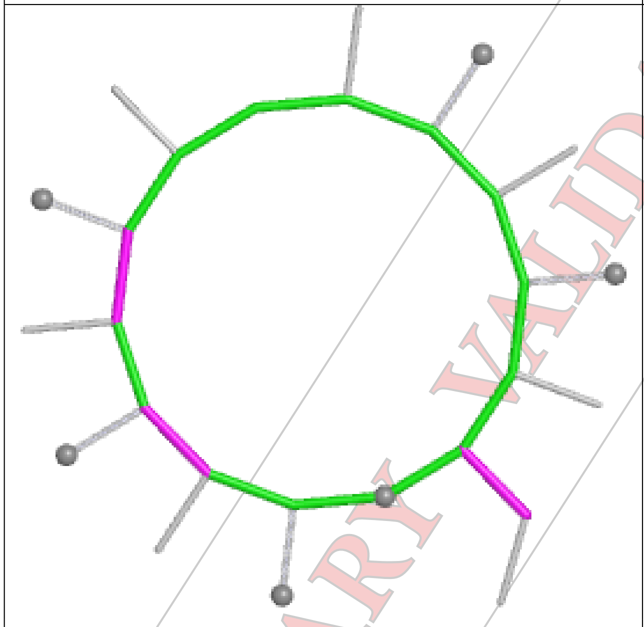 Torsions	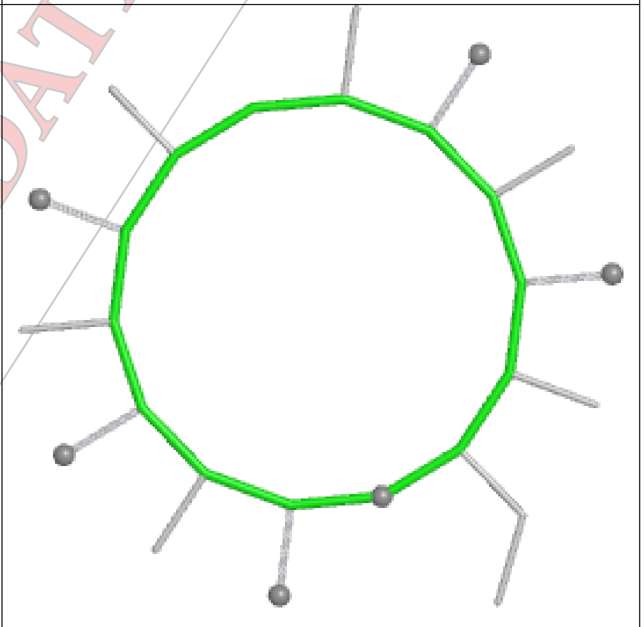 Rings

PRELIMINARY VALIDATION REPORT

Ligand DEB Z 502

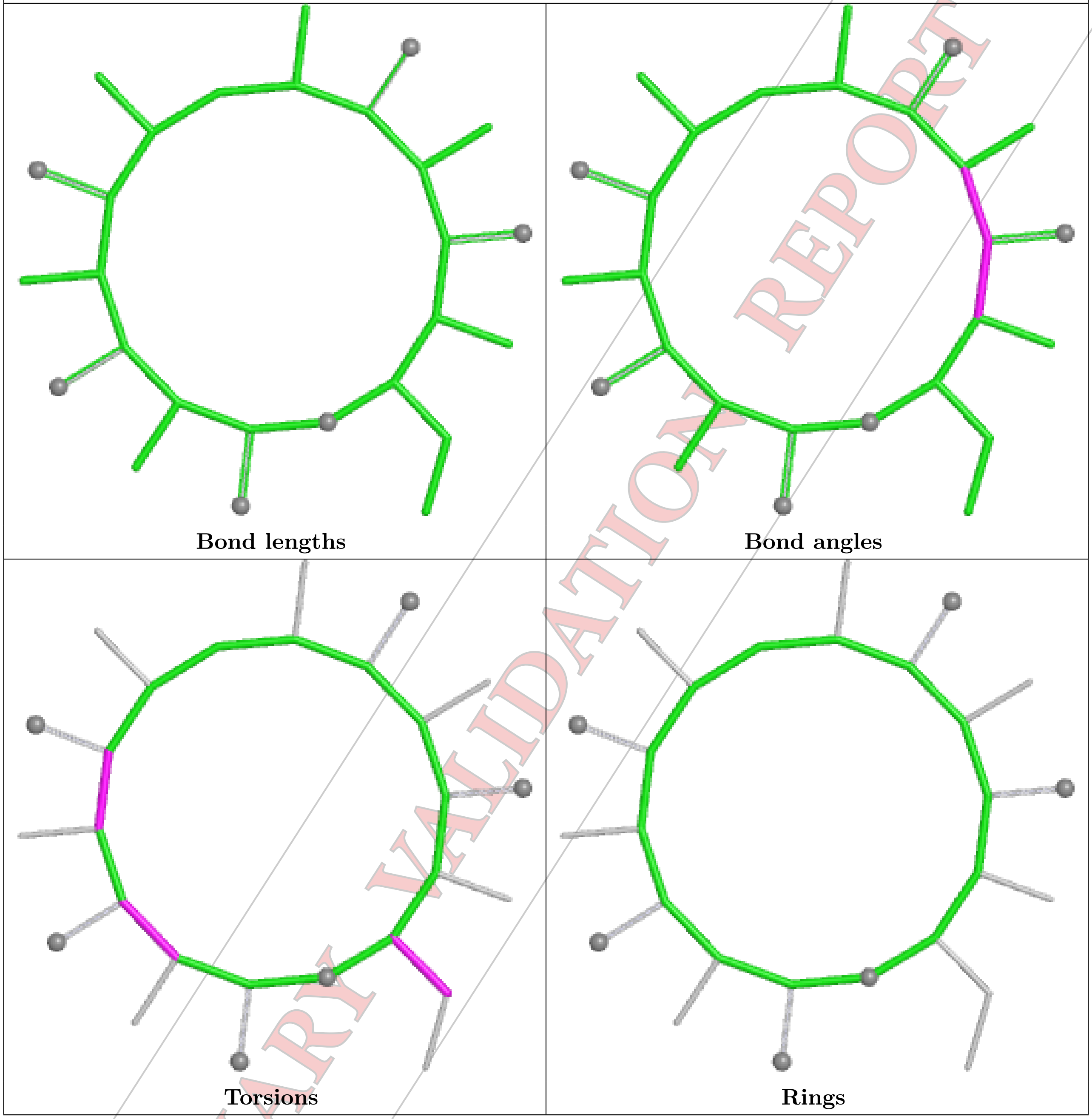

PRELIMINARY VALIDATION REPORT

Ligand HEM B 501

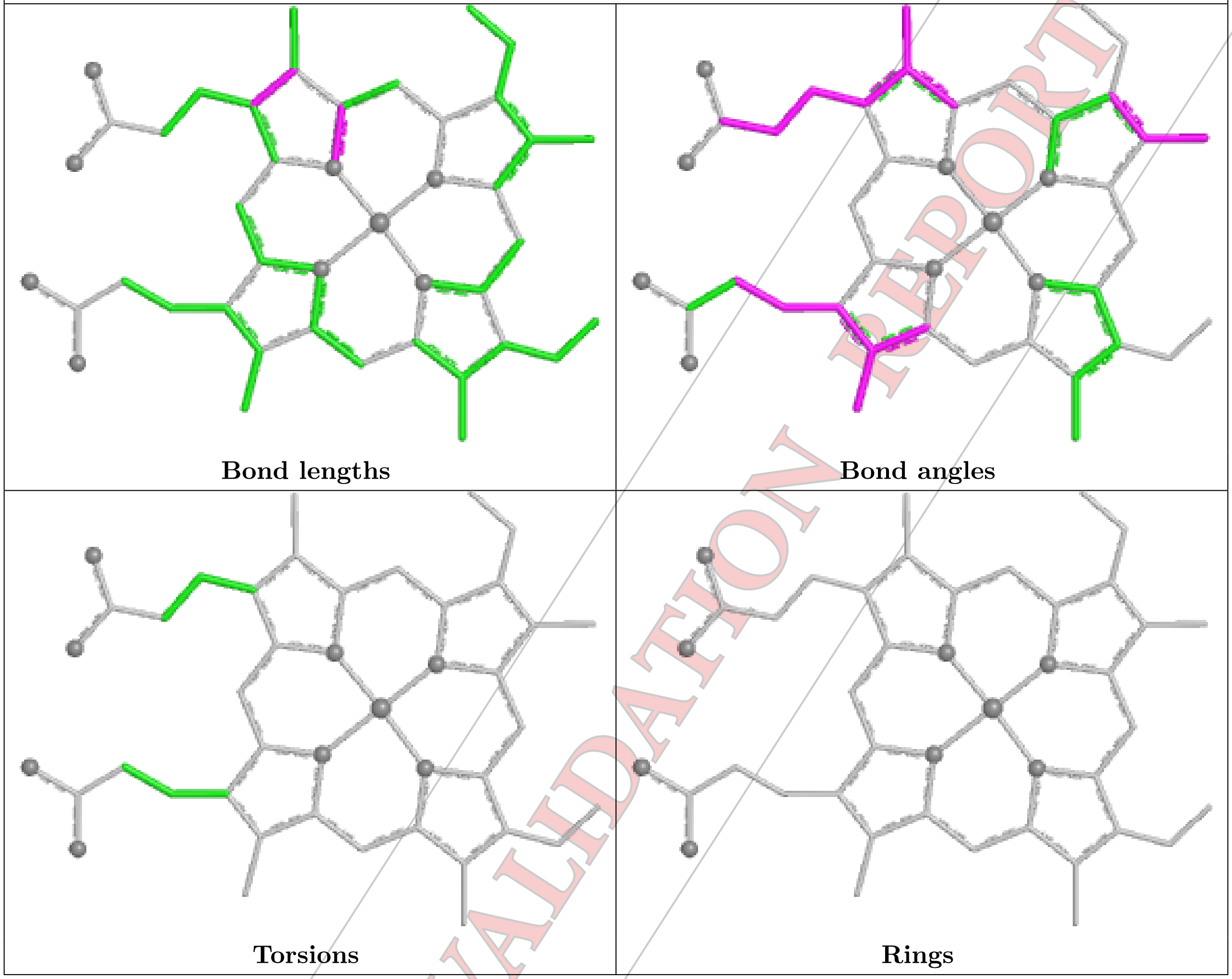

PRELIMINARY VALIDATION REPORT

Ligand DEB F 502

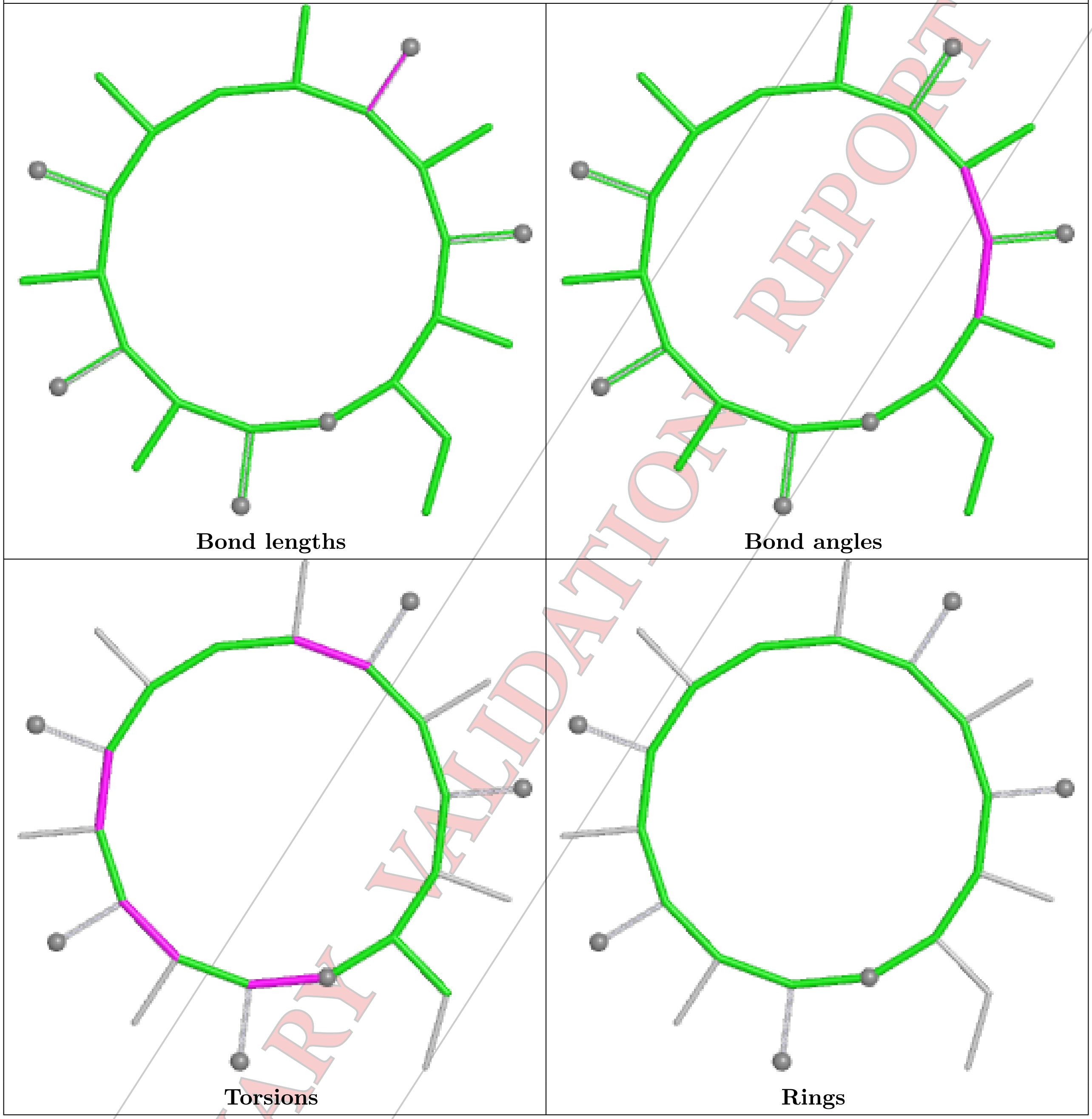

PRELIMINARY VALIDATION REPORT

Ligand HEM A 501	
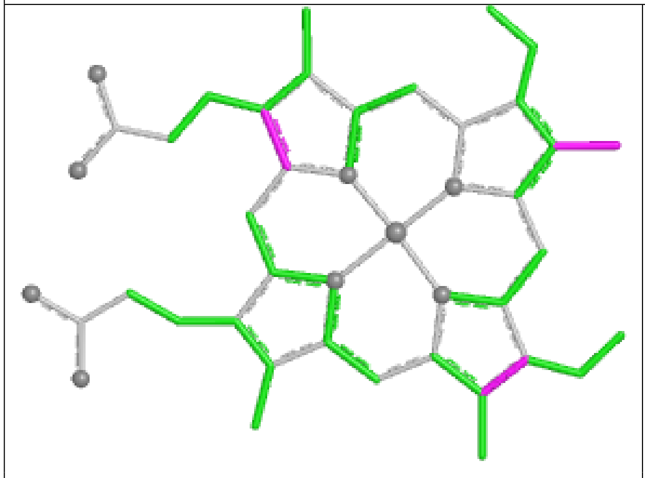 Bond lengths	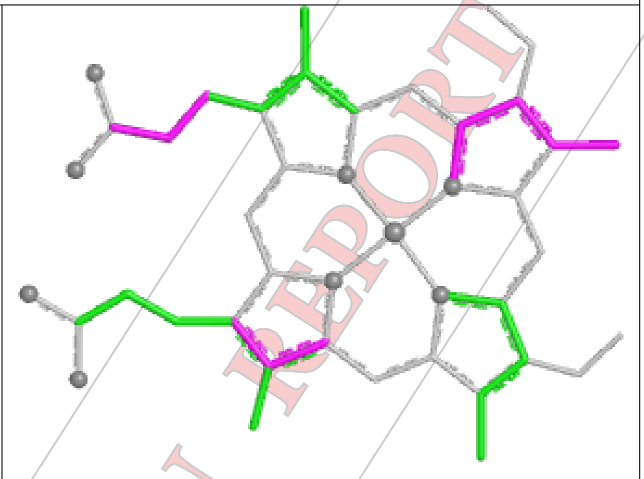 Bond angles
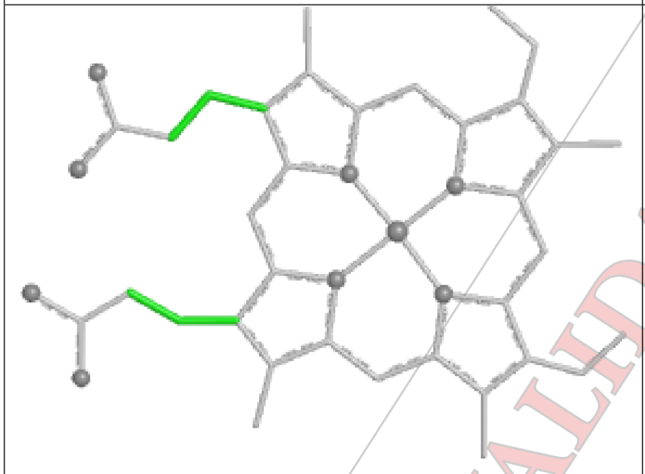 Torsions	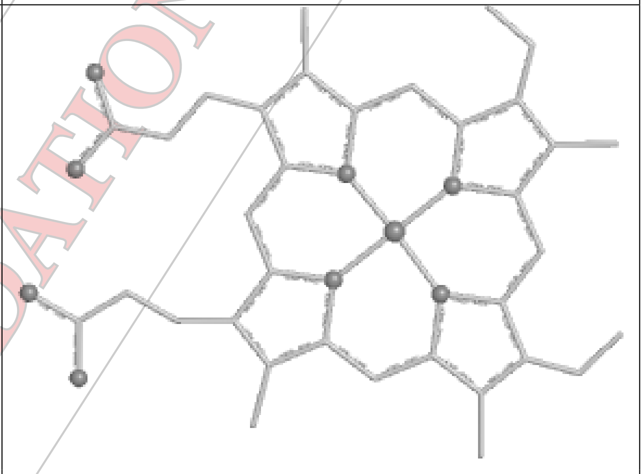 Rings

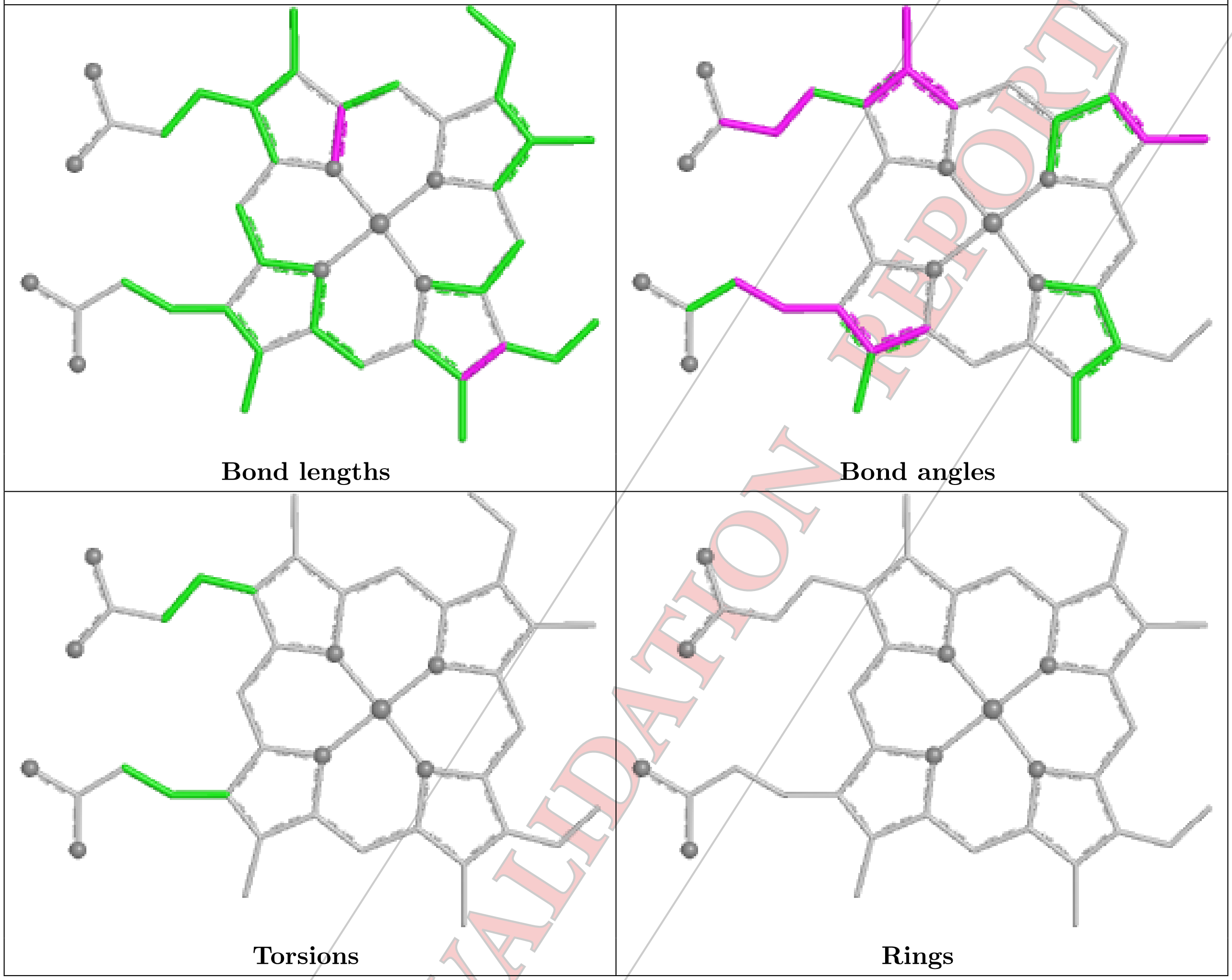

Ligand HEM D 501

Bond lengths | Bond angles | Torsions | Rings

5.7 Other polymers ⓘ

There are no such residues in this entry.

5.8 Polymer linkage issues ⓘ

There are no chain breaks in this entry.

PRELIMINARY VALIDATION REPORT

6 Fit of model and data (i)

6.1 Protein, DNA and RNA chains (i)

In the following table, the column labelled '#RSRZ> 2' contains the number (and percentage) of RSRZ outliers, followed by percent RSRZ outliers for the chain as percentile scores relative to all X-ray entries and entries of similar resolution. The OWAB column contains the minimum, median, 95th percentile and maximum values of the occupancy-weighted average B-factor per residue. The column labelled 'Q< 0.9' lists the number of (and percentage) of residues with an average occupancy less than 0.9.

Mol	Chain	Analysed	<RSRZ>	#RSRZ>2			OWAB(Å^2)	Q<0.9
1	A	396/396 (100%)	0.46	29 (7%)	15	18	31, 47, 70, 110	0
1	D	396/396 (100%)	0.62	40 (10%)	7	8	39, 57, 84, 153	0
1	F	396/396 (100%)	1.41	103 (26%)	0	0	42, 68, 107, 127	0
2	B	402/402 (100%)	0.38	29 (7%)	15	19	33, 49, 76, 136	0
3	C	408/408 (100%)	0.31	18 (4%)	34	39	34, 46, 71, 145	0
4	E	395/395 (100%)	0.95	85 (21%)	0	0	44, 63, 96, 166	0
All	All	2393/2393 (100%)	0.69	304 (12%)	3	4	31, 54, 95, 166	0

All (304) RSRZ outliers are listed below:

Mol	Chain	Res	Type	RSRZ
4	E	212	THR	11.6
1	F	405	VAL	11.1
1	F	262	THR	10.4
2	B	10[A]	PRO	9.3
1	F	127	LEU	9.0
1	F	277	VAL	8.9
4	E	211	PRO	8.8
2	B	209	ASP	8.6
3	C	9	THR	8.2
1	D	226	ASP	7.9
1	F	342	GLU	7.8
1	F	124	VAL	7.7
1	F	343	ARG	7.5
1	F	125	ARG	7.2
1	F	21	LEU	7.1
1	F	380	LEU	6.9
4	E	313	ALA	6.7
1	F	132	LEU	6.7
1	F	129	ASP	6.6

Continued on next page...

PRELIMINARY VALIDATION REPORT

Continued from previous page...

Mol	Chain	Res	Type	RSRZ
1	F	123	ARG	6.5
1	F	385	PRO	6.4
4	E	265	LYS	6.4
1	F	406	SER	6.2
1	F	141	PRO	6.1
1	F	36	ARG	6.1
1	F	377	PHE	6.1
1	F	140	SER	5.9
1	F	382	LEU	5.9
1	F	267	TYR	5.9
4	E	306	LEU	5.7
4	E	209	ASP	5.7
2	B	210	ALA	5.7
1	F	261	LEU	5.6
1	D	210	ALA	5.6
3	C	123	ARG	5.6
1	F	328	GLU	5.5
1	F	376	ARG	5.4
4	E	343	ARG	5.4
1	F	274	PRO	5.4
1	F	273	ASP	5.4
1	F	265	LYS	5.3
1	F	126	SER	5.3
1	F	270	LEU	5.2
1	F	327	ASP	5.2
2	B	211	PRO	5.1
1	F	340	HIS	5.0
1	D	209	ASP	5.0
1	F	142	ALA	5.0
1	F	27	LEU	4.9
2	B	6	THR	4.9
4	E	213	GLU	4.9
1	F	272	ALA	4.9
1	F	271	VAL	4.8
3	C	11	ALA	4.7
1	F	333	HIS	4.6
1	A	12	ASP	4.6
1	F	379	THR	4.6
1	D	227	ASP	4.5
1	F	332	ASP	4.5
1	F	407	TRP	4.5
1	D	228	HIS	4.5

Continued on next page...

PRELIMINARY VALIDATION REPORT

Continued from previous page...

Mol	Chain	Res	Type	RSRZ
1	F	269	SER	4.5
4	E	90	PRO	4.4
1	D	223	THR	4.4
1	D	208	ARG	4.4
1	F	337	LEU	4.4
4	E	271	VAL	4.4
4	E	308	THR	4.4
1	F	284[A]	MET	4.4
1	F	37	ASP	4.3
1	F	137	ALA	4.3
1	F	23	HIS	4.3
4	E	27	LEU	4.3
4	E	332	ASP	4.2
1	F	136	VAL	4.2
1	F	131	LEU	4.2
1	F	388	GLY	4.1
1	F	215	LEU	4.1
3	C	8	PRO	4.1
4	E	312	ARG	4.0
4	E	330	VAL	4.0
2	B	9[A]	THR	4.0
1	F	378	PRO	3.9
4	E	333	HIS	3.9
1	D	406	SER	3.8
4	E	310	THR	3.8
4	E	342	GLU	3.8
1	A	189	ILE	3.8
1	D	224	ASP	3.8
2	B	333	HIS	3.7
4	E	69	ARG	3.7
1	D	205	ALA	3.7
1	F	224	ASP	3.7
1	F	331	PHE	3.7
4	E	226	ASP	3.7
1	A	243	ILE	3.7
1	F	404	ILE	3.6
2	B	342[A]	GLU	3.6
4	E	67	ASP	3.6
4	E	305	GLU	3.6
4	E	309	VAL	3.6
2	B	7	GLY	3.6
4	E	307	SER	3.6

Continued on next page...

PRELIMINARY VALIDATION REPORT

Continued from previous page...

Mol	Chain	Res	Type	RSRZ
3	C	343	ARG	3.5
4	E	36	ARG	3.5
3	C	10	PRO	3.5
4	E	228	HIS	3.5
1	D	141	PRO	3.5
4	E	13	ALA	3.4
4	E	29	PRO	3.4
3	C	5	HIS	3.4
1	F	225	ASN	3.4
1	F	374	VAL	3.4
1	F	208	ARG	3.4
1	F	268	GLU	3.4
4	E	304	VAL	3.4
1	F	402	ARG	3.4
4	E	329	GLU	3.4
4	E	37	ASP	3.3
2	B	213	GLU	3.3
1	D	225	ASN	3.3
1	A	192	VAL	3.3
4	E	34	LEU	3.3
4	E	385	PRO	3.3
4	E	300	ALA	3.3
4	E	227	ASP	3.2
1	A	92[A]	TRP	3.2
1	F	329	GLU	3.2
2	B	357	ILE	3.2
2	B	228	HIS	3.2
1	F	400	LEU	3.2
1	F	339	PHE	3.2
4	E	24	ALA	3.2
1	F	307	SER	3.2
1	F	371	SER	3.2
1	F	29	PRO	3.1
1	A	44[A]	ARG	3.1
4	E	301	THR	3.1
4	E	388	GLY	3.1
1	D	184	LEU	3.1
1	A	13	ALA	3.1
4	E	328	GLU	3.1
4	E	223	THR	3.1
4	E	331	PHE	3.1
4	E	60	ASP	3.0

Continued on next page...

PRELIMINARY VALIDATION REPORT

Continued from previous page...

Mol	Chain	Res	Type	RSRZ
1	D	220	ALA	3.0
1	F	384	GLU	3.0
1	D	357	ILE	3.0
1	D	267	TYR	3.0
1	F	130	SER	3.0
1	A	209	ASP	3.0
4	E	229	LEU	3.0
1	F	375	ARG	2.9
4	E	189	ILE	2.9
2	B	227	ASP	2.9
4	E	23	HIS	2.9
4	E	338	ASP	2.9
4	E	335	ASP	2.9
1	F	330	VAL	2.9
1	A	177	ALA	2.9
4	E	33	GLU	2.9
1	F	326	ARG	2.9
4	E	327	ASP	2.9
1	F	165	LEU	2.9
1	F	280	ALA	2.9
4	E	285	LEU	2.8
1	A	224	ASP	2.8
1	D	333	HIS	2.8
4	E	340	HIS	2.8
4	E	311	VAL	2.8
1	F	383	ALA	2.8
1	A	85	PRO	2.8
1	F	387	ALA	2.7
4	E	225	ASN	2.7
1	F	128	VAL	2.7
1	A	180[A]	SER	2.7
1	A	179	LEU	2.7
4	E	64	VAL	2.7
1	D	332	ASP	2.7
4	E	140	SER	2.7
4	E	406	SER	2.7
1	D	263	GLU	2.7
1	D	137	ALA	2.7
1	F	22	PRO	2.7
4	E	274	PRO	2.7
4	E	21	LEU	2.7
1	F	386	VAL	2.6

Continued on next page...

PRELIMINARY VALIDATION REPORT

Continued from previous page...

Mol	Chain	Res	Type	RSRZ
4	E	268	GLU	2.6
1	D	27	LEU	2.6
1	F	139	GLY	2.6
1	F	346	HIS	2.6
4	E	383	ALA	2.6
1	F	335	ASP	2.6
1	D	36	ARG	2.6
1	F	287	TYR	2.6
1	F	57	ARG	2.6
3	C	4[A]	THR	2.6
1	D	385	PRO	2.6
4	E	43	VAL	2.6
1	F	263	GLU	2.6
1	A	94	LEU	2.6
1	F	389	LEU	2.6
2	B	265	LYS	2.5
2	B	291	VAL	2.5
1	F	308	THR	2.5
1	F	364	LEU	2.5
3	C	291	VAL	2.5
1	A	379	THR	2.5
4	E	39	PRO	2.5
4	E	261	LEU	2.5
4	E	136	VAL	2.5
3	C	14	VAL	2.5
4	E	208	ARG	2.5
4	E	303	ASP	2.5
3	C	84	PHE	2.5
4	E	272	ALA	2.5
1	F	58	MET	2.4
1	F	122	PRO	2.4
1	A	236[A]	ASN	2.4
4	E	302	GLU	2.4
1	A	187	ALA	2.4
4	E	25	LEU	2.4
1	F	38	GLU	2.4
1	D	359	ALA	2.4
4	E	339	PHE	2.4
2	B	221	LEU	2.4
4	E	337	LEU	2.4
4	E	387	ALA	2.4
1	F	192	VAL	2.4

Continued on next page...

PRELIMINARY VALIDATION REPORT

Continued from previous page...

Mol	Chain	Res	Type	RSRZ
1	D	24	ALA	2.4
1	A	182	THR	2.4
1	A	47	TYR	2.4
1	D	358	GLY	2.4
1	D	380	LEU	2.4
1	A	16	ALA	2.4
1	A	181	SER	2.4
1	D	189	ILE	2.4
4	E	28	ASP	2.4
1	F	370	LEU	2.3
1	F	121	ARG	2.3
4	E	334	ALA	2.3
4	E	287	TYR	2.3
1	D	94	LEU	2.3
3	C	184	LEU	2.3
4	E	42	ARG	2.3
1	D	231	LYS	2.3
1	F	347	ILE	2.3
2	B	204	VAL	2.3
1	A	267	TYR	2.3
1	D	342	GLU	2.3
3	C	189	ILE	2.3
4	E	269	SER	2.3
1	F	147	PHE	2.3
1	F	336	GLU	2.3
4	E	207	ARG	2.3
4	E	166	GLU	2.3
1	D	337[A]	LEU	2.2
1	F	276	LEU	2.2
3	C	396	LEU	2.2
1	A	265	LYS	2.2
2	B	406	SER	2.2
1	D	257	VAL	2.2
1	D	386	VAL	2.2
2	B	225	ASN	2.2
4	E	106[A]	ARG	2.2
3	C	342	GLU	2.2
2	B	224	ASP	2.2
1	F	264	ARG	2.2
4	E	382	LEU	2.1
1	F	213	GLU	2.1
2	B	271	VAL	2.1

Continued on next page...

PRELIMINARY VALIDATION REPORT

Continued from previous page...

Mol	Chain	Res	Type	RSRZ
3	C	394	GLY	2.1
1	A	81	PRO	2.1
1	A	141	PRO	2.1
1	D	330	VAL	2.1
4	E	299	VAL	2.1
1	D	334	ALA	2.1
1	F	59	SER	2.1
2	B	222	ALA	2.1
1	F	279	ALA	2.1
1	F	138	HIS	2.1
2	B	136	VAL	2.1
1	A	210	ALA	2.1
1	F	134	ASP	2.1
1	D	329	GLU	2.1
2	B	159	GLU	2.1
3	C	7	GLY	2.1
1	D	241	LEU	2.1
1	A	184	LEU	2.0
1	F	26	ASP	2.0
2	B	332	ASP	2.0
2	B	335	ASP	2.0
2	B	8[A]	PRO	2.0
1	A	86	THR	2.0
1	D	192	VAL	2.0
4	E	210	ALA	2.0
4	E	314	GLY	2.0
2	B	285	LEU	2.0
4	E	336	GLU	2.0
2	B	350	GLY	2.0
1	F	209	ASP	2.0
3	C	333	HIS	2.0
1	A	262	THR	2.0
1	D	179	LEU	2.0

6.2 Non-standard residues in protein, DNA, RNA chains (i)

There are no non-standard protein/DNA/RNA residues in this entry.

6.3 Carbohydrates (i)

There are no monosaccharides in this entry.

PRELIMINARY VALIDATION REPORT

6.4 Ligands ⓘ

In the following table, the Atoms column lists the number of modelled atoms in the group and the number defined in the chemical component dictionary. The B-factors column lists the minimum, median, 95th percentile and maximum values of B factors of atoms in the group. The column labelled 'Q< 0.9' lists the number of atoms with occupancy less than 0.9.

Mol	Type	Chain	Res	Atoms	RSCC	RSR	B-factors(Å^2)	Q<0.9
7	FMT	H	920	3/?	-0.12	0.37	98,98,105,106	0
7	FMT	H	6	3/?	0.25	0.28	94,94,106,108	0
7	FMT	H	62	3/?	0.28	0.44	86,86,95,100	0
7	FMT	H	929	3/?	0.32	0.43	88,88,95,108	0
7	FMT	H	976	3/?	0.33	0.47	89,89,102,105	0
7	FMT	H	905	3/?	0.34	0.35	95,95,108,112	0
7	FMT	H	909	3/?	0.35	0.25	90,90,95,99	0
7	FMT	H	915	3/?	0.37	0.33	66,66,85,86	0
7	FMT	H	972	3/?	0.40	0.36	73,73,82,94	0
7	FMT	H	32	3/?	0.40	0.23	85,85,106,110	0
7	FMT	H	1036	3/?	0.40	0.65	75,75,80,94	0
7	FMT	H	1062	3/?	0.41	0.29	91,91,92,97	0
7	FMT	H	7	3/?	0.42	0.37	83,83,83,88	0
7	FMT	H	1005	3/?	0.44	0.47	86,86,94,101	0
7	FMT	H	96	3/?	0.45	0.15	94,94,104,106	0
7	FMT	H	1020	3/?	0.45	0.52	94,94,101,105	0
7	FMT	H	14	3/?	0.46	0.17	76,76,87,92	0
7	FMT	H	937	3/?	0.47	0.26	78,78,91,94	0
7	FMT	H	948	3/?	0.49	0.36	99,99,102,106	0
7	FMT	H	966	3/?	0.49	0.22	88,88,88,101	0
7	FMT	H	1030	3/?	0.50	0.16	92,92,107,121	0
7	FMT	H	997	3/?	0.50	0.30	78,78,93,93	0
7	FMT	H	1023	3/?	0.50	0.16	106,106,107,108	0
7	FMT	H	938	3/?	0.51	0.22	78,78,80,96	0
7	FMT	H	65	3/?	0.51	0.33	84,84,95,97	0
7	FMT	H	1004	3/?	0.51	0.55	73,73,88,91	0
9	GOL	K	11	6/?	0.53	0.53	76,93,96,109	0
7	FMT	H	1033	3/?	0.55	0.33	81,81,92,92	0
7	FMT	H	66	3/?	0.56	0.24	64,64,65,76	0
7	FMT	H	946	3/?	0.56	0.23	76,76,77,92	0
7	FMT	H	968	3/?	0.56	0.28	75,75,80,81	0
7	FMT	H	940	3/?	0.57	0.54	86,86,93,103	0
9	GOL	K	3	6/?	0.58	0.27	70,88,89,89	0
7	FMT	H	1028	3/?	0.58	0.19	71,71,92,93	0
7	FMT	H	1025	3/?	0.59	0.26	74,74,87,103	0
7	FMT	H	908	3/?	0.59	0.34	78,78,87,100	0

Continued on next page...

PRELIMINARY VALIDATION REPORT

Continued from previous page...

Mol	Type	Chain	Res	Atoms	RSCC	RSR	B-factors(Å^2)	Q<0.9
7	FMT	H	1040	3/?	0.60	0.39	71,71,74,83	0
7	FMT	H	41	3/?	0.60	0.31	50,50,61,75	0
7	FMT	H	928	3/?	0.60	0.46	85,85,99,101	0
7	FMT	H	1021	3/?	0.60	0.41	78,78,83,97	0
7	FMT	H	72	3/?	0.61	0.45	59,59,73,85	0
7	FMT	H	917	3/?	0.62	0.46	68,68,84,86	0
7	FMT	H	1064	3/?	0.62	0.38	72,72,77,79	0
7	FMT	H	998	3/?	0.62	0.46	83,83,95,112	0
7	FMT	H	1006	3/?	0.62	0.17	73,73,73,81	0
7	FMT	H	995	3/?	0.63	0.19	102,102,102,106	0
7	FMT	H	944	3/?	0.65	0.18	61,61,74,90	0
7	FMT	H	969	3/?	0.67	0.20	79,79,91,92	0
9	GOL	K	5	6/?	0.67	0.41	58,72,83,91	0
7	FMT	H	911	3/?	0.67	0.29	79,79,90,95	0
7	FMT	H	95	3/?	0.68	0.32	63,63,85,89	0
7	FMT	H	925	3/?	0.69	0.31	89,89,99,101	0
7	FMT	H	912	3/?	0.69	0.21	85,85,94,101	0
7	FMT	H	1069	3/?	0.69	0.35	86,86,87,89	0
7	FMT	H	71	3/?	0.69	0.16	68,68,73,84	0
7	FMT	H	961	3/?	0.69	0.24	79,79,85,87	0
7	FMT	H	1058	3/?	0.69	0.22	65,65,73,78	0
7	FMT	H	56	3/?	0.71	0.33	86,86,87,94	0
7	FMT	H	933	3/?	0.71	0.17	93,93,96,100	0
7	FMT	H	1019	3/?	0.72	0.23	91,91,93,97	0
7	FMT	H	19	3/?	0.72	0.25	64,64,78,79	0
7	FMT	H	1044	3/?	0.72	0.14	69,69,78,78	0
7	FMT	H	980	3/?	0.72	0.31	92,92,98,106	0
7	FMT	H	984	3/?	0.72	0.51	95,95,101,103	0
7	FMT	H	1009	3/?	0.73	0.19	73,73,88,93	0
7	FMT	H	1068	3/?	0.73	0.23	93,93,95,99	0
7	FMT	H	942	3/?	0.74	0.17	64,64,78,89	0
7	FMT	H	67	3/?	0.74	0.23	79,79,81,83	0
7	FMT	H	57	3/?	0.74	0.44	86,86,90,92	0
7	FMT	H	1045	3/?	0.74	0.40	86,86,91,92	0
7	FMT	H	932	3/?	0.75	0.19	75,75,83,83	0
7	FMT	H	1038	3/?	0.75	0.18	66,66,72,84	0
7	FMT	H	971	3/?	0.75	0.19	60,60,64,84	0
7	FMT	H	935	3/?	0.75	0.23	69,69,70,86	0
7	FMT	H	1065	3/?	0.75	0.21	83,83,87,101	0
7	FMT	H	29	3/?	0.76	0.15	73,73,78,86	0
7	FMT	H	970	3/?	0.76	0.16	78,78,83,96	0
9	GOL	K	4[A]	6/?	0.76	0.38	45,57,59,60	6

Continued on next page...

PRELIMINARY VALIDATION REPORT

Continued from previous page...

Mol	Type	Chain	Res	Atoms	RSCC	RSR	B-factors(Å^2)	Q<0.9
9	GOL	K	4[B]	6/?	0.76	0.38	121,125,127,128	6
7	FMT	H	941	3/?	0.76	0.26	80,80,89,93	0
7	FMT	H	960	3/?	0.76	0.39	68,68,71,80	0
7	FMT	H	74	3/?	0.77	0.34	91,91,93,96	0
7	FMT	H	955	3/?	0.77	0.14	81,81,86,94	0
7	FMT	H	982	3/?	0.78	0.17	74,74,81,89	0
7	FMT	H	979	3/?	0.78	0.14	92,92,95,97	0
7	FMT	H	12	3/?	0.78	0.23	61,61,71,83	0
7	FMT	H	1008	3/?	0.79	0.23	69,69,71,89	0
7	FMT	H	906	3/?	0.79	0.17	66,66,67,79	0
9	GOL	K	13	6/?	0.79	0.28	46,63,70,80	6
9	GOL	K	9	6/?	0.80	0.27	68,80,81,83	6
7	FMT	H	914	3/?	0.81	0.45	90,90,95,98	0
7	FMT	H	950	3/?	0.81	0.73	73,73,74,77	0
7	FMT	H	70	3/?	0.81	0.17	55,55,59,74	0
7	FMT	H	33	3/?	0.81	0.25	67,67,87,87	0
7	FMT	H	1001	3/?	0.81	0.37	72,72,79,82	0
7	FMT	H	934	3/?	0.81	0.40	82,82,83,89	0
7	FMT	H	1003	3/?	0.82	0.25	64,64,72,79	0
7	FMT	H	996	3/?	0.82	0.29	84,84,87,100	0
7	FMT	H	904	3/?	0.82	0.21	62,62,75,80	0
7	FMT	H	943	3/?	0.82	0.34	74,74,80,84	0
7	FMT	H	981	3/?	0.82	0.22	75,75,79,90	0
9	GOL	K	1[A]	6/?	0.82	0.27	53,57,60,61	6
9	GOL	K	1[B]	6/?	0.82	0.27	26,40,43,47	6
6	DEB	F	502	27/?	0.83	0.19	56,64,74,83	0
7	FMT	H	21	3/?	0.83	0.16	77,77,93,94	0
7	FMT	H	901	3/?	0.83	0.16	80,80,85,87	0
7	FMT	H	921	3/?	0.83	0.16	82,82,90,98	0
9	GOL	K	7[A]	6/?	0.83	0.28	36,39,43,45	6
9	GOL	K	7[B]	6/?	0.83	0.28	66,72,80,83	6
7	FMT	H	923	3/?	0.83	0.10	86,86,105,106	0
7	FMT	H	601	3/?	0.83	0.33	89,89,93,96	0
7	FMT	H	1014	3/?	0.83	0.17	84,84,86,88	0
7	FMT	H	1018	3/?	0.84	0.21	84,84,90,92	0
7	FMT	H	1024	3/?	0.85	0.15	87,87,91,93	0
7	FMT	H	1015	3/?	0.85	0.29	85,85,86,90	0
9	GOL	K	8[A]	6/?	0.85	0.39	32,46,51,56	6
9	GOL	K	8[B]	6/?	0.85	0.39	32,40,45,45	6
7	FMT	H	1046	3/?	0.85	0.34	50,50,51,73	3
7	FMT	H	10	3/?	0.85	0.35	70,70,80,90	0
7	FMT	H	13	3/?	0.85	0.21	58,58,60,64	0

Continued on next page...

PRELIMINARY VALIDATION REPORT

Continued from previous page...

Mol	Type	Chain	Res	Atoms	RSCC	RSR	B-factors(Å^2)	Q<0.9
7	FMT	H	918	3/?	0.86	0.29	61,61,70,75	0
7	FMT	H	931	3/?	0.86	0.25	66,66,77,79	0
7	FMT	H	919	3/?	0.86	0.20	69,69,75,81	0
7	FMT	H	965	3/?	0.86	0.39	85,85,88,106	0
7	FMT	H	58	3/?	0.86	0.12	85,85,87,89	0
7	FMT	H	1060	3/?	0.86	0.11	59,59,83,96	0
7	FMT	H	1007	3/?	0.86	0.15	74,74,89,93	0
6	DEB	Z	502	27/?	0.86	0.16	30,37,50,54	0
7	FMT	H	64	3/?	0.86	0.19	75,75,84,84	0
7	FMT	H	947	3/?	0.86	0.27	76,76,81,84	0
7	FMT	H	15	3/?	0.86	0.27	57,57,64,72	0
7	FMT	H	27	3/?	0.86	0.22	73,73,74,80	0
6	DEB	E	502	27/?	0.87	0.15	47,53,61,74	0
7	FMT	H	1047	3/?	0.87	0.08	84,84,86,89	0
7	FMT	H	8	3/?	0.87	0.23	68,68,72,75	0
6	DEB	D	502	27/?	0.87	0.17	32,42,46,47	0
8	NA	I	6	1/?	0.87	0.30	63,63,63,63	0
7	FMT	H	11	3/?	0.87	0.14	65,65,75,85	0
7	FMT	H	926	3/?	0.88	0.19	76,76,80,87	0
7	FMT	H	986	3/?	0.88	0.28	78,78,79,80	0
7	FMT	H	94	3/?	0.88	0.15	63,63,66,68	0
5	HEM	F	501	43/?	0.89	0.17	47,54,64,92	0
8	NA	I	10	1/?	0.89	0.12	56,56,56,56	0
7	FMT	H	939	3/?	0.89	0.17	74,74,76,84	0
7	FMT	H	913	3/?	0.89	0.13	83,83,88,91	0
8	NA	I	4	1/?	0.89	0.20	56,56,56,56	0
7	FMT	H	20	3/?	0.90	0.32	63,63,64,82	0
7	FMT	H	1053	3/?	0.91	0.30	71,71,72,93	0
7	FMT	H	910	3/?	0.91	0.91	82,82,94,98	0
6	DEB	A	502	27/?	0.91	0.21	30,37,50,54	0
7	FMT	H	40	3/?	0.91	0.16	59,59,63,68	0
7	FMT	H	1010	3/?	0.92	0.27	52,52,83,96	0
5	HEM	E	501	43/?	0.92	0.13	39,44,53,66	0
8	NA	I	5	1/?	0.93	0.22	63,63,63,63	0
7	FMT	H	945	3/?	0.93	0.19	62,62,79,96	0
6	DEB	B	502	27/?	0.93	0.18	33,40,51,59	0
7	FMT	H	93	3/?	0.93	0.35	65,65,70,79	0
7	FMT	H	1048	3/?	0.94	0.18	67,67,71,76	0
7	FMT	H	1061	3/?	0.94	0.28	50,50,71,78	0
7	FMT	H	902	3/?	0.94	0.15	46,46,67,70	0
7	FMT	H	922	3/?	0.94	0.17	71,71,73,91	0
5	HEM	D	501	43/?	0.95	0.19	25,36,49,62	0

Continued on next page...

PRELIMINARY VALIDATION REPORT

Continued from previous page...

Mol	Type	Chain	Res	Atoms	RSCC	RSR	B-factors($Å^2$)	Q<0.9
7	FMT	H	951	3/?	0.95	0.12	58,58,68,70	0
7	FMT	H	31	3/?	0.95	0.16	62,62,64,66	0
7	FMT	H	989	3/?	0.96	0.15	52,52,54,61	0
5	HEM	B	501	43/?	0.96	0.18	29,34,45,51	0
5	HEM	A	501	43/?	0.96	0.17	29,35,43,61	0
8	NA	I	3	1/?	0.97	0.14	47,47,47,47	0
5	HEM	C	501	43/?	0.97	0.16	31,36,41,55	0
8	NA	I	13	1/?	0.97	0.27	47,47,47,47	0
8	NA	I	1	1/?	0.97	0.14	52,52,52,52	0
8	NA	I	2	1/?	0.98	0.18	50,50,50,50	0

The following is a graphical depiction of the model fit to experimental electron density of all instances of the Ligand of Interest. In addition, ligands with molecular weight > 250 and outliers as shown on the geometry validation Tables will also be included. Each fit is shown from different orientation to approximate a three-dimensional view.

PRELIMINARY VALIDATION REPORT

Electron density around DEB F 502:

2mF$_o$-DF$_c$ (at 0.7 rmsd) in gray
mF$_o$-DF$_c$ (at 3 rmsd) in purple (negative)
and green (positive)

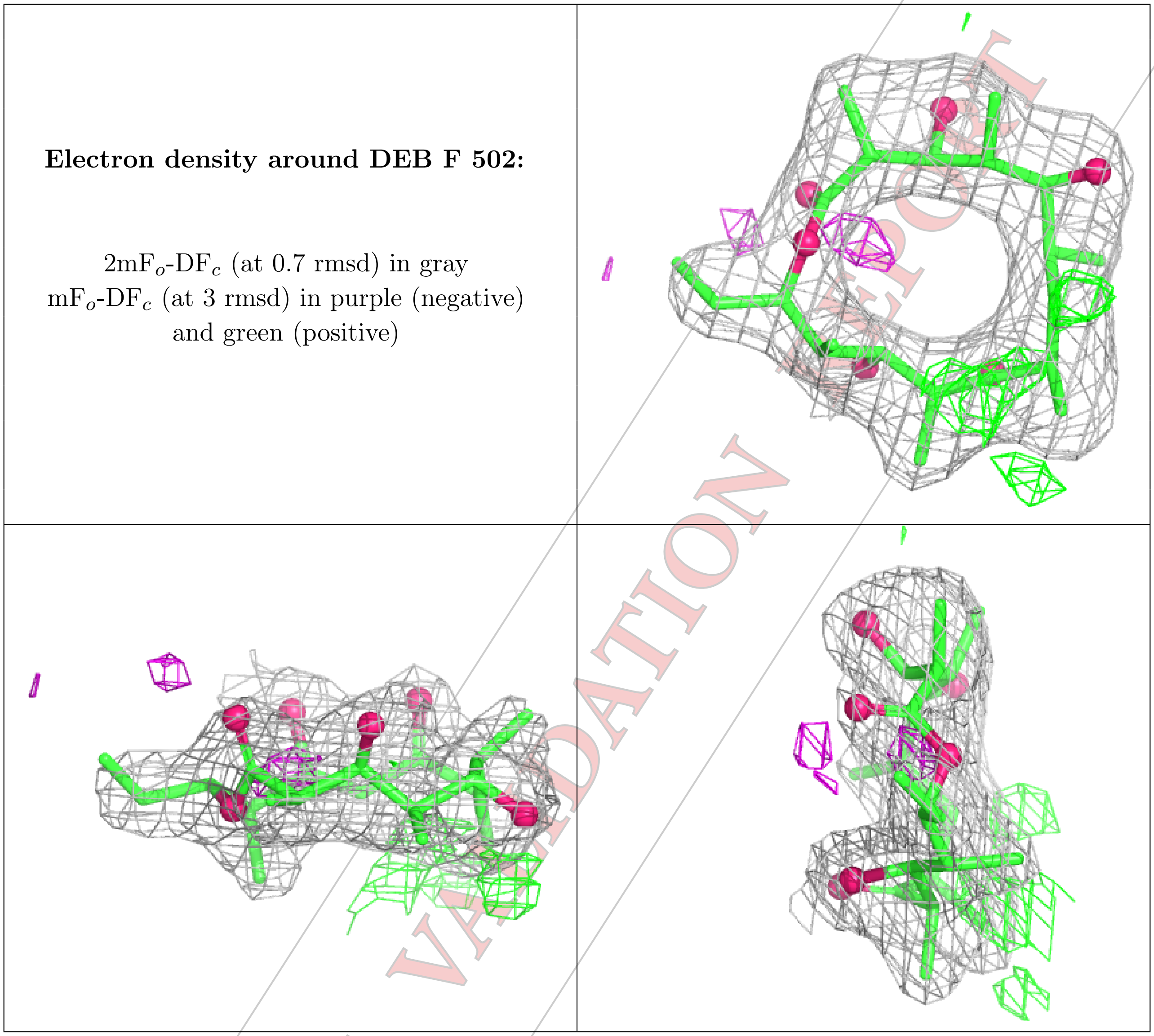

PRELIMINARY VALIDATION REPORT

Electron density around DEB Z 502:

$2mF_o$-DF_c (at 0.7 rmsd) in gray
mF_o-DF_c (at 3 rmsd) in purple (negative)
and green (positive)

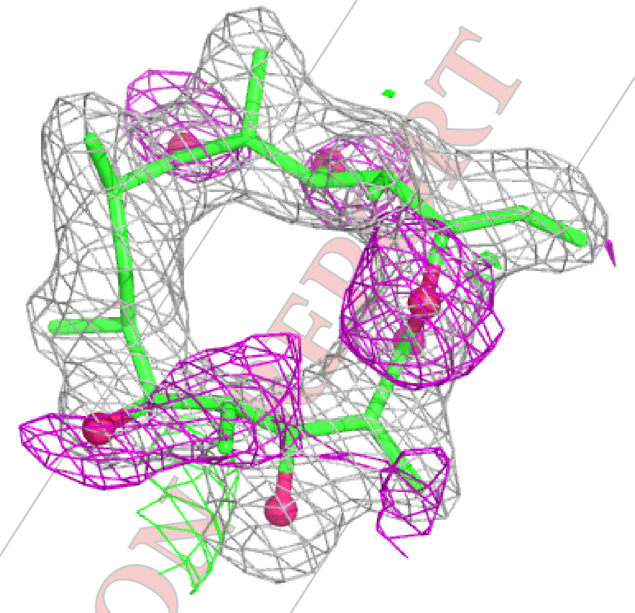

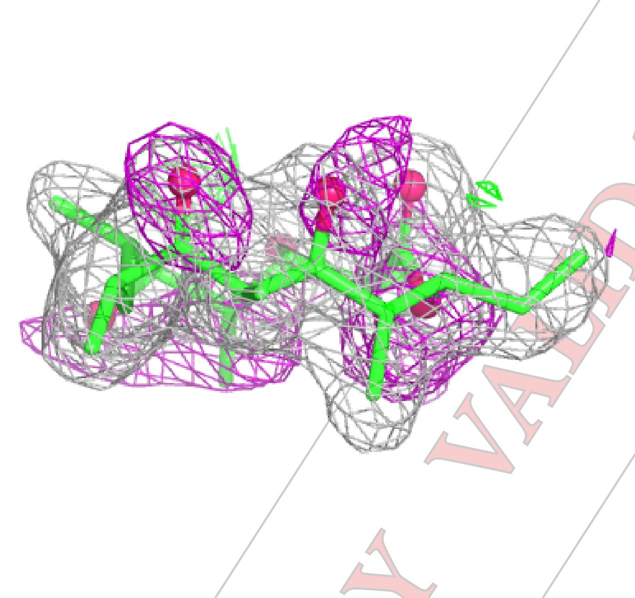

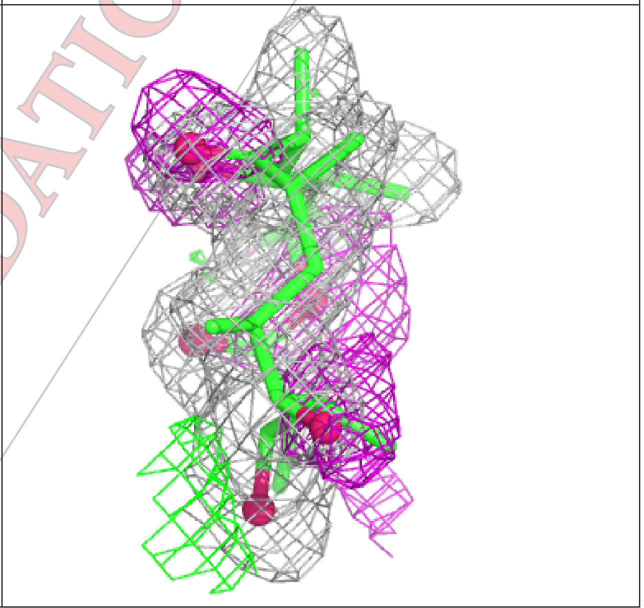

PRELIMINARY VALIDATION REPORT

Electron density around DEB E 502:

2mF$_o$-DF$_c$ (at 0.7 rmsd) in gray
mF$_o$-DF$_c$ (at 3 rmsd) in purple (negative)
and green (positive)

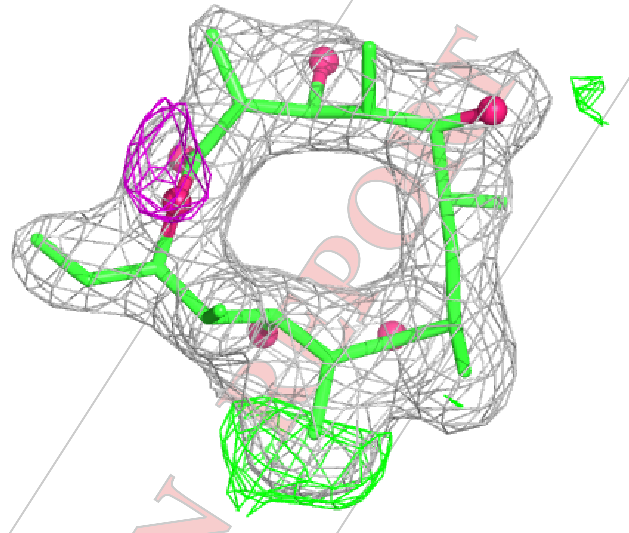

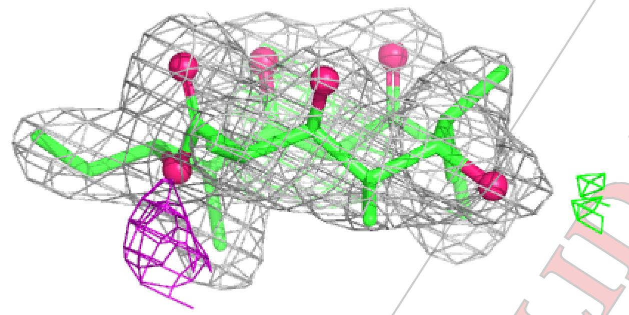

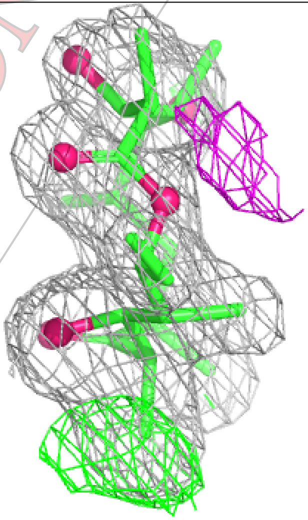

PRELIMINARY VALIDATION REPORT

Electron density around DEB D 502:

$2mF_o$-DF_c (at 0.7 rmsd) in gray
mF_o-DF_c (at 3 rmsd) in purple (negative)
and green (positive)

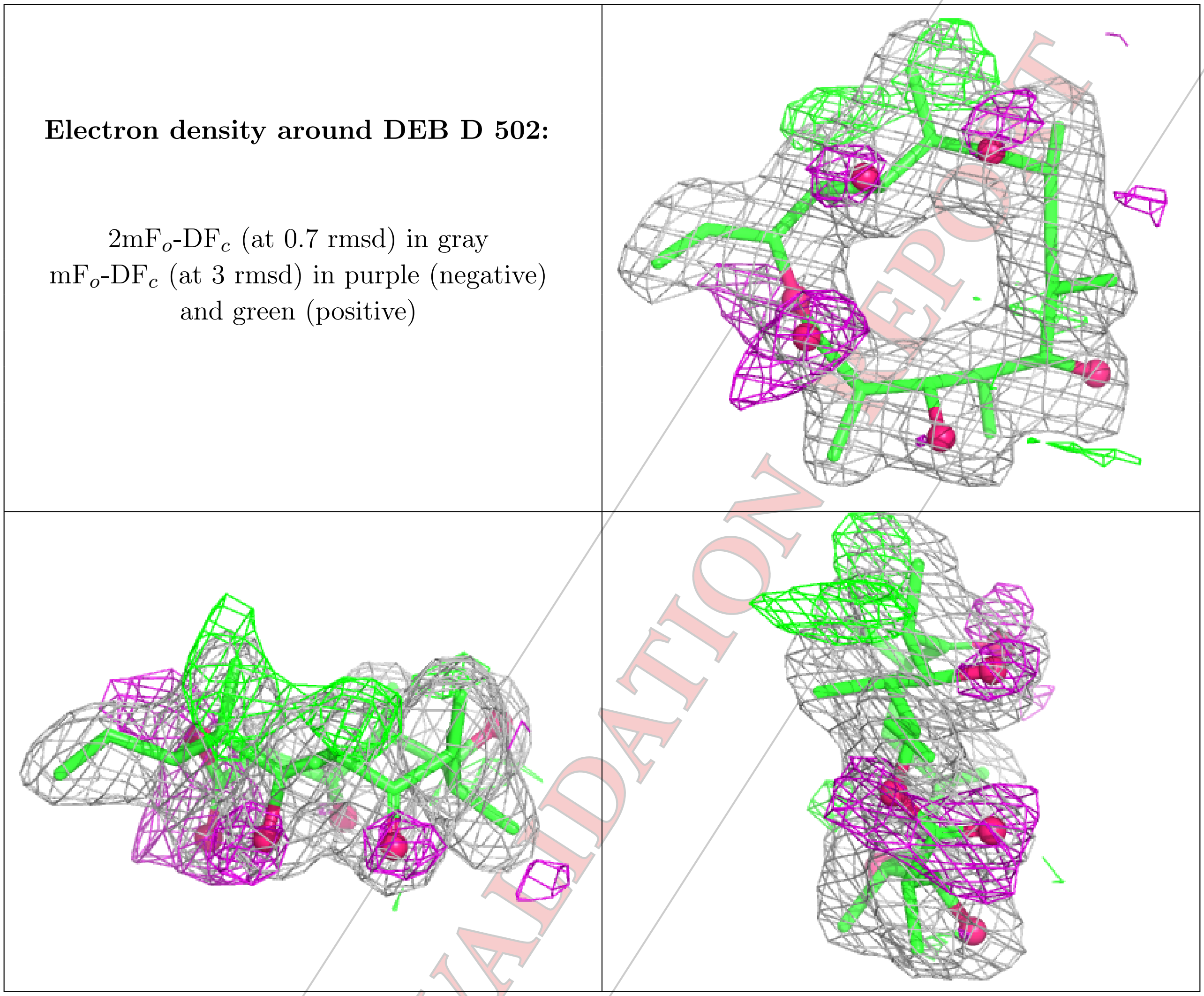

PRELIMINARY VALIDATION REPORT

Electron density around HEM F 501:

$2mF_o$-DF_c (at 0.7 rmsd) in gray
mF_o-DF_c (at 3 rmsd) in purple (negative)
and green (positive)

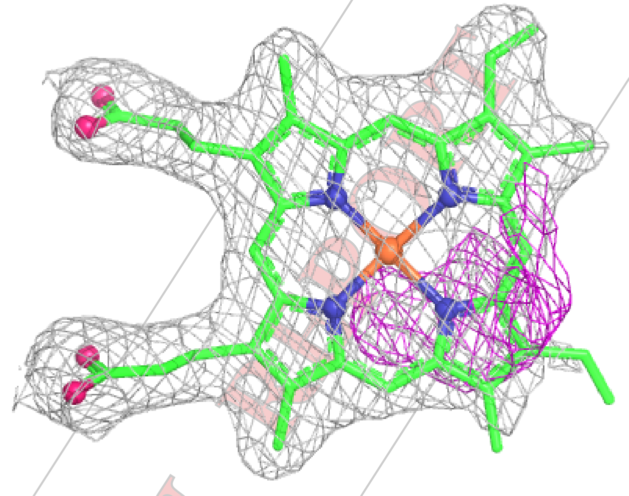

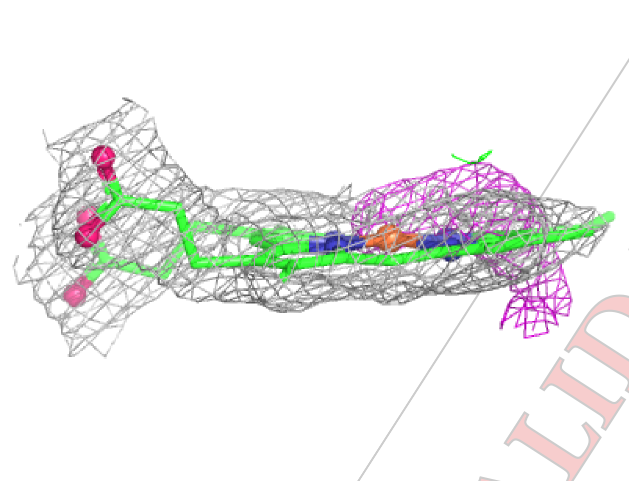

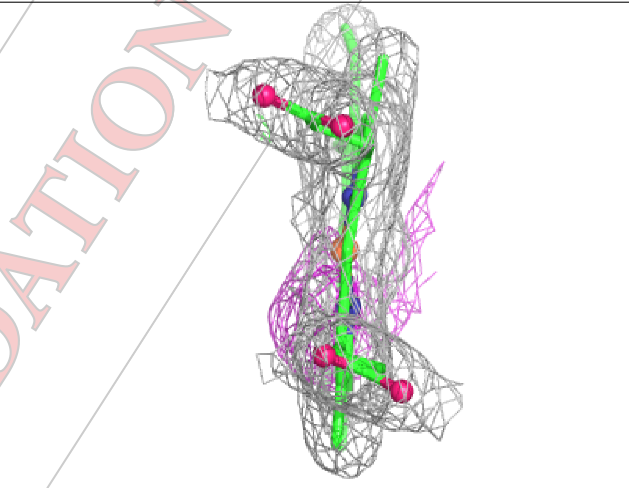

PRELIMINARY VALIDATION

Electron density around DEB A 502:

$2mF_o$-DF_c (at 0.7 rmsd) in gray
mF_o-DF_c (at 3 rmsd) in purple (negative)
and green (positive)

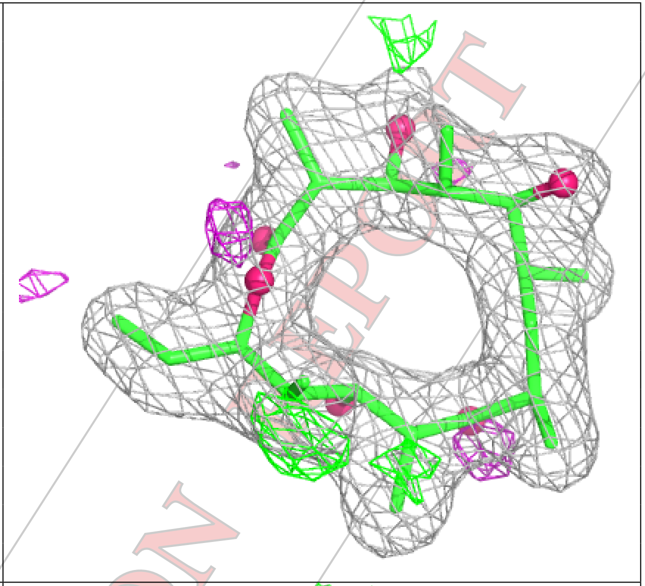

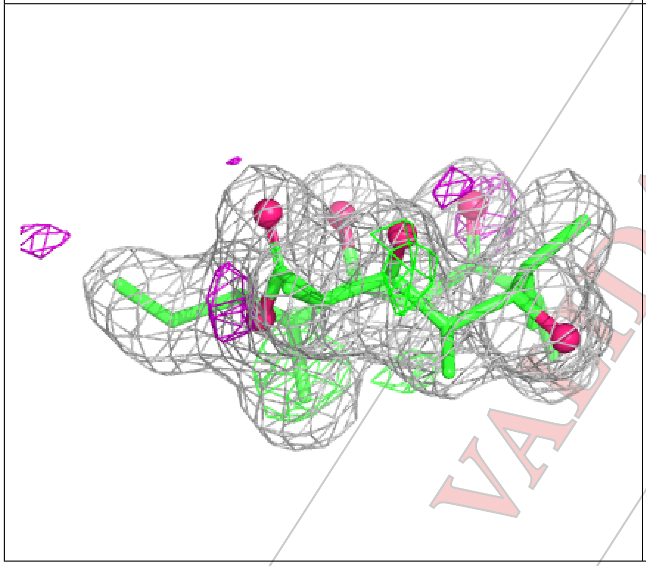

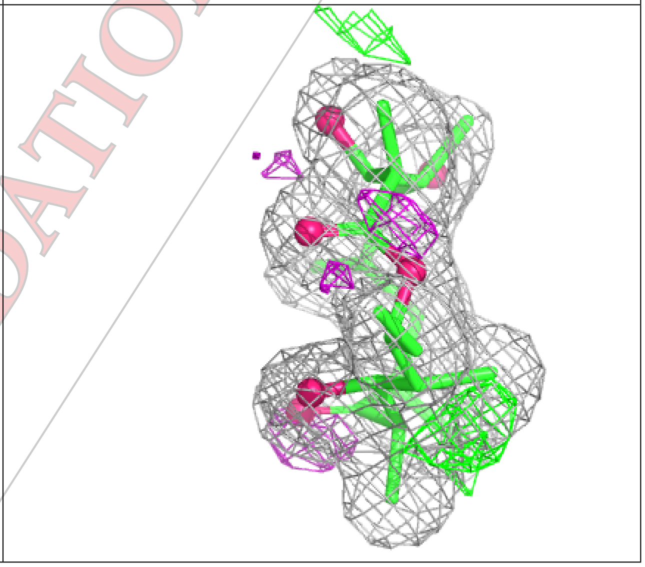

PRELIMINARY VALIDATION REPORT

Electron density around HEM E 501:

$2mF_o$-DF_c (at 0.7 rmsd) in gray
mF_o-DF_c (at 3 rmsd) in purple (negative)
and green (positive)

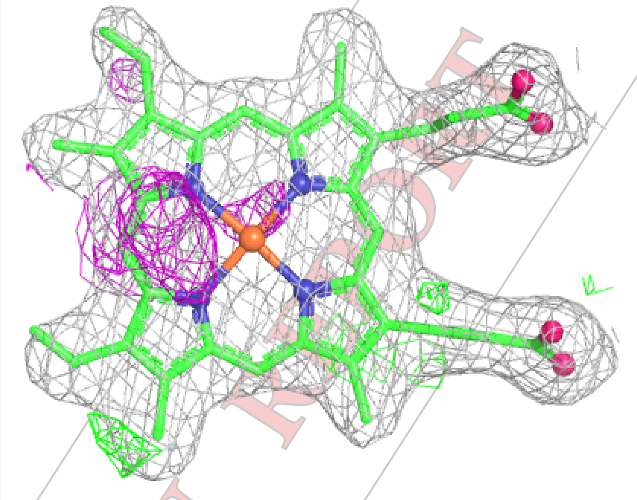

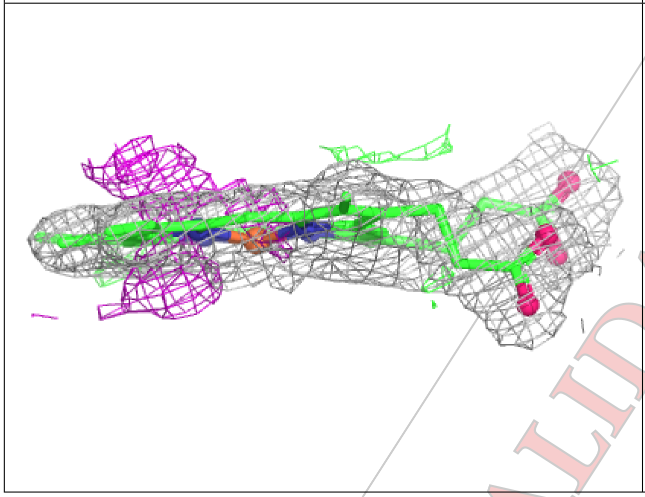

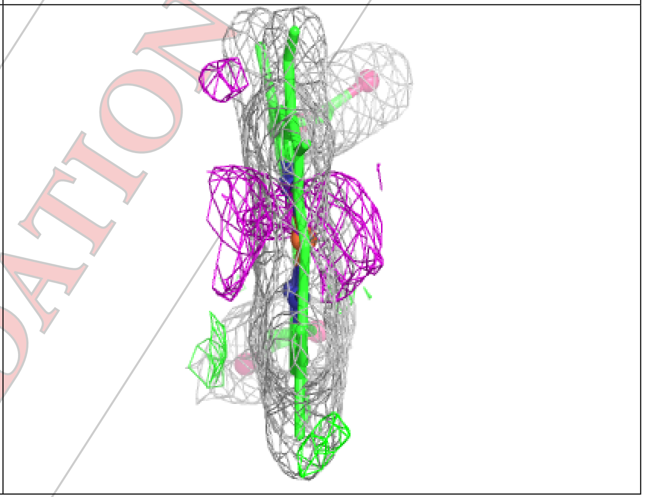

PRELIMINARY VALIDATION REPORT

Electron density around DEB B 502:

2mF$_o$-DF$_c$ (at 0.7 rmsd) in gray
mF$_o$-DF$_c$ (at 3 rmsd) in purple (negative) and green (positive)

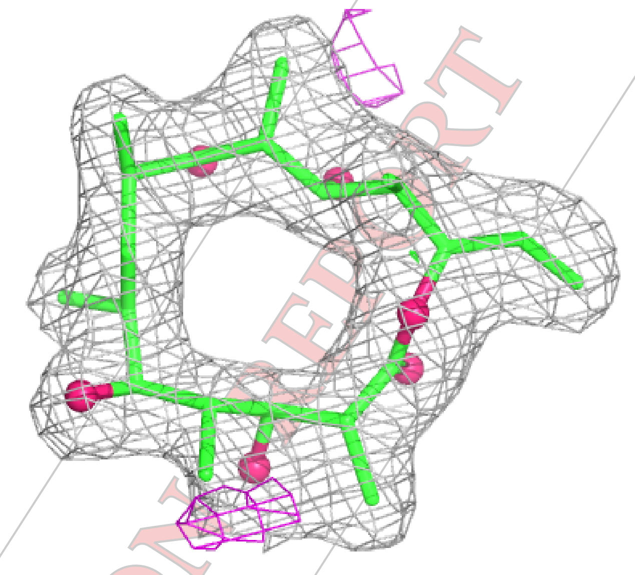

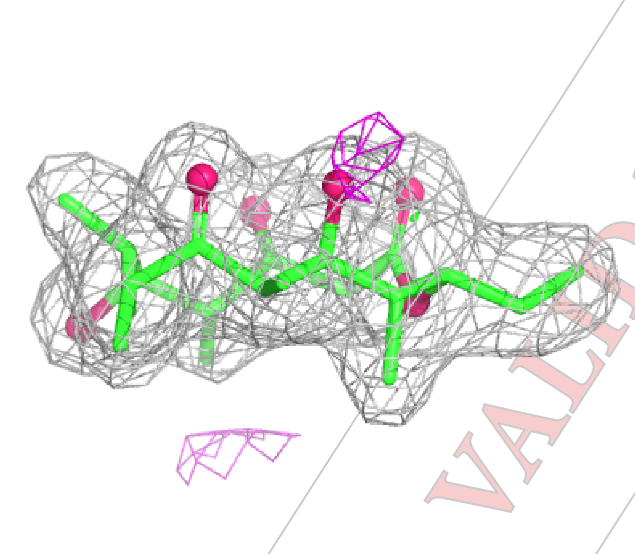

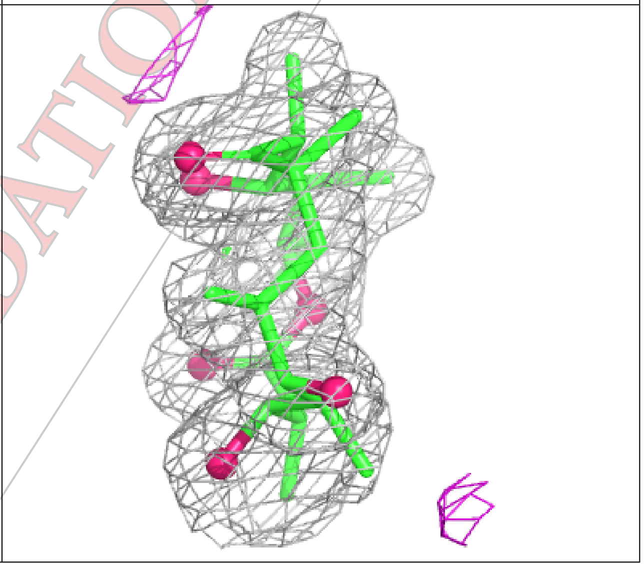

PRELIMINARY VALIDATION REPORT

Electron density around HEM D 501:

2mF$_o$-DF$_c$ (at 0.7 rmsd) in gray
mF$_o$-DF$_c$ (at 3 rmsd) in purple (negative)
and green (positive)

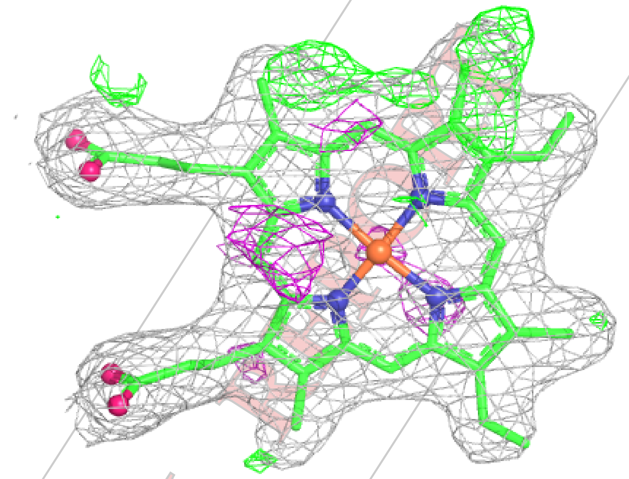

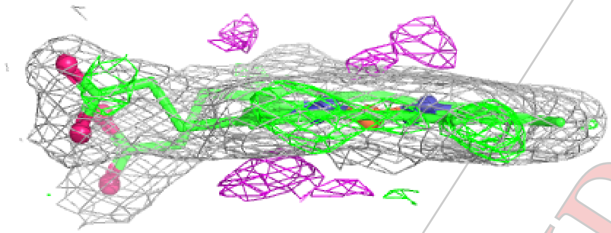

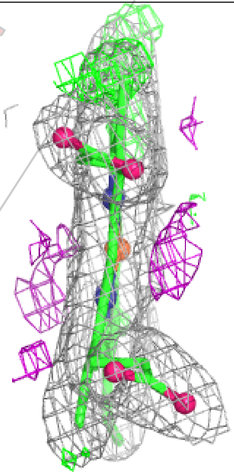

PRELIMINARY VALIDATION

**Electron density around HEM B 501:**<br><br>2mF$_o$-DF$_c$ (at 0.7 rmsd) in gray<br>mF$_o$-DF$_c$ (at 3 rmsd) in purple (negative)<br>and green (positive)	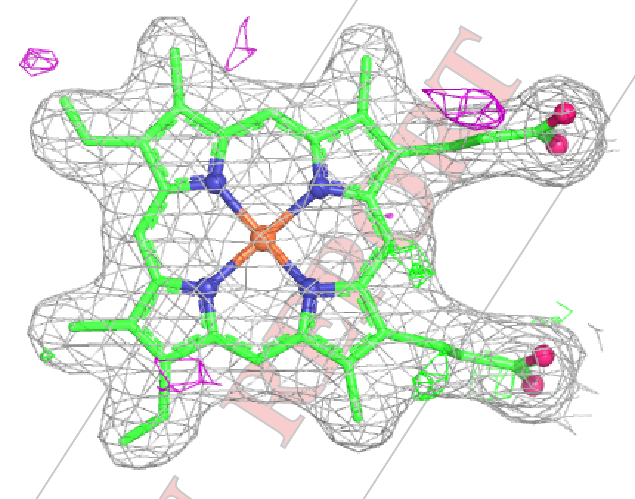
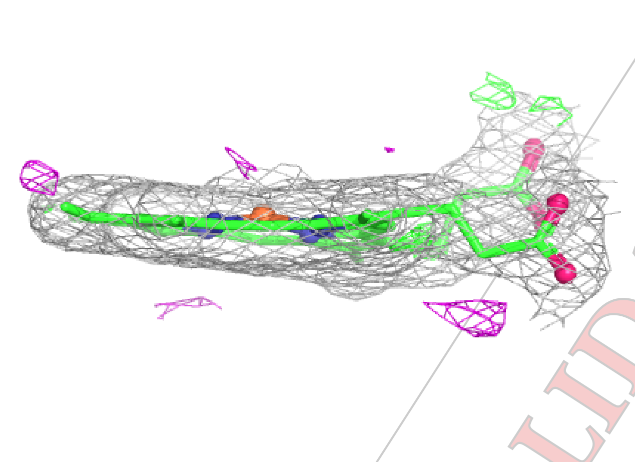	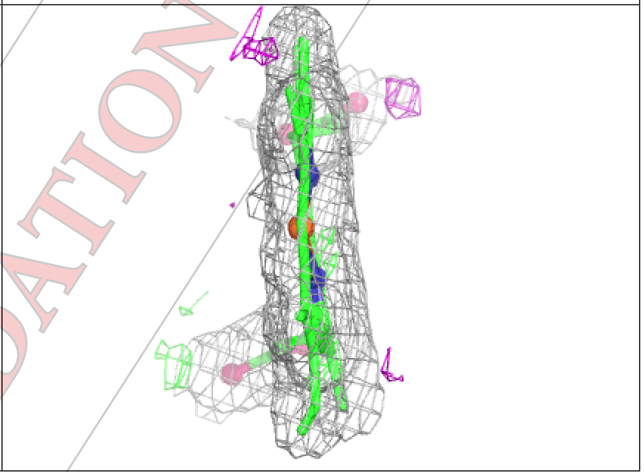

PRELIMINARY VALIDATION REPORT

Electron density around HEM A 501:

2mF$_o$-DF$_c$ (at 0.7 rmsd) in gray
mF$_o$-DF$_c$ (at 3 rmsd) in purple (negative)
and green (positive)

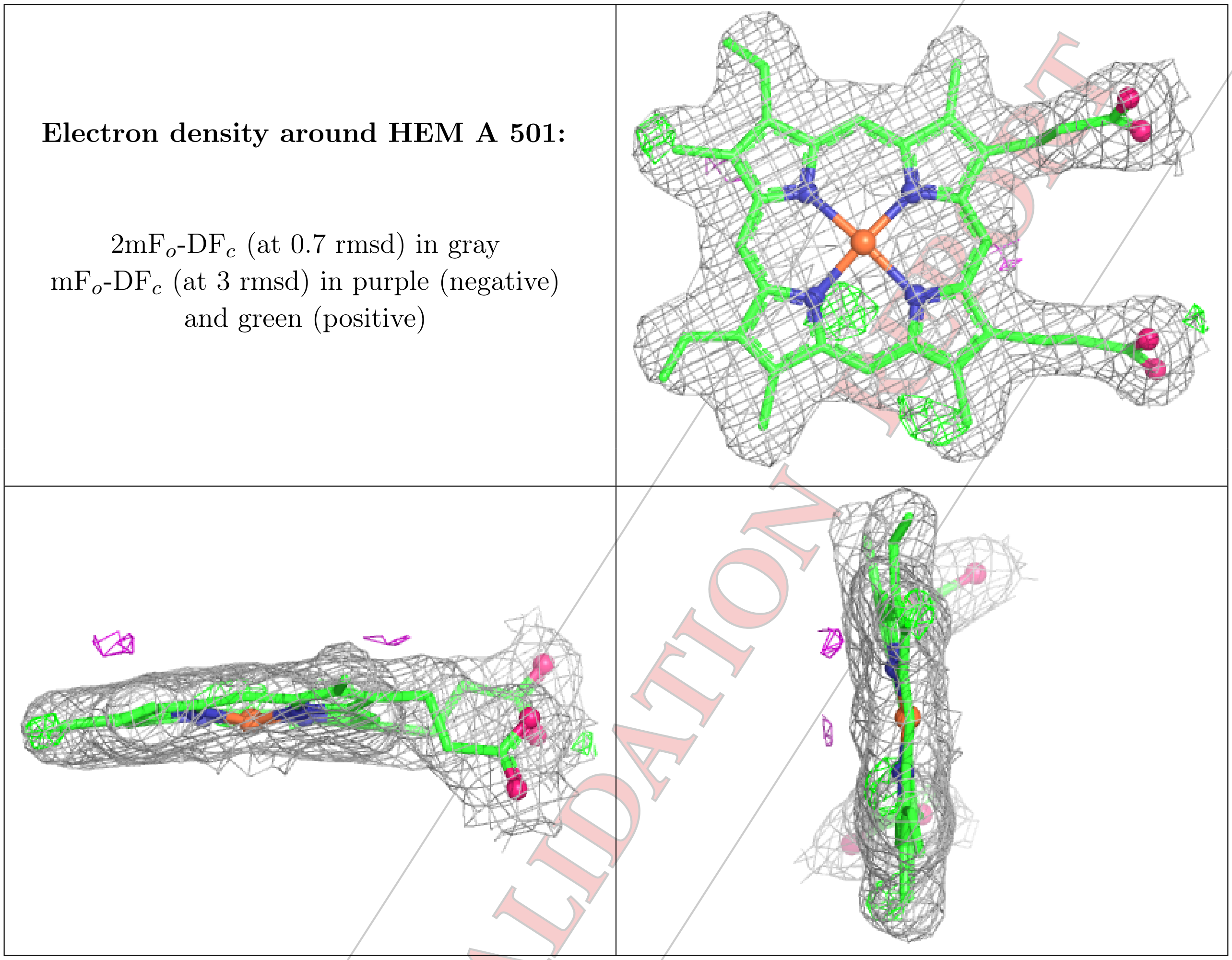

PRELIMINARY VALIDATION REPORT

Electron density around HEM C 501:

2mF$_o$-DF$_c$ (at 0.7 rmsd) in gray
mF$_o$-DF$_c$ (at 3 rmsd) in purple (negative)
and green (positive)

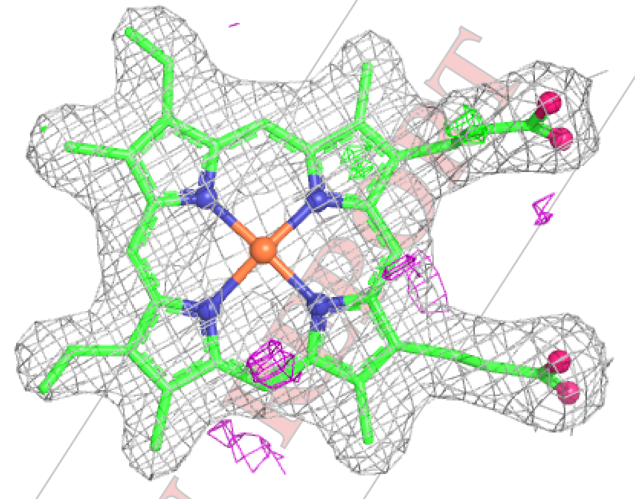

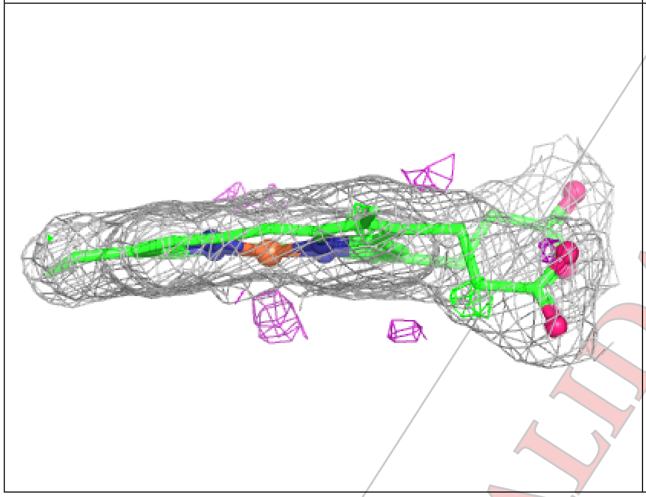

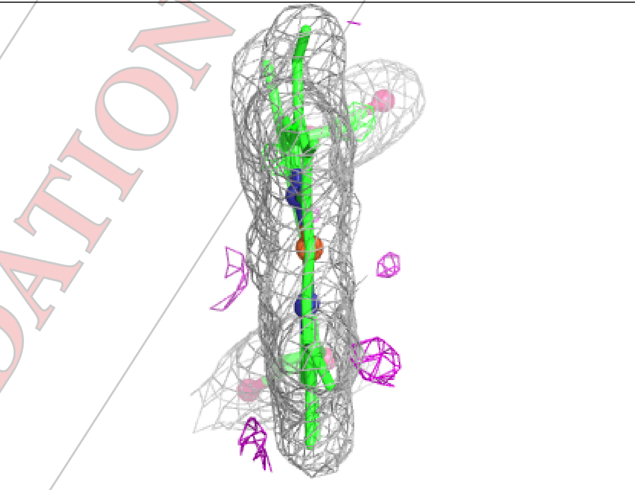

6.5 Other polymers (i)

There are no such residues in this entry.

PRELIMINARY VALIDATION REPORT
