# Supplementary material for: Point Mutations at a Key Site Alter the Cytochrome P450 OleP Structural Dynamics
Source: Biomolecules. 2021 Dec 31;12(1):55. doi: 10.3390/biom12010055 (PMC8774231; doi:10.3390/biom12010055)
Supplement: Supplementary file 1 [file biomolecules-12-00055-s001.zip › Supplementary_Files_Revised/supplementary_material_proof.pdf]

Article

Point mutations at a key site alter the cytochrome P450 OleP structural dynamics

Montemiglio Linda Celeste[1,‡], Gugole Elena[2,‡], Freda Ida[2,‡], Exertier Cécile[2], D'Auria Lucia[2,+], Chen Cheng Giuseppe[3], Nardi Alessandro Nicola[3], Cerutti Gabriele[2,++], Parisi Giacomo[4], D'Abramo Marco[3], Savino Carmelinda[1,*], Vallone Beatrice[2,*]

1 Institute of Molecular Biology and Pathology, CNR c/o Department of Biochemical Sciences "A. Rossi Fanelli", University of Rome Sapienza, P.le A. Moro 5, 00185 Rome, Italy; lindaceleste.montemiglio@cnr.it (M.L.C.); cecile.exertier@uniroma1.it (E.C.); carmelinda.savino@cnr.it (S.C.)
2 Department of Biochemical Sciences "A. Rossi Fanelli", University of Rome, Sapienza, P.le A. Moro 5, 00185 Rome, Italy; elena.gugole@uniroma1.it (G.E.); ida.freda@uniroma1.it (F.I.); beatrice.vallone@uniroma1.it (V.B.)
3 Department of Chemistry, University of Rome, Sapienza, P.le A. Moro 5, 00185 Rome, Italy; giuseppe.chen@uniroma1.it (C.C.G.); alessandronicola.nardi@uniroma1.it (N.A.N.); marco.dabramo@uniroma1.it (D.M.)
4 Center for Life Nano & Neuro-Science, Fondazione Istituto Italiano di Tecnologia, IIT, 00185 Rome, Italy; giacomo.parisi@iit.it (P.G.)
+ Present Address: IBS, Institute de Biologie Structurale, 38000 Grenoble, France; lucia.dauria@ibs.fr (D.L.)
++ Present Address: Zuckerman Mind Brain Behavior Institute, Columbia University, New York, NY 10027, USA; gc2695@columbia.edu (C.G.)
‡ These authors contributed equally
* Correspondence: carmelinda.savino@cnr.it (S.C.), beatrice.vallone@uniroma1.it (V.B.); Tel.: 39 06 49910548.

SUPPLEMENTARY MATERIAL

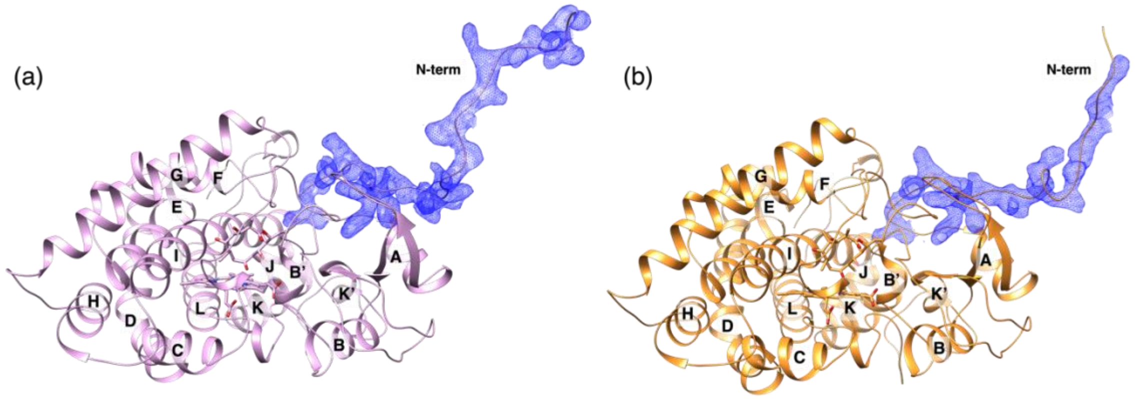

Figure S1. Reconstruction of the N-terminus of OleP in G92W-6DEB and S240Y-6DEB. Detail of the N-terminus of OleP G92W-6DEB (a, pink ribbon representation) and S240Y-6DEB (b, orange ribbon representation) whose clear electron density (shown as $2|F_o|-|F_c|$ contoured at ± 1 σ blue mesh around residues number 0-25 in panel (a) and 2-25 in panel (b) allowed the visualization and the reconstruction of the tail for the first time. Heme group and 6DEB are represented in sticks; capital letters indicate the secondary structure elements according to the P450 nomenclature.

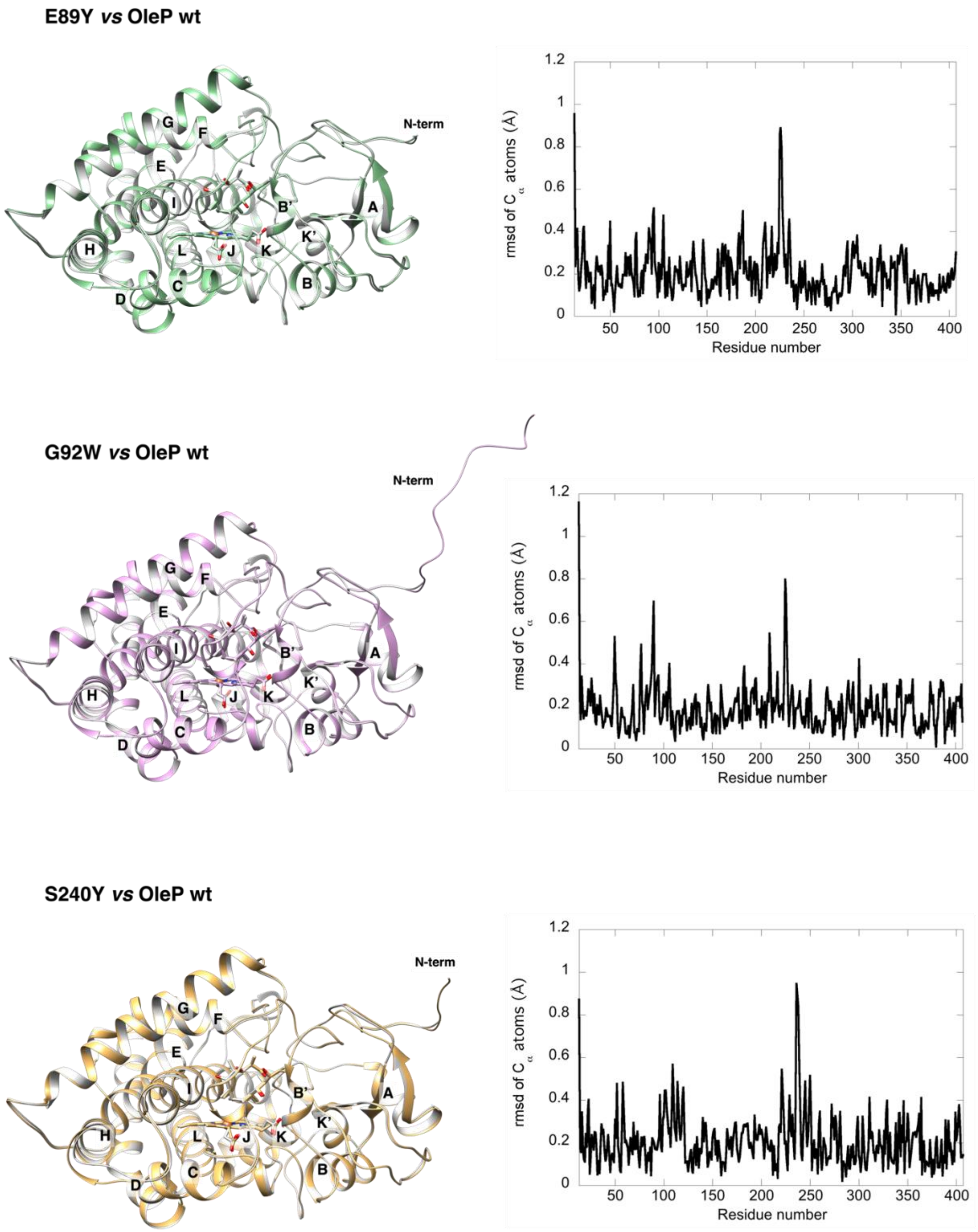

Figure S2. Overall structure of OleP mutants bound to 6DEB. Left. Superposition of OleP mutant structures (E89Y, G92W, S240Y) bound to 6DEB with the structure of the OlePwt-6DEB complex in the closed state [8]. E89Y is in green, G92W in pink, S240Y in weat and OleP wt is in light grey ribbon representation. Heme group and 6DEB are represented in sticks; capital letters indicate the secondary structure elements according to the P450 nomenclature. Right. The rmsd on Cα, calculated superposing the structures of OleP-6DEB wt and mutants, is reported as a function of the residue number.

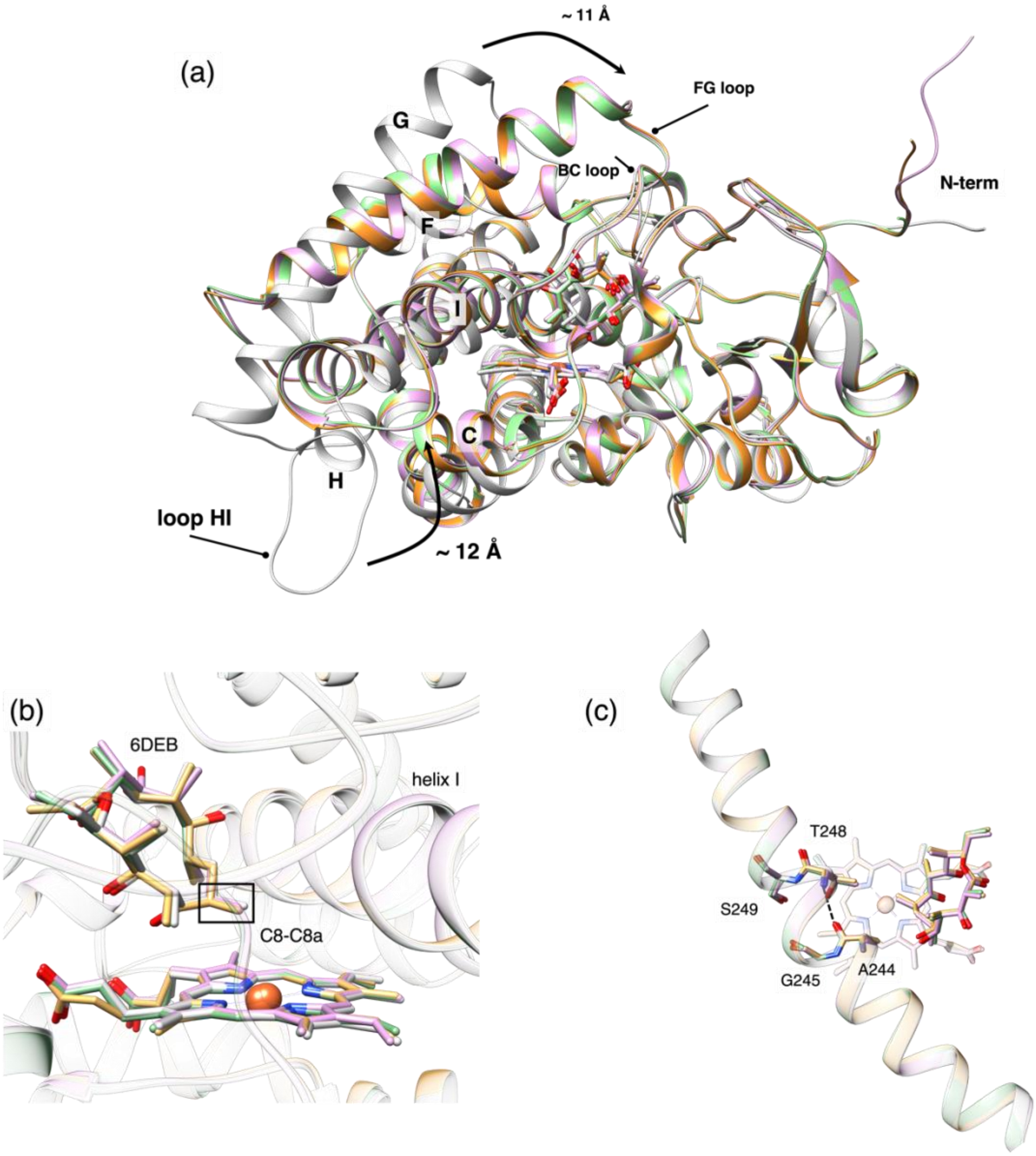

Figure S3. The typical structural features of the closed OleP-6DEB complex are preserved in mutants. (a) Secondary structure superposition of the open structure of wtOleP-6DEB (grey, pdb code 5MNV [8]) and the structures of the closed OleP-6DEB mutants: E89Y is in green ribbon representation, G92W is in pink, S240Y is in orange. The structures are displayed in a nonstandard orientation for P450s to enable the visualization of the structural transition. 6DEB and the heme molecules are in sticks, colored according to the corresponding structure. The secondary structure elements mostly involved in the transition are labelled. Arrows follow the direction of the open-to-closed transition. **(b)** Close up view of the active site of the OleP mutants. The molecules of 6DEB bound to each mutant are superposed and colored depending on the corresponding structure (E89Y, green; G92W, pink; S240Y, orange). The black squared box indicates the C8-C8a bond, which is the OleP target, placed at ~ 4 Å from the heme iron. **(c)** Zoom on the I helix of the OleP mutants in complex with 6DEB. In all mutants the position adopted by 6DEB in the active site allows the

displacement of the axial water ligand and induces the I helix bending and the formation of the catalytic cleft at the 244-248 turn. Residues lining the cleft are labelled and represented as sticks. Dashed line indicates hydrogen bond. 6DEB is in sticks colored according to the corresponding mutant structure.

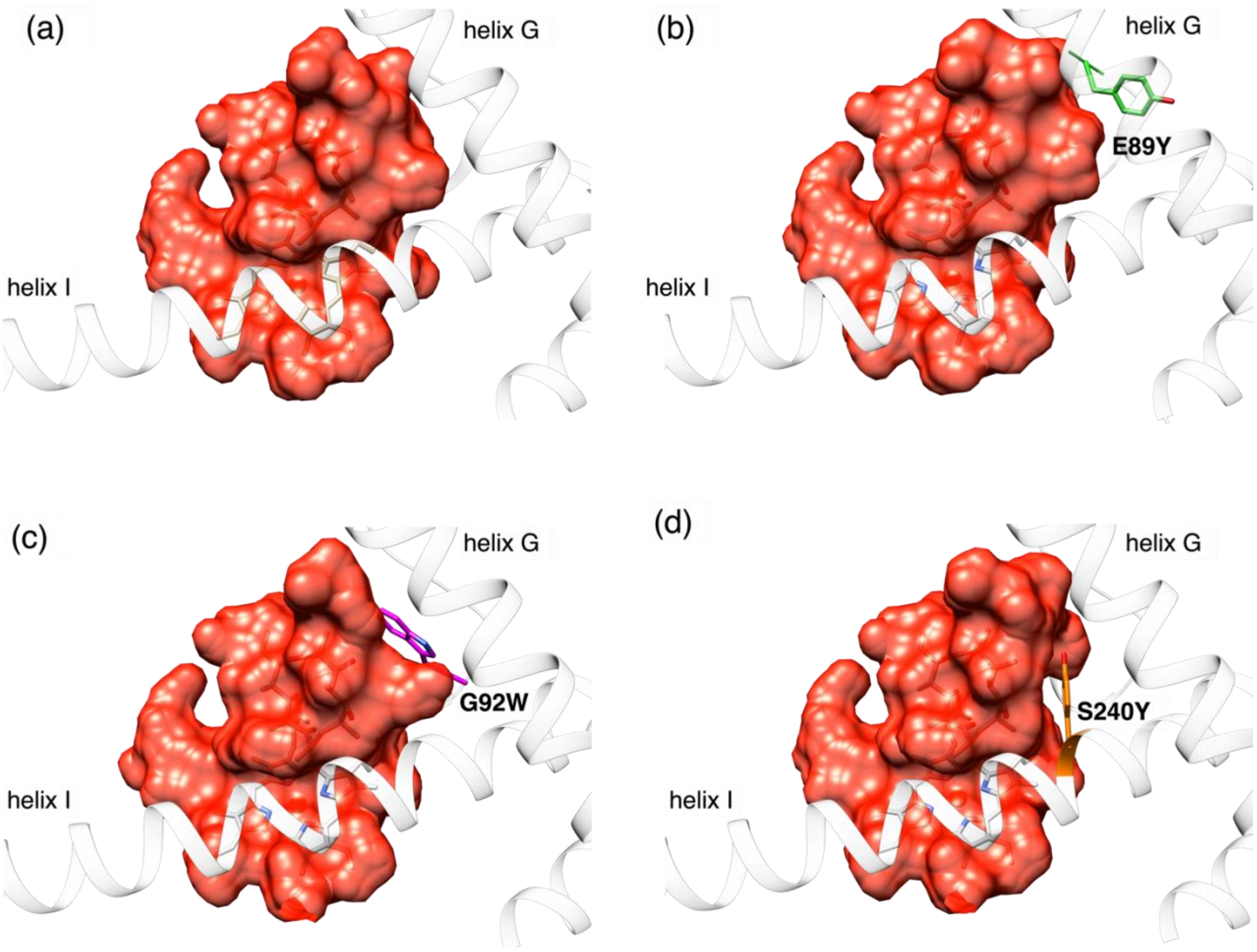

Figure S4. Binding site of OleP wt (pdb 5MNS, [8]) and mutants bound to 6DEB. The surface-accessible volume of the heme pocket of OleP wt (a, ~606.7 Å^3), E89Y (b, ~671.2 Å^3), G92W (c, ~582.5 Å^3), S240Y (d, ~566.8 Å^3) is shown as a red surface, as calculated by CASTp server [30]. Secondary structural elements are labelled. The mutated residues (E89Y, forest green; G92W, magenta; S240Y, orange), heme and 6DEB are shown as sticks.

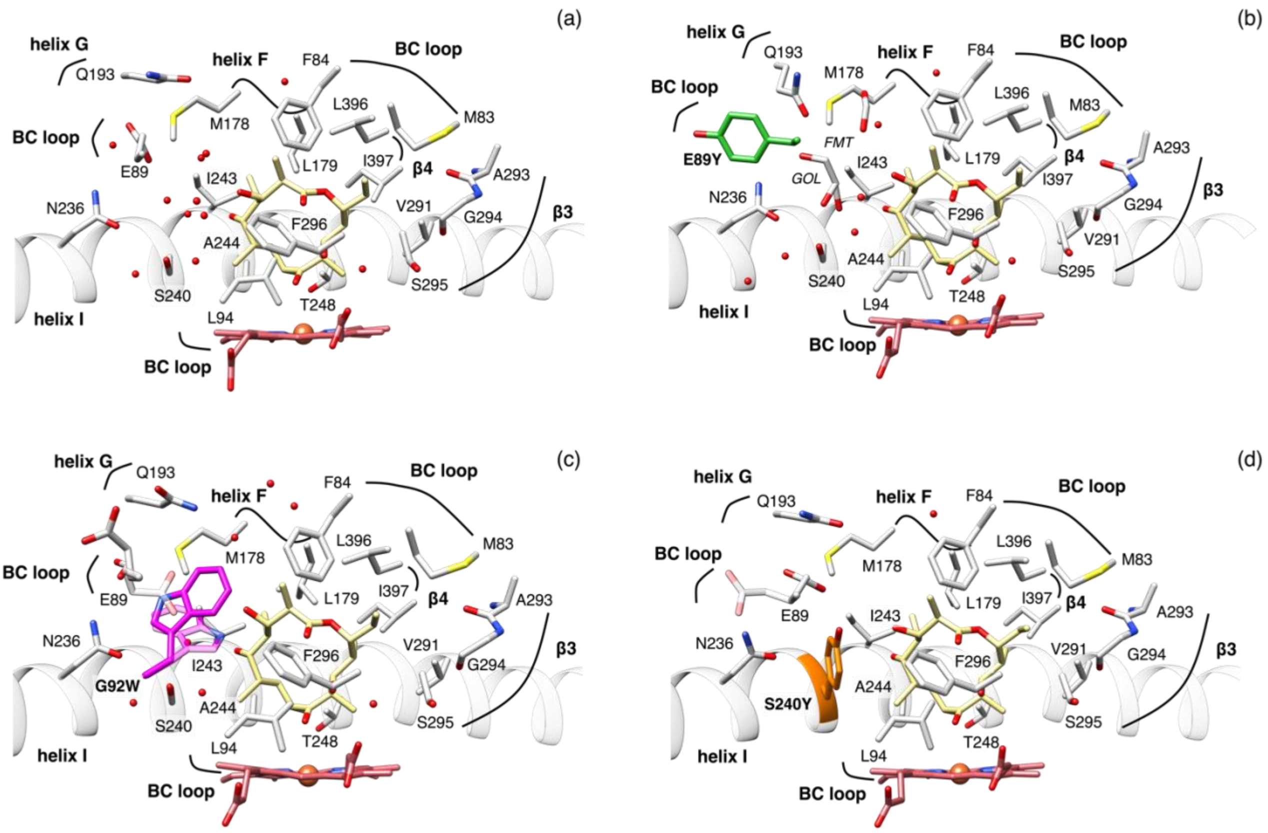

Figure S5. Active site organization of OleP mutants bound to 6DEB. Mutations at the solvent cavity do not alter the van der Waals interactions that the aglycone substrate 6DEB (khaki sticks) establishes with OleP residues exposed to the main cavity and located on the BC loop (M83, F84, L94), the helix F (M178, L179), the central portion of helix I (I243, A244, T248), the β-hairpin β 3 (V291, A293, G294, S295, F296) and the β-hairpin β 4 (L396 , I397). (a) Closed wild type OleP-6DEB active site (pdb code 5MNS, [8]); (b) E89Y-6DEB active site; (c) G92W-6DEB active site; (d) S240Y-6DEB active site. Residues within 5 Å and involved in substrate contacts are shown as sticks. E89Y, G92W and S240Y are colored in forest green, magenta and orange, respectively. Double conformations are reported, showing the less occupied conformer in transparency. Solvent molecules of glycerol (GOL) and formate ion (FMT) are labelled in italic. Secondary structures are represented and labelled in bold. Heme is represented as red sticks, waters are in red spheres.

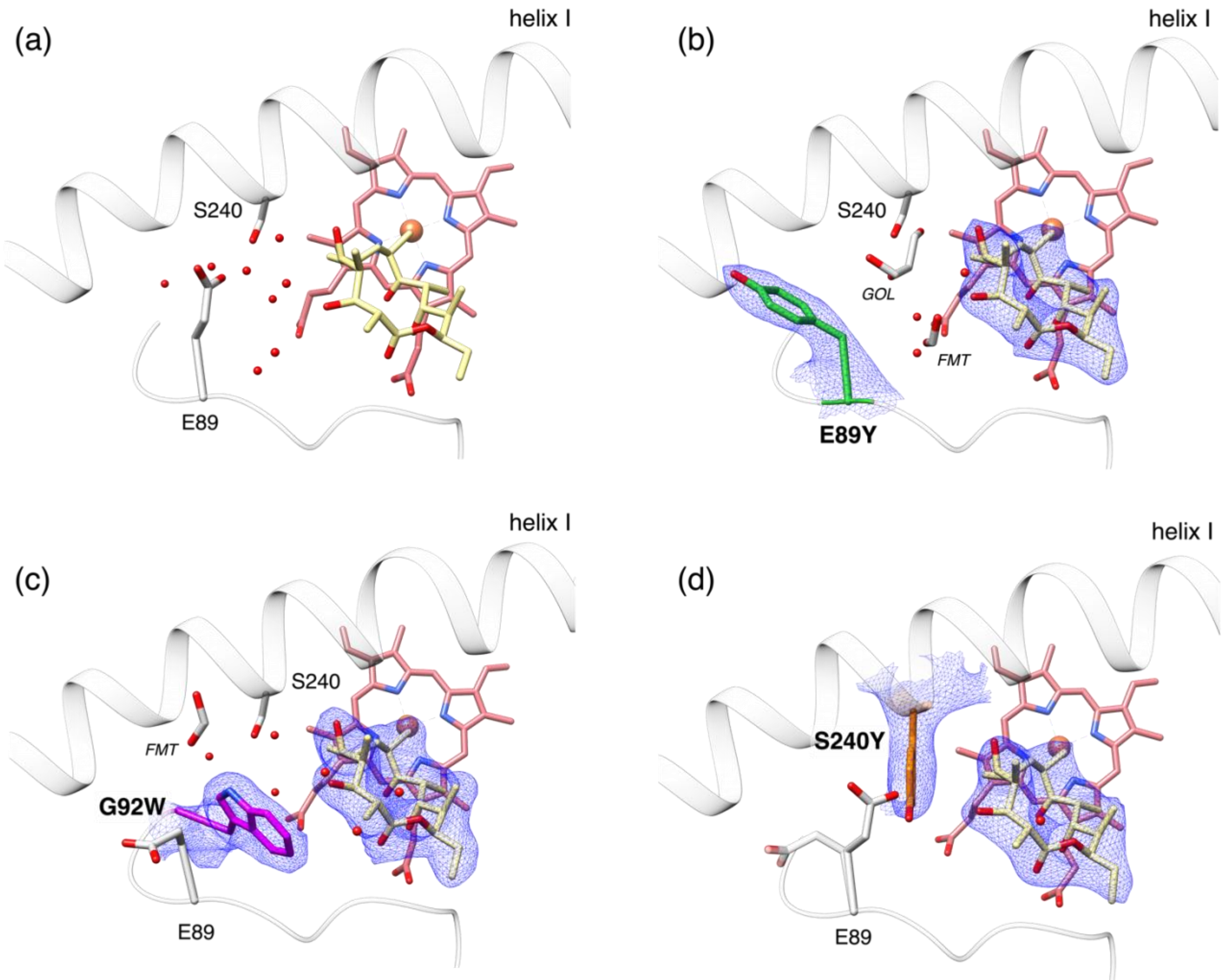

Figure S6. The solvent cavity in OleP wild type and mutants. Close up view of the changes in the solvent cavity induced by mutations E89Y (panel b), G92W (panel c), and S240Y (panel d) with respect to the wild type (panel a, [8]). Residues at position 89, 92, and 240 are shown as sticks and labelled. The electron density found at mutated sites and 6DEB is shown as $2|F_o|-|F_c|$ contoured at $\pm$ 1 σ blue mesh. Double conformations are reported, showing the less occupied conformer in transparency. Solvent molecules of glycerol (GOL) and formate ion (FMT) are labelled in italic. Heme and 6DEB are represented as red and khaki sticks, respectively; waters are in red spheres.

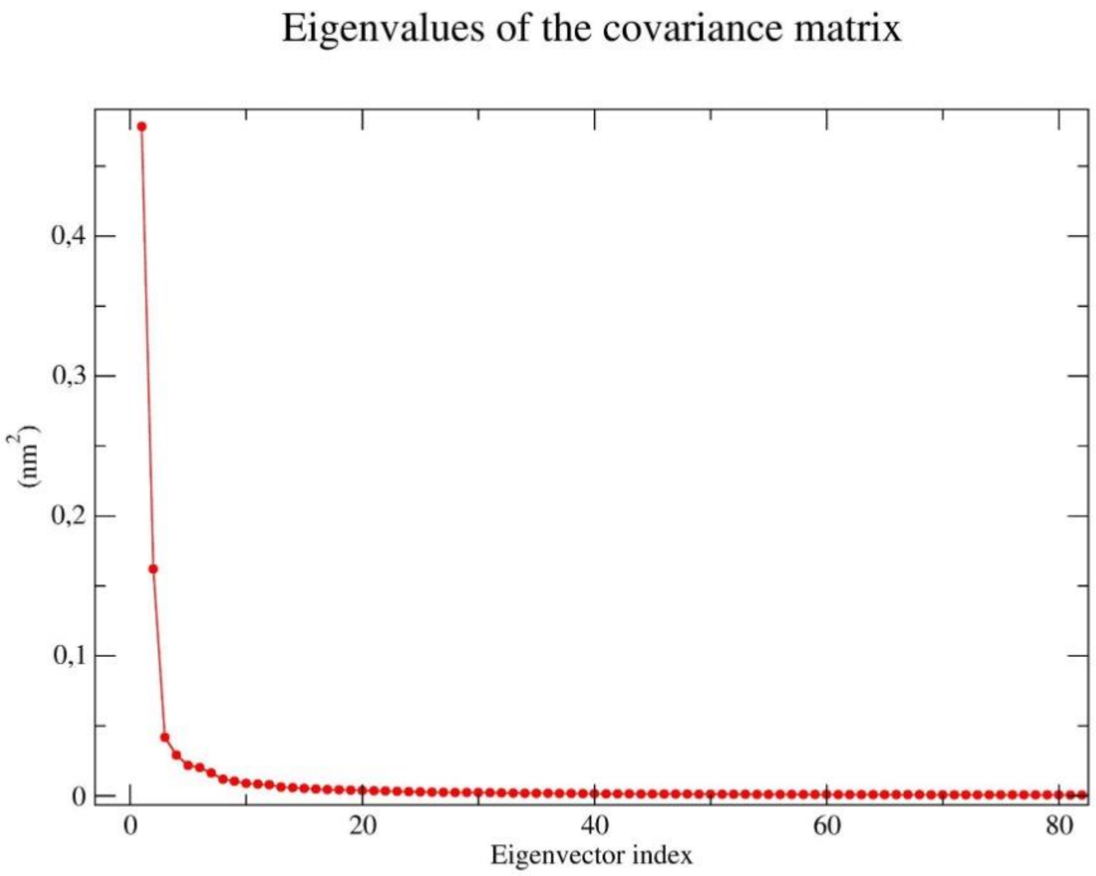

Figure S7. Essential dynamics of concatenated trajectory: inspection of eigenvalues. Normalized spectrum of the eigenvalues as obtained by the essential dynamics analysis of the concatenated MD trajectory.

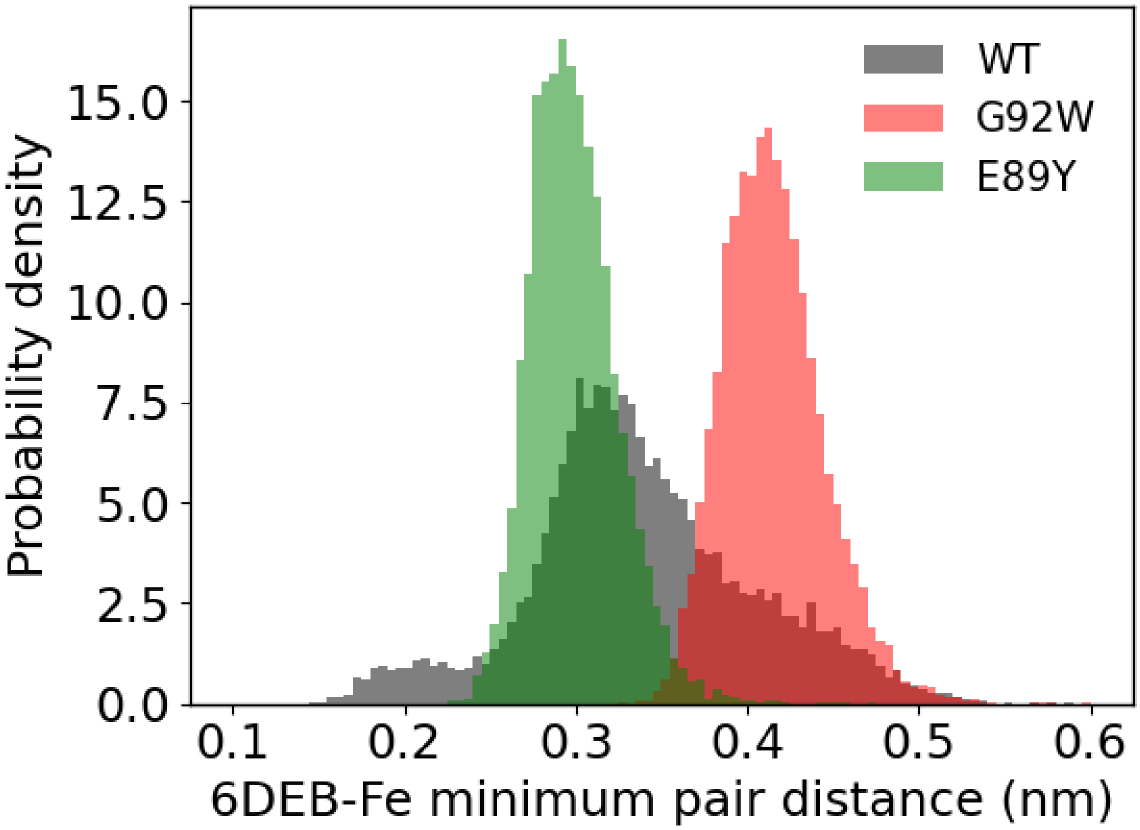

Figure S8. The distributions of the minimum pair distance between heme iron and substrate heavy atoms for OleP WT, E89Y, and G92W bound to 6DEB, as obtained by MD simulations. The modes for the minimum pair distance between heme iron and substrate heavy atoms distributions for the WT, E89Y, and G92W, are 0.30, 0.29, and 0.41 nm, respectively.

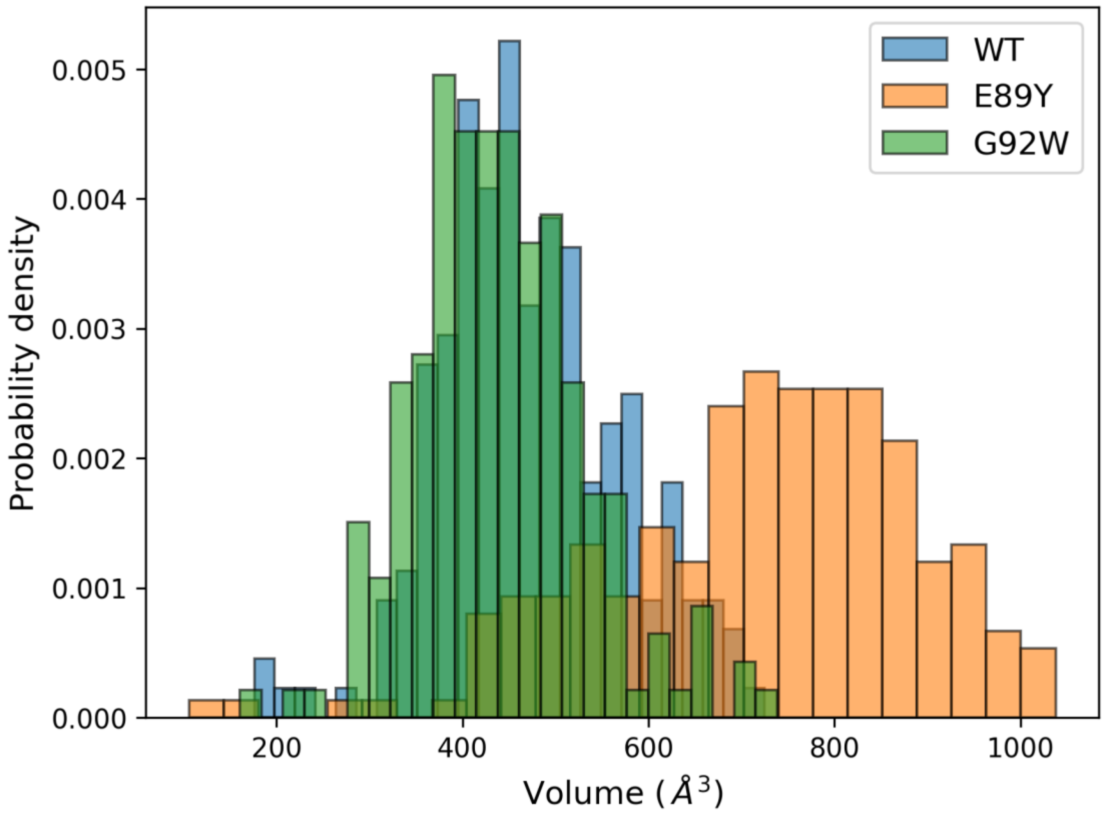

Figure S9. The distributions of the volume of the OleP active site for the WT, E89Y, and G92W, as obtained by MD simulations. The distribution modes of the volumes of the OleP active site for the WT, E89Y and G92W are 450.3, 721.0 and 380.3 Å^3, respectively.

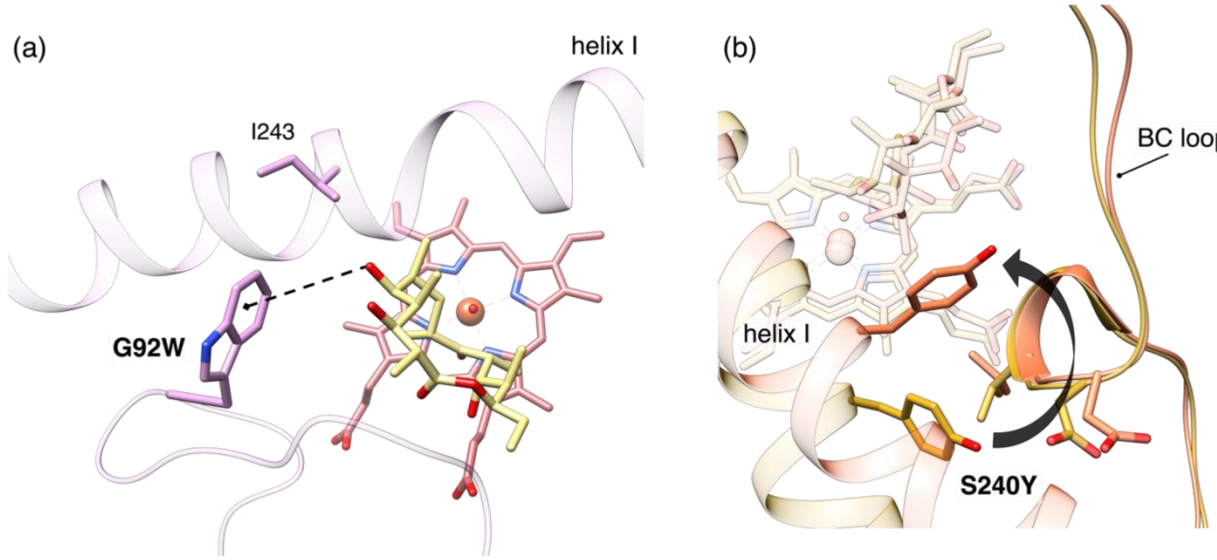

Figure S10. Modeling G92W and S240Y in the open-6DEB bound OleP wild type structure. Mutations at G92W and S240Y were manually introduced in the structure of the open wild type OleP-6DEB complex (pdb code 5MNV [8]). Rotamers showing unfavorable contacts were discarded. (a) The possible structural scenario of the open active site of G92W-6DEB is shown. Dashed line indicates the induced dipole-dipole type interaction that could form between the aromatic ring of W92 and the hydroxyl group at C5 of 6DEB (~ 5 Å). I243 located on helix I is also shown. It is placed at ~ 4.4 Å distance from G92W, and ~ 3.6 Å distance from 6DEB, establishing van der Waals interactions. Heme and 6DEB are represented as red and khaki sticks, respectively; the axial water ligand is shown as red sphere. (b) Superposition of the active site of S240Y-6DEB in the open conformation (orange) and in the closed conformation (salmon) as derived from the S240Y-6DEB open structure modelling and the S240Y-6DEB closed crystal structure. The black arrow follows the possible movement that Y240 could experience during the open-to-closed transition. Residues 91-93 on the BC loop are shown as sticks. Heme and 6DEB are represented as transparent sticks; the axial water ligand is shown as transparent red sphere.
