# Supplementary material for: Point Mutations at a Key Site Alter the Cytochrome P450 OleP Structural Dynamics
Source: Biomolecules. 2021 Dec 31;12(1):55. doi: 10.3390/biom12010055 (PMC8774231; doi:10.3390/biom12010055)
Supplement: Supplementary file 1 [file biomolecules-12-00055-s001.zip › Supplementary_Files_Revised/e89y-6deb_val-report-full_P1.pdf]

Preliminary Full wwPDB X-ray Structure Validation Report (i)

Nov 16, 2021 – 11:56 am GMT

Deposition ID : D_1292119129

This is a Preliminary Full wwPDB X-ray Structure Validation Report.

This report is produced by the wwPDB Deposition System during initial deposition but before annotation of the structure.

We welcome your comments at *validation@mail.wwpdb.org*
A user guide is available at
https://www.wwpdb.org/validation/2017/XrayValidationReportHelp
with specific help available everywhere you see the (i) symbol.

PRELIMINARY VALIDATION REPORT

The following versions of software and data (see references (i)) were used in the production of this report:

MolProbity	:	4.02b-467
Mogul	:	1.8.4 (270009), CSD as541be (2020)
Xtriage (Phenix)	:	1.13
EDS	:	2.23.2
Percentile statistics	:	20191225.v01 (using entries in the PDB archive December 25th 2019)
Refmac	:	5.8.0267
CCP4	:	7.1.010 (Gargrove)
Ideal geometry (proteins)	:	Engh & Huber (2001)
Ideal geometry (DNA, RNA)	:	Parkinson et al. (1996)
Validation Pipeline (wwPDB-VP)	:	2.23.2

1 Overall quality at a glance (i)

The following experimental techniques were used to determine the structure:
X-RAY DIFFRACTION

The reported resolution of this entry is 2.44 Å.

Percentile scores (ranging between 0-100) for global validation metrics of the entry are shown in the following graphic. The table shows the number of entries on which the scores are based.

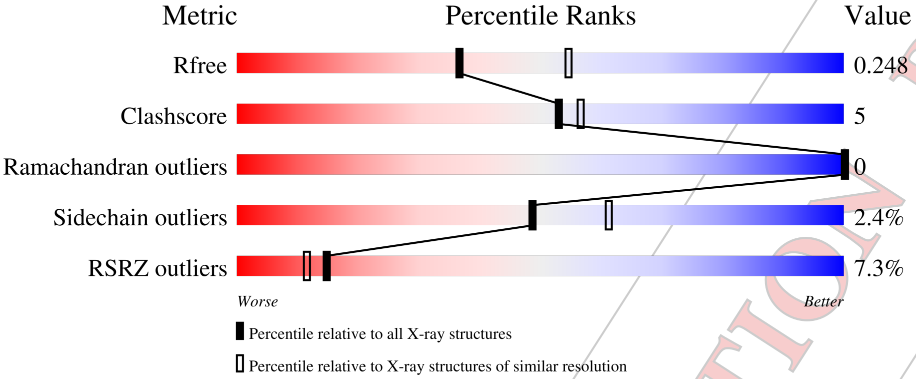

Metric	Whole archive (#Entries)	Similar resolution (#Entries, resolution range(Å))
R_{free}	130704	1564 (2.46-2.42)
Clashscore	141614	1631 (2.46-2.42)
Ramachandran outliers	138981	1617 (2.46-2.42)
Sidechain outliers	138945	1617 (2.46-2.42)
RSRZ outliers	127900	1547 (2.46-2.42)

The table below summarises the geometric issues observed across the polymeric chains and their fit to the electron density. The red, orange, yellow and green segments of the lower bar indicate the fraction of residues that contain outliers for >=3, 2, 1 and 0 types of geometric quality criteria respectively. A grey segment represents the fraction of residues that are not modelled. The numeric value for each fraction is indicated below the corresponding segment, with a dot representing fractions <=5% The upper red bar (where present) indicates the fraction of residues that have poor fit to the electron density. The numeric value is given above the bar.

Mol	Chain	Length	Quality of chain
1	A	396	5%<br>86% 14%
1	B	396	3%<br>88% 11% •
1	D	396	6%<br>81% 18% •
2	C	398	%<br>89% 10% •
3	E	390	11%<br>87% 13% •

Continued on next page...

PRELIMINARY VALIDATION REPORT

Continued from previous page...

Mol	Chain	Length	Quality of chain
4	F	394	19% 86% 12% •

The following table lists non-polymeric compounds, carbohydrate monomers and non-standard residues in protein, DNA, RNA chains that are outliers for geometric or electron-density-fit criteria:

Mol	Type	Chain	Res	Chirality	Geometry	Clashes	Electron density
7	GOL	H	12	-	-	-	X
8	FMT	J	2	-	-	-	X
8	FMT	J	21	-	-	-	X
8	FMT	J	22	-	-	X	-

PRELIMINARY VALIDATION REPORT

2 Entry composition (i)

There are 9 unique types of molecules in this entry. The entry contains 20411 atoms, of which 0 are hydrogens and 0 are deuteriums.

In the tables below, the ZeroOcc column contains the number of atoms modelled with zero occupancy, the AltConf column contains the number of residues with at least one atom in alternate conformation and the Trace column contains the number of residues modelled with at most 2 atoms.

- Molecule 1 is a protein called Cytochrome P-450.

Mol	Chain	Residues	Atoms					ZeroOcc	AltConf	Trace
1	A	396	Total 3270	C 2066	N 594	O 596	S 14	0	27	0
1	B	396	Total 3231	C 2041	N 583	O 593	S 14	0	22	0
1	D	396	Total 3294	C 2081	N 597	O 603	S 13	0	29	0

There are 3 discrepancies between the modelled and reference sequences:

Chain	Residue	Modelled	Actual	Comment	Reference
A	89	TYR	GLU	engineered mutation	UNP Q59819
B	89	TYR	GLU	engineered mutation	UNP Q59819
D	89	TYR	GLU	engineered mutation	UNP Q59819

- Molecule 2 is a protein called Cytochrome P-450.

Mol	Chain	Residues	Atoms					ZeroOcc	AltConf	Trace
2	C	398	Total 3171	C 2000	N 573	O 585	S 13	0	10	0

There is a discrepancy between the modelled and reference sequences:

Chain	Residue	Modelled	Actual	Comment	Reference
C	89	TYR	GLU	engineered mutation	UNP Q59819

- Molecule 3 is a protein called Cytochrome P-450.

Mol	Chain	Residues	Atoms					ZeroOcc	AltConf	Trace
3	E	390	Total 3183	C 2006	N 573	O 589	S 15	0	18	0

There is a discrepancy between the modelled and reference sequences:

PRELIMINARY VALIDATION REPORT

Chain	Residue	Modelled	Actual	Comment	Reference
E	89	TYR	GLU	engineered mutation	UNP Q59819

- Molecule 4 is a protein called Cytochrome P-450.

Mol	Chain	Residues	Atoms					ZeroOcc	AltConf	Trace
			Total	C	N	O	S			
4	F	394	3284	2089	589	593	13	0	33	0

There is a discrepancy between the modelled and reference sequences:

Chain	Residue	Modelled	Actual	Comment	Reference
F	89	TYR	GLU	engineered mutation	UNP Q59819

- Molecule 5 is PROTOPORPHYRIN IX CONTAINING FE (three-letter code: HEM) (formula: $C_{34}H_{32}FeN_4O_4$).

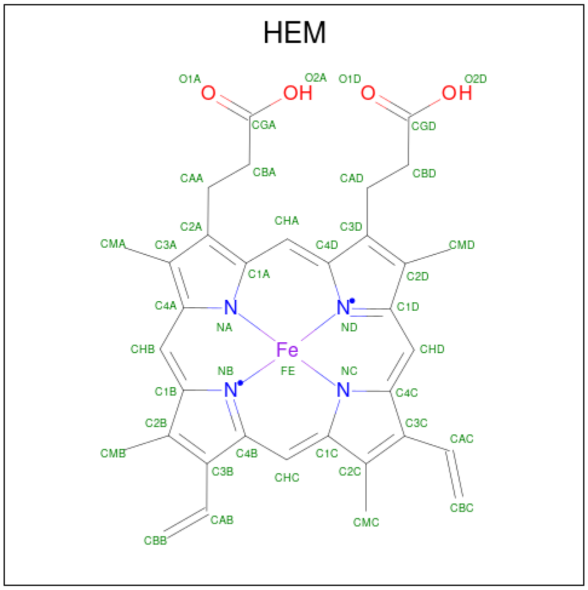

Mol	Chain	Residues	Atoms					ZeroOcc	AltConf
5	A	1	Total 43	C 34	Fe 1	N 4	O 4	0	0
5	B	1	Total 43	C 34	Fe 1	N 4	O 4	0	0
5	C	1	Total 43	C 34	Fe 1	N 4	O 4	0	0
5	D	1	Total 43	C 34	Fe 1	N 4	O 4	0	0
5	E	1	Total 43	C 34	Fe 1	N 4	O 4	0	0

Continued on next page...

PRELIMINARY VALIDATION REPORT

Continued from previous page...

Mol	Chain	Residues	Atoms	ZeroOcc	AltConf
5	F	1	Total C Fe N O<br>43 34 1 4 4	0	0

- Molecule 6 is 6-DEOXYERYTHRONOLIDE B (three-letter code: DEB) (formula: $C_{21}H_{38}O_6$).

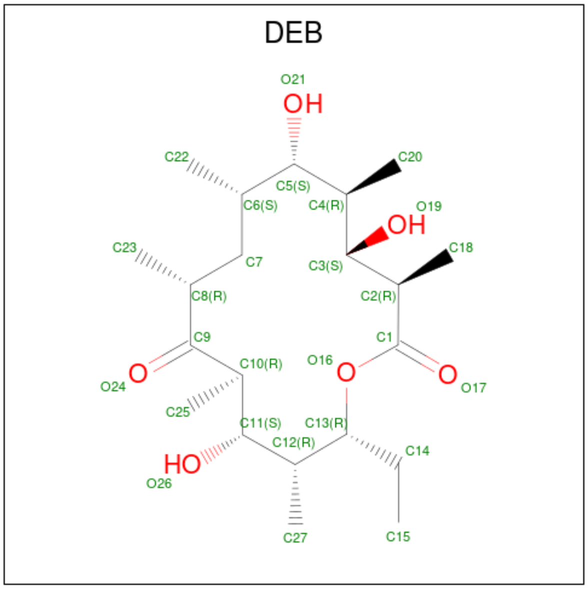

Mol	Chain	Residues	Atoms	ZeroOcc	AltConf
6	A	1	Total C O<br>27 21 6	0	0
6	B	1	Total C O<br>27 21 6	0	0
6	C	1	Total C O<br>27 21 6	0	0
6	D	1	Total C O<br>27 21 6	0	0
6	E	1	Total C O<br>27 21 6	0	0
6	F	1	Total C O<br>27 21 6	0	0

- Molecule 7 is GLYCEROL (three-letter code: GOL) (formula: $C_3H_8O_3$).

PRELIMINARY VALIDATION REPORT

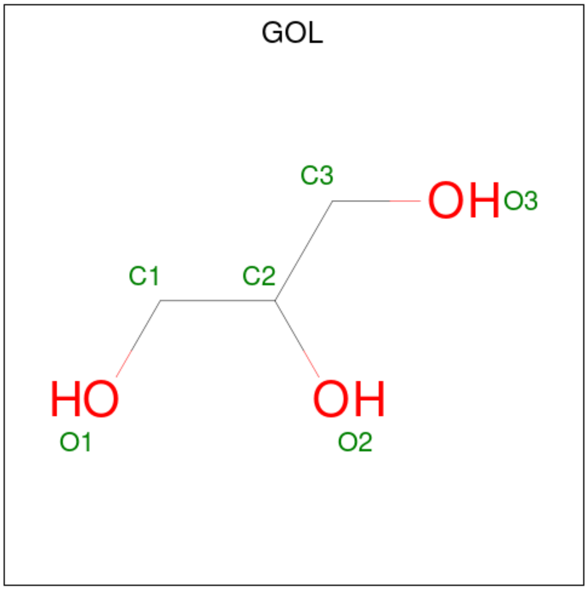

Mol	Chain	Residues	Atoms	ZeroOcc	AltConf
7	H	1	Total C O<br>6 3 3	0	0
7	H	1	Total C O<br>6 3 3	0	0
7	H	1	Total C O<br>6 3 3	0	0
7	H	1	Total C O<br>6 3 3	0	0
7	H	1	Total C O<br>6 3 3	0	0
7	H	1	Total C O<br>6 3 3	0	0
7	H	1	Total C O<br>6 3 3	0	0
7	H	1	Total C O<br>6 3 3	0	0
7	H	1	Total C O<br>6 3 3	0	0
7	H	1	Total C O<br>6 3 3	0	0
7	H	1	Total C O<br>6 3 3	0	0
7	H	1	Total C O<br>6 3 3	0	0
7	H	1	Total C O<br>6 3 3	0	0
7	H	1	Total C O<br>6 3 3	0	0

PRELIMINARY VALIDATION REPORT

- Molecule 8 is FORMIC ACID (three-letter code: FMT) (formula: CH_2O_2).

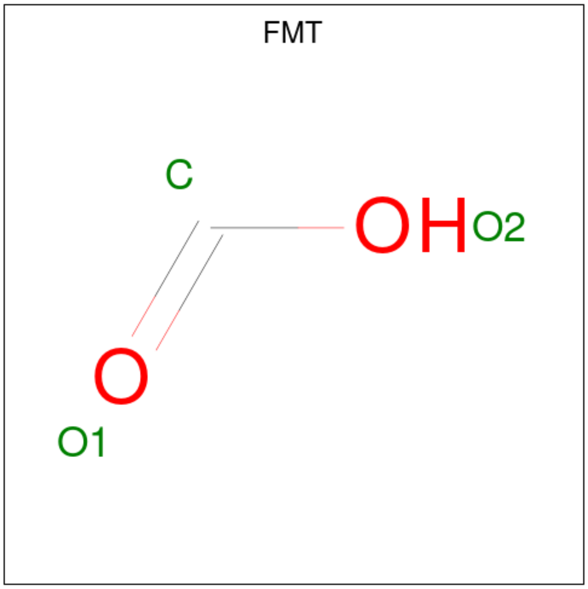

Mol	Chain	Residues	Atoms	ZeroOcc	AltConf
8	J	1	Total C O<br>3 1 2	0	0
8	J	1	Total C O<br>3 1 2	0	0
8	J	1	Total C O<br>3 1 2	0	0
8	J	1	Total C O<br>3 1 2	0	0
8	J	1	Total C O<br>3 1 2	0	0
8	J	1	Total C O<br>3 1 2	0	0
8	J	1	Total C O<br>3 1 2	0	0
8	J	1	Total C O<br>3 1 2	0	0
8	J	1	Total C O<br>3 1 2	0	0
8	J	1	Total C O<br>3 1 2	0	0
8	J	1	Total C O<br>3 1 2	0	0
8	J	1	Total C O<br>3 1 2	0	0
8	J	1	Total C O<br>3 1 2	0	0

Continued on next page...

PRELIMINARY VALIDATION REPORT

Continued from previous page...

Mol	Chain	Residues	Atoms	ZeroOcc	AltConf
8	J	1	Total C O<br>3 1 2	0	0
8	J	1	Total C O<br>3 1 2	0	0
8	J	1	Total C O<br>3 1 2	0	0
8	J	1	Total C O<br>3 1 2	0	0
8	J	1	Total C O<br>3 1 2	0	0
8	J	1	Total C O<br>3 1 2	0	0
8	J	1	Total C O<br>3 1 2	0	0
8	J	1	Total C O<br>3 1 2	0	0
8	J	1	Total C O<br>3 1 2	0	0
8	J	1	Total C O<br>3 1 2	0	0
8	J	1	Total C O<br>3 1 2	0	0
8	J	1	Total C O<br>3 1 2	0	0
8	J	1	Total C O<br>3 1 2	0	0
8	J	1	Total C O<br>3 1 2	0	0
8	J	1	Total C O<br>3 1 2	0	0
8	J	1	Total C O<br>3 1 2	0	0
8	J	1	Total C O<br>3 1 2	0	0
8	J	1	Total C O<br>3 1 2	0	0
8	J	1	Total C O<br>3 1 2	0	0
8	J	1	Total C O<br>3 1 2	0	0

- Molecule 9 is water.

PRELIMINARY VALIDATION REPORT

Mol	Chain	Residues	Atoms		ZeroOcc	AltConf
			Total	O		
9	G	375	375	375	0	0

PRELIMINARY VALIDATION REPORT

3 Residue-property plots (i)

These plots are drawn for all protein, RNA, DNA and oligosaccharide chains in the entry. The first graphic for a chain summarises the proportions of the various outlier classes displayed in the second graphic. The second graphic shows the sequence view annotated by issues in geometry and electron density. Residues are color-coded according to the number of geometric quality criteria for which they contain at least one outlier: green = 0, yellow = 1, orange = 2 and red = 3 or more. A red dot above a residue indicates a poor fit to the electron density (RSRZ > 2). Stretches of 2 or more consecutive residues without any outlier are shown as a green connector. Residues present in the sample, but not in the model, are shown in grey.

- Molecule 1: Cytochrome P-450

Chain A:

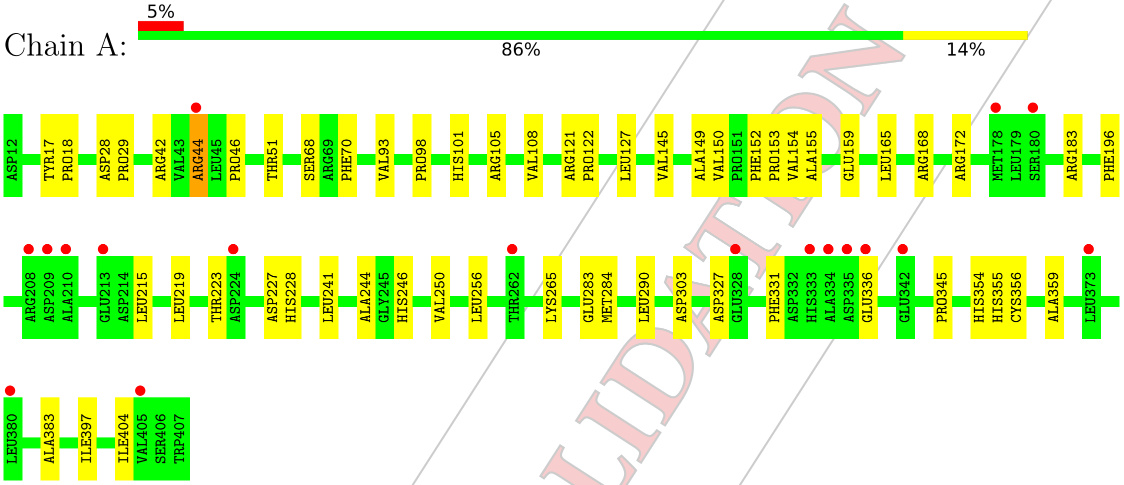

- Molecule 1: Cytochrome P-450

Chain B:

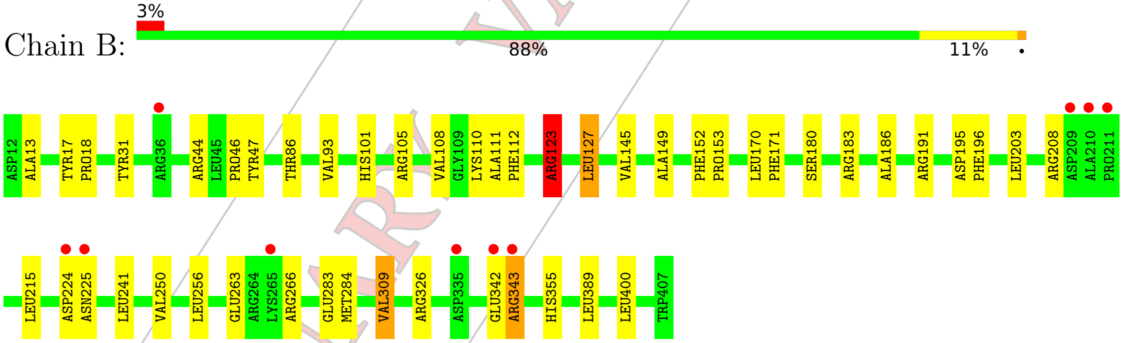

- Molecule 1: Cytochrome P-450

Chain D:

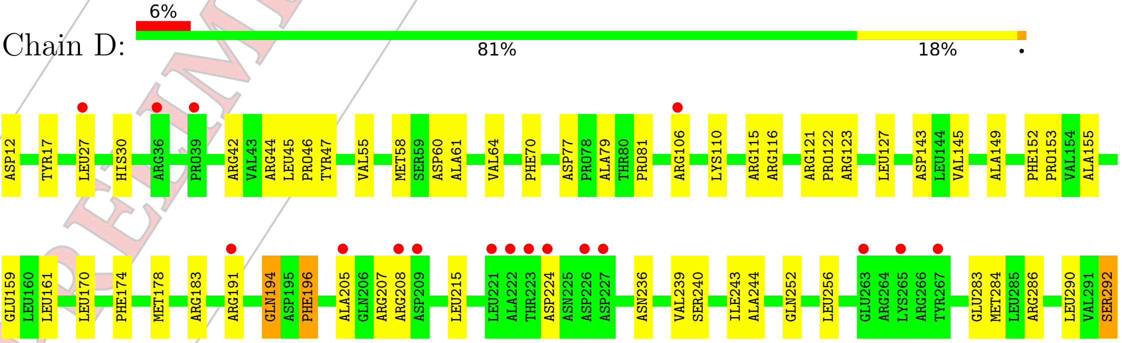

PRELIMINARY VALIDATION REPORT

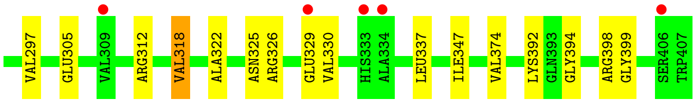

• Molecule 2: Cytochrome P-450

Chain C:

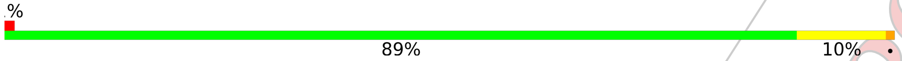

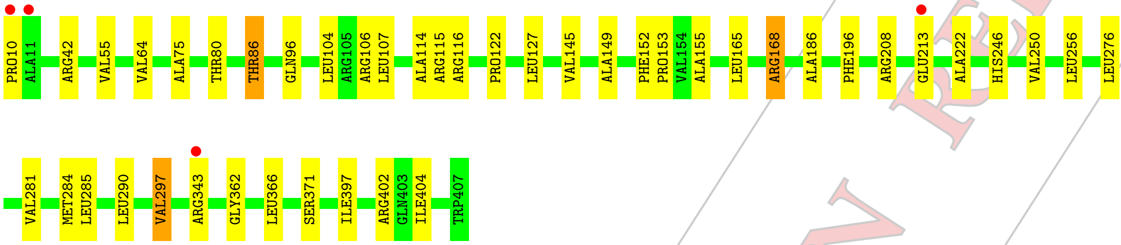

• Molecule 3: Cytochrome P-450

Chain E:

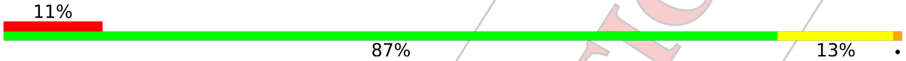

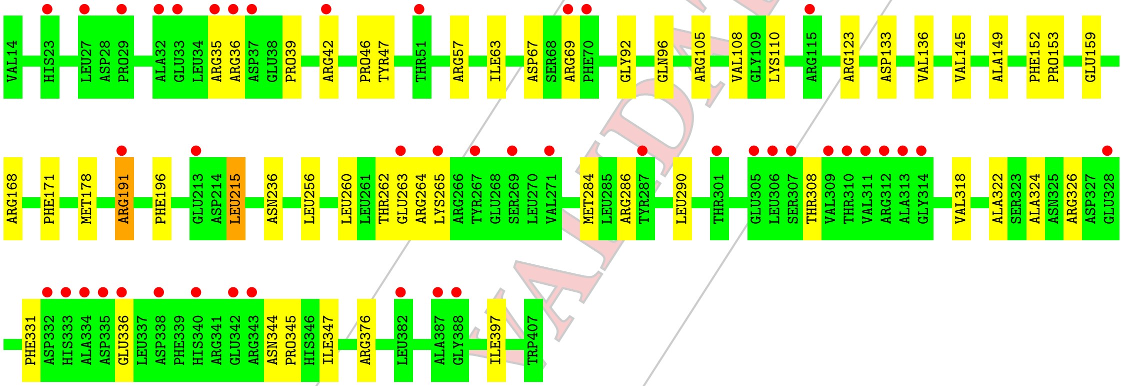

• Molecule 4: Cytochrome P-450

Chain F:

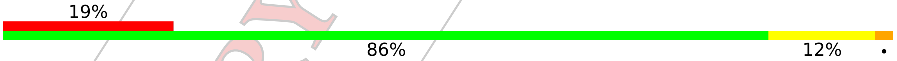

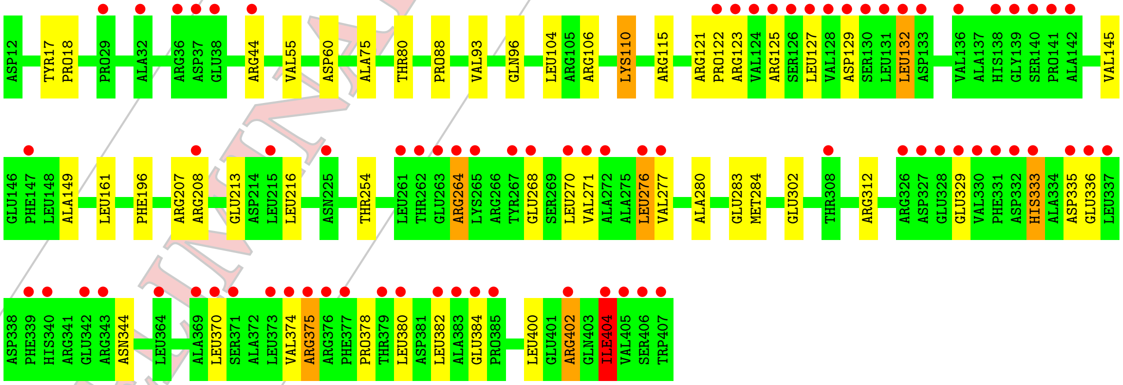

PRELIMINARY VALIDATION REPORT

4 Data and refinement statistics (i)

Property	Value	Source
Space group	C 1 2 1	Depositor
Cell constants<br>a, b, c, α, β, γ	248.13Å 111.12Å 160.54Å<br>90.00° 129.67° 90.00°	Depositor
Resolution (Å)	48.07 – 2.44<br>48.02 – 2.44	Depositor<br>EDS
% Data completeness<br>(in resolution range)	99.0 (48.07-2.44)<br>99.1 (48.02-2.44)	Depositor<br>EDS
R_{merge}	0.05	Depositor
R_{sym}	(Not available)	Depositor
$< I/\sigma(I) >$ [1]	2.13 (at 2.45Å)	Xtriage
Refinement program	REFMAC 5.8.0267	Depositor
R, R_{free}	0.187 , 0.243<br>0.200 , 0.248	Depositor<br>DCC
R_{free} test set	5991 reflections (4.86%)	wwPDB-VP
Wilson B-factor ($Å^2$)	62.2	Xtriage
Anisotropy	0.317	Xtriage
Bulk solvent k_{sol}(e/$Å^3$), B_{sol}($Å^2$)	(Not available) , (Not available)	EDS
L-test for twinning[2]	$< \|L\| > = 0.51$, $< L^2 > = 0.34$	Xtriage
Estimated twinning fraction	0.004 for -h-2*l,-k,l	Xtriage
F_o,F_c correlation	0.96	EDS
Total number of atoms	20411	wwPDB-VP
Average B, all atoms ($Å^2$)	64.0	wwPDB-VP

Xtriage's analysis on translational NCS is as follows: *The analyses of the Patterson function reveals a significant off-origin peak that is 38.21 % of the origin peak, indicating pseudo-translational symmetry. The chance of finding a peak of this or larger height randomly in a structure without pseudo-translational symmetry is equal to 3.8145e-04. The detected translational NCS is most likely also responsible for the elevated intensity ratio.*

[1] Intensities estimated from amplitudes.

[2] Theoretical values of $< |L| >$, $< L^2 >$ for acentric reflections are 0.5, 0.333 respectively for untwinned datasets, and 0.375, 0.2 for perfectly twinned datasets.

PRELIMINARY VALIDATION REPORT

5 Model quality (i)

5.1 Standard geometry (i)

Bond lengths and bond angles in the following residue types are not validated in this section: HEM, FMT, GOL, DEB

The Z score for a bond length (or angle) is the number of standard deviations the observed value is removed from the expected value. A bond length (or angle) with $|Z| > 5$ is considered an outlier worth inspection. RMSZ is the root-mean-square of all Z scores of the bond lengths (or angles).

Mol	Chain	Bond lengths		Bond angles	
		RMSZ	#$|Z|$ >5	RMSZ	#$|Z|$ >5
1	A	0.78	12/3417 (0.4%)	0.93	16/4641 (0.3%)
1	B	0.78	10/3368 (0.3%)	0.90	12/4577 (0.3%)
1	D	0.83	16/3439 (0.5%)	0.94	16/4671 (0.3%)
2	C	0.74	6/3270 (0.2%)	0.87	8/4447 (0.2%)
3	E	0.71	6/3279 (0.2%)	0.84	8/4454 (0.2%)
4	F	0.97	34/3450 (1.0%)	1.07	44/4686 (0.9%)
All	All	0.81	84/20223 (0.4%)	0.93	104/27476 (0.4%)

Chiral center outliers are detected by calculating the chiral volume of a chiral center and verifying if the center is modelled as a planar moiety or with the opposite hand.A planarity outlier is detected by checking planarity of atoms in a peptide group, atoms in a mainchain group or atoms of a sidechain that are expected to be planar.

Mol	Chain	#Chirality outliers	#Planarity outliers
3	E	0	5

All (84) bond length outliers are listed below:

Mol	Chain	Res	Type	Atoms	Z	Observed(Å)	Ideal(Å)
1	B	224[A]	ASP	C-O	7.45	1.37	1.23
1	B	224[B]	ASP	C-O	7.45	1.37	1.23
4	F	336[A]	GLU	C-O	7.28	1.37	1.23
4	F	336[B]	GLU	C-O	7.28	1.37	1.23
4	F	380[A]	LEU	C-O	7.25	1.37	1.23
4	F	380[B]	LEU	C-O	7.25	1.37	1.23
4	F	208[A]	ARG	C-O	7.24	1.37	1.23
4	F	208[B]	ARG	C-O	7.24	1.37	1.23
1	B	342[A]	GLU	C-O	7.21	1.37	1.23
1	B	342[B]	GLU	C-O	7.21	1.37	1.23
1	B	225[A]	ASN	C-O	7.21	1.37	1.23
1	B	225[B]	ASN	C-O	7.21	1.37	1.23

Continued on next page...

PRELIMINARY VALIDATION REPORT

Continued from previous page...

Mol	Chain	Res	Type	Atoms	Z	Observed(Å)	Ideal(Å)
4	F	270[A]	LEU	C-O	7.13	1.36	1.23
4	F	270[B]	LEU	C-O	7.13	1.36	1.23
1	A	228[A]	HIS	C-O	7.12	1.36	1.23
1	A	228[B]	HIS	C-O	7.12	1.36	1.23
1	A	227[A]	ASP	C-O	7.11	1.36	1.23
1	A	227[B]	ASP	C-O	7.11	1.36	1.23
1	D	329[A]	GLU	C-O	7.08	1.36	1.23
1	D	329[B]	GLU	C-O	7.08	1.36	1.23
4	F	404[A]	ILE	C-O	7.07	1.36	1.23
4	F	404[B]	ILE	C-O	7.07	1.36	1.23
4	F	333[A]	HIS	C-O	7.05	1.36	1.23
4	F	333[B]	HIS	C-O	7.05	1.36	1.23
4	F	44[A]	ARG	C-O	7.05	1.36	1.23
4	F	44[B]	ARG	C-O	7.05	1.36	1.23
4	F	213[A]	GLU	C-O	7.02	1.36	1.23
4	F	213[B]	GLU	C-O	7.02	1.36	1.23
4	F	125[A]	ARG	C-O	7.00	1.36	1.23
4	F	125[B]	ARG	C-O	7.00	1.36	1.23
1	B	343[A]	ARG	C-O	7.00	1.36	1.23
1	B	343[B]	ARG	C-O	7.00	1.36	1.23
2	C	208[A]	ARG	C-O	6.99	1.36	1.23
2	C	208[B]	ARG	C-O	6.99	1.36	1.23
4	F	402[A]	ARG	C-O	6.99	1.36	1.23
4	F	402[B]	ARG	C-O	6.99	1.36	1.23
4	F	329[A]	GLU	C-O	6.95	1.36	1.23
4	F	329[B]	GLU	C-O	6.95	1.36	1.23
1	D	42[A]	ARG	C-O	6.95	1.36	1.23
1	D	42[B]	ARG	C-O	6.95	1.36	1.23
3	E	336[A]	GLU	C-O	6.94	1.36	1.23
3	E	336[B]	GLU	C-O	6.94	1.36	1.23
4	F	276[A]	LEU	C-O	6.90	1.36	1.23
4	F	276[B]	LEU	C-O	6.90	1.36	1.23
1	D	115[A]	ARG	C-O	6.89	1.36	1.23
1	D	115[B]	ARG	C-O	6.89	1.36	1.23
1	A	336[A]	GLU	C-O	6.89	1.36	1.23
1	A	336[B]	GLU	C-O	6.89	1.36	1.23
1	D	106[A]	ARG	C-O	6.88	1.36	1.23
1	D	106[B]	ARG	C-O	6.88	1.36	1.23
1	D	30[A]	HIS	C-O	6.78	1.36	1.23
1	D	30[B]	HIS	C-O	6.78	1.36	1.23
4	F	132[A]	LEU	C-O	6.77	1.36	1.23
4	F	132[B]	LEU	C-O	6.77	1.36	1.23

Continued on next page...

PRELIMINARY VALIDATION REPORT

Continued from previous page...

Mol	Chain	Res	Type	Atoms	Z	Observed(Å)	Ideal(Å)
2	C	213[A]	GLU	C-O	6.77	1.36	1.23
2	C	213[B]	GLU	C-O	6.77	1.36	1.23
4	F	123[A]	ARG	C-O	6.77	1.36	1.23
4	F	123[B]	ARG	C-O	6.77	1.36	1.23
1	A	44[A]	ARG	C-O	6.76	1.36	1.23
1	A	44[B]	ARG	C-O	6.76	1.36	1.23
1	A	265[A]	LYS	C-O	6.74	1.36	1.23
1	A	265[B]	LYS	C-O	6.74	1.36	1.23
1	D	305[A]	GLU	C-O	6.73	1.36	1.23
1	D	305[B]	GLU	C-O	6.73	1.36	1.23
4	F	110[A]	LYS	C-O	6.68	1.36	1.23
4	F	110[B]	LYS	C-O	6.68	1.36	1.23
4	F	127[A]	LEU	C-O	6.65	1.35	1.23
4	F	127[B]	LEU	C-O	6.65	1.35	1.23
1	B	123[A]	ARG	C-O	6.64	1.35	1.23
1	B	123[B]	ARG	C-O	6.64	1.35	1.23
3	E	265[A]	LYS	C-O	6.59	1.35	1.23
3	E	265[B]	LYS	C-O	6.59	1.35	1.23
1	A	183[A]	ARG	C-O	6.58	1.35	1.23
1	A	183[B]	ARG	C-O	6.58	1.35	1.23
1	D	194[A]	GLN	C-O	6.45	1.35	1.23
1	D	194[B]	GLN	C-O	6.45	1.35	1.23
3	E	110[A]	LYS	C-O	6.42	1.35	1.23
3	E	110[B]	LYS	C-O	6.42	1.35	1.23
2	C	168[A]	ARG	C-O	6.20	1.35	1.23
2	C	168[B]	ARG	C-O	6.20	1.35	1.23
1	D	110[A]	LYS	CA-C	6.04	1.68	1.52
1	D	110[B]	LYS	CA-C	6.04	1.68	1.52
4	F	374[A]	VAL	CA-C	5.17	1.66	1.52
4	F	374[B]	VAL	CA-C	5.17	1.66	1.52

All (104) bond angle outliers are listed below:

Mol	Chain	Res	Type	Atoms	Z	Observed(^{o})	Ideal(^{o})
4	F	375[A]	ARG	CB-CA-C	-8.93	92.53	110.40
4	F	375[B]	ARG	CB-CA-C	-8.93	92.53	110.40
2	C	213[A]	GLU	CA-C-O	6.35	133.44	120.10
2	C	213[B]	GLU	CA-C-O	6.35	133.44	120.10
1	A	183[A]	ARG	CA-C-O	6.08	132.87	120.10
1	A	183[B]	ARG	CA-C-O	6.08	132.87	120.10
4	F	333[A]	HIS	CA-C-O	6.07	132.85	120.10
4	F	333[B]	HIS	CA-C-O	6.07	132.85	120.10

Continued on next page...

PRELIMINARY VALIDATION REPORT

Continued from previous page...

Mol	Chain	Res	Type	Atoms	Z	Observed(°)	Ideal(°)
1	D	305[A]	GLU	CA-C-O	6.07	132.84	120.10
1	D	305[B]	GLU	CA-C-O	6.07	132.84	120.10
1	D	194[A]	GLN	CA-C-O	6.04	132.80	120.10
1	D	194[B]	GLN	CA-C-O	6.04	132.80	120.10
4	F	276[A]	LEU	CA-C-O	6.00	132.70	120.10
4	F	276[B]	LEU	CA-C-O	6.00	132.70	120.10
2	C	208[A]	ARG	CA-C-O	6.00	132.69	120.10
2	C	208[B]	ARG	CA-C-O	6.00	132.69	120.10
4	F	329[A]	GLU	CA-C-O	5.97	132.63	120.10
4	F	329[B]	GLU	CA-C-O	5.97	132.63	120.10
4	F	213[A]	GLU	CA-C-O	5.95	132.60	120.10
4	F	213[B]	GLU	CA-C-O	5.95	132.60	120.10
2	C	168[A]	ARG	CA-C-O	5.94	132.57	120.10
2	C	168[B]	ARG	CA-C-O	5.94	132.57	120.10
1	A	228[A]	HIS	CA-C-O	5.93	132.56	120.10
1	A	228[B]	HIS	CA-C-O	5.93	132.56	120.10
1	A	227[A]	ASP	CA-C-O	5.91	132.52	120.10
1	A	227[B]	ASP	CA-C-O	5.91	132.52	120.10
1	D	106[A]	ARG	CA-C-O	5.91	132.51	120.10
1	D	106[B]	ARG	CA-C-O	5.91	132.51	120.10
4	F	208[A]	ARG	CA-C-O	5.89	132.46	120.10
4	F	208[B]	ARG	CA-C-O	5.89	132.46	120.10
4	F	123[A]	ARG	CA-C-O	5.87	132.43	120.10
4	F	123[B]	ARG	CA-C-O	5.87	132.43	120.10
1	B	225[A]	ASN	CA-C-O	5.84	132.36	120.10
1	B	225[B]	ASN	CA-C-O	5.84	132.36	120.10
1	A	44[A]	ARG	CA-C-O	5.82	132.32	120.10
1	A	44[B]	ARG	CA-C-O	5.82	132.32	120.10
1	A	265[A]	LYS	CA-C-O	5.79	132.26	120.10
1	A	265[B]	LYS	CA-C-O	5.79	132.26	120.10
1	B	123[A]	ARG	CA-C-O	5.75	132.18	120.10
1	B	123[B]	ARG	CA-C-O	5.75	132.18	120.10
4	F	127[A]	LEU	CA-C-O	5.75	132.17	120.10
4	F	127[B]	LEU	CA-C-O	5.75	132.17	120.10
3	E	336[A]	GLU	CA-C-O	5.74	132.16	120.10
3	E	336[B]	GLU	CA-C-O	5.74	132.16	120.10
4	F	110[A]	LYS	CA-C-O	5.74	132.15	120.10
4	F	110[B]	LYS	CA-C-O	5.74	132.15	120.10
4	F	336[A]	GLU	CA-C-O	5.72	132.11	120.10
4	F	336[B]	GLU	CA-C-O	5.72	132.11	120.10
4	F	125[A]	ARG	CA-C-O	5.70	132.07	120.10
4	F	125[B]	ARG	CA-C-O	5.70	132.07	120.10

Continued on next page...

PRELIMINARY VALIDATION REPORT

Continued from previous page...

Mol	Chain	Res	Type	Atoms	Z	Observed(°)	Ideal(°)
4	F	44[A]	ARG	CA-C-O	5.68	132.04	120.10
4	F	44[B]	ARG	CA-C-O	5.68	132.04	120.10
1	D	30[A]	HIS	CA-C-O	5.68	132.02	120.10
1	D	30[B]	HIS	CA-C-O	5.68	132.02	120.10
3	E	265[A]	LYS	CA-C-O	5.67	132.01	120.10
3	E	265[B]	LYS	CA-C-O	5.67	132.01	120.10
4	F	270[A]	LEU	CA-C-O	5.67	132.01	120.10
4	F	270[B]	LEU	CA-C-O	5.67	132.01	120.10
1	D	329[A]	GLU	CA-C-O	5.64	131.96	120.10
1	D	329[B]	GLU	CA-C-O	5.64	131.96	120.10
1	B	342[A]	GLU	CA-C-O	5.63	131.93	120.10
1	B	342[B]	GLU	CA-C-O	5.63	131.93	120.10
1	A	336[A]	GLU	CA-C-O	5.61	131.89	120.10
1	A	336[B]	GLU	CA-C-O	5.61	131.89	120.10
1	B	343[A]	ARG	CA-C-O	5.60	131.86	120.10
1	B	343[B]	ARG	CA-C-O	5.60	131.86	120.10
3	E	110[A]	LYS	CA-C-O	5.60	131.86	120.10
3	E	110[B]	LYS	CA-C-O	5.60	131.86	120.10
4	F	132[A]	LEU	CA-C-O	5.59	131.84	120.10
4	F	132[B]	LEU	CA-C-O	5.59	131.84	120.10
4	F	402[A]	ARG	CA-C-O	5.58	131.82	120.10
4	F	402[B]	ARG	CA-C-O	5.58	131.82	120.10
1	A	227[A]	ASP	O-C-N	-5.50	113.90	122.70
1	A	227[B]	ASP	O-C-N	-5.50	113.90	122.70
1	D	110[A]	LYS	CA-C-O	5.49	131.63	120.10
1	D	110[B]	LYS	CA-C-O	5.49	131.63	120.10
4	F	380[A]	LEU	CA-C-O	5.39	131.43	120.10
4	F	380[B]	LEU	CA-C-O	5.39	131.43	120.10
1	D	42[A]	ARG	CA-C-O	5.33	131.29	120.10
1	D	42[B]	ARG	CA-C-O	5.33	131.29	120.10
1	D	115[A]	ARG	CA-C-O	5.32	131.26	120.10
1	D	115[B]	ARG	CA-C-O	5.32	131.26	120.10
1	B	224[A]	ASP	CA-C-O	5.28	131.19	120.10
1	B	224[B]	ASP	CA-C-O	5.28	131.19	120.10
2	C	213[A]	GLU	O-C-N	-5.23	114.33	122.70
2	C	213[B]	GLU	O-C-N	-5.23	114.33	122.70
4	F	329[A]	GLU	O-C-N	-5.22	114.35	122.70
4	F	329[B]	GLU	O-C-N	-5.22	114.35	122.70
4	F	276[A]	LEU	O-C-N	-5.16	114.44	122.70
4	F	276[B]	LEU	O-C-N	-5.16	114.44	122.70
1	B	225[A]	ASN	O-C-N	-5.15	114.46	122.70
1	B	225[B]	ASN	O-C-N	-5.15	114.46	122.70

Continued on next page...

PRELIMINARY VALIDATION REPORT

Continued from previous page...

Mol	Chain	Res	Type	Atoms	Z	Observed(°)	Ideal(°)
4	F	404[A]	ILE	CA-C-O	5.15	130.91	120.10
4	F	404[B]	ILE	CA-C-O	5.15	130.91	120.10
4	F	374[A]	VAL	CA-C-O	5.14	130.90	120.10
4	F	374[B]	VAL	CA-C-O	5.14	130.90	120.10
4	F	333[A]	HIS	CA-C-N	-5.05	106.08	117.20
4	F	333[B]	HIS	CA-C-N	-5.05	106.08	117.20
1	A	228[A]	HIS	O-C-N	-5.04	114.64	122.70
1	A	228[B]	HIS	O-C-N	-5.04	114.64	122.70
4	F	336[A]	GLU	O-C-N	-5.01	114.68	122.70
4	F	336[B]	GLU	O-C-N	-5.01	114.68	122.70
3	E	336[A]	GLU	O-C-N	-5.01	114.69	122.70
3	E	336[B]	GLU	O-C-N	-5.01	114.69	122.70

There are no chirality outliers.

All (5) planarity outliers are listed below:

Mol	Chain	Res	Type	Group
3	E	178[B]	MET	Mainchain
3	E	191[A]	ARG	Mainchain
3	E	262	THR	Mainchain
3	E	263[B]	GLU	Mainchain

5.2 Too-close contacts (i)

In the following table, the Non-H and H(model) columns list the number of non-hydrogen atoms and hydrogen atoms in the chain respectively. The H(added) column lists the number of hydrogen atoms added and optimized by MolProbity. The Clashes column lists the number of clashes within the asymmetric unit, whereas Symm-Clashes lists symmetry-related clashes.

Mol	Chain	Non-H	H(model)	H(added)	Clashes	Symm-Clashes
1	A	3270	0	3309	36	0
1	B	3231	0	3262	37	0
1	D	3294	0	3340	45	0
2	C	3171	0	3187	23	0
3	E	3183	0	3188	34	0
4	F	3284	0	3384	29	0
5	A	43	0	30	5	0
5	B	43	0	30	4	0
5	C	43	0	30	1	0
5	D	43	0	30	5	0
5	E	43	0	30	2	0

Continued on next page...

PRELIMINARY VALIDATION REPORT

Continued from previous page...

Mol	Chain	Non-H	H(model)	H(added)	Clashes	Symm-Clashes
5	F	43	0	30	3	0
6	A	27	0	38	0	0
6	B	27	0	38	0	0
6	C	27	0	38	0	0
6	D	27	0	38	0	0
6	E	27	0	38	0	0
6	F	27	0	38	0	0
7	H	84	0	112	9	0
8	J	99	0	33	3	0
9	G	375	0	0	11	0
All	All	20411	0	20223	217	0

The all-atom clashscore is defined as the number of clashes found per 1000 atoms (including hydrogen atoms). The all-atom clashscore for this structure is 5.

All (217) close contacts within the same asymmetric unit are listed below, sorted by their clash magnitude.

Atom-1	Atom-2	Interatomic distance (Å)	Clash overlap (Å)
4:F:384[B]:GLU:CD	4:F:402[B]:ARG:HH12	1.19	1.43
4:F:384[B]:GLU:OE1	4:F:402[B]:ARG:NH1	1.69	1.23
4:F:384[B]:GLU:CD	4:F:402[B]:ARG:NH1	1.98	1.16
3:E:256:LEU:HD22	3:E:284[B]:MET:HB3	1.51	0.90
3:E:256:LEU:CD2	3:E:284[B]:MET:HB3	2.02	0.89
1:B:101[A]:HIS:CE1	1:B:105:ARG:HD2	2.11	0.85
3:E:256:LEU:HD22	3:E:284[A]:MET:HB3	1.62	0.80
1:A:172:ARG:HD2	9:G:38:HOH:O	1.83	0.78
3:E:256:LEU:HD22	3:E:284[B]:MET:CB	2.14	0.78
4:F:384[B]:GLU:OE2	4:F:402[B]:ARG:NH1	2.13	0.76
1:A:108:VAL:HG22	1:A:215:LEU:HD22	1.70	0.74
4:F:264[A]:ARG:HD3	4:F:268[A]:GLU:OE2	1.88	0.73
3:E:256:LEU:CD2	3:E:284[A]:MET:HB3	2.17	0.73
5:B:501:HEM:HMC1	5:B:501:HEM:HBC2	1.70	0.73
1:A:98:PRO:HD2	7:H:12:GOL:H11	1.71	0.72
3:E:256:LEU:HD22	3:E:284[A]:MET:CB	2.16	0.70
1:B:108:VAL:HG13	1:B:215:LEU:HD22	1.72	0.70
2:C:256:LEU:HD22	2:C:284:MET:HB3	1.73	0.70
5:E:501:HEM:HHC	5:E:501:HEM:HBB2	1.74	0.69
5:F:501:HEM:HMB2	5:F:501:HEM:HBB2	1.75	0.69
1:D:152:PHE:HB3	1:D:153:PRO:HD3	1.77	0.67
1:D:143:ASP:OD2	9:G:348:HOH:O	2.13	0.67
7:H:8:GOL:H11	9:G:62:HOH:O	1.96	0.66

Continued on next page...

PRELIMINARY VALIDATION REPORT

Continued from previous page...

Atom-1	Atom-2	Interatomic distance (Å)	Clash overlap (Å)
1:B:256:LEU:HD22	1:B:284:MET:HB3	1.79	0.65
1:D:58:MET:HE3	1:D:347:ILE:HD13	1.79	0.65
1:A:327:ASP:OD2	9:G:227:HOH:O	2.14	0.64
1:D:17:TYR:O	1:D:46:PRO:HD3	1.96	0.64
1:B:46:PRO:HB2	1:B:47:TYR:CD1	2.33	0.64
1:A:70:PHE:O	7:H:12:GOL:H2	1.97	0.63
1:A:101[B]:HIS:CE1	1:A:354[B]:HIS:CD2	2.86	0.63
1:B:195:ASP:OD2	4:F:106[B]:ARG:NH2	2.31	0.62
1:D:64:VAL:HA	1:D:70:PHE:CD2	2.35	0.62
4:F:145:VAL:HA	4:F:149:ALA:HB3	1.81	0.62
1:B:110[B]:LYS:NZ	9:G:242:HOH:O	2.32	0.60
1:A:355[B]:HIS:CE1	8:J:22:FMT:O1	2.53	0.60
5:A:501:HEM:HBB2	5:A:501:HEM:HMB2	1.84	0.59
1:B:123[A]:ARG:NH1	1:B:127:LEU:HG	2.18	0.59
5:F:501:HEM:HBC2	5:F:501:HEM:HMC1	1.84	0.58
2:C:145:VAL:HA	2:C:149:ALA:HB3	1.86	0.58
3:E:236:ASN:HD21	7:H:5:GOL:H2	1.68	0.58
1:A:355[B]:HIS:HE1	8:J:22:FMT:O1	1.87	0.58
1:D:58:MET:HE1	1:D:347:ILE:HG21	1.86	0.57
1:D:322:ALA:O	1:D:326:ARG:HG2	2.05	0.57
1:D:145:VAL:HA	1:D:149:ALA:HB3	1.87	0.56
1:D:183:ARG:HG3	1:D:394:GLY:HA3	1.86	0.56
1:A:152:PHE:HB3	1:A:153:PRO:HD3	1.87	0.56
1:D:297:VAL:HG22	1:D:318:VAL:HG13	1.88	0.56
1:A:256:LEU:HD22	1:A:284:MET:HB3	1.87	0.56
1:D:392:LYS:HG3	1:D:399:GLY:O	2.06	0.56
4:F:277:VAL:HG13	4:F:370[B]:LEU:HD23	1.86	0.55
1:A:154:VAL:HG11	1:A:168[B]:ARG:HG2	1.88	0.55
1:B:46:PRO:HB2	1:B:47:TYR:CE1	2.42	0.55
3:E:133:ASP:O	3:E:136:VAL:HG22	2.06	0.55
4:F:88:PRO:HA	9:G:200:HOH:O	2.08	0.54
5:B:501:HEM:HBC2	5:B:501:HEM:CMC	2.35	0.54
4:F:280:ALA:O	4:F:284:MET:HG3	2.07	0.53
1:B:208:ARG:HB2	4:F:302:GLU:HG2	1.89	0.53
2:C:127:LEU:HD21	2:C:155:ALA:HB3	1.91	0.53
3:E:39:PRO:HD2	3:E:308:THR:HG21	1.91	0.52
3:E:322:ALA:O	3:E:326:ARG:HG2	2.09	0.52
1:D:252[B]:GLN:OE1	1:D:252[B]:GLN:HA	2.08	0.52
5:D:501:HEM:HBB2	5:D:501:HEM:HMB2	1.90	0.52
1:B:108:VAL:HG13	1:B:215:LEU:CD2	2.40	0.52
1:D:127:LEU:HD21	1:D:155:ALA:HB3	1.91	0.52

Continued on next page...

PRELIMINARY VALIDATION REPORT

Continued from previous page...

Atom-1	Atom-2	Interatomic distance (Å)	Clash overlap (Å)
1:A:150:VAL:O	1:A:154:VAL:HG23	2.10	0.52
1:D:191[A]:ARG:CZ	1:D:194[A]:GLN:HE22	2.23	0.52
5:D:501:HEM:HBC2	5:D:501:HEM:HMC2	1.92	0.52
1:D:77:ASP:OD2	1:D:79:ALA:HB3	2.11	0.51
1:D:174:PHE:HB3	1:D:196:PHE:CD2	2.46	0.51
1:D:240:SER:OG	7:H:4:GOL:H32	2.11	0.51
3:E:256:LEU:CD2	3:E:284[A]:MET:CB	2.84	0.51
3:E:133:ASP:OD1	3:E:376:ARG:NH2	2.35	0.51
4:F:96:GLN:HG3	4:F:104:LEU:HD12	1.92	0.51
2:C:86:THR:HG22	2:C:186:ALA:HA	1.92	0.50
1:D:292:SER:HB3	1:D:398:ARG:HE	1.76	0.50
2:C:343[A]:ARG:HG3	2:C:343[A]:ARG:HH11	1.76	0.50
1:B:17:TYR:HA	1:B:18:PRO:C	2.31	0.50
1:D:256:LEU:HD22	1:D:284:MET:HB3	1.93	0.50
1:D:152:PHE:CB	1:D:153:PRO:HD3	2.42	0.49
5:A:501:HEM:HMC2	5:A:501:HEM:HBC2	1.94	0.49
1:B:86:THR:HG22	1:B:186:ALA:HA	1.93	0.49
1:D:58:MET:HG2	1:D:330:VAL:HG21	1.93	0.49
3:E:290:LEU:O	3:E:397:ILE:HA	2.12	0.49
1:A:159[A]:GLU:HA	1:A:159[A]:GLU:OE2	2.11	0.49
3:E:260:LEU:HG	3:E:284[A]:MET:CE	2.43	0.49
3:E:286:ARG:HD3	3:E:344:ASN:OD1	2.13	0.49
4:F:283:GLU:HA	4:F:344:ASN:HD21	1.78	0.48
1:A:246:HIS:O	1:A:250:VAL:HG23	2.13	0.48
1:A:383:ALA:HB3	1:A:404:ILE:HG22	1.95	0.48
2:C:246:HIS:O	2:C:250:VAL:HG23	2.13	0.48
1:D:123[A]:ARG:CZ	1:D:159:GLU:HG3	2.44	0.48
3:E:256:LEU:O	3:E:284[A]:MET:HE1	2.14	0.48
1:A:145:VAL:HA	1:A:149:ALA:HB3	1.94	0.48
1:B:309:VAL:HG22	2:C:122:PRO:HB3	1.93	0.48
1:D:252[A]:GLN:OE1	1:D:252[A]:GLN:HA	2.12	0.48
4:F:75:ALA:HA	4:F:80:THR:HG21	1.94	0.48
1:B:13:ALA:HB2	2:C:114:ALA:HB1	1.96	0.48
1:D:58:MET:HE1	1:D:347:ILE:CG2	2.43	0.48
1:D:45:LEU:HD12	1:D:81:PRO:HB2	1.96	0.48
1:B:263:GLU:HB2	1:B:266:ARG:HD2	1.95	0.48
1:D:292:SER:HA	1:D:398:ARG:HE	1.77	0.48
4:F:17:TYR:HA	4:F:18:PRO:C	2.34	0.48
1:B:101[B]:HIS:NE2	1:B:105:ARG:HD2	2.28	0.48
3:E:35[B]:ARG:HG2	3:E:57:ARG:HG2	1.96	0.48
1:B:101[A]:HIS:CE1	1:B:105:ARG:CD	2.91	0.47

Continued on next page...

PRELIMINARY VALIDATION REPORT

Continued from previous page...

Atom-1	Atom-2	Interatomic distance (Å)	Clash overlap (Å)
1:B:108:VAL:HG11	1:B:241:LEU:HD11	1.96	0.47
3:E:63:ILE:O	3:E:67:ASP:HB2	2.14	0.47
1:B:101[B]:HIS:CE1	5:B:501:HEM:O2D	2.68	0.47
1:D:252[B]:GLN:HG2	1:D:290:LEU:HD13	1.96	0.47
1:A:17:TYR:HA	1:A:18:PRO:C	2.34	0.47
3:E:152:PHE:HB3	3:E:153:PRO:HD3	1.97	0.47
1:D:239:VAL:HG12	1:D:243:ILE:HD12	1.96	0.47
1:B:110[A]:LYS:HG3	1:B:111:ALA:N	2.30	0.47
1:D:58:MET:HE3	1:D:347:ILE:CD1	2.44	0.47
1:A:303:ASP:OD2	9:G:44:HOH:O	2.20	0.46
2:C:55:VAL:HG21	2:C:64:VAL:HG21	1.96	0.46
1:B:266:ARG:HG2	7:H:13:GOL:H11	1.98	0.46
1:A:127:LEU:HD21	1:A:155:ALA:HB3	1.97	0.46
5:D:501:HEM:HBB2	5:D:501:HEM:CMB	2.45	0.46
5:D:501:HEM:HBC2	5:D:501:HEM:CMC	2.46	0.46
3:E:105:ARG:HH21	5:E:501:HEM:HBD1	1.81	0.46
1:B:145:VAL:HA	1:B:149:ALA:HB3	1.98	0.46
7:H:8:GOL:H31	9:G:61:HOH:O	2.16	0.46
1:B:17:TYR:HH	1:B:31:TYR:HH	1.63	0.45
1:D:283:GLU:HG3	1:D:337:LEU:CD2	2.45	0.45
3:E:92:GLY:O	3:E:96:GLN:HG2	2.16	0.45
3:E:331:PHE:CE1	3:E:345:PRO:HD2	2.51	0.45
1:A:290:LEU:O	1:A:397:ILE:HA	2.17	0.45
1:D:337:LEU:HD23	1:D:337:LEU:HA	1.83	0.45
1:B:152:PHE:HB3	1:B:153:PRO:HD3	1.98	0.45
1:B:180:SER:HB2	1:B:183:ARG:HB2	1.99	0.45
2:C:75:ALA:HB1	2:C:297:VAL:HG22	1.99	0.45
1:B:108:VAL:HG12	1:B:112:PHE:CE2	2.51	0.45
2:C:75:ALA:HA	2:C:80:THR:HG21	1.99	0.45
2:C:152:PHE:HB3	2:C:153:PRO:HD3	1.98	0.45
1:B:389:LEU:HB3	1:B:400:LEU:HD21	1.99	0.44
1:D:121:ARG:N	1:D:122:PRO:CD	2.81	0.44
8:J:12:FMT:O1	9:G:319:HOH:O	2.21	0.44
2:C:96:GLN:HG3	2:C:104:LEU:HD12	2.00	0.44
1:D:27[B]:LEU:HD23	1:D:27[B]:LEU:HA	1.87	0.44
2:C:402:ARG:HD2	2:C:404:ILE:HD11	1.99	0.44
1:A:108:VAL:HG11	1:A:241:LEU:HD11	1.98	0.44
1:B:215:LEU:HD23	1:B:215:LEU:HA	1.88	0.44
3:E:123:ARG:NH2	3:E:159:GLU:OE2	2.48	0.44
1:D:58:MET:O	1:D:61:ALA:HB3	2.18	0.43
1:D:161:LEU:HD23	1:D:215:LEU:HB2	1.99	0.43

Continued on next page...

PRELIMINARY VALIDATION REPORT

Continued from previous page...

Atom-1	Atom-2	Interatomic distance (Å)	Clash overlap (Å)
3:E:260:LEU:HG	3:E:284[A]:MET:HE1	2.00	0.43
4:F:384[B]:GLU:HB2	4:F:404[B]:ILE:HG12	2.00	0.43
2:C:290:LEU:O	2:C:397:ILE:HA	2.18	0.43
1:A:244:ALA:HB1	5:A:501:HEM:C4C	2.54	0.43
1:A:283:GLU:HA	1:A:283:GLU:OE1	2.18	0.43
1:B:309:VAL:HG13	2:C:122:PRO:HA	1.99	0.43
4:F:254:THR:HB	4:F:400:LEU:HB2	2.01	0.43
1:A:121:ARG:N	1:A:122:PRO:CD	2.82	0.43
1:B:101[B]:HIS:HE1	5:B:501:HEM:O2D	2.02	0.43
2:C:107:LEU:HD11	2:C:222:ALA:HB2	2.00	0.43
4:F:55:VAL:HG13	4:F:60:ASP:HB2	2.01	0.43
3:E:67:ASP:OD1	3:E:69:ARG:HG3	2.19	0.43
1:A:356:CYS:HB3	1:A:359:ALA:HB2	2.02	0.42
1:D:191[A]:ARG:HD3	1:D:194[A]:GLN:HE21	1.82	0.42
1:D:239:VAL:HG12	1:D:243:ILE:CD1	2.49	0.42
3:E:108:VAL:HG12	3:E:215:LEU:HD22	2.01	0.42
3:E:168:ARG:HA	3:E:171:PHE:CE2	2.54	0.42
1:A:68:SER:HA	7:H:12:GOL:H12	2.00	0.42
1:B:105:ARG:NH2	1:B:355:HIS:O	2.53	0.42
1:D:55:VAL:HG13	1:D:60:ASP:HB2	2.02	0.42
2:C:362:GLY:O	2:C:366:LEU:HG	2.19	0.42
3:E:324:ALA:HB3	3:E:347:ILE:HD11	2.02	0.42
4:F:161:LEU:O	4:F:216:LEU:HB2	2.19	0.42
3:E:36[B]:ARG:HE	3:E:36[B]:ARG:HB2	1.52	0.42
2:C:165:LEU:HD12	2:C:165:LEU:HA	1.90	0.42
1:D:286:ARG:HG3	1:D:325:ASN:HB3	2.01	0.42
5:F:501:HEM:HBB2	5:F:501:HEM:CMB	2.48	0.42
1:A:17:TYR:O	1:A:46:PRO:HD3	2.19	0.42
1:A:219:LEU:O	1:A:223:THR:HG23	2.20	0.41
1:B:170:LEU:HD12	1:B:170:LEU:HA	1.89	0.41
2:C:106:ARG:NE	9:G:338:HOH:O	2.17	0.41
3:E:46:PRO:HB2	3:E:47:TYR:CD1	2.55	0.41
5:A:501:HEM:HBB2	5:A:501:HEM:CMB	2.48	0.41
2:C:42:ARG:HD3	9:G:96:HOH:O	2.20	0.41
1:D:170:LEU:C	1:D:170:LEU:HD23	2.41	0.41
4:F:271:VAL:HG11	4:F:378:PRO:HB3	2.01	0.41
4:F:382:LEU:HD23	4:F:382:LEU:HA	1.92	0.41
1:D:27[A]:LEU:HD12	1:D:27[A]:LEU:HA	1.83	0.41
2:C:276:LEU:HD12	2:C:276:LEU:HA	1.91	0.41
5:A:501:HEM:HBC2	5:A:501:HEM:CMC	2.50	0.41
1:A:215:LEU:HD23	1:A:215:LEU:HA	1.87	0.41

Continued on next page...

PRELIMINARY VALIDATION REPORT

Continued from previous page...

Atom-1	Atom-2	Interatomic distance (Å)	Clash overlap (Å)
1:D:205:ALA:O	1:D:208[B]:ARG:HB3	2.20	0.41
3:E:215:LEU:HD23	3:E:215:LEU:HA	1.79	0.41
1:A:331:PHE:CE1	1:A:345:PRO:HD2	2.56	0.41
1:A:44[B]:ARG:HE	1:A:44[B]:ARG:HB2	1.61	0.41
5:C:501:HEM:HAB	5:C:501:HEM:HHC	1.81	0.41
3:E:256:LEU:CD2	3:E:284[B]:MET:CB	2.81	0.41
1:A:42:ARG:CZ	1:A:51:THR:HG23	2.51	0.41
1:D:244:ALA:HB1	5:D:501:HEM:CHD	2.50	0.41
3:E:145:VAL:HA	3:E:149:ALA:HB3	2.01	0.41
4:F:121:ARG:N	4:F:122:PRO:HD2	2.36	0.41
1:A:28:ASP:HA	1:A:29:PRO:HD3	1.96	0.41
2:C:281:VAL:O	2:C:285:LEU:HG	2.21	0.40
1:B:123[B]:ARG:H	1:B:123[B]:ARG:HG3	1.66	0.40
1:B:191:ARG:HA	1:B:191:ARG:HD2	1.90	0.40
1:D:46:PRO:HB2	1:D:47:TYR:CE1	2.56	0.40
1:A:165[B]:LEU:HD23	1:A:165[B]:LEU:HA	1.97	0.40
1:B:203:LEU:HD23	1:B:203:LEU:HA	1.96	0.40
1:B:283:GLU:OE1	1:B:283:GLU:HA	2.22	0.40
1:D:236:ASN:OD1	7:H:4:GOL:H2	2.21	0.40

There are no symmetry-related clashes.

5.3 Torsion angles (i)

5.3.1 Protein backbone (i)

In the following table, the Percentiles column shows the percent Ramachandran outliers of the chain as a percentile score with respect to all X-ray entries followed by that with respect to entries of similar resolution.

The Analysed column shows the number of residues for which the backbone conformation was analysed, and the total number of residues.

Mol	Chain	Analysed	Favoured	Allowed	Outliers	Percentiles	
1	A	421/396 (106%)	405 (96%)	16 (4%)	0	100	100
1	B	416/396 (105%)	404 (97%)	12 (3%)	0	100	100
1	D	423/396 (107%)	403 (95%)	20 (5%)	0	100	100
2	C	406/398 (102%)	392 (97%)	14 (3%)	0	100	100
3	E	404/390 (104%)	393 (97%)	11 (3%)	0	100	100

Continued on next page...

PRELIMINARY VALIDATION REPORT

Continued from previous page...

Mol	Chain	Analysed	Favoured	Allowed	Outliers	Percentiles	
4	F	423/394 (107%)	406 (96%)	17 (4%)	0	100	100
All	All	2493/2370 (105%)	2403 (96%)	90 (4%)	0	100	100

There are no Ramachandran outliers to report.

5.3.2 Protein sidechains (i)

In the following table, the Percentiles column shows the percent sidechain outliers of the chain as a percentile score with respect to all X-ray entries followed by that with respect to entries of similar resolution.

The Analysed column shows the number of residues for which the sidechain conformation was analysed, and the total number of residues.

Mol	Chain	Analysed	Rotameric	Outliers	Percentiles	
1	A	358/332 (108%)	356 (99%)	2 (1%)	86	91
1	B	354/332 (107%)	343 (97%)	11 (3%)	40	52
1	D	361/332 (109%)	349 (97%)	12 (3%)	38	49
2	C	343/333 (103%)	334 (97%)	9 (3%)	46	58
3	E	345/327 (106%)	337 (98%)	8 (2%)	50	63
4	F	363/330 (110%)	345 (95%)	18 (5%)	24	33
All	All	2124/1986 (107%)	2064 (97%)	60 (3%)	49	56

All (60) residues with a non-rotameric sidechain are listed below:

Mol	Chain	Res	Type
1	A	93	VAL
1	A	196	PHE
1	B	44	ARG
1	B	93	VAL
1	B	123[A]	ARG
1	B	123[B]	ARG
1	B	127	LEU
1	B	171	PHE
1	B	196	PHE
1	B	309	VAL
1	B	326	ARG
1	B	343[A]	ARG
1	B	343[B]	ARG

Continued on next page...

PRELIMINARY VALIDATION REPORT

Continued from previous page...

Mol	Chain	Res	Type
2	C	10	PRO
2	C	86	THR
2	C	115	ARG
2	C	116	ARG
2	C	168[A]	ARG
2	C	168[B]	ARG
2	C	196	PHE
2	C	297	VAL
2	C	371	SER
1	D	12	ASP
1	D	44[A]	ARG
1	D	44[B]	ARG
1	D	116	ARG
1	D	178	MET
1	D	196	PHE
1	D	207	ARG
1	D	224	ASP
1	D	292	SER
1	D	312	ARG
1	D	318	VAL
1	D	374	VAL
3	E	42[A]	ARG
3	E	42[B]	ARG
3	E	191[A]	ARG
3	E	191[B]	ARG
3	E	196	PHE
3	E	215	LEU
3	E	264	ARG
3	E	318	VAL
4	F	93	VAL
4	F	110[A]	LYS
4	F	110[B]	LYS
4	F	115	ARG
4	F	132[A]	LEU
4	F	132[B]	LEU
4	F	196	PHE
4	F	207	ARG
4	F	264[A]	ARG
4	F	264[B]	ARG
4	F	276[A]	LEU
4	F	276[B]	LEU
4	F	312	ARG

Continued on next page...

PRELIMINARY VALIDATION REPORT

Continued from previous page...

Mol	Chain	Res	Type
4	F	333[A]	HIS
4	F	333[B]	HIS
4	F	335	ASP
4	F	404[A]	ILE
4	F	404[B]	ILE

Sometimes sidechains can be flipped to improve hydrogen bonding and reduce clashes. All (9) such sidechains are listed below:

Mol	Chain	Res	Type
1	A	193	GLN
1	A	340	HIS
1	B	258	HIS
2	C	206	GLN
1	D	360	GLN
3	E	206	GLN
3	E	236	ASN
3	E	351	HIS
4	F	194	GLN

5.3.3 RNA (i)

There are no RNA molecules in this entry.

5.4 Non-standard residues in protein, DNA, RNA chains (i)

There are no non-standard protein/DNA/RNA residues in this entry.

5.5 Carbohydrates (i)

There are no monosaccharides in this entry.

5.6 Ligand geometry (i)

59 ligands are modelled in this entry.

There are no bond length outliers.

There are no bond angle outliers.

There are no chirality outliers.

PRELIMINARY VALIDATION REPORT

There are no torsion outliers.

There are no ring outliers.

No monomer is involved in short contacts.

5.7 Other polymers (i)

There are no such residues in this entry.

5.8 Polymer linkage issues (i)

The following chains have linkage breaks:

Mol	Chain	Number of breaks
3	E	1
4	F	1

All chain breaks are listed below:

Model	Chain	Residue-1	Atom-1	Residue-2	Atom-2	Distance (Å)
1	E	208:ALA	C	213:GLU	N	11.18
1	F	272:ALA	C	275:ALA	N	5.48

PRELIMINARY VALIDATION REPORT

6 Fit of model and data (i)

6.1 Protein, DNA and RNA chains (i)

In the following table, the column labelled '#RSRZ> 2' contains the number (and percentage) of RSRZ outliers, followed by percent RSRZ outliers for the chain as percentile scores relative to all X-ray entries and entries of similar resolution. The OWAB column contains the minimum, median, 95th percentile and maximum values of the occupancy-weighted average B-factor per residue. The column labelled 'Q< 0.9' lists the number of (and percentage) of residues with an average occupancy less than 0.9.

Mol	Chain	Analysed	<RSRZ>	#RSRZ>2			OWAB(Å^2)	Q<0.9
1	A	396/396 (100%)	0.33	18 (4%)	33	30	35, 56, 83, 113	0
1	B	396/396 (100%)	0.13	10 (2%)	57	53	36, 54, 78, 159	0
1	D	396/396 (100%)	0.45	22 (5%)	24	20	40, 65, 94, 148	0
2	C	398/398 (100%)	0.10	4 (1%)	82	81	37, 48, 68, 123	0
3	E	390/390 (100%)	0.45	44 (11%)	5	3	44, 67, 97, 126	0
4	F	394/394 (100%)	0.94	76 (19%)	1	0	48, 75, 112, 135	0
All	All	2370/2370 (100%)	0.40	174 (7%)	15	11	35, 60, 98, 159	0

All (174) RSRZ outliers are listed below:

Mol	Chain	Res	Type	RSRZ
4	F	375[A]	ARG	7.6
2	C	10	PRO	6.8
4	F	124	VAL	6.6
4	F	405	VAL	6.5
4	F	129[A]	ASP	6.4
4	F	126	SER	6.4
4	F	271	VAL	6.4
4	F	127[A]	LEU	6.4
4	F	380[A]	LEU	6.2
4	F	270[A]	LEU	5.9
4	F	404[A]	ILE	5.5
1	B	209	ASP	5.4
4	F	130	SER	5.2
4	F	132[A]	LEU	5.1
4	F	407	TRP	5.0
4	F	142	ALA	5.0
4	F	125[A]	ARG	4.9
4	F	379	THR	4.8
4	F	131	LEU	4.8

Continued on next page...

PRELIMINARY VALIDATION REPORT

Continued from previous page...

Mol	Chain	Res	Type	RSRZ
3	E	313	ALA	4.8
3	E	305[A]	GLU	4.7
1	B	265[A]	LYS	4.7
4	F	370[A]	LEU	4.6
4	F	385	PRO	4.5
4	F	371	SER	4.5
4	F	272	ALA	4.4
3	E	36[A]	ARG	4.4
3	E	37	ASP	4.3
4	F	377	PHE	4.2
3	E	334	ALA	4.2
2	C	11	ALA	4.1
4	F	140	SER	4.1
1	D	265[A]	LYS	4.1
4	F	383	ALA	4.0
1	B	210	ALA	4.0
4	F	261	LEU	4.0
1	D	27[A]	LEU	3.9
3	E	33[A]	GLU	3.9
4	F	339	PHE	3.9
3	E	333	HIS	3.9
1	A	210	ALA	3.8
1	D	36[A]	ARG	3.8
4	F	133	ASP	3.8
4	F	330	VAL	3.8
3	E	336[A]	GLU	3.8
1	D	226	ASP	3.7
4	F	337	LEU	3.7
4	F	329[A]	GLU	3.7
4	F	141	PRO	3.6
1	D	406	SER	3.6
4	F	36[A]	ARG	3.6
4	F	328	GLU	3.6
3	E	307	SER	3.5
4	F	267	TYR	3.5
3	E	335	ASP	3.5
4	F	332	ASP	3.5
4	F	331	PHE	3.5
3	E	311	VAL	3.5
1	B	211	PRO	3.4
3	E	265[A]	LYS	3.4
3	E	32	ALA	3.4

Continued on next page...

PRELIMINARY VALIDATION REPORT

Continued from previous page...

Mol	Chain	Res	Type	RSRZ
4	F	139	GLY	3.4
1	D	209[A]	ASP	3.4
1	A	373	LEU	3.4
1	D	227	ASP	3.4
4	F	277	VAL	3.4
1	A	334	ALA	3.3
4	F	376	ARG	3.3
1	A	213[A]	GLU	3.3
4	F	123[A]	ARG	3.3
3	E	340	HIS	3.3
1	D	208[A]	ARG	3.2
4	F	406	SER	3.2
1	A	209[A]	ASP	3.2
4	F	147	PHE	3.2
4	F	364	LEU	3.2
1	D	267	TYR	3.1
3	E	29	PRO	3.1
1	B	224[A]	ASP	3.1
4	F	336[A]	GLU	3.0
4	F	138	HIS	3.0
4	F	374[A]	VAL	3.0
4	F	276[A]	LEU	3.0
1	D	224	ASP	3.0
3	E	306	LEU	3.0
3	E	263[A]	GLU	3.0
3	E	342[A]	GLU	3.0
4	F	333[A]	HIS	2.9
4	F	263[A]	GLU	2.9
3	E	267	TYR	2.9
4	F	382	LEU	2.9
1	A	208[A]	ARG	2.8
1	D	191[A]	ARG	2.8
2	C	343[A]	ARG	2.8
4	F	384[A]	GLU	2.8
4	F	262	THR	2.8
1	B	342[A]	GLU	2.7
4	F	128	VAL	2.7
3	E	310	THR	2.7
1	A	342[A]	GLU	2.7
4	F	32	ALA	2.7
1	B	36[A]	ARG	2.7
4	F	343[A]	ARG	2.7

Continued on next page...

PRELIMINARY VALIDATION REPORT

Continued from previous page...

Mol	Chain	Res	Type	RSRZ
1	D	333	HIS	2.6
1	B	335[A]	ASP	2.6
3	E	269	SER	2.6
4	F	225	ASN	2.6
3	E	382	LEU	2.6
4	F	29	PRO	2.6
3	E	312	ARG	2.6
4	F	208[A]	ARG	2.6
4	F	122	PRO	2.5
4	F	326	ARG	2.5
4	F	38[A]	GLU	2.5
1	D	223	THR	2.5
4	F	340	HIS	2.5
3	E	115[A]	ARG	2.5
4	F	44[A]	ARG	2.5
4	F	369	ALA	2.5
4	F	308	THR	2.5
4	F	373	LEU	2.4
1	D	263[A]	GLU	2.4
3	E	271	VAL	2.4
1	A	336[A]	GLU	2.4
4	F	268[A]	GLU	2.4
3	E	51	THR	2.4
4	F	264[A]	ARG	2.4
4	F	136	VAL	2.4
1	B	225[A]	ASN	2.4
3	E	388	GLY	2.4
3	E	213	GLU	2.4
1	A	380	LEU	2.4
3	E	23	HIS	2.4
4	F	37	ASP	2.3
1	A	44[A]	ARG	2.3
1	D	334	ALA	2.3
1	B	343[A]	ARG	2.3
3	E	332	ASP	2.3
3	E	309	VAL	2.3
3	E	42[A]	ARG	2.3
3	E	387	ALA	2.3
1	D	221	LEU	2.3
1	D	309	VAL	2.3
4	F	342	GLU	2.3
1	A	405	VAL	2.3

Continued on next page...

PRELIMINARY VALIDATION REPORT

Continued from previous page...

Mol	Chain	Res	Type	RSRZ
3	E	301	THR	2.3
4	F	215	LEU	2.3
1	A	262	THR	2.2
1	A	333	HIS	2.2
3	E	314	GLY	2.2
1	D	39	PRO	2.2
2	C	213[A]	GLU	2.2
3	E	338	ASP	2.2
4	F	327	ASP	2.2
4	F	335	ASP	2.2
1	A	328[A]	GLU	2.2
3	E	27	LEU	2.1
1	A	180	SER	2.1
3	E	35[A]	ARG	2.1
1	D	205	ALA	2.1
3	E	287	TYR	2.1
4	F	265[A]	LYS	2.1
1	A	224	ASP	2.1
3	E	343[A]	ARG	2.1
1	D	329[A]	GLU	2.1
3	E	69	ARG	2.1
1	A	335	ASP	2.1
1	D	106[A]	ARG	2.1
1	A	178	MET	2.0
1	D	222	ALA	2.0
4	F	402[A]	ARG	2.0
3	E	70	PHE	2.0
3	E	191[A]	ARG	2.0
3	E	328	GLU	2.0

6.2 Non-standard residues in protein, DNA, RNA chains (i)

There are no non-standard protein/DNA/RNA residues in this entry.

6.3 Carbohydrates (i)

There are no monosaccharides in this entry.

PRELIMINARY VALIDATION REPORT

6.4 Ligands (i)

In the following table, the Atoms column lists the number of modelled atoms in the group and the number defined in the chemical component dictionary. The B-factors column lists the minimum, median, 95th percentile and maximum values of B factors of atoms in the group. The column labelled 'Q< 0.9' lists the number of atoms with occupancy less than 0.9.

Mol	Type	Chain	Res	Atoms	RSCC	RSR	B-factors($Å^2$)	Q<0.9
8	FMT	J	2	3/?	0.60	0.85	80,80,85,90	0
7	GOL	H	12	6/?	0.62	0.48	69,71,81,82	0
8	FMT	J	24	3/?	0.69	0.31	81,81,84,94	0
7	GOL	H	13	6/?	0.71	0.21	73,89,95,104	0
8	FMT	J	29	3/?	0.74	0.24	76,76,78,83	0
8	FMT	J	33	3/?	0.74	0.22	59,59,64,65	0
8	FMT	J	21	3/?	0.76	0.87	85,85,91,91	0
8	FMT	J	28	3/?	0.77	0.23	74,74,80,85	0
8	FMT	J	10	3/?	0.78	0.17	78,78,80,80	0
8	FMT	J	31	3/?	0.79	0.21	67,67,77,81	0
8	FMT	J	18	3/?	0.80	0.30	82,82,89,90	0
8	FMT	J	26	3/?	0.80	0.20	88,88,91,95	0
8	FMT	J	4	3/?	0.81	0.31	71,71,77,77	0
8	FMT	J	1	3/?	0.81	0.50	75,75,81,84	0
8	FMT	J	16	3/?	0.82	0.24	68,68,77,84	0
8	FMT	J	23	3/?	0.82	0.56	90,90,90,94	0
8	FMT	J	5	3/?	0.83	0.19	84,84,89,89	0
8	FMT	J	25	3/?	0.84	0.39	79,79,83,84	0
8	FMT	J	17	3/?	0.84	0.10	84,84,91,100	0
8	FMT	J	32	3/?	0.85	0.10	85,85,86,92	0
8	FMT	J	13	3/?	0.85	0.34	76,76,80,87	0
8	FMT	J	3	3/?	0.86	0.45	64,64,67,81	0
7	GOL	H	9	6/?	0.86	0.16	71,88,95,96	0
7	GOL	H	4	6/?	0.88	0.27	69,74,77,78	0
7	GOL	H	8	6/?	0.88	0.23	51,66,72,80	0
8	FMT	J	8	3/?	0.88	0.17	62,62,64,69	0
8	FMT	J	30	3/?	0.89	0.09	88,88,88,92	0
8	FMT	J	12	3/?	0.89	0.16	77,77,85,90	0
7	GOL	H	2	6/?	0.90	0.25	55,58,59,60	0
8	FMT	J	27	3/?	0.90	0.25	72,72,73,77	0
7	GOL	H	1	6/?	0.91	0.34	50,58,67,72	0
8	FMT	J	11	3/?	0.91	0.23	60,60,65,68	0
7	GOL	H	14	6/?	0.91	0.15	80,83,87,88	0
8	FMT	J	9	3/?	0.91	0.18	76,76,78,88	0
8	FMT	J	14	3/?	0.91	0.63	79,79,81,83	0
7	GOL	H	11	6/?	0.92	0.16	58,59,62,69	0
8	FMT	J	7	3/?	0.92	0.27	72,72,75,77	0

Continued on next page...

PRELIMINARY VALIDATION REPORT

Continued from previous page...

Mol	Type	Chain	Res	Atoms	RSCC	RSR	B-factors(Å^2)	Q<0.9
8	FMT	J	19	3/?	0.92	0.12	65,65,75,79	0
7	GOL	H	10	6/?	0.93	0.29	70,82,84,87	0
8	FMT	J	15	3/?	0.93	0.27	77,77,78,83	0
7	GOL	H	5	6/?	0.93	0.28	57,59,60,68	0
8	FMT	J	6	3/?	0.93	0.22	76,76,78,90	0
6	DEB	F	502	27/?	0.93	0.18	50,61,66,67	0
6	DEB	E	502	27/?	0.93	0.17	47,53,58,60	0
6	DEB	D	502	27/?	0.94	0.20	44,50,60,64	0
7	GOL	H	6	6/?	0.94	0.20	58,65,70,72	0
8	FMT	J	20	3/?	0.94	0.21	49,49,52,57	0
5	HEM	F	501	43/?	0.95	0.14	48,60,69,84	0
6	DEB	C	502	27/?	0.95	0.20	35,40,45,49	0
7	GOL	H	7	6/?	0.95	0.21	54,58,59,59	0
7	GOL	H	3	6/?	0.96	0.20	50,54,57,58	0
5	HEM	B	501	43/?	0.97	0.20	30,36,46,52	0
5	HEM	E	501	43/?	0.97	0.15	38,47,57,61	0
5	HEM	A	501	43/?	0.97	0.19	31,38,43,63	0
6	DEB	A	502	27/?	0.97	0.23	38,40,49,53	0
6	DEB	B	502	27/?	0.97	0.20	39,41,44,47	0
8	FMT	J	22	3/?	0.98	0.11	51,51,53,54	0
5	HEM	D	501	43/?	0.98	0.19	30,38,54,58	0
5	HEM	C	501	43/?	0.98	0.18	32,38,42,57	0

6.5 Other polymers (i)

There are no such residues in this entry.

PRELIMINARY VALIDATION REPORT
